# Mechanochemical Syntheses of Ln(hfac)_3_(H_2_O)_*x*_ (Ln = La-Sm, Tb): Isolation of 10-, 9-, and 8-Coordinate Ln(hfac)_*n*_ Complexes

**DOI:** 10.1021/acs.inorgchem.2c01274

**Published:** 2022-07-27

**Authors:** Deepthi Y. Chappidi, Matthew N. Gordon, Hannah M. Ashberry, Junjie Huang, Bruce M. Labedis, Riley E. Cooper, Brandon J. Cooper, Veronica Carta, Sara E. Skrabalak, Kim R. Dunbar, Elisabeth M. Fatila

**Affiliations:** † Department of Chemistry and Physics, Louisiana Tech University, 1 Adams Blvd., Ruston, Louisiana 71272, United States; ‡ Department of Chemistry, Indiana University Bloomington, 800 E. Kirkwood Ave., Bloomington, Indiana 47405, United States; § Department of Chemistry, Texas A & M University, College Station, Texas 77842, United States

## Abstract

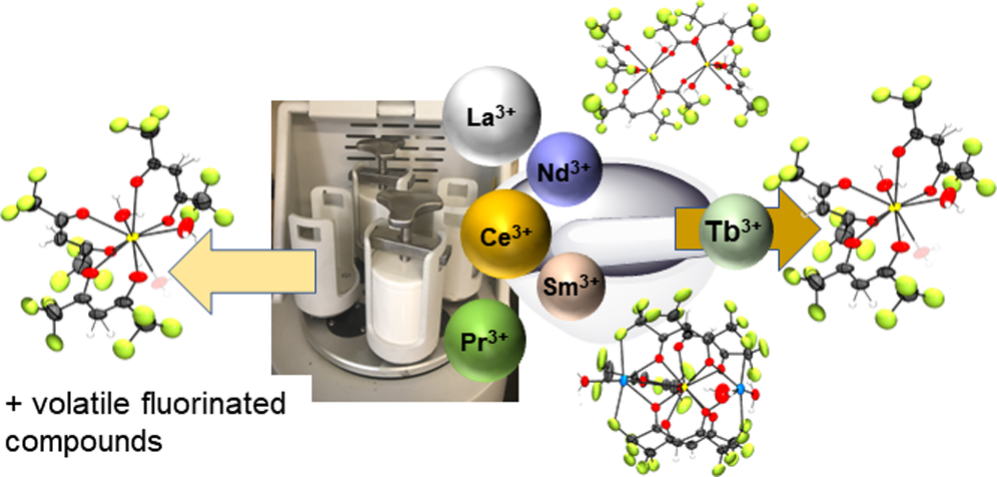

Volatile lanthanide coordination complexes are critical to the generation of new optical and magnetic materials. One of the most common precursors for preparing volatile lanthanide complexes is the hydrate with the general formula Ln(hfac)_3_(H_2_O)_*x*_ (*x* = 3 for La-Nd, *x* = 2 for Sm) (hfac = 1,1,1,5,5,5-hexafluoroacetylacetonato). We have investigated the synthesis of Ln(hfac)_3_(H_2_O)_*x*_ using more environmentally sustainable mechanochemical approaches. Characterization of the products using Fourier transform infrared spectroscopy, nuclear magnetic resonance spectroscopy, elemental analysis, and powder X-ray diffraction shows substantial differences in product distribution between methods. The mechanochemical synthesis of the hydrate complexes leads to a variety of coordination compounds including the expected hydrate product, the known retro-Claisen impurity, and hydrated protonated Hhfac ligand depending on the technique employed. Surprisingly, 10-coordinate complexes of the form Na_2_Ln(hfac)_5_·3H_2_O for Ln = La-Nd were also isolated from reactions using a mortar and pestle. The electrostatic bonding of lanthanide coordination complexes is a challenge for obtaining reproducible reactions and clean products. The reproducibility issues are most acute for the large, early lanthanides whereas for the mid to late lanthanides, reproducibility in terms of product distribution and yield is less of an issue because of their smaller size and greater charge to radius ratio. Ball milling increases reproducibility in terms of generating the desired Ln(hfac)_3_(H_2_O)_*x*_ along with hydrated Hhfac (tetraol) and free Hhfac products. The results illustrate the dynamic behavior of lanthanide complexes in solution and the solid state as well as the structural diversity available to the early lanthanides.

## Abstract 2

Mechanochemical reactions to prepare Ln(hfac)_3_(H_2_O)_*x*_ (Ln = La-Sm, Tb) complexes are used to illustrate the highly variable coordination chemistry of the early lanthanides compared to the mid-lanthanides. Using either a mortar and pestle or ball mill results in unexpected products and different product distributions. An open mortar and pestle can yield 10-coordinate pentakis-hfac complexes of La, Ce, Pr, and Nd, whereas ball milling consistently results in Ln(hfac)_3_(H_2_O)_*x*_ complexes with volatile Hhfac and hydrated Hhfac·2H_2_O (tetraol) that can be removed by vacuum.

## Introduction

Lanthanide β-diketonate complexes are often studied for their magnetic^1−5^ and optical properties.^6−8^ The versatility afforded by the variety of commercially available β-diketone ligands combined with the structurally isomorphous nature of lanthanide cations allows for delineation of structure–function relationships while also facilitating diverse applications.^9^ More specifically, the fluorinated β-diketone 1,1,1,5,5,5-hexafluoroacetylacetone (Hhfac) ligand imparts volatility.^10−12^ Although complexes of the type Ln(hfac)_3_(H_2_O)_*x*_ have been known for over 50 years,^13^ only the single-crystal structures of the later lanthanides Tb,^14^ Ho,^15^ Er,^16^ and Dy^17^ are currently known. The early lanthanides (La-Nd) have differing hydration numbers and are structurally different based on powder X-ray diffraction (PXRD) patterns compared to later lanthanides.^13^ Because of their larger ionic radius, the early lanthanides have greater variability in their coordination number and geometry than the later lanthanides. The result is that there can be great difficulty attempting to synthesize these materials reproducibly even when carefully controlling for stoichiometry. Although the synthesis of these compounds seems simple, there are complications on account of the labile nature of the ligands, their acid–base reactivity, ability to tautomerize, and electrophilicity.^9,18−20^ This results in the Hhfac ligand being able to undergo several side reactions including hydration to form a tetraol and retro-Claisen condensation to form trifluoroacetate.^13,21^

Mechanochemical synthesis has the advantage of reducing the use of solvent and potentially reducing losses of soluble starting materials and products. Mechanochemistry has been used to synthesize various lanthanide complexes including some containing β-diketonates^22−28^ as well as Ln-based metal–organic frameworks.^29^ Mechanochemical reactions can include small amounts of solvent, and the reaction can be described as either “liquid-assisted” or a “slurry” depending on the amount of solvent used relative to the mass of solid reagents.^30,31^ Reactions where a reagent is a liquid are still considered “neat” reactions and may not be considered “liquid-assisted”.^31^ Furthermore, when solvated compounds such as hydrates are used in mechanochemical reactions, these reactions are termed solvate-assisted.^32^ In order for a mechanochemical process to be sustainable and “green”, solvent reduction should happen at both the point of synthesis and during purification of the desired products. With this in mind, minimal amounts of solvent should be used for purification. Mechanochemistry can also reveal differences in reactivity and product distribution compared to the analogous reaction performed using conventional solution synthesis.^33^

Lanthanide coordination complexes are known for their dynamic coordination environment compared to transition metal chemistry. Conditions such as high humidity, recrystallization solvent, and time required for isolation can affect the product distribution and yield for lanthanide complexes.^34^ An underexplored benefit of mechanochemistry is the potential for discovery of compounds inaccessible through solution chemistry. We compare the products of solution and mechanochemical syntheses of these complexes using Fourier transform infrared spectroscopy (FT-IR), nuclear magnetic resonance (NMR), and XRD analysis. In doing so, we discovered that 10-coordinate complexes of the form Na_2_Ln(hfac)_5_·3H_2_O for the early lanthanides can be successfully isolated from certain mechanochemical reactions. Additionally, the first single-crystal structure of an early lanthanide trihydrate, Ce(hfac)_3_(H_2_O)_3_, was also obtained from a mechanochemical reaction using an open mortar and pestle. Using one-pot ball milling shows great promise for increasing yields and purity. The 9- and 10-coordinate structures of the early lanthanide illustrate the rich coordination chemistry and structural diversity accessible to the larger early lanthanides.

## Methods of Synthesis

For comparison to mechanochemical reactions, solution syntheses were conducted by first dissolving Na_2_CO_3_·H_2_O in water followed by the addition of Hhfac. After formation of the deprotonated ligand, LnCl_3_·7H_2_O was added and then the aqueous solution was extracted with 3 × 100 mL portions of Et_2_O. The Et_2_O layer was then dried using anhydrous MgSO_4_. The Et_2_O layer was then concentrated down to an oil, and hexanes were added to facilitate formation of a solid Ln(hfac)_3_(H_2_O)_*x*_ material. The work-up using Et_2_O extraction and hexanes was also applied to mechanochemical reactions.

Several types of mechanochemical reactions were performed in an analogous fashion to the solution reactions. Reactions described as M1-solvate assisted grinding (M1-SAG) were conducted in an open mortar and pestle. For these reactions, Na_2_CO_3_·H_2_O (1.5 equiv) was ground into a fine powder and spread out to maximize the surface area; Hhfac (liquid) (3 equiv) was then added and ground until a fine powder was obtained. Caution: Hhfac is a corrosive, volatile liquid. Care should be taken to ensure that only a minimal amount evaporates in the fumehood as this will affect yield. After formation of Na(hfac) in the first step, LnCl_3_·7H_2_O (1 equiv) was then added as a solid and ground until complete conversion from a viscous mixture to a solid, fine powder. For the “slurry” (M2) method, reactions were performed similarly to the M1 reaction in a mortar and pestle, except that LnCl_3_·7H_2_O was added alongside Et_2_O and ground for several minutes, and then, the small amount of solvent was allowed to evaporate. Hexanes were then added and ground for a few minutes and allowed to evaporate. Solvent amounts are detailed in the experimental portion of the Supporting Information. The time required ranges from 10 to 20 min as manual grinding is affected by the operator’s use of force and diligence as well as the flow rate of the fumehood and humidity; therefore, times are approximated. During our studies, we determined that the appearance of the reaction mixture as a free-flowing powder was the best indicator of reaction completion.

**Scheme 1:**
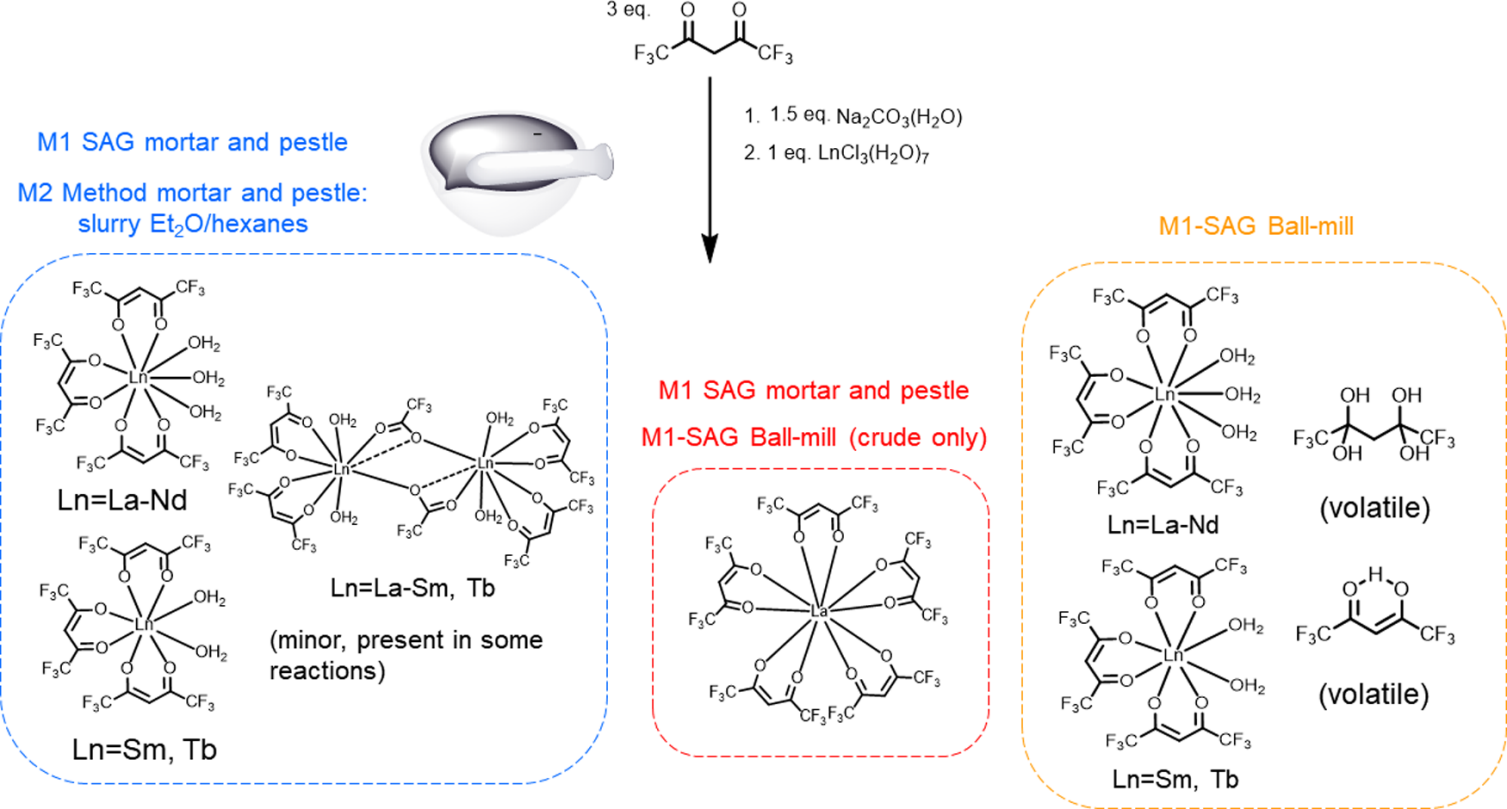
One-Pot Two-Step Synthesis Reactions of Ln Complexes with Main Product Distributions and Impurities for the Various Methods

**Scheme 2:**
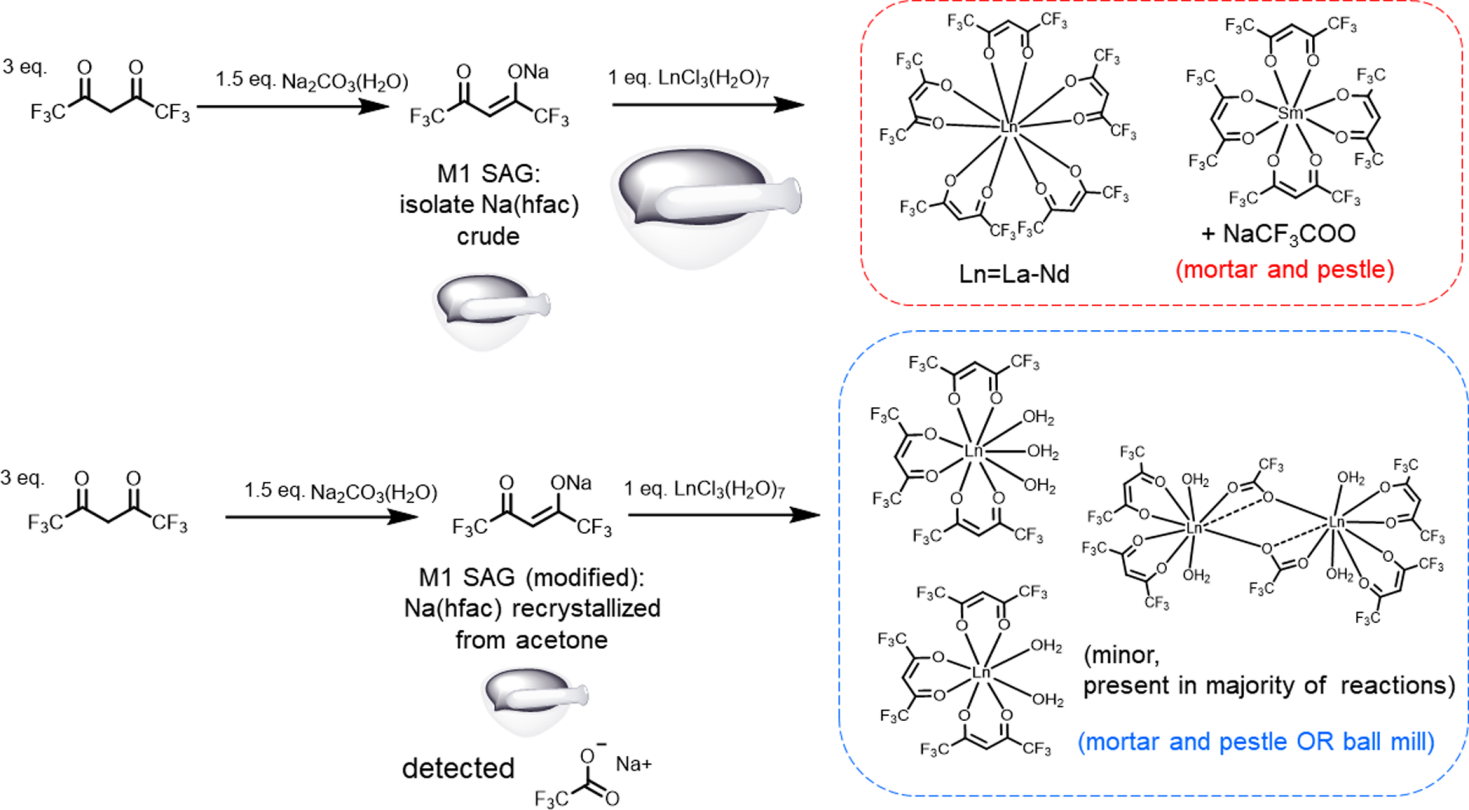
Two-Pot Two-Step Reactions of Ln Complexes with Main Product Distributions and Impurities Dependent on the Purity of Na(hfac) Used

To control for the evaporation of the free Hhfac ligand and manual use of force, ball-milled experiments were also carried out. The Hhfac (3 equiv) and Na_2_CO_3_·H_2_O (1.5 equiv) were combined first and ball-milled at 500 rpm for 20 min. Caution: the vessel should be opened slowly as there is built-up CO_2_. At this point, the material was completely converted to a fine white solid. LnCl_3_·7H_2_O (1 equiv) was then added and milled for 60 min at 500 rpm. The crude material was then extracted using a small amount of Et_2_O (25–30 mL) and concentrated down into an oil. A small amount (2–5 mL) of hexanes was occasionally added to facilitate formation of a solid. Because of the smaller amounts of diethyl ether needed for extraction, solid formation was often rapid. Additional control reactions using recrystallized 3 equiv Na(hfac) and LnCl_3_·7H_2_O with a high-power ball mill and mortar and pestle were also performed. These two sets of mechanochemical experiments can be further distinguished as one pot or two pot when isolated Na(hfac) was used. A general summary of the products from the different reactions is shown in Schemes 1 and 2.

## Results and Discussion

### Synthesis of 10-Coordinate Pentakis-hfac Complexes (La-Nd)

Lanthanide bonding is dominated by electrostatic interactions; therefore, yield and product purity are affected by subtle changes in ambient experimental conditions. The chemistry of the early lanthanides, which can accommodate more ligands and has a lower charge to radius ratio, is more prone to lack of stoichiometric control. Typically, access to tetrakis or even pentakis-hfac complexes has been through the addition of the required stoichiometric equivalents of β-diketonate to lanthanide.^13^ During our attempts to synthesize Ln(hfac)_3_(H_2_O)_3_ mechanochemically, we isolated pentakis-hfac complexes of the early lanthanides (La-Nd) and tetrakis-hfac complexes of Sm. Pentakis-hfac complexes were obtained inconsistently from the one-pot syntheses with 1:3 stoichiometry (Scheme 1), and we initially hypothesized that the humidity increased the hydration of the lanthanide chloride salts and increased their mass, therefore leading to an apparent reduction of moles of LnCl_3_·7H_2_O relative to Hhfac. We discovered that the pentakis- and tetrakis-hfac complexes were routinely obtained using the two-pot method with crude Na(hfac) in a 1:3 stoichiometry (Scheme 2). The product from our attempt to correct for the additional hydration of the LaCl_3_·7H_2_O salt yielded the same pentakis-hfac complex based on XRD and FT-IR data (Figures S12 and S13). The crude Na(hfac) contains excess cation (Na^+^) and basic carbonates that may hinder some amount of Ln^3+^ from reacting and leading to an effectively higher amount of available hfac. These factors would favor formation of the pentakis- and tetrakis-hfac assemblies (Section S2). Using recrystallized Na(hfac) affords neither the pentakis- nor tetrakis-hfac complexes in 1:3 stoichiometries. We note that two-pot mechanochemical reactions using recrystallized Na(hfac) do not yield the tetrakis product (Sm) even when an excess of recrystallized Na(hfac) is used (Figures S14 and S15). While the pentakis can be present in crude one-pot ball-milled reaction mixtures based on FT-IR and NMR, following extraction, the product is converted to Ln(hfac)_3_(H_2_O)_3_ along with tetraol and free Hhfac (*vide infra*).

For one-pot open mortar and pestle mechanochemical experiments, the volatility of Hhfac results in lower-than-expected stoichiometric equivalents of Na(hfac). This loss of Hhfac is dependent on the surface area of Na_2_CO_3_ and flow rate of the fumehood. In an open environment, this is not easy to control. Through several trials of repeating the first step of the M1-SAG reaction, it was determined that the yield of Na(hfac) that was soluble in acetone was 55–60%. For a trial involving thinly ground NaHCO_3_ spread over a larger surface area and covering the reaction with a watch glass to lower the rate of evaporation, the yield of the soluble material obtained was 70%. Therefore, for open mortar and pestle reactions, Na(hfac) could be considered the limiting reagent. Therefore, yields were calculated using the lanthanide starting material as the intended limiting reagent, which underestimates the yield. Yields were also calculated, assuming that 70% Hhfac reacted (Table S8 and Section S5a). This factor also explains why the pentakis-complexes are afforded occasionally for the one-pot M1-SAG reactions but consistently observed for the crude Na(hfac) reactions where the stoichiometry is partially corrected to account for the loss of Hhfac.

### Structural Characterization of 10-Coordinate Pentakis-hfac Lanthanide Complexes

**Figure 1:**
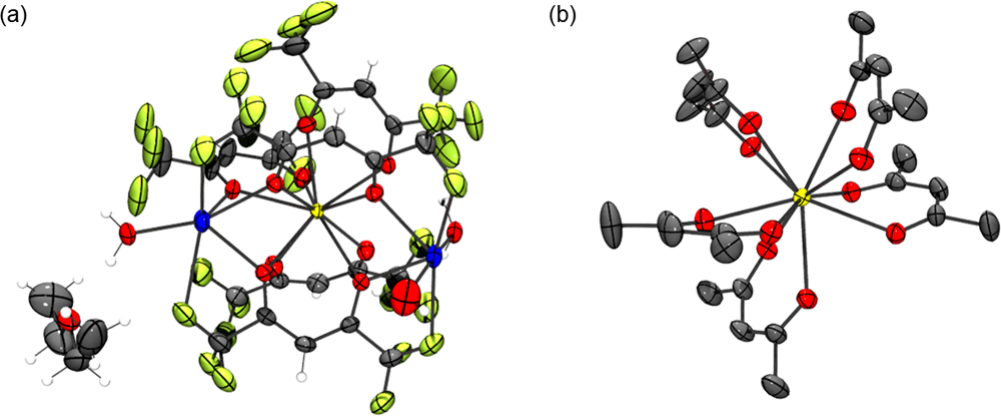
(a) Molecular structure of Na_2_Ce(hfac)_5_·3H_2_O·Et_2_O solvate collected at *T* = 173 K. Na^+^ (blue), Ce^3+^ (yellow), fluorine (yellow-green), carbon (gray), oxygen (red), and hydrogen (white). Disorder in the fluorine atoms and water was removed for clarity. The positions of the hydrogen atoms were calculated as riding on their respective atoms. (b) Molecular structure of [Ce(hfac)_5_]^2−^ with fluorine atoms removed for clarity. CCDC deposition no. 2107503. Ellipsoids plotted at 50% probability.

Crystals of the 10-coordinate pentakis-hfac complexes of the form Na_2_Ln(hfac)_5_·3H_2_O·Et_2_O were obtained for La-Nd. Previously, 10-coordinate complexes of La-Nd have been reported using pyridinium hfac,^13^ and a Cs_2_La(hfac)_5_ complex has also been reported.^35,36^ Two crystals, Na_2_Ce(hfac)_5_·3H_2_O·Et_2_O and Na_2_Pr(hfac)_5_·3H_2_O·Et_2_O (CCDC no. 2181477), had sufficient data quality to yield fully refined structures. All crystals have the same morphology and single-crystal determination, indicating that they have similar unit cells (Table S5). All crystals were grown from recrystallization in diethyl ether and hexanes. In the molecular structure of the 10-coordinate Ce coordination complex (Figure 1a), the central Ce^3+^ cation is coordinated to five deprotonated hfac ligands. Two Na^+^ counter cations are coordinated to the oxygens of the hfac and the fluorine atoms of the CF_3_ groups. The Na^+^ cations are also coordinated to water molecules. There is additional hydrogen bonding between these water molecules and an ether molecule. Figure 1b clearly shows the geometry about the Ce^3+^ ion showing coordination to five hfac ligands. The distance between Ce^3+^ and the nearest Na^+^ center is 3.731 Å. A dimer of the Na_2_Ce(hfac)_5_·3H_2_O·Et_2_O complex is formed through Na^+^ ions that coordinate to bridging water molecules. Pairs of these complexes form extended networks supported by hydrogen bonding through an H_2_O coordinated to the Na^+^ cation and an Et_2_O (Figure S18).

**Figure 2:**
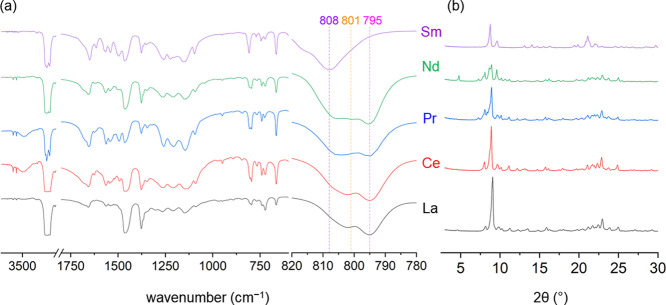
Comparison of (a) FT-IR (KBr, Nujol, 2 cm^–1^ res.) and (b) XRD patterns of Na_2_Ln(hfac)_5_·3H_2_O (La-Nd), NaSm(hfac)_4_ bulk materials.

The IR spectra of the pentakis-hfac products (Figure 2a) have subtle differences from the hydrates, primarily in the OH region. All 10-coordinate complexes from La-Nd have two sharp −OH peaks at 3716 and 3639 cm^–1^. The ∼800 cm^–1^ region is useful for determining the coordination number of the lanthanide for Ln(hfac)_*n*_L_*x*_ complexes.^26,37,38^ Lower values are associated with higher coordination numbers. For the La-Nd complexes in the IR spectrum, there is a peak at 795 cm^–1^ that is not observed in the corresponding 9-coordinate hydrates. The La-Nd complexes also have a shoulder peak at ∼801 cm^–1^ that increases in relative intensity to the 795 cm^–1^ from La to Nd. The Pr complex has an additional shoulder at 805 cm^–1^. The Nd complex has a broad band that contains peaks at 795, 801, and 805 cm^–1^. The IR spectrum of crystals of Na_2_Nd(hfac)_5_ has only a broad shoulder at 802 cm^–1^ (Figure S20), indicating that, because of its smaller size, the Nd bulk material may have a small population of the 9- and/or 8-coordinate complex. The Sm complex has one peak at 808 cm^–1^ and no peak at 795 or 800 cm^–1^, indicating that it is not a pentakis-hfac complex.

From analysis of the XRD patterns, the La, Ce, and Pr diffraction patterns appear similar with minor differences between 5 and 10° 2θ (Figure 2b). The Nd complex has extra peaks compared to La-Pr below 12° 2θ; however, above 15° 2θ, the Nd complex is extremely similar to La-Pr. This is consistent with primarily 10-coordinate complex formation with small amounts of the 9- or 8-coordinate tetrakis complex. The XRD results are corroborated by the IR data. For the Sm complex, the XRD pattern is completely different, indicating that it is unlikely to be a pentakis complex. The elemental analyses for La-Nd are consistent with formation of 10-coordinate pentakis-hfac complexes of the form Na_2_Ln(hfac)_5_·3H_2_O.

**Figure 3:**
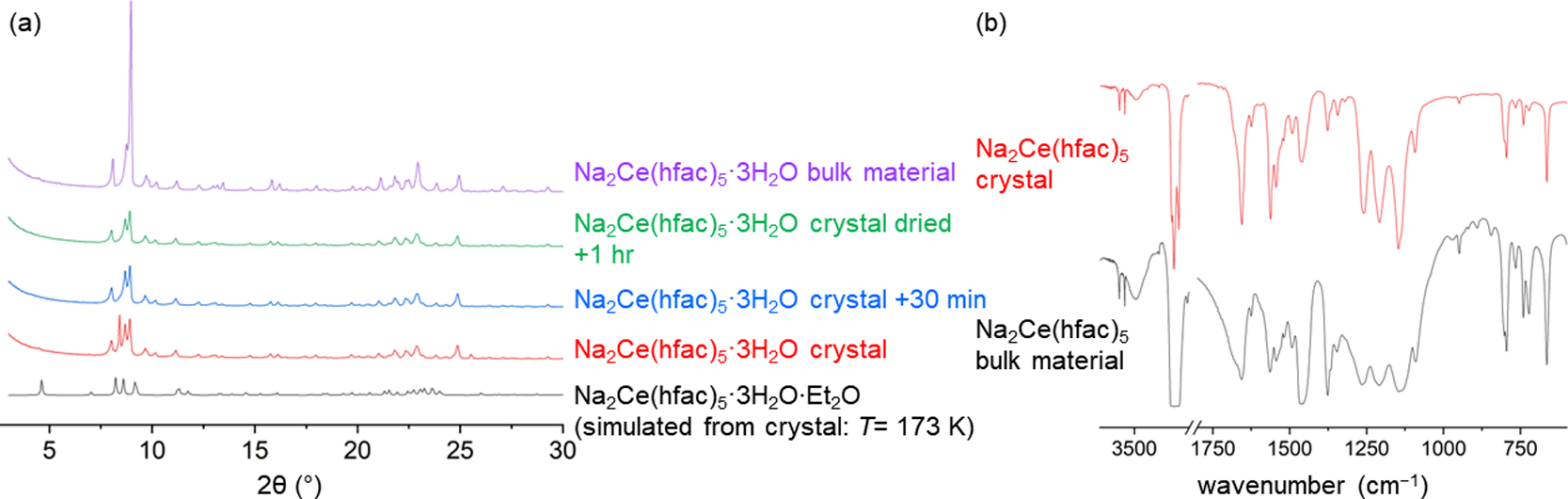
Comparison of (a) XRD patterns of simulated Na_2_Ce(hfac)_5_·3H_2_O·Et_2_O, desolvated crystal Na_2_Ce(hfac)_5_·3H_2_O, and bulk material Na_2_Ce(hfac)_5_·3H_2_O (from Figure 2b). (b) FT-IR spectra of bulk Na_2_Ce(hfac)_5_·3H_2_O (from Figure 2a) to IR of a single crystal of Na_2_Ce(hfac)_5_·3H_2_O (KBr, Nujol, 2 cm^–1^ res.).

One issue is that the simulated diffraction pattern generated from the single-crystal structure obtained at 173 K for Na_2_Ce(hfac)_5_·3H_2_O·Et_2_O does not match the diffraction patterns obtained from bulk PXRD measurements at room temperature because the single-crystal structure is a solvate. A crystal that was cut for single-crystal XRD was taken for powder XRD analysis (Figure 3a). A ground crystal of Na_2_Ce(hfac)_5_·3H_2_O does not match the simulated diffraction pattern. Over time, the crystal used for data collection begins to match more closely with the bulk un-recrystallized material. An IR spectrum of the crystal sample was recorded, and it overlaps well with the bulk material (Figure 3b).

### Synthesis and Structural Characterization of 9- and 8-Coordinate Samarium Complexes

**Figure 4:**
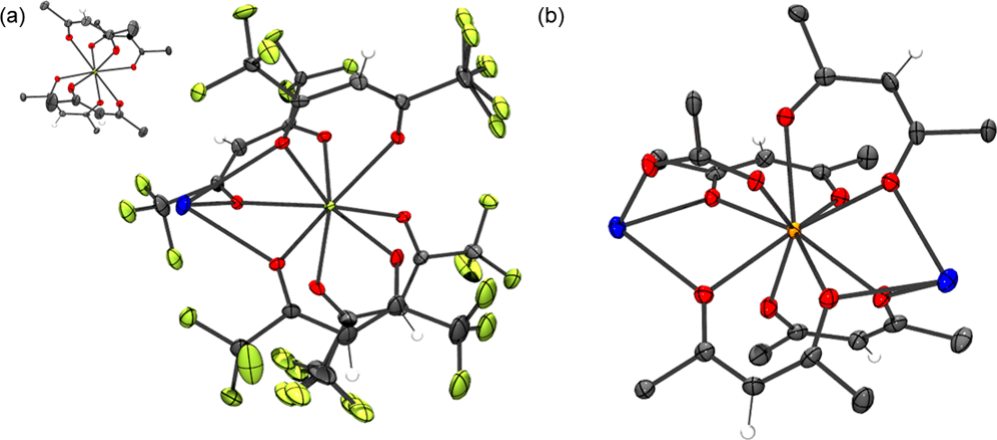
(a) Molecular structure of NaTb(hfac)_4_ (inset: coordination geometry about the Tb center). CCDC deposition no. 2181469. (b) Molecular structure of the NaSm(hfac)_4_NaTFA unit in a 1D chain structure with fluorine atoms removed for clarity. CCDC deposition no. 2181482. Disorder in the fluorine atoms was removed for clarity, ellipsoids plotted at 50% probability. Both structures were collected at *T* = 110 K. Terbium (light green), samarium (orange), fluorine (yellow-green), oxygen (red), sodium (blue), carbon (gray), and hydrogens (white). The position of the hydrogen atoms was calculated as riding on their respective atoms.

**Figure 5:**
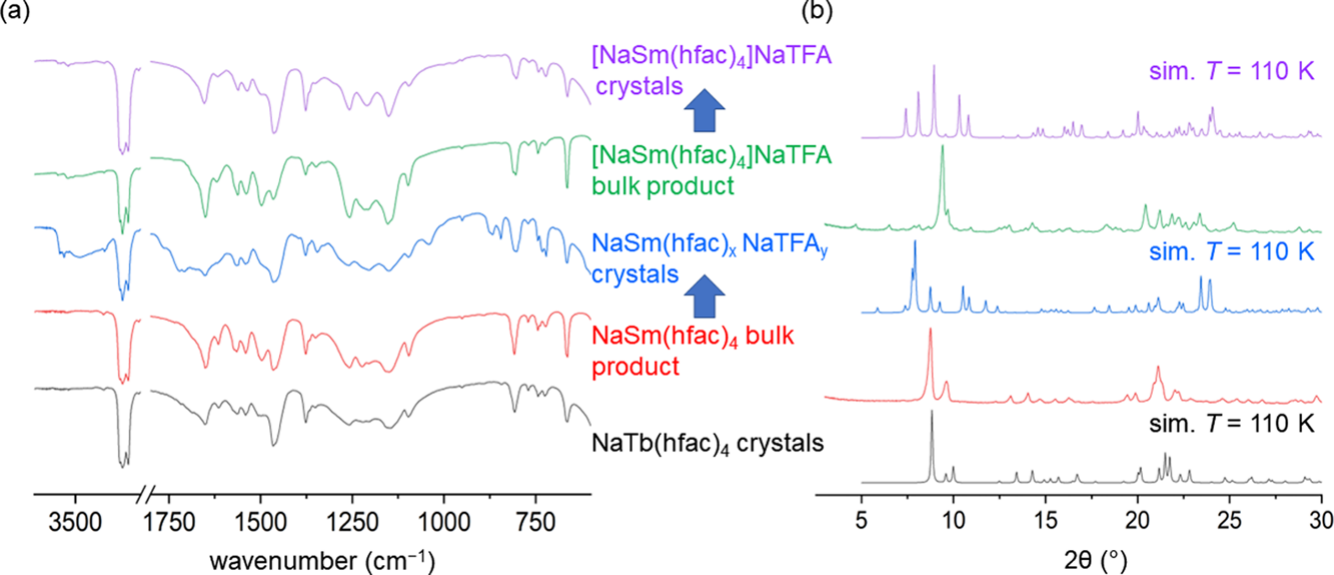
Comparison of (a) FT-IR spectra and (b) XRD patterns of 8-coordinate NaTb(hfac)_4_ and various NaSm(hfac)_4_NaTFA complexes. Bulk compounds and their crystallized counterparts are indicated with blue arrows. The NaSm(hfac)_4_ bulk product also used for comparison in Figure 2.

Unlike La-Nd, Sm forms a variety of tetrakis-hfac complexes, some of which contain the retro-Claisen impurity. NaTb(hfac)_4_ was also prepared (Section S2) as a means of obtaining a pure 8-coordinate tetrakis-hfac complex for comparison purposes. The single-crystal structure of NaTb(hfac)_4_ shows four hfac ligands coordinated to Tb with the oxygen and fluorine atoms of the hfac ligand also bridging a Na^+^ ion (Figure 4a). The NaTb(hfac)_4_ complex is polymeric and isostructural to other mid to late lanthanide complexes of the form NaLn(hfac)_4_.^39,40^ Based on XRD and IR studies, the Sm complex obtained using the same methodology and crude Na(hfac) starting material as the 10-coordinate pentakis-hfac complexes was an 8-coordinate complex of NaSm(hfac)_4_. The IR of the initial bulk NaSm(hfac)_4_ closely corresponds to the IR spectrum obtained for NaTb(hfac)_4_ crystals. Additionally, the XRD pattern is quite similar to the simulated NaTb(hfac)_4_ pattern with some small differences in position and broadness because of temperature differences in collection. An attempt to crystallize this compound resulted in crystals growing very slowly over 6 months. The IR spectrum of these crystals does not match the initial bulk product (Figure 5a). These crystals had insufficient data quality to afford a full refinement; however, the connectivity plot shows a complex network of bridging hfac and tfa ligands (Figure S21) and has been termed NaSm(hfac)_*x*_NaTFA_*y*_. The presence of a large amount of tfa is corroborated by the IR spectrum showing peaks at *ca*. 870 and 732 cm^–1^. Unlike La to Nd, an attempt to repeat the reaction of SmCl_3_·7H_2_O with crude Na(hfac) according to Scheme 2 resulted in a different product based on IR and XRD. This structure was fully refined and shows the tfa anion in a bridging motif between Na and Sm (Figure 4b). The presence of tfa is also indicated in the IR spectrum of the crystals (Figure 5a). This structure is a 9-coordinate Sm center coordinated by four hfac ligands and one bridging trifluoroacetate ligand (Figure 4b). This tfa ligand is also engaged in coordination with a Na^+^ cation. The other oxygen atom of the tfa anion is coordinated to two Na^+^ cations. The simulated XRD patterns of the obtained Sm crystals do not match the initial bulk product (Figure 5b).

### Synthesis of 9- and 8-Coordinate Ln(hfac)_3_(H_2_O)_*x*_ Complexes

While the M1-SAG method for synthesizing Ln(hfac)_3_(H_2_O)_*x*_ can lead to the surprising isolation of pentakis- and tetrakis-hfac lanthanide complexes, this method does not lead to the reproducible synthesis of Ln(hfac)_3_(H_2_O)_*x*_ complexes. The use of a closed environment for conducting mechanochemical reactions was expected to result in higher yields, more pure products, and greater reproducibility. For ball milling, the size of the milling material, time for each step, and speed can affect the product distributions of the reaction (Table S9). Following ball milling of the initial Na_2_CO_3_ and Hhfac, weighing of the resulting fine white solid indicated that negligible amounts of Hhfac evaporated. Following addition of LnCl_3_·7H_2_O, ball milling results in moderate yields and large amounts of Hhfac dihydrate (tetraol) based on ^1^H and ^19^F NMR, IR, and elemental analysis (*vide infra*). The use of excess base does not reduce the amount of tetraol impurity as expected (Figure S42 and Table S9). The presence of tetraol and free Hhfac is likely because, unlike the open mortar and pestle reactions, water cannot escape and will instead react with Hhfac. From one ball milling reaction, the pentakis complex was observed in the IR spectrum of the crude reaction mixture. Following extraction with Et_2_O, the crude pentakis complex converted to La(hfac)_3_(H_2_O)_3_ and tetraol as well as some free enol form of Hhfac (Figures S44 and S45). Less Et_2_O was required for extraction than the mortar and pestle reactions because of the greater surface area afforded by the milling material. Using less Et_2_O also reduced the amount of hexanes required for solidification. Details on optimization of the reaction and NMR yields for La(hfac)_3_(H_2_O)_3_ are provided in the Supporting Information.

When deprotonating Hhfac, even with a weak base such as Na_2_CO_3_ or NaHCO_3_ using a mortar and pestle, some trifluoroacetate material forms based on ^19^F and ^13^C NMR analyses (Figures S34, S48, and S49). Na(hfac) from ball milling reactions primarily contains the tetraol impurity; however, if placed under vacuum, then the tetraol and any free Hhfac can be removed (Section S6b). Using this purified material in a ball milling reaction results in cleaner La(hfac)_3_(H_2_O)_3_ based on ^13^C NMR analysis (Figure S58) compared to using the recrystallized Na(hfac) obtained from the mortar and pestle (Figure S51). While it would be satisfying to conclude that this is the major origin of the retro-Claisen impurity seen in the two pot syntheses (Scheme 2), La(hfac)_3_(H_2_O)_3_, essentially free of the retro-Claisen impurity (Figure S36) based on ^19^F NMR, was prepared from trifluoroacetate-contaminated Na(hfac) (Figure S34).

### Structural Characterization of Ln(hfac)_3_(H_2_O)_3_

**Figure 6:**
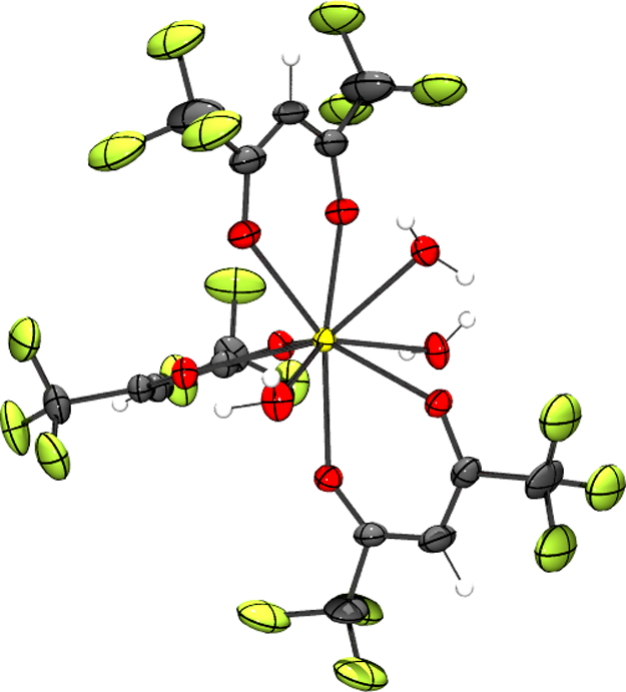
Molecular structure of Ce(hfac)_3_(H_2_O)_3_ collected at *T* = 123 K. Disorder in the fluorine atoms, water ligands, and the hfac backbone is removed for clarity. Ce^3+^ is shown in yellow, oxygen in red, carbon in gray, fluorine in yellow-green and hydrogen in white. The positions of the hydrogen atoms were calculated as riding on their respective atoms. Ellipsoids were plotted at 50% probability. CCDC deposition no. 2062848.

Despite Ln(hfac)_3_(H_2_O)_3_ being known for over 50 years, a published report of a single-crystal structure of any early trihydrate (La-Nd) has remained unavailable to the best of our knowledge. We obtained a single crystal of an early lanthanide trihydrate, Ce(hfac)_3_(H_2_O)_3_ (Figure 6), from a modified M1-SAG reaction (Section S5b) that was recrystallized by slow evaporation from diethyl ether and hexanes. The crystal structure has substantial disorder of the hfac ligands and the H_2_O ligands. The three H_2_O ligands coordinated to the Ce center do not have the same bond distance, with one H_2_O ligand being 0.03–0.05 Å longer. The Ce–O bond lengths are shorter in the Ce(hfac)_3_(H_2_O)_3_ structure than the Na_2_Ce(hfac)_5_·3H_2_O structure by approximately 0.1 Å. The carbonyl bond lengths are, on average, slightly longer for the Ce(hfac)_3_(H_2_O)_3_ complex than the Na_2_Ce(hfac)_5_·3H_2_O complex.

The single crystal of Ce(hfac)_3_(H_2_O)_3_ was used to generate simulated powder X-ray data^41^ to compare to the crude materials obtained from the experiment. Many of the PXRD patterns for the early lanthanides do not match closely with the simulated Ce(hfac)_3_(H_2_O)_3_ pattern. The lack of similarity between the simulated XRD and the experimental XRD of the trihydrates may be caused by differences in solvent incorporation between the single-crystal structure and the bulk materials obtained using different methods. Polymorphism and temperature-induced phase changes may also be contributing factors to the difference in diffraction patterns. To determine the presence of the dinuclear impurity in the PXRD, the preliminary structure of [Ce(hfac)_2_(CF_3_COO)(H_2_O)_2_]_2_ was obtained at 123 K. This structure has the same unit cell as the previously obtained [La(hfac)_2_(CF_3_COO)(H_2_O)_2_]_2_ (Refcode: KAMVAW).^42^

**Figure 7:**
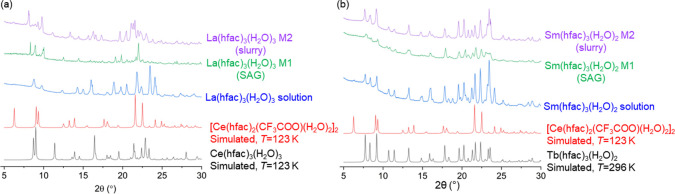
(a) Comparison of XRD patterns for La(hfac)_3_(H_2_O)_3_ (*T* = 296 K) prepared using various synthetic methods as well as the simulated diffraction pattern of [Ce(hfac)_2_(CF_3_COO)(H_2_O)_2_]_2_ and Ce(hfac)_3_(H_2_O)_3_ (λ = 1.54056 Å).^41^ (b) Comparison of XRD patterns for Sm(hfac)_3_(H_2_O)_2_ using different synthetic methods (*T* = 296 K, λ = 1.54178 Å) compared to Tb(hfac)_3_(H_2_O)_2_ (Refcode: NALCEL)^14^ and Ce cluster simulated (λ = 1.54056 Å).

From the XRD patterns, the differences between synthetic methods are especially acute for the early lanthanides (La-Nd) (Figure 7a and Section S9). In terms of morphology, the solution syntheses all yielded powders that have smaller grain sizes, whereas the mechanochemical synthesis generally yielded larger crystals that were found to occasionally be waxy in consistency. Focusing on the well-characterized La(hfac)_3_(H_2_O)_3_ complexes provides some rationale for the differences in XRD patterns observed. First, for the M1-SAG synthesis, a few of the La samples contain solvents based on ^1^H NMR and EA, which was not surprising based on their larger grain sizes than solution synthesis. The XRD patterns between the solution and M1-SAG synthesis are substantially different. Neither the solution nor the M1-SAG synthesis resembles the retro-Claisen XRD pattern for La. The solution and M2 (slurry) synthesis appear to have some retro-Claisen impurity as indicated by a small peak at *ca.* 6.2°(2θ). However, this does not mean that all M1-SAG syntheses or solution syntheses are free of this impurity. In addition, the 6.2° peak alone should not be used to assign the presence of the retro-Claisen impurity; confirmation should also be sought out from the IR spectrum (Tables S10 and S11 and Figure S66). From the modified M1-SAG methods of Ln(hfac)_3_(H_2_O)_3_ using recrystallized Na(hfac), the retro-Claisen impurity was found to be present based on samples that were taken for both single-crystal and powder XRD and IR analyses. Block crystals that were mechanically isolated and separated based on morphology were determined to be free of dinuclear impurity by PXRD and FT-IR (Figures S102 and S103). The presence or absence of the dinuclear impurity is further shown in comparing the different synthetic methods for Sm(hfac)_3_(H_2_O)_2_ (Figure 7b). On account of its smaller size, the Sm complex is a dihydrate and is isomorphous to the Tb(hfac)_3_(H_2_O)_2_ complex.^14^ However, the dinuclear retro-Claisen impurity expected for Sm^13^ would still be isomorphous to either the La^42^ or Ce dinuclear impurity.

**Figure 8:**
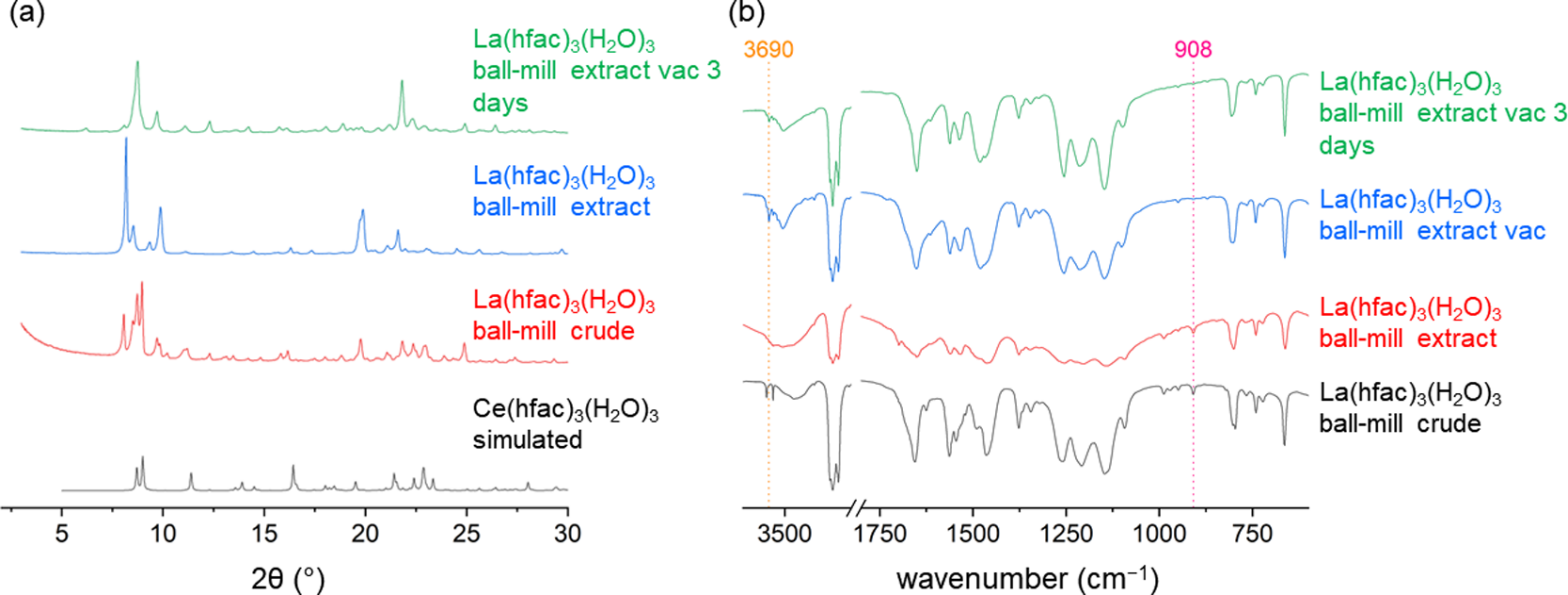
Optimized ball milling studies of La(hfac)_3_(H_2_O)_3_ showing (a) XRD diffraction patterns and (b) FT-IR spectra (Nujol, 2 cm^–1^ res.) following work up from the crude material to the extracted and vacuumed product.

For the optimized ball mill reaction, conversion from the pentakis-hfac complex to a product containing La(hfac)_3_(H_2_O)_3_ and tetraol was observed through XRD (Figure 8a) but was more difficult to interpret than FT-IR spectroscopy (Figure 8b). When the sample was vacuumed, a peak at 3690 cm^–1^ appeared in the IR spectrum and the peak at 908 cm^–1^ lost intensity. Performing ball milling results in readily identifiable fragments in the fingerprint region that indicate tetraol (Table S10 and Figure 8b). The IR fingerprint region is useful for illustrating the effect of removing the tetraol through vacuum.

### Mortar and Pestle Mechanochemical Synthesis of Tb(hfac)_3_(H_2_O)_2_

**Figure 9:**
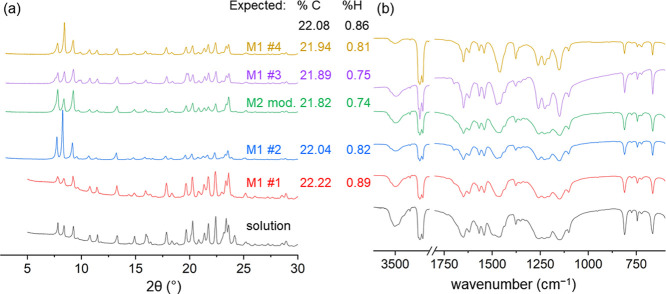
Comparison of reactions of solution (bottom) and repeated mortar and pestle mechanochemical reactions (M1-SAG and M2) to form Tb(hfac)_3_(H_2_O)_2_. (a) XRD patterns and (b) FT-IR (KBr, Nujol, 2 cm^–1^ res.) along with elemental analysis results. The top value (black) is the expected %C and %H values for Tb(hfac)_3_(H_2_O)_2_.

Compared to the early lanthanides, greater reproducibility is achieved when preparing complexes of Tb(hfac)_3_(H_2_O)_2_ from mechanochemical reactions using an open mortar and pestle. This greater reproducibility can be seen from comparison of yields and characterization through XRD patterns, IR spectra, and elemental analyses (Figure 9). Using TbCl_3_·6H_2_O, the open mortar and pestle reactions consistently yielded Tb(hfac)_3_(H_2_O)_2_ regardless of conditions. Careful analysis of the IR spectra of the products from multiple reactions showed small amounts of the retro-Claisen impurity in the M2 reaction that has the lowest %C and %H. However, all products were within ±0.3% elemental analysis. These reactions were conducted under different environmental conditions and with small variations. Ball milling for the Tb reactions results in tetraol impurities based on FT-IR spectra and elemental analysis, which shows high %C and high %H (Section S11). In a similar fashion to the La(hfac)_3_(H_2_O)_3_ sample, using a vacuum does reduce the presence of the tetraol.

## Conclusions

We investigated the mechanochemical synthesis of early Ln(hfac)_3_(H_2_O)_*x*_ complexes. In terms of reactivity, differences were found between the open mortar and pestle and ball-milled Ln(hfac)_3_(H_2_O)_3_. One of the surprises was the isolation of pentakis-hfac and tetrakis-hfac complexes from mechanochemical reactions, even though the volatility of Hhfac should result in lower than 3:1 equiv of Hhfac to Ln in an open mortar and pestle. While ball milling ameliorated the issue of Hhfac volatility, pentakis-hfac or tetrakis-hfac complexes were not isolated, rather they formed the intended Ln(hfac)_3_(H_2_O)_*x*_ product in moderate yield in addition to tetraol and free Hhfac. Fortunately, the tetraol and Hhfac can be removed using vacuum resulting in a purified product. These results demonstrate that the synthesis of Ln(hfac)_3_(H_2_O)_3_ complexes, where Ln = La-Nd in particular, could be described as low fidelity. Additionally, the Lewis acidic hfac ligand facilitates increased coordination of available ligands favoring 9- and 10-coordinate complexes. This implies that the 10-coordinate pentakis-hfac complex may be a relatively common intermediate in these reactions and is simply not isolated. Some of the challenges in reproducibility are inherent to the lanthanide. The larger ionic radius and lower charge to radius ratio of the early lanthanides cause the coordination environment of the lanthanide to be especially fluxional and difficult to control. The most reproducible synthesis for the early lanthanides was the formation of the coordinatively saturated 10-coordinate complexes with anionic ligands. The mid-lanthanide complex of Tb(hfac)_3_(H_2_O)_2_ was prepared using an open mortar and pestle and gave more consistent yields, pure materials by elemental analysis, with only minute amounts of retro-Claisen impurity detected in a minority of reactions by IR spectroscopy. Therefore, when characterizing Ln(β-diketonate)_*n*_ complexes, the IR spectrum is quite useful for identifying products and impurities even when the elemental analysis is consistent with the pure Ln(hfac)_3_(H_2_O)_*x*_ material. While the open mortar and pestle is a more accessible means of conducting mechanochemistry and can be a viable means of preparing Ln(hfac)_3_(H_2_O)_3_ complexes, ball milling is superior in providing reproducible preparation that is especially needed for the early lanthanides.

## Supporting Information Available

Additional information regarding synthetic details (La-Sm, Tb), additional control syntheses, ^1^H, ^13^C, and ^19^F NMR spectra, FT-IR spectra, TGA-DSC plots for hydrates and pentakis complexes, ball milling optimization, additional PXRD, and details on single-crystal collection (PDF)
