# Supplementary material for: Mechanochemical Syntheses of Ln(hfac)3(H2O)x (Ln = La-Sm, Tb): Isolation of 10-, 9-, and 8-Coordinate Ln(hfac)n Complexes
Source: Inorg Chem. 2022 Jul 27;61(31):12197–206. doi: 10.1021/acs.inorgchem.2c01274 (PMC9374134; doi:10.1021/acs.inorgchem.2c01274)
Supplement: Supplementary file 1 — ic2c01274_si_001.pdf [file ic2c01274_si_001.pdf]

# ***Mechanochemical Syntheses of Ln(hfac)$_3$(H$_2$O)$_x$ (Ln=La-Sm, Tb): Isolation of 10-, 9- and 8-Coordinate Ln(hfac)$_n$ Complexes***

*Deepthi Y. Chappidi*[†], *Matthew N. Gordon*[‡], *Hannah M. Ashberry*[‡], *Junjie Huang,*[§], *Bruce M. Labedis*[†], *Riley E. Cooper*[†], *Brandon J. Cooper*[†], *Veronica Carta*[‡], *Sara E. Skrabalak*[‡], *Kim R. Dunbar*[§], *Elisabeth M. Fatila**[†]

† Department of Chemistry and Physics, Louisiana Tech University, 1 Adams Blvd. Ruston, LA, 71272, USA

‡Department of Chemistry, Indiana University Bloomington, 800 E. Kirkwood Ave. Bloomington, IN, 47405, USA

§Department of Chemistry, Texas A & M University, College Station, TX 77842, USA

**S1. General Considerations** page SI-2 to SI-3

**S2. Synthesis of Na$_2$Ln(hfac)$_5$ and NaLn(hfac)$_4$ Complexes** page SI-4 to SI-22

Figures S1-S17

**S3a. Crystal Structure of Na$_2$Ce(hfac)$_5$·3H$_2$O·Et$_2$O**

Figures S18-S19 page SI-22 to SI-25

Tables S1 to S2

**S3b. Crystal structure of Na$_2$Pr(hfac)$_5$·3H$_2$O·Et$_2$O** page SI-25 to SI-27

Tables S3-S4

**S3c. Comparison of structural data for Na$_2$Ln(hfac)$_5$·3H$_2$O·Et$_2$O** page SI-28

Table S5

Figure S20

**S4a. Crystal structure of NaTb(hfac)$_4$** page SI-28 to SI-30

Table S6

**S4b. Crystal structure of NaSm(hfac)$_4$Na(TFA)** page SI-30 to SI-31

Table S7

**S4c. Preliminary structures and connectivity plot of Sm complex** page SI-32

Figure S21

**S5a. One-pot Hydrate Syntheses and Characterization (La to Sm)** page S1-32 to SI-49

Solution and mortar and pestle mechanochemical experiments

Figures S22-S32

Table S8

A note on the effect of sample preparation on IR peaks

**S5b. Mechanochemical Reactions with recrystallized Na(hfac)** page SI-49 to SI-57

Figures S33-S41

**S6a: One Pot Ball Mill Synthesis…** page SI-57 to SI-61

Figures S42-S46

Table S9

**S6b: Ball Mill Synthesis: $LnCl_3·7H_2O$ and Recrystallized Na(hfac)** page SI-62 to SI-70

Figures S47-S58

**S7. FT-IR Spectroscopy of $Ln(hfac)_3(H_2O)_x$ Complexes** page SI-71 to SI-78

Figures S59-S69

Tables S10-S11

Discussion about IR sample preparation and impurities

**S8. DSC-TGA Studies of $Ln(hfac)_3(H_2O)_x$ Complexes** page SI-79 to SI-88

Figures S70-S89

**S9: PXRD Studies of $Ln(hfac)_3(H_2O)_x$ Complexes** page SI-89 to SI-93

Figures S90-S97

**S10: Crystallographic Details for $Ce(hfac)_3(H_2O)_3$**

Figures S98-S103 page SI-93 to SI-99

Tables S12-S13

**S11: Solution and mechanochemical syntheses using $TbCl_3·6H_2O$** page SI-99 to SI-103

Figures S104-S105

**S12: References** page SI-104

## S1. General Considerations

$LnCl_3·7H_2O$ and $TbCl_3·6H_2O$ salts were purchased from Acros, Strem and Alfa Aesar and used as received. The ligand 1,1,1,5,5,5-hexafluoroacetylacetone (Hhfac) was purchased from Alfa Aesar and Acros and was used without any further work-up or purification. $Na_2CO_3·H_2O$ and $MgSO_4$ were obtained from Mallinckdrodt and used as received. Diethyl ether and hexanes were purchased from Fisher and used as received. The solution synthesis method employed is a modification of literature procedures.[1,2] $Nd(hfac)_3(H_2O)_3$ was purchased from Alfa Aesar and used as received. All reactions were conducted in a fumehood. IR spectra were collected at Louisiana Tech University on a Mattson Genesis II and a ThermoFisher Nicolet 6700 spectrometer using KBr plates (4000 to

600 $cm^{-1}$) with Nujol* (Acros) collecting 32 scans at a resolution of 2 $cm^{-1}$ unless stated otherwise. IR spectra were also collected at Texas A&M University on a Nicolet 470 using NaCl plates (4000 to 600 $cm^{-1}$) with Nujol* (Acros) collecting 32 scans at a resolution of 4 $cm^{-1}$. Wavenumbers in italics are peaks associated with known impurities. A vertical planetary ball mill (MSE supplies) was used for 1 g scale mechanochemical experiments along with 50 mL yttria-stabilized zirconia (YSZ) vessels. These reactions used either 3 or 5 mm diameter YSZ spherical grinding media from MSE supplies. A Beadbug D10 homogenizer with polypropylene 2 mL vials were used for the smaller scale recrystallized Na(hfac) + $LnCl_3 \cdot 7H_2O$ reactions. These reactions used either 1.4-1.7 mm or 3 mm diameter YSZ spherical grinding media from MSE supplies. $^1H$ NMR, $^{13}C$ NMR and $^{19}F$ NMR spectra were collected on a Bruker 400 MHz Avance Neo Spectrometer with $d_6$-acetone purchased from Acros Organics. For acetone-$d_6$, the $^1H$ NMR residual solvent signal was referenced to 2.05 ppm and the $^{13}C$ NMR C=O residual solvent signal was referenced to 206.26 ppm[3] unless otherwise indicated. Select $^{19}F$ NMR spectra were referenced with hexafluorobenzene (Alfa Aesar) (Table S10). Elemental analysis (EA) results were obtained from Midwest Microlabs (Indianapolis, IN) and Atlantic Microlabs (Atlanta, GA). At Indiana University-Bloomington, DSC and TGA was performed on a Setaram Labsys evo STA 1600 instrument using alumina crucibles under an argon atmosphere while heating at 3 K/min from 25°C to 350 °C. The evolved gases from the DSC/TGA instrument were analyzed by a Hiden QGA mass spectrometer. At Indiana University Bloomington, PXRD data was collected on a PANalytical Empyrean instrument (Cu $K_\alpha$, $\lambda$=1.54178 Å) and an X'Celerator linear strip detector under an applied voltage of 45 kV and 40 mA current. Using a rotating zero-background silicon holder, data was collected from 5 to 50° at a step size of 0.0167° (2θ). At Louisiana Tech, PXRD data was collected on a Rigaku Minflex 6G (Cu $K_\alpha$ source, $\lambda$=1.54178 Å) under an applied voltage of 40 kV and 15 mA current. Data was collected on a stainless steel 0.2 mm indent holder rotated at 80 rpm from 3 to 50° at a step size of 0.01° (2θ). Limited quantity samples were collected using a zero-background silicon holder. Simulated XRD patterns were calculated from single crystal data using Mercury software[4] assuming a Cu $K_{\alpha 1}$ source ($\lambda$= 1.54056 Å). Intensity for all XRD plots is in arbitrary units. Single crystal data was collected at the Indiana University Molecular Structure Center and Texas A&M University, details are provided in Sections **S3a to S4c and S10**. Renderings of refined crystal structures in the main text and supporting information were performed using ORTEP[5] and POVRAY[6].

## S2. Synthesis of $Na_2Ln(hfac)_5$ and $NaLn(hfac)_4$ complexes

### $Na_2La(hfac)_5 \cdot 3H_2O$ (M1-SAG one pot method) using 1:5 stoichiometry

To a porcelain mortar, $Na_2CO_3 \cdot H_2O$ (0.5584 g, 4.503 mmol) was added and ground lightly until powdery. Hhfac (1.4 mL, 10 mmol) was added and ground until a powdery solid. $LaCl_3 \cdot 7H_2O$ (0.739 g, 1.99 mmol) was added and ground for ~10 min until the material was a fine powder. The solid was extracted with 4×20 mL of $Et_2O$. The solution was concentrated down to a colorless oil. To the colorless oil, which was rapidly forming a precipitate, 10 mL of hexanes were added and 1.0027 g of white solid was obtained (0.7869 mmol, 40%). Yield assuming 70% Hhfac as limiting reagent (0.7869 mmol, 56%). FT-IR (KBr, Nujol): 3715vw-sp, 3638vw-sp, 3462vw-br, 3185w, 3142w, 1722mw, 1673mw-sh, 1655m, 1563m-br, 1544mw, 1529mw-sh, 1518w, 1492w, 1462* vs, 1366m, 1349m, 1345w-br, 1265ms, 1206ms, 1147m, 1093mw, 968w-br, 949w, 916vw, 889w, 845w, 801mw, **796mw**, 767w, 740mw, 664mw $cm^{-1}$. $^{1}H$ NMR (400 MHz, $d_6$-acetone, 298 K): 5.80 (s, presumed hfac-H), 2.98 (s, $H_2O$) ppm. $^{19}F$ NMR (376 MHz, $d_6$-acetone, 298 K, unreferenced): −76.19 (minor), −77.47 ppm. $^{13}C$ NMR (100 MHz, $d_6$-acetone, 298 K): 175.56 (q, $^{2}J_{C\text{-}F}$=32 Hz), 119.0 (q, $^{1}J_{C\text{-}F}$=286 Hz), 88.1 ppm. Elem. Anal. Calcd. for $Na_2La(hfac)_5 \cdot 3H_2O$ $LaNa_2C_{25}H_{11}F_{30}O_{13}$: %C 23.57, %H 0.87. Found %C 23.82, %H 0.87, duplicate %C 23.73, %H 0.80. This sample was recrystallized from diethyl ether and hexanes and produced single crystals of sufficient quality for X-ray diffraction. FT-IR of crystals (Nujol, NaCl): 3716mw-sp, 3639mw-sp, 3513mw-sh, 3446mw-br, 3307mw-sh, 3137w, 1676ms-sh, 1656s, 1624mw, 1563s, 1545ms, 1516mw, 1493mw, 1463*vs, 1367mw, 134m5w, 1320w, 1258s, 1210s, 1148s-br, 1092ms, 1043mw, 1019w, 976w-br, 949w, 932w, 916w, 889w, 842w, 829w, 802m-sh, **795mw**, 765w, 756vw, 740m, 723mw, 664ms.

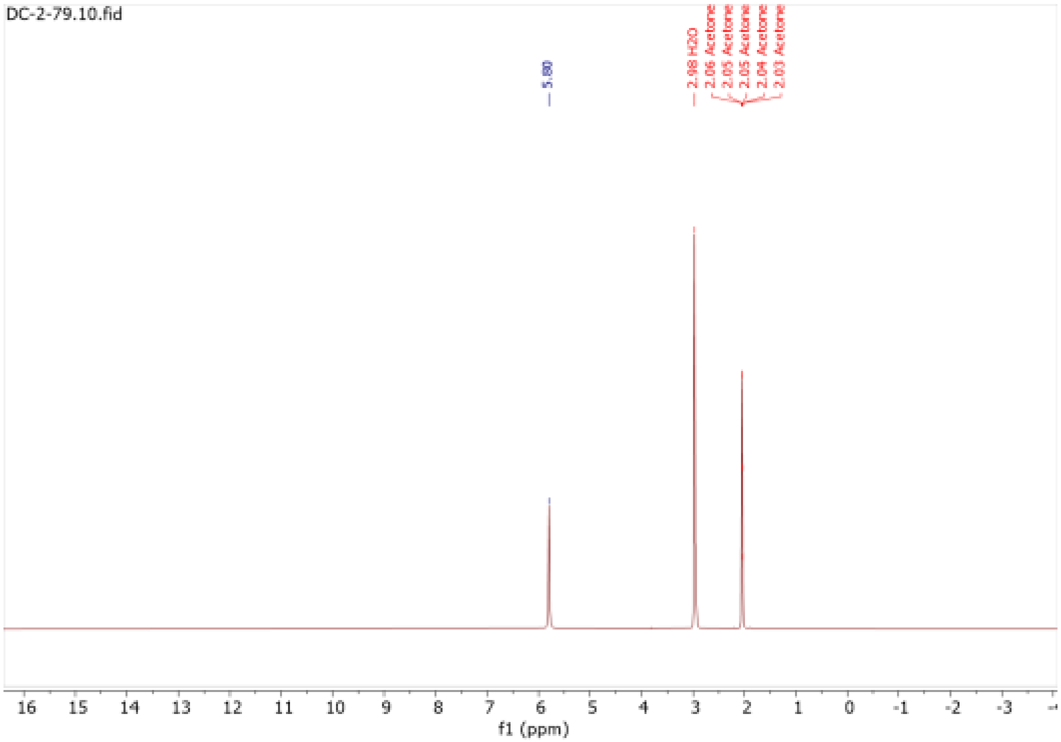


**Figure S1**: $^{1}H$ NMR of $Na_2La(hfac)_5{\cdot}3H_2O$ from one pot M1-SAG synthesis (298 K, 400 MHz, $d_6$-acetone).

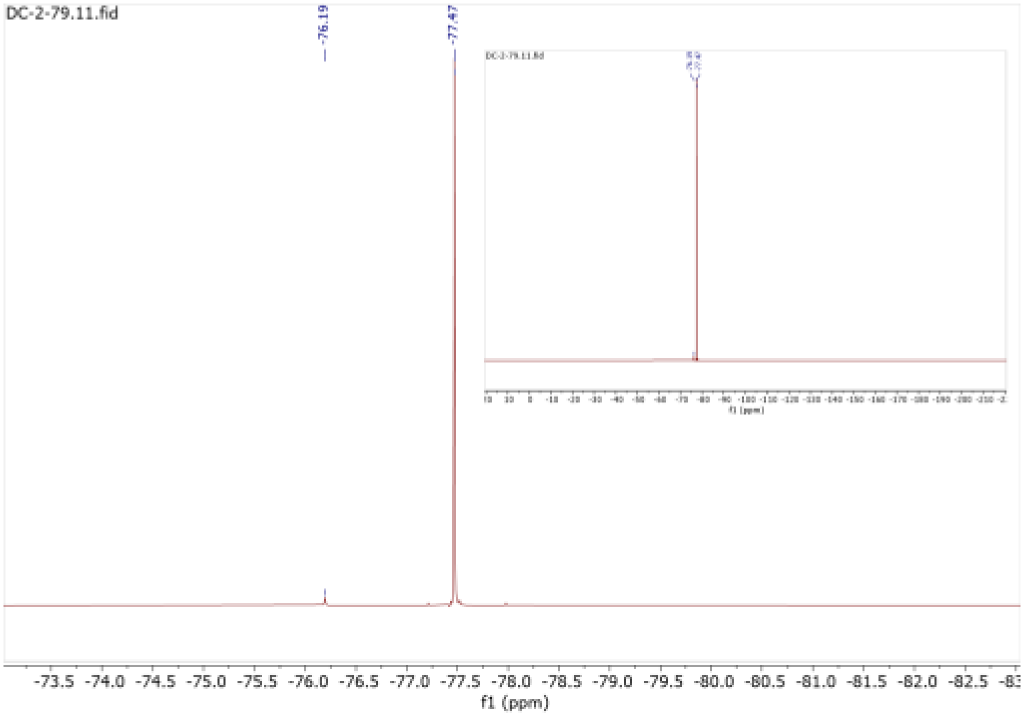


**Figure S2**: $^{19}F$ NMR of $Na_2La(hfac)_5{\cdot}3H_2O$ from one pot M1-SAG synthesis (298 K, 376 MHz, $d_6$-acetone).

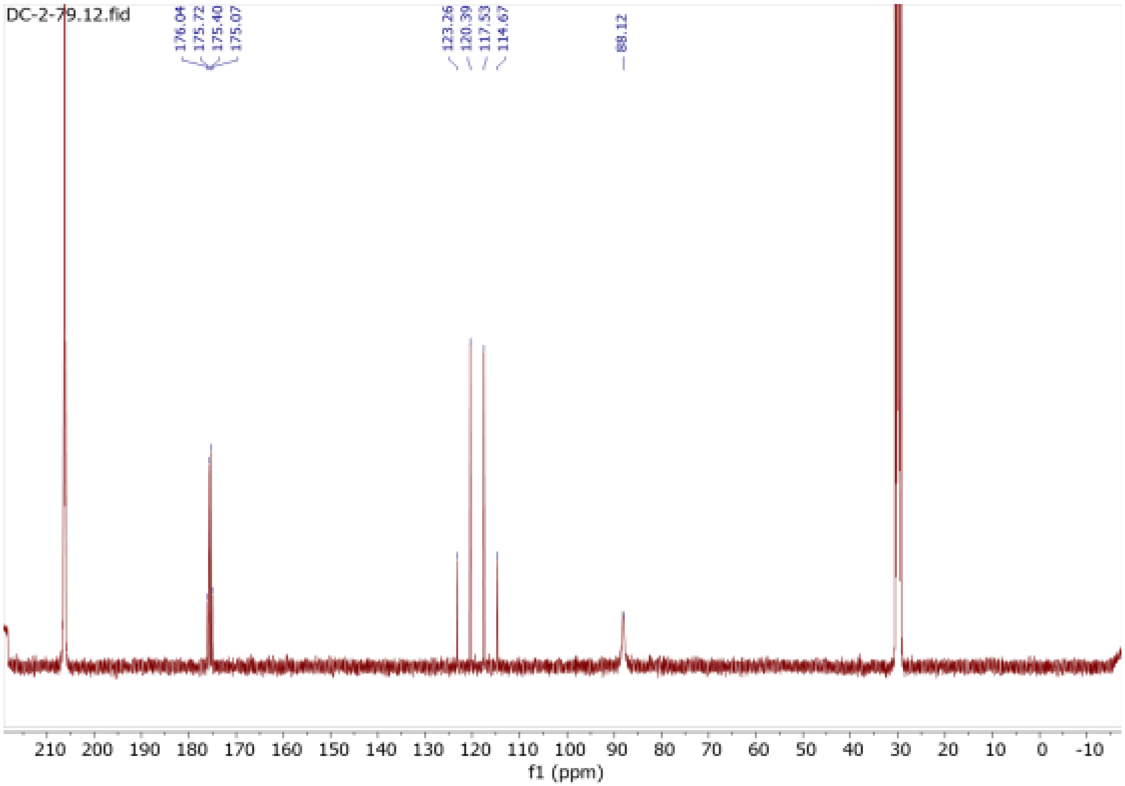

**Figure S3**: $^{13}$C NMR of $Na_2La(hfac)_5{\cdot}3H_2O$ from one pot M1-SAG synthesis (298 K, 100 MHz, $d_6$-acetone).

**Reaction to produce Na(hfac) unpurified:**

In a porcelain mortar, ground $Na_2CO_3{\cdot}H_2O$ (3.0045 g, 24.23 mmol) was ground until a fine powder. To this solid, was added Hhfac (6.8 mL, 49 mmol) and lightly ground until a fine white solid. The yield of this crude material was 6.6 g (59%). The material was used in the following reactions without any further purification. (assume monohydrate): %C 24.21, %H 1.22. Found %C 20.40, %H 1.22, duplicate %C 20.50, %H 1.06. The low percent carbon was expected because of the presence of inorganic impurities. Yields for complexes were calculated assuming anhydrous Na(hfac).

**Reaction with $LaCl_3{\cdot}7H_2O$:**

In a porcelain mortar, ground $LaCl_3{\cdot}7H_2O$ (0.6761 g, 1.820 mmol) and Na(hfac) (1.2533 g, 5.444 mmol) were ground together for 15 min. until a finely ground solid was obtained. This material was extracted with 4 portions of 10 mL of $Et_2O$. Upon concentrating down, a colorless oil was obtained. To facilitate formation of a solid, 5 mL of hexanes was added and concentrated down to obtain a white solid. The final yield was 46% (0.6338 g, 0.4974 mmol). FT-IR (KBr, Nujol):

3717w-sp, 3639w-sp, 3444vw-br, 3166w-br, 3143w, 1671mw-sh, 1656m, 1624w, 1563mw-br, 1540mw, 1527mw, 1515w, 1507w, 1462*vs, 1366s, 1349m, 1341m, 1301mw, 1266ms, 1209ms, 1148s, 1136s-sh, 1089m, 1044w, 969w-br, 948w, 934w-br, 918vw, 890w, 845w, 802mw, **795m**, 767w, 739m, 664m $cm^{-1}$. $^{1}H$ NMR ($d_6$-acetone, 298 K): 5.77 (s, hfac-H), 3.04 (s, $H_2O$) ppm. Additional small peaks appear to correspond to ethanol. $^{19}F$ NMR ($d_6$-acetone, 298 K, unreferenced): −76.07 (minor), −77.47, −77.98 (minor) ppm. $^{13}C$ NMR ($d_6$-acetone, 298 K): 175.4 (q, $^{2}J_{C\text{-}F}$=32 Hz), 118.9 (q, $^{1}J_{C\text{-}F}$=286 Hz), 87.6 ppm. Elem. Anal. Calcd. for (assume $Na_2La(hfac)_5{\cdot}3H_2O$) $Na_2LaC_{25}H_{11}F_{30}O_{13}$: %C 23.57, %H 0.87. Found %C 23.56, %H 0.85, duplicate %C 23.48, %H 0.78.

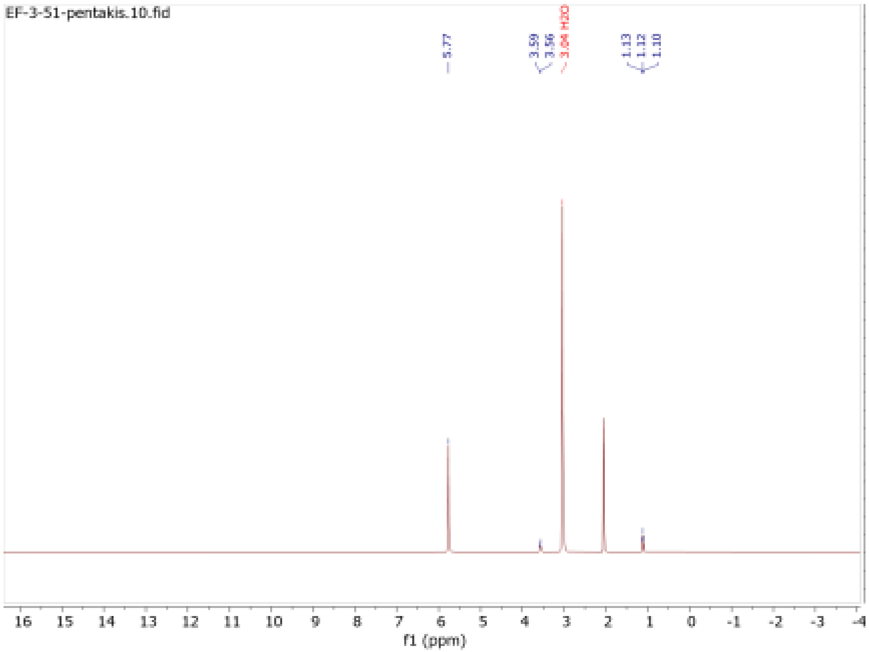


**Figure S4**: $^{1}H$ NMR of $Na_2La(hfac)_5{\cdot}3H_2O$ from M1-SAG synthesis with crude Na(hfac) (298 K, 400 MHz, $d_6$-acetone).

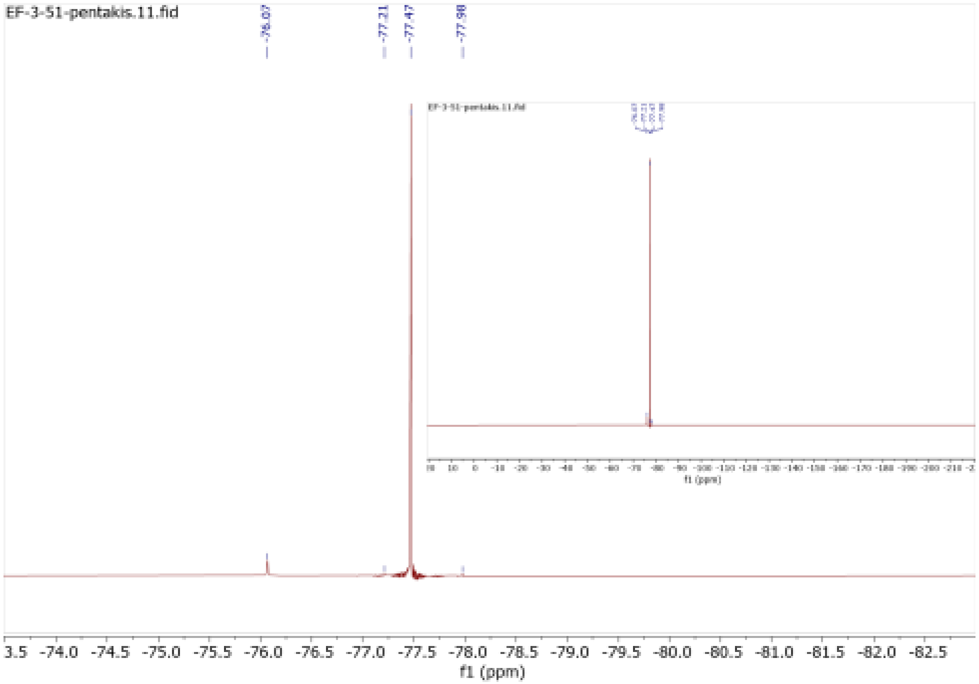


**Figure S5**: $^{19}$F NMR of $Na_2La(hfac)_5{\cdot}3H_2O$ from M1-SAG synthesis with crude Na(hfac) (298 K, 376 MHz, $d_6$-acetone).

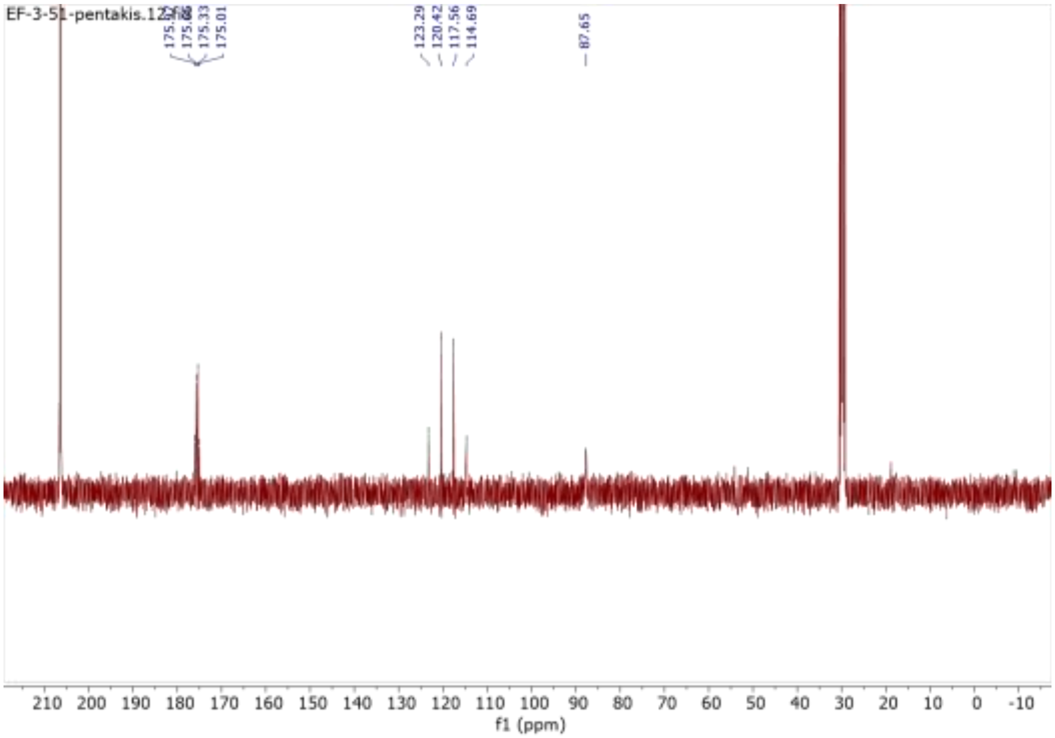


**Figure S6**: $^{13}$C NMR of $Na_2La(hfac)_5{\cdot}3H_2O$ from M1-SAG synthesis with crude Na(hfac) (298 K, 100 MHz, $d_6$-acetone).

**Reaction with $CeCl_3 \cdot 7H_2O$:**

In a porcelain mortar, ground $CeCl_3 \cdot 7H_2O$ (0.5500 g, 1.476 mmol) and Na(hfac) (1.0141 g, 4.408 mmol) were ground together for 20 min. until a finely ground solid was obtained. This material was extracted with 4 portions of 10 mL of $Et_2O$. Upon concentrating down, a yellow oil was obtained. To facilitate formation of a solid, 5 mL of hexanes was added and concentrated down to obtain a yellow solid. The final yield was 44% (0.4953 g, 0.3883 mmol). FT-IR (KBr, Nujol): 3717mw-sp, 3640mw-sp, 3473m-br, 3187w-br, 3139w, 1675ms-sh, 1656s, 1625mw, 1564s, 1544ms, 1517m, 1492mw, 1463*vs-br, 1367s, 1347m, 1322w, 1265s-br, 1209s-br, 1146s-br, 1135s-sh, 1090m, 972vw, 948w, 935w-br, 918vw, 889vw, 845w, 802m, **795ms**, 765w, 740mw, 664mw $cm^{-1}$. This sample was recrystallized from diethyl ether and hexanes and produced single crystals of sufficient quality for X-ray diffraction. Elem. Anal. Calcd. for $Na_2CeC_{25}H_{11}F_{30}O_{13}$: %C 23.54, %H 0.87. Found %C 23.08, %H 1.05, duplicate %C 22.95, %H 0.96,

**Reaction with $PrCl_3 \cdot 7H_2O$:**

In a porcelain mortar, ground $PrCl_3 \cdot 7H_2O$ (0.5537 g, 1.4814 mmol) and Na(hfac) (1.0160 g, 4.4166 mmol) were ground together for 20 min. until a finely ground solid was obtained. This material was extracted with 4 portions of 10 mL of $Et_2O$. Upon concentrating down, a pale green oil was obtained. To facilitate formation of a solid, 8 mL of hexanes was added and concentrated down to obtain a light green solid. The final yield of this product was 49% (0.5527 g, 0.4331 mmol). FT-IR (KBr, Nujol): 3717mw-sp, 3639mw-sp, 3473mw-br, 3413w-sh, 3309w-sh, 3139w, 1671m-sh, 1653s, 1623w, 1613w-s, 1563s, 1541ms, 1517w, 1493m-sh, 1464*vs, 1365w, 1345w, 1321w, 1258s, 1221s-sh, 1207s, 1146s, 1094mw, 1045w, 949w, 935vw, 918w, 890w, 843w, 804m, **795m**, 766w, 741mw, 725w, 664m $cm^{-1}$. Elem. Anal. Calcd. for $Na_2PrC_{25}H_{11}F_{30}O_{13}$: %C 23.53, %H 0.87. Found %C 23.55, %H 0.77, duplicate %C 23.66, %H 0.77. This sample was recrystallized from diethyl ether and hexanes and produced single crystals of sufficient quality for X-ray diffraction. FT-IR (Nujol, NaCl): 3716w, 3669w, 3637w, 3440w-br, 3133w, 1718mw, 1676ms, 1655ms-sh, 1612w, 1553m-sh, 1536m, 1514mw, 1423m-sh, 1464*vs, 1367mw, 1340w, 1319w, 1260s, 1206s, 1147s, 1095m, 1082mw, 970w, 948w, 932vw, 916vw, 888vw, 846w, 801m, **795m-sh**, 772w, 764w, 740mw, 725*mw, 664ms.

**Reaction with $NdCl_3 \cdot 7H_2O$:**

In a porcelain mortar, ground $NdCl_3 \cdot 7H_2O$ (0.6201 g, 1.646 mmol) and Na(hfac) (1.1238 g, 4.885 mmol) were ground together for 15 min. until a finely ground solid was obtained. This material was extracted with 4 portions of 10 mL of $Et_2O$. Upon concentrating down, a lilac oil was obtained. To facilitate formation of a solid, a total of 10 mL of hexanes was added and concentrated down to obtain a lilac solid. The final yield was 34% (0.4268 g, 0.3356 mmol). FT-IR (KBr, Nujol): 3717w-sp, 3639w-sp, 3456vw-br, 3185w-br, 3143w, 1677m-sh, 1657ms, 1623w, 1614w, 1565ms, 1541m, 1517m, 1494mw, 1462*vs, 1366m, 1348mw, 1261s-br, 1207s-br, 1146s-br, 1093mw, 1043w, 970vw-br, 948w, 933vw, 918vw, 889vw, 879vw, 842vw, 805m, 801m, **795m**, 765w, 741mw, 725w, 664ms $cm^{-1}$. Elem. Anal. Calcd. for $Na_2NdC_{25}H_{11}F_{30}O_{13}$: %C 23.47, %H 0.87. Found %C 23.74, %H 0.62, duplicate %C 23.72, %H 0.53.

**Reaction with $SmCl_3 \cdot 7H_2O$:**

In a porcelain mortar, ground $SmCl_3 \cdot 7H_2O$ (0.6543 g, 1.709 mmol) and Na(hfac) (1.1752 g, 5.108 mmol) were ground together for 10 min. and then allowed to sit for 15 minutes until dry and a finely ground beige solid was obtained. This material was extracted with four portions of $Et_2O$ (10 mL, 15 mL, 10 mL, 5 mL). Upon concentrating down, a pale yellow oil was obtained. To facilitate formation of a solid, a total of 15 mL of hexanes was added and concentrated down to obtain a pale yellow solid. The final yield was 29% (0.3731 g, 0.3725 mmol). FT-IR (KBr, Nujol): 3158vw-br, 3144vw, 1651s, 1615m, 1570m, 1565m, 1540m, 1496ms, 1465*vs, 1457vs, 1365mw, 1350mw, 1258s, 1223s, 1204ms-br, 1158vs-sh, 1149s-br, 1096ms, 972vw-br, 950w, 936vw-br, 917vw, 889vw-br, 843vw, **808m**, 770w, 744mw, 738mw-sh, 666ms-sh, 664ms. Elem. Anal. Calcd. for $NaSm(hfac)_4$ $NaSmC_{24}H_4F_{24}O_8$: %C 23.98, %H 0.40. Found %C 23.71, %H 0.59. This sample was recrystallized by slow evaporation from diethyl ether and hexanes to yield single crystals for X-ray diffraction. FT-IR of crystals (NaCl, Nujol/Paratone): 3690mw-sp, 3638mw, 3588mw-br, 3436mw-br, 3299mw, 3143mw, 3123mw-sh, 1803w-sh, 1759mw-br, 1720ms, 1708ms, 1685ms-sh, 1675ms-sh, 1670ms-sh, 1649s, 1631m-sh, 1616m-sh, 1565m, 1541m, 1535m-sh, 1465*vs-br, 1459vs-sh, 1395w, 1367w, 1345mw, 1327w-sh, 1260s-br, 1221s-sh, 1205s-br, 1150s, 1097m, 1039mw, 1003w-sh, 967vw-br, 950w, 918vw, 875mw-sh, *868mw*, 845mw, 803m-br, 770w, 744mw, *732m*, 665ms $cm^{-1}$.

**Repeat reactions to form pentakis and tetrakis complexes from crude Na(hfac)**

**Reaction to produce Na(hfac) unpurified:**

In a porcelain mortar, ground $Na_2CO_3·H_2O$ (3.0075 g, 24.254 mmol) was ground until a fine powder. To this solid, was added Hhfac (6.8 mL, 49 mmol) and lightly ground until a fine white solid. The yield of this crude material was 7.10 g (63%). The material was used in the following reactions without any further purification. FT-IR (KBr, Nujol): 3708w-sh, 3690w, 3520mw, 3391mw-br, 3135w-br, 1674s, 1652ms, 1621w-br, 1555ms, 1536ms-sh, 1531ms, 1494vs, 1463*s, 1407w, 1366mw, 1336w, 1318vw-sh, 1257s, 1217s, 1194s, 1140vs, 1080mw, *988w*, 970vw, 947vw, *908w*, *865w-br*, 847w, 833w, 822w, 798m-sh, 795m, 771w-br, 758w, 739w, 686vw, 665m $cm^{-1}$. $^1$H NMR ($d_6$-acetone, 298 K): 5.60 (s, hfac-H), 3.21 (s, $H_2O$) ppm. Additional small singlets are seen at 1.78, 1.56, 1.48, 1.42, 1.22 ppm. $^{19}$F NMR ($d_6$-acetone, 298 K, unreferenced): −75.8 (minor), −77.5, −85.9 (minor), −86.2 (minor), −88.0 (minor) ppm. $^{13}$C NMR ($d_6$-acetone, 298 K): 174.8 (q, $^2J_{C\text{-}F}$=30 Hz), 118.9 (q, $^1J_{C\text{-}F}$=288 Hz), 84.9 ppm. Additional peaks are observed that belong to a small amount of retro-Claisen impurity: 161.6 (q, $^2J_{C\text{-}F}$=32 Hz), 117.8 (q, $^1J_{C\text{-}F}$=292 Hz).

**Reaction to generate $Na_2La(hfac)_5·3H_2O$**

In a porcelain mortar, ground $LaCl_3·7H_2O$ (0.3934 g, 1.059 mmol) and Na(hfac) (0.7221 g, 3.139 mmol) were ground together for 15 min. until a finely ground solid was obtained. This material was extracted with 4 portions of 10 mL of $Et_2O$. Upon concentrating down, a colorless oil was obtained. To facilitate formation of a solid, 5 mL of hexanes was added and concentrated down to obtain a white solid. The final yield was 22% (0.1758 g, 0.1380 mmol). FT-IR (KBr, Nujol): 3716w-sp, 3639w-sp, 3448mw-br, 3137w, 1673mw-sh, 1656s, 1624w, 1611vvw, 1563s-br, 1556mw-sh, 1545m, 1536mw-sh, 1531mw-sh, 1519w, 1494mw, 1462*vs-br, 1366w, 1344mw, 1320w, 1258s-br, 1210s, 1148vs, 1092mw, 949w, 932vw, 917vw, 889vw, 843vw, 802mw, **796m**, 765w, 756vw-sh, 740mw, 664m $cm^{-1}$. $^1$H NMR ($d_6$-acetone, 298 K): 5.79 (s, hfac-H), 2.93 (s, $H_2O$) ppm. Additional small peaks appear to correspond to diethyl ether and hexanes. $^{19}$F NMR ($d_6$-acetone, 298 K, unreferenced): −76.17 (minor), −77.49, −77.98 (minor) ppm. $^{13}$C NMR ($d_6$-acetone, 298 K): 175.5 (q, $^2J_{C\text{-}F}$=32 Hz), 119.0 (q, $^1J_{C\text{-}F}$=286 Hz), 88.0 ppm. Additional peaks belong to diethyl ether, hexanes and TMS.

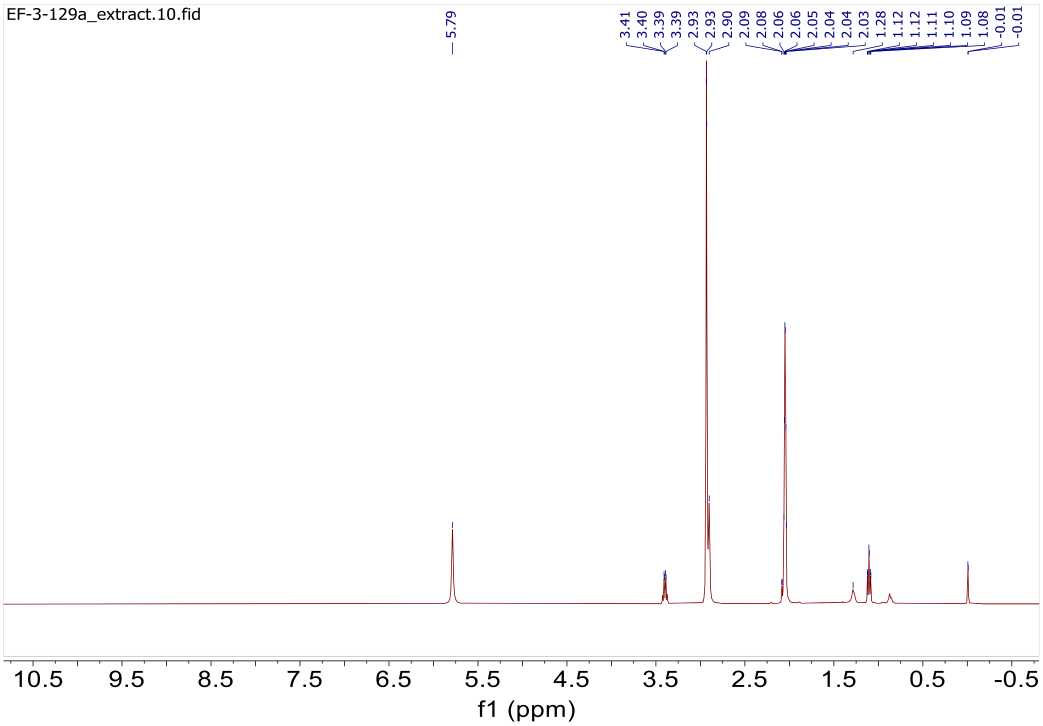


**Figure S7**: $^{1}H$ NMR Spectrum of $Na_2La(hfac)_5{\cdot}3H_2O$ with crude Na(hfac) repeat (298 K, 400 MHz, $d_6$-acetone).

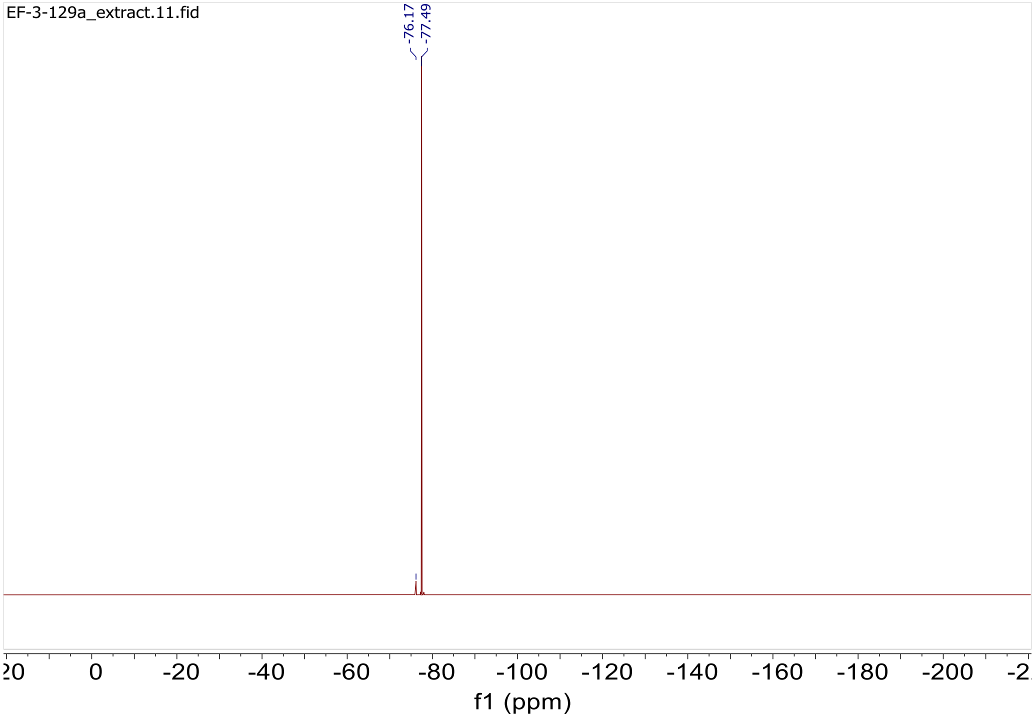


**Figure S8**: $^{19}F$ NMR Spectrum of $Na_2La(hfac)_5{\cdot}3H_2O$ with crude Na(hfac) repeat (298 K, 376 MHz, $d_6$-acetone).

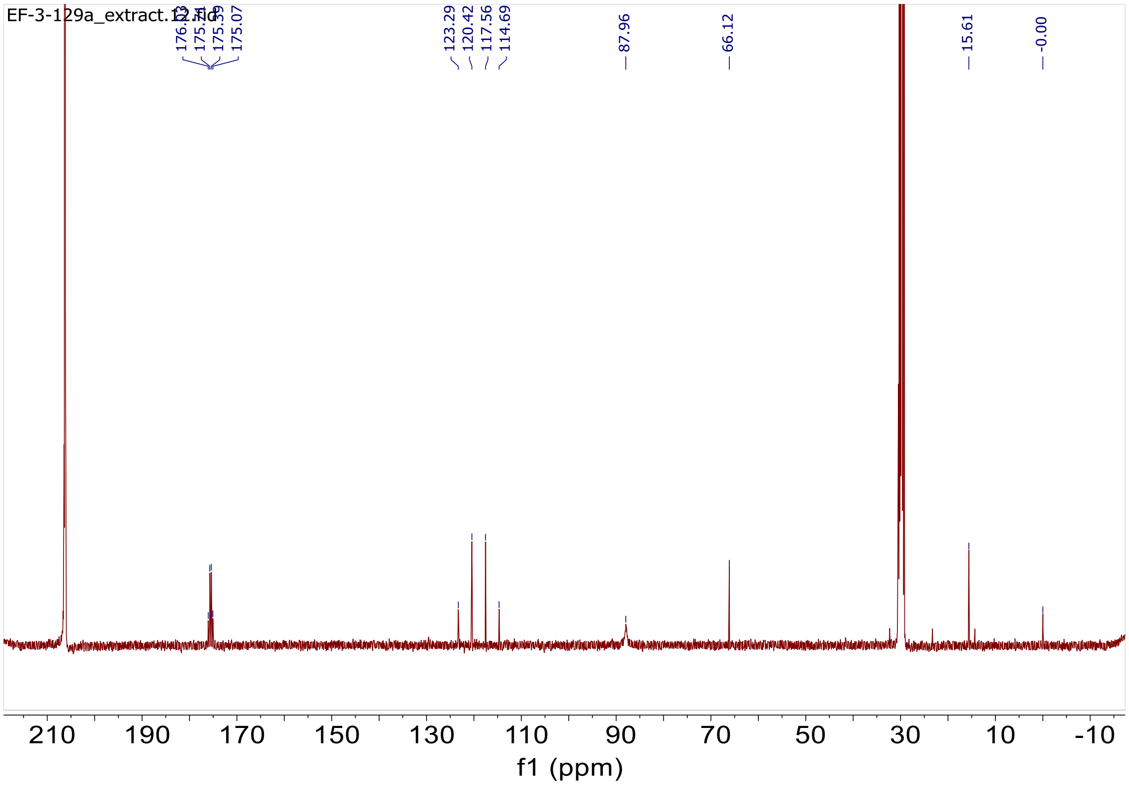

**Figure S9**: $^{13}C$ NMR Spectrum of $Na_2La(hfac)_5 \cdot 3H_2O$ (298 K, 100 MHz, $d_6$-acetone) with crude Na(hfac) repeat.

**Synthesis of $Na_2Ce(hfac)_5 \cdot 3H_2O$**

In a porcelain mortar, ground $CeCl_3 \cdot 7H_2O$ (0.3358 g, 0.8988 mmol) and Na(hfac) (0.6208 g, 2.699 mmol) were ground together for 20 min. until a finely ground solid was obtained. This material was extracted with 4 portions of 10 mL of $Et_2O$. Upon concentrating down, a yellow oil was obtained. To facilitate formation of a solid, 5 mL of hexanes was added and concentrated down to obtain a yellow solid. The final yield was 37% (0.2538 g, 0.1990 mmol). FT-IR (KBr, Nujol): 3716w-sp, 3639w-sp, 3458w-sh, 3449w-br, 3191vw-br, 3143vw-br, 1675mw-sh, 1655s, 1624vw, 1611vw-sh, 1563ms, 1542m, 1536m-sh, 1518w, 1493mw, 1461*vs, 1367mw, 1346w, 1301vw, 1257s, 1220s-sh, 1209s-br, 1148s, 1093mw, 968vw-br, 949w, 932vw, 916vw, 890vw, 844vw-br, 804mw, **795m**, 766w, 740mw, 664 mw $cm^{-1}$.

**Synthesis of $Na_2Pr(hfac)_5 \cdot 3H_2O$**

In a porcelain mortar, ground $PrCl_3 \cdot 7H_2O$ (0.3392 g, 0.9084 mmol) and Na(hfac) (0.6190 g, 2.691 mmol) were ground together for 15 min. until a finely ground solid was obtained. This material

was extracted with 4 portions of 10 mL of $Et_2O$. Upon concentrating down, a pale green oil was obtained. To facilitate formation of a solid, 8 mL of hexanes was added and concentrated down to obtain a light green solid. The final yield of this product was 46% (0.3127 g, 0.2450 mmol). FT-IR (KBr, Nujol): 3716w-sp, 3639w-sp, 3450w-br, 3183vw-br, 3139vw-br, 1720w, 1673mw-sh, 1655s, 1623w, 1613w-sh, 1564s, 1544m, 1517mw, 1494mw, 1461*vs, 1366mw, 1345w, 1323vw, 1258s, 1220ms-sh, 1209s, 1147vs, 1094m, 1045w, 971vw-br, 950w, 934vw, 917vw, 890vw, 844w, 803m-br, **795m**, 765w, 740mw, 664m $cm^{-1}$.

**Synthesis of $Na_2Nd(hfac)_5·3H_2O$**

In a porcelain mortar, ground $NdCl_3·7H_2O$ (0.3557 g, 0.9442 mmol) and Na(hfac) (0.6504 g, 2.827 mmol) were ground together for 15 min. until a finely ground solid was obtained. This material was extracted with 4 portions of 10 mL of $Et_2O$. Upon concentrating down, a lilac oil was obtained. To facilitate formation of a solid, a total of 10 mL of hexanes was added and concentrated down to obtain a lilac solid. The final yield was 41% (0.2957 g, 0.2311 mmol). FT-IR (KBr, Nujol): 3717w-sp, 3639w-sp, 3458w-br, 3301w-sh, 3139w, 1673m-sh, 1657ms, 1625w, 1614w, 1564s, 1544m, 1519mw, 1494mw, 1464*vs, 1366w, 1345w, 1321w, 1257s, 1219ms-sh, 1208s-br, 1146s-br, 1095mw, 1043w, 970vw-br, 950w, 932vw, 918vw, 892vw-br, 841vw, 804m-br, 801m, **795m**, 766w, 741mw, 664ms. This sample was recrystallized from diethyl ether and hexanes and produced single crystals of sufficient quality for X-ray diffraction. FT-IR (NaCl, Paratone/Nujol,): 3716mw-sp, 3639mw-sp, 3461w-br, 3305w-sh, 3214vw-br, 3139w, 1682mw-sh, 1656s, 1624mw, 1564s, 1545ms, 1529mw-sh, 1520mw-sh, 1493mw, 1465*mw, 1459mw-sh, 1366mw, 1345mw, 1321w, 1258s-br, 1219s-sh, 1208s-br, 1146s, 1137s-sh, 1094m, 950w, 932vw, 916vw, 890vw, 841vw, 802m-sh, **795m**, 765w, 741mw, 725vw, 664ms $cm^{-1}$.

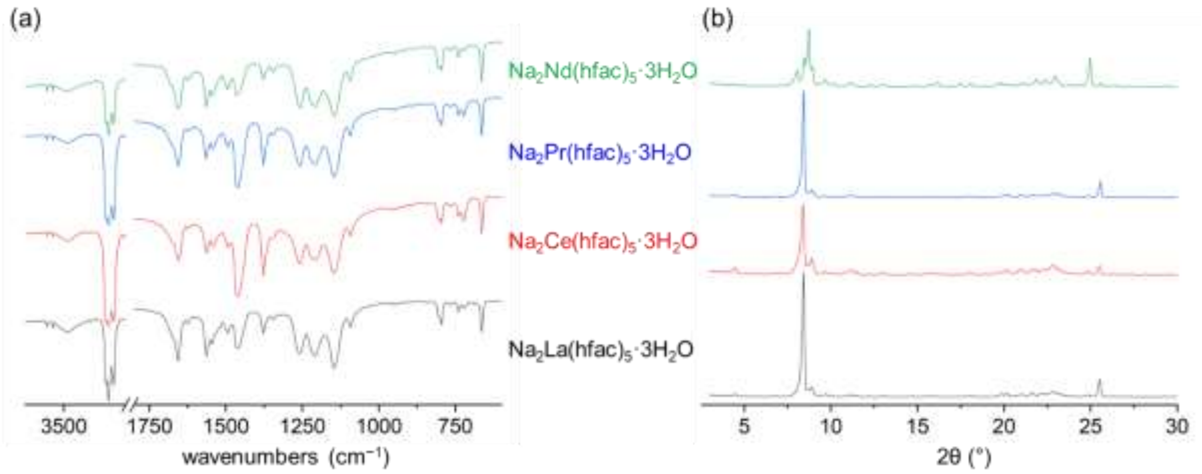


**Figure S10**: (a) Comparison of FT-IR spectra of $Na_2Ln(hfac)_5{\cdot}3H_2O$ complexes (KBr, Nujol, 2 cm$^{-1}$ res.) from repeat reactions and (b) comparison of XRD patterns of $Na_2Ln(hfac)_5{\cdot}3H_2O$ complexes (Cu Kα, 10°/min).

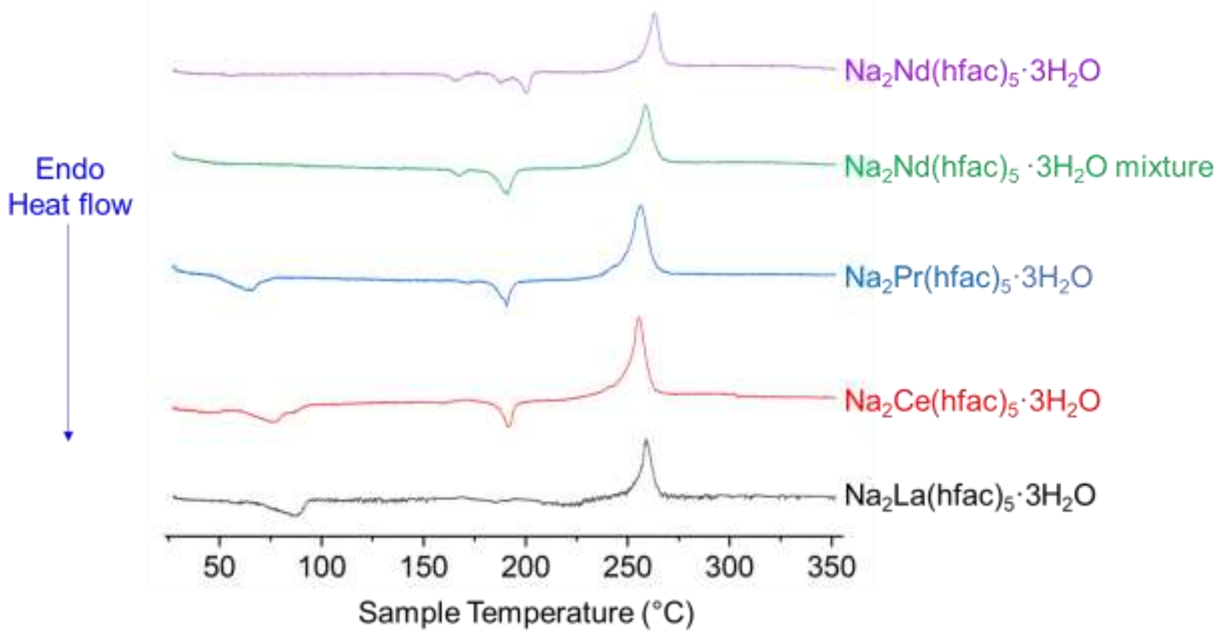


**Figure S11:** DSC plots of $Na_2Ln(hfac)_5{\cdot}3H_2O$ from bulk samples where crystals were obtained and an additional $Na_2Nd(hfac)_5{\cdot}3H_2O$ sample for comparison ($T_{initial}$ = 25 °C, $T_{ramp}$= 3 °C/min.).

**Synthesis of [NaSm(hfac)$_4$Na(TFA)]**

In a porcelain mortar, ground $SmCl_3{\cdot}7H_2O$ (0.3642 g, 0.9513 mmol) and Na(hfac) (0.6571 g, 2.8565 mmol) were ground together for 10 min. and then allowed to sit for 15 minutes until dry and a finely ground beige solid was obtained. This material was extracted with four portions of

$Et_2O$ (10 mL, 15 mL, 10 mL, 5 mL). Upon concentrating down, a pale yellow oil was obtained. To facilitate formation of a solid, a total of 15 mL of hexanes was added and concentrated down to obtain a pale yellow solid. The final yield was 57% (0.3731 g, 0.3280 mmol) assuming five eq. Na(hfac) used to generate 4 hfac and 1 tfa ligand. FT-IR (KBr, Nujol): 3713vw, 3589vw-br, 3526vw, 3434vw-br, 3147vw-br, 1650s, 1619w, 1562m, 1540m, 1497ms, 1465*vs, 1367w, 1349w, 1324vw, 1258s, 1216ms, 1207ms, 1153vs-br, 1143s-sh, 1098m, 951w, 892vw-br, 843vw, **810m**, **804m,** 769w, 744mw, 725mw, 664ms $cm^{-1}$.

This sample was recrystallized from diethyl ether and hexanes and produced single crystals of sufficient quality for X-ray diffraction. FT-IR (NaCl, paratone): 3712w, 3601w, 3583w, 3418vw-vbr, 3185vw-sh, 3145w, 1653s, 1617w, 1561m, 1536m, 1495mw-sh, 1464*vs, 1366m, 1349mw, 1299w-br, 1258s, 1210s-br, 1151s, 1096m, 1002vw-br, 973vw-br, 951w, 918vw, 890w, 870vw-br, 843vw-br, 809mw-sh, 803mw-br, 768w, 742mw, 663ms $cm^{-1}$.

**Control Experiment with 1 to 2.2 stoichiometry of $LaCl_3·7H_2O$ to Na(hfac)**

In a porcelain mortar, ground $LaCl_3·7H_2O$ (0.2071 g, 0.5577 mmol) and isolated, unpurified Na(hfac) (0.2881 g, 1.258 mmol) were ground together for 20 min. until a finely ground solid was obtained. This material was extracted with 2 portions of 10 mL of $Et_2O$. Upon concentrating down, a colorless oil was obtained. To facilitate formation of a solid, 3×5 mL of hexanes was added and the resulting solution was concentrated down to obtain a white solid. The final yield was 49% (0.1557 g, 0.1222 mmol). FT-IR (KBr, Nujol): 3717w-sp, 3639w-sp, 3458vw-br, 3175w-br, 3143w, 1671mw-sh, 1655m, 1625w, 1563m, 1544mw, 1527w, 1518w, 1506w, 1462*vs, 1367s, 1350m-br, 1301mw, 1266mw, 1208mw, 1147m, 1137m-sh, 1090mw, 1040w, 969w-br, 949w, 934vw, 918vw, 890w, 877vw, 846vw, 802mw, **795mw**, 765w, 740mw, 664 mw $cm^{-1}$. Elem. Anal. Calcd. for (assume $Na_2La(hfac)_5·3H_2O$) $Na_2LaC_{25}H_{11}F_{30}O_{13}$: %C 23.57, %H 0.87. Found %C 23.36, %H 0.92.

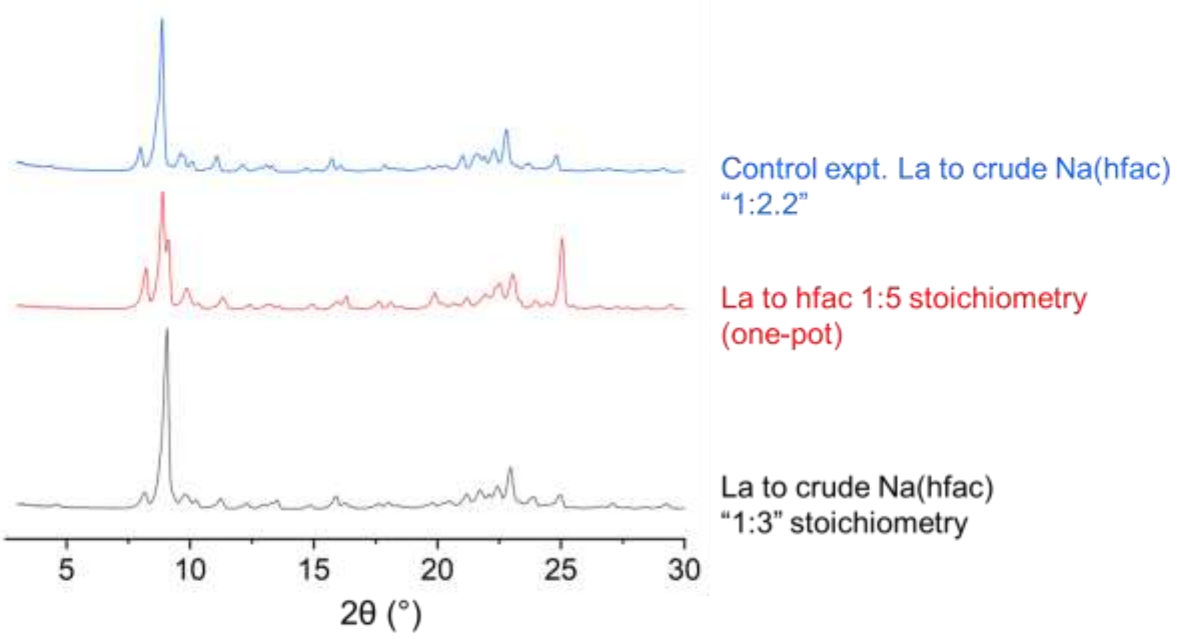


**Figure S12:** Comparison of XRD patterns of control experiment to stoichiometric and non-stoichiometric reactions to obtain $Na_2La(hfac)_5{\cdot}3H_2O$. Bottom "1:3" diffraction pattern from main text **Figure 2**.

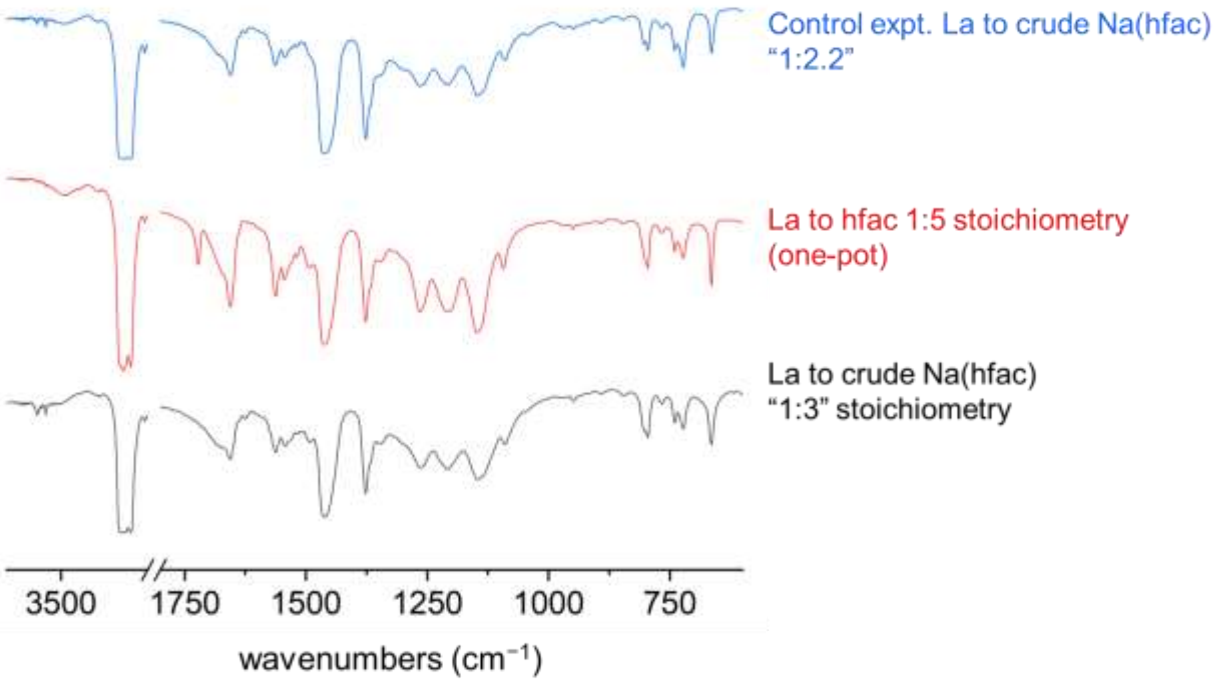


**Figure S13:** Comparison of FT-IR spectra of control experiment to stoichiometric and non-stoichiometric reactions to obtain $Na_2La(hfac)_5{\cdot}3H_2O$. (Nujol, 32 scans, 2 $cm^{-1}$ res.). Bottom "1:3" IR spectrum from main text **Figure 2**.

**Control Experiments with 4 eq. recrystallized Na(hfac)**

In a porcelain mortar, ground $SmCl_3 \cdot 7H_2O$ (0.2476 g, 0.6468 mmol) and recrystallized Na(hfac) (0.5937 g, 2.581 mmol) were ground together for 15 min. until a finely ground solid was obtained. An IR was taken of the crude material prior to extraction. FT-IR (KBr, Nujol): 3394vw-br, 3144vw, 1649s, 1619mw, 1561m, 1540m, 1497m, 1464*vs, 1367mw, 1347w, 1339w, 1258s, 1224s, 1204ms, 1146s-br, 1098m, 970vw-br, 950w, 916vw, 890vw, *872w*, 842w, 806m-br, 770w, 742mw, 736w, 663ms. This material was extracted with four portions of 10 mL of $Et_2O$. Upon concentrating down, a pale-yellow oil was obtained. To facilitate formation of a solid, 4×10 mL of hexanes was added and the resulting solution was concentrated down to obtain a pale yellow solid. The final yield was 0.1460 g. FT-IR (KBr, Nujol): 3670mw-sp, 3616w, 3394w-br, 3192vw-sh, 3150vw, 1650s, 1621m, 1564m, 1540m, 1496m, 1464*vs, 1367mw, 1349w, 1339w-sh, 1259s, 1222s, 1205s, 1151s-br, 1098m, 970vw-br, 950w, 916vw, 890vw, *872w*, 844w, **806m-br**, 771w, 742mw, *736mw*, 664ms $cm^{-1}$. Elem. Anal. Calcd. for NaSm(hfac)$_4$ $NaSmC_{24}H_4F_{24}O_8$: %C 23.98, %H 0.40. Elem. Anal. Calcd. for Sm(hfac)$_3$($H_2O$)$_2$: %C 22.31, %H 0.87. Found: $SmC_{15}H_7F_{18}O_8$: %C 22.39, %H 0.76. Duplicate: %C 22.31, %H 0.76.

**Control Experiment with 4 eq. crude Na(hfac)**

In a porcelain mortar, ground $SmCl_3 \cdot 7H_2O$ (0.1695 g, 0.4427 mmol) and isolated, unpurified Na(hfac) (0.4080 g, 1.774 mmol) were ground together for 15 min. until a finely ground solid was obtained. An IR was taken of the crude material prior to extraction. FT-IR (KBr, Nujol): 3428vw-br, 3142vw, 1699vw-sh, 1686mw-sh, 1674m-sh, 1659mw, 1651m, 1615w, 1560m, 1553m, 1540m, 1533m, 1498ms, 1465*vs, 1459s, 1366w, 1349w, 1260s, 1229m-sh, 1207s-br, 1156s-sh, 1143s-br, 1094w, 1081w, 969vw-br, 946w, 933vw, 915vvw, 889vvw, 862w-br, 852w-br, 843w-br, 806mw, 793w-sh, 774w, 757w, 740w-br, 726w, 665ms. The crude material was extracted with three portions of 10 mL of $Et_2O$. To facilitate formation of a solid, 15 mL of hexanes was added and the resulting solution was concentrated down to obtain a pale-yellow solid. FT-IR (KBr, Nujol): 3689w-sp, 351w-br, 3141vw, 1700w-sh, 1686mw-sh, 1672m-sh, 1661m, 1651mw, 1614w, 1561m, 1537m, 1512mw-sh, 1497ms, 1464*vs, 1367w, 1348w, 1337w, 1263s, 1225m-sh, 1205s-br, 1156s, 1144s-br, 1097w-br, 969vw-br, 946w, 915vvw, 890vvw, 843w, **805mw, 802mw-**sh, 770w, 761w, 741w-br, 726w, 666ms $cm^{-1}$. The final yield was 49% (0.1557 g, 0.1222 mmol).

Elem. Anal. Calcd. for NaSm(hfac)4 $NaSmC_{24}H_4F_{24}O_8$: %C 23.98, %H 0.40. Found %C 24.58, %H 0.64, duplicate: %C 24.74, %H 0.75.

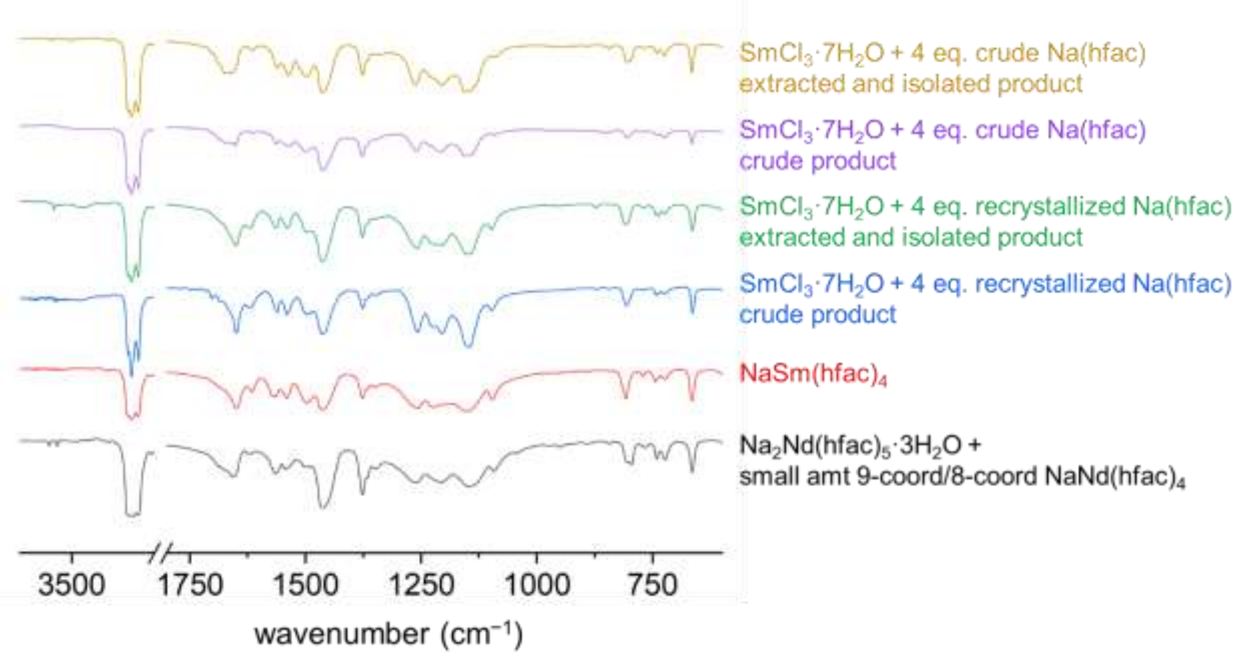


**Figure S14:** Comparison of IR Spectra of $Sm(hfac)_3(H_2O)_2$ syntheses performed with 4 eq. of Na(hfac). (KBr, Nujol, 2 $cm^{-1}$ res.) Bottom two spectra in Figure 2 in main text for comparison purposes.

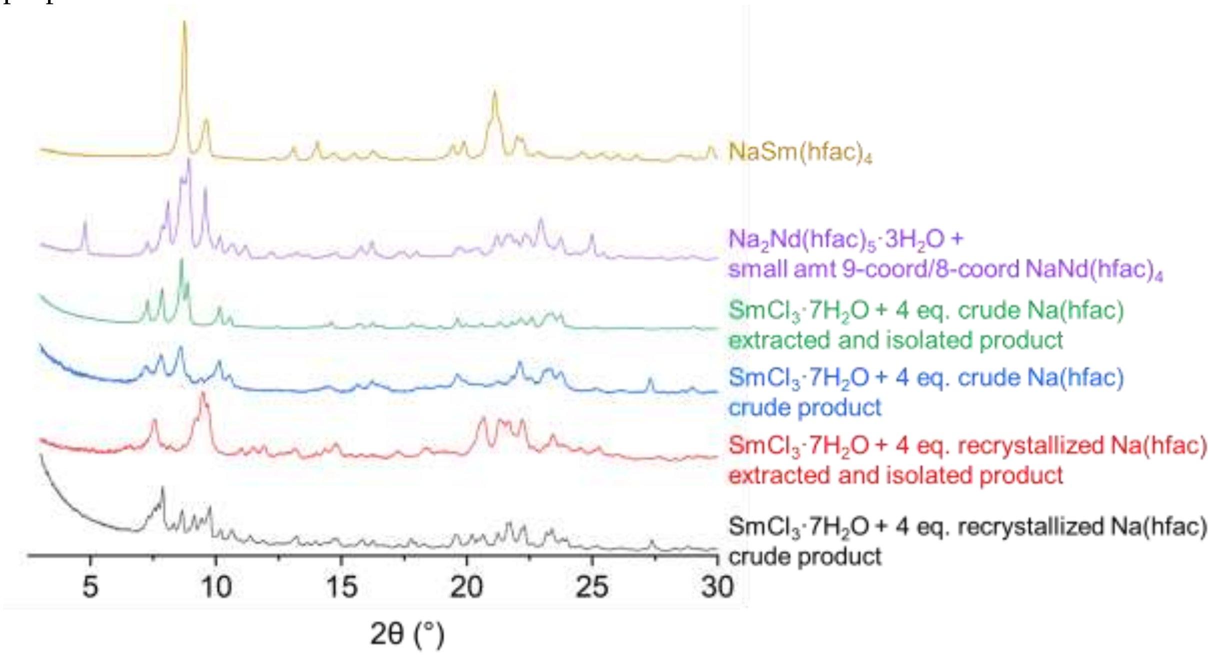


**Figure S15:** Comparison of XRD diffraction patterns of $Sm(hfac)_3(H_2O)_2$ syntheses performed with 4 eq. of Na(hfac). Top two diffraction patterns from Figure 2 in main text for comparison purposes.

**Mechanochemical Synthesis of Na[Tb(hfac)$_4$]**

In an agate mortar and pestle, Tb(hfac)$_3$(DME) (0.5484 g, 0.6302 mmol) was ground with Na(hfac) (0.1498 g, 0.6512 mmol) for 30 minutes until it turned to a fine flowing powder. The mixture was washed with hexanes to remove any excess Tb(hfac)$_3$(DME). The isolated insoluble material has only a weak OH peak and luminesces green under 365 nm light. The yield of the insoluble material was 0.3627 g (0.3591 mmol, 57%). This material was recrystallized in diethyl ether/hexanes to afford crystals suitable for single crystal data collection. FT-IR (KBr, Nujol): 3408vw-br, 3151vw, 1649s, 1612mw, 1564mw-br, 1537mw, 1500m, 1467mw, 1460*mw-sh, 1452mw, 1412vw, 1367w, 1348vw, 1327w-br, 1256s, 1223ms, 1204ms-sh, 1154ms-sh, 1145s, 1100mw, 1046w, 1019vvw, 952vw, 867vw, 846vvw, 806m, 770w, 743w, 737vw-sh, 664m cm$^{-1}$. Elem. Anal. Calcd. for $NaTbC_{20}H_4O_8F_{24}$: %C 23.78, %H 0.40. Found %C 23.52, %H 0.58. IR of crystal taken (NaCl, Nujol): 3157vw, 3142vw, 1684mw-sh, 1652s, 1615mw, 1564mw-br, 1541mw, 1506mw, 1495mw, 1487mw, 1465*s-sh, 1366w, 1350vw, 1258s, 1221ms, 1205ms, 1154s-sh, 1146s, 1097mw, 949vw, 918vw, 890vw, 843vw, 807m, 770w, 743w, 737vw-sh, 667m-sh, 664m cm$^{-1}$.
An alternate synthesis using crude Na(hfac) was conducted as described for La-Sm for comparison purposes. FT-IR (NaCl, Nujol): 3410w-br, 3151vw, 1687w-sh, 1650ms, 1612w, 1571mw, 1565mw-br, 1540mw, 1500mw, 1463*s-sh, 1367mw, 1352w, 1258s, 1223ms, 1204m, 1158s, 1149s-sh, 1100mw, 968vw, 951vw, 935vw, 918vw, 889vw, 845w, 807m, 771w, 743w, 735vw-sh, 665m cm$^{-1}$.

**One pot M1-SAG Reactions: Same stoichiometry yielding different products**

*One pot M1-SAG reaction that yields Ln(hfac)$_3$(H$_2$O)$_3$ 1:3 ratio (Ln:hfac)*

To a porcelain mortar, $Na_2CO_3 \cdot H_2O$ (1.3348 g, 10.765 mmol) was added and ground lightly until powdery. Hhfac (3 mL, 21 mmol) was added and ground until a powdery solid. $LaCl_3 \cdot 7H_2O$ (2.6609 g, 7.1643 mmol) was added and ground for ~10 min until the material was a fine powder. The solid was extracted with 3×20 mL of $Et_2O$. The solution was concentrated down to a colorless oil. To the colorless oil, 30 mL of hexanes was required to yield 2.7808 g of white solid was obtained (3.416 mmol, 48%). Yield assuming 70% Hhfac as limiting reagent (3.416 mmol, 70%). FT-IR (KBr, Nujol): 3524w-br, 3143vw, 1652ms, 1560mw-br, 1540mw, 1532w-sh, 1488mw-sh, 1460* vs, 1396vw, 1365w, 1349vw, 1340w-br, 1257ms, 1208ms, 1149s, 1094mw, 1062w, 1035w, 972vw-br, 949w, 918vw, 881w-br, *870w*, 846vw, 802m, 771w, 741mw, *735w*, 662m cm$^{-1}$. $^1$H NMR (400 MHz, d$_6$-acetone, 298 K): 5.96 (s, hfac-H), 3.77 (s, $H_2O$), 3.59 (q, EtOH), 1.12 (t,

EtOH) ppm. $^{19}$F NMR (376 MHz, $d_6$-acetone, 298 K, unreferenced): −76.8 (minor), −77.5 ppm. $^{13}$C NMR (100 MHz, $d_6$-acetone, 298 K): 176.0 (q, $^{2}J_{C\text{-}F}$=34 Hz), 118.6 (q, $^{1}J_{C\text{-}F}$=285 Hz), 89.7 ppm. Ethanol found at 57.8, 18.6 ppm. Elem. Anal. Calcd. for $La(hfac)_3(H_2O)_3$: %C 22.13, %H 1.11. Found %C 23.33, %H 1.48, duplicate %C 23.47, %H 1.38.

*One pot M1-SAG reaction that yields pentakis-hfac 1:3 ratio (Ln:hfac)*

To a porcelain mortar, $Na_2CO_3{\cdot}H_2O$ (0.4521 g, 3.675 mmol) was added and ground lightly until powdery. Hhfac (1 mL, 7 mmol) was added and ground until a powdery solid. $LaCl_3{\cdot}7H_2O$ (0.8868 g, 2.388 mmol) was added and ground for ~10 min until the material was a fine powder. The solid was extracted with 3×20 mL of $Et_2O$. The solution was concentrated down to a colorless oil. To the colorless oil, 20 mL of hexanes were added to facilitate the formation of a white solid. The yield was 1.0040 g of white solid (0.7879 mmol, 56%). Yield assuming 70% Hhfac as limiting reagent (0.7879 mmol, 80%). FT-IR (KBr, Nujol): 3717w-sp, 3639w-sp, 3481mw-br, 3139w, 1722mw, 1673mw-sh, 1655m, 1625w, 1562m-br, 1544mw, 1529mw-sh, 1518w, 1493mw, 1460* vs, 1367mw, 1345w-br, 1318vvw-sh, 1263ms, 1209ms, 1147s-br, 1091mw, 971vw-br, 949w, 918vw, 890vw, 843vw-br, 803mw-sh, **796m**, 766w, 740mw, 664m cm$^{-1}$. $^{1}$H NMR (400 MHz, $d_6$-acetone, 298 K): 5.80 (s, hfac-H), 3.12 (s, $H_2O$) ppm. $^{19}$F NMR (376 MHz, $d_6$-acetone, 298 K, unreferenced): −76.16 (minor), −77.47 ppm. Elem. Anal. Calcd. for $Na_2La(hfac)_5{\cdot}3H_2O$ $LaNa_2C_{25}H_{11}F_{30}O_{13}$: %C 23.57, %H 0.87. Found %C 23.74, %H 0.86, duplicate %C 23.78, %H 0.78.

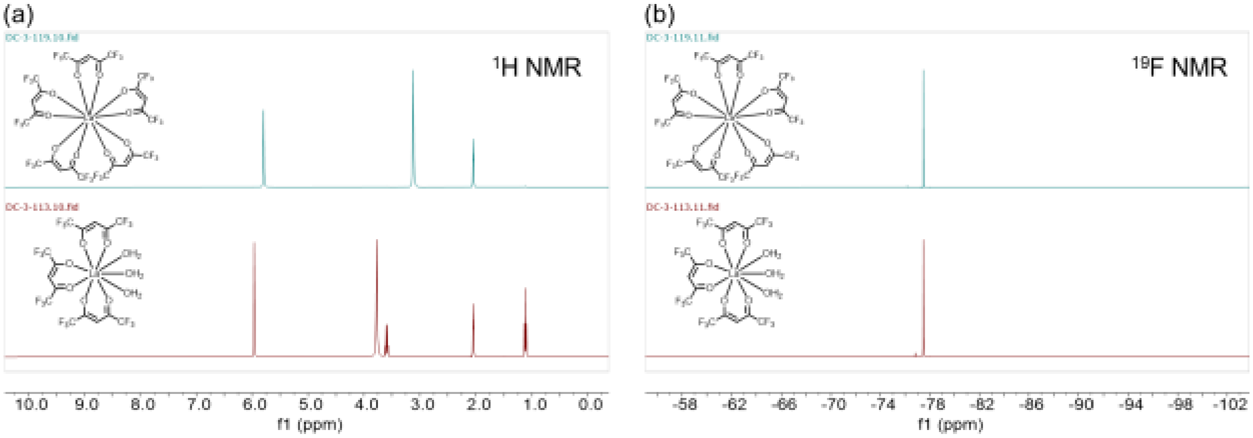


**Figure S16:** Comparison of (a) $^{1}$H NMR spectra (400 MHz, 298 K, $d_6$-acetone) and (b) $^{19}$F NMR spectra (376 MHz, 298 K, $d_6$-acetone) of $La(hfac)_3(H_2O)_3$ (bottom) and $Na_2La(hfac)_5{\cdot}3H_2O$.

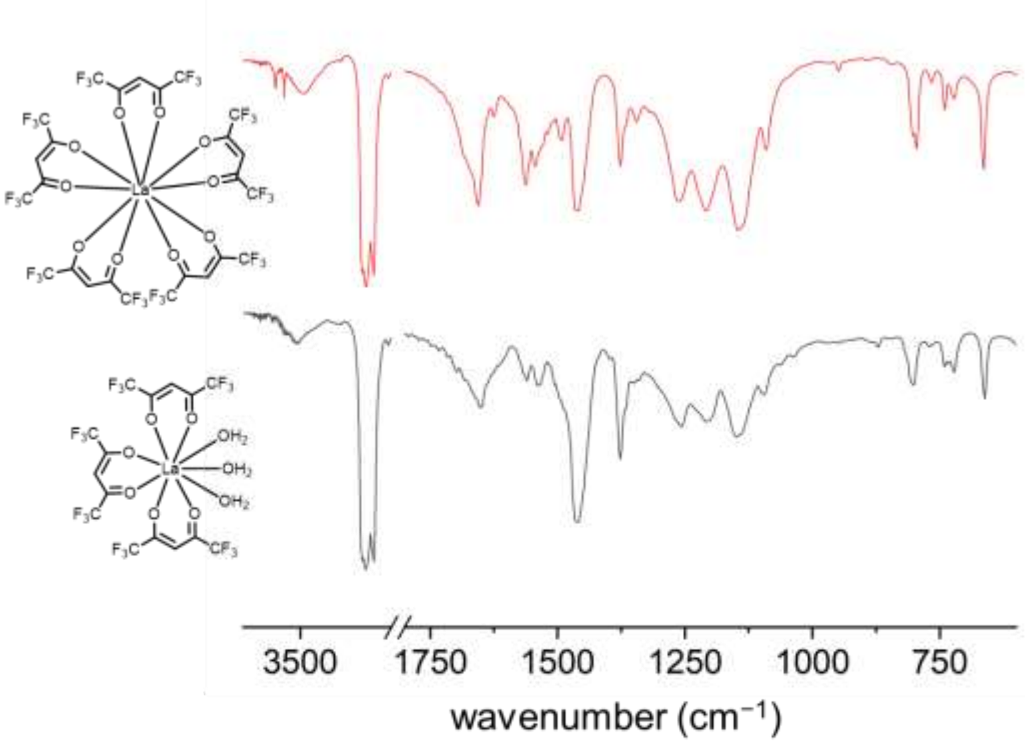


**Figure S17:** Comparison of FT-IR spectra of La(hfac)$_3$($H_2O$)$_3$ (bottom) and $Na_2$La(hfac)$_5$·3$H_2O$ (top) (Nujol, 2cm$^{-1}$ res.).

### S3a. Crystal structure of Pentakis-hfac cerium salt $Na_2$Ce(hfac)$_5$·3$H_2O$·$Et_2O$

Single crystals suitable for X-ray diffraction were grown by slow evaporation of diethyl ether and hexanes. A yellow crystal (block, approximate dimensions 0.38 × 0.18 × 0.14 mm$^3$) was placed onto the tip of a MiTeGen pin and mounted on a Bruker Venture D8 diffractometer equipped with a PhotonIII detector at 173.0 K.

### Data collection

The data collection was carried out using Mo Kα radiation (λ = 0.71073 Å, graphite monochromator) with a frame time of 1 second and a detector distance of 40 mm. A collection strategy was calculated and complete data to a resolution of 0.70 Å with a redundancy of 4.3 were collected. The frames were integrated with the Bruker SAINT[7] software package using a narrow-frame algorithm. The integration of the data using a triclinic unit cell yielded a total of 159133 reflections to a maximum θ angle of 30.30° (0.70 Å resolution), of which 14367 were independent

(average redundancy 11.076, completeness = 99.6%, Rint = 7.58%, Rsig = 4.06%) and 11963 (83.27%) were greater than $2\sigma(F^2)$. The final cell constants of a = 9.9933(7) Å, b = 12.5907(9) Å, c = 19.7678(14) Å, α = 92.353(2)°, β = 104.273(2)°, γ = 91.517(2)°, volume = 2406.7(3) $Å^3$, are based upon the refinement of the XYZ-centroids of reflections above 20 σ(I). Data were corrected for absorption effects using the Multi-Scan method (SADABS).[8] The calculated minimum and maximum transmission coefficients (based on crystal size) are 0.6720 and 0.8570.

**Structure solution and refinement**

The space group P-1 was determined based on intensity statistics and systematic absences. The structure was solved using the SHELX suite of programs[9,10] and refined using full-matrix least-squares on $F^2$ within the OLEX2 suite.[11] An intrinsic phasing solution was calculated, which provided most non-hydrogen atoms from the E-map. Full-matrix least squares / difference Fourier cycles were performed, which located the remaining non-hydrogen atoms. All non-hydrogen atoms were refined with anisotropic displacement parameters. The hydrogen atoms were placed in ideal positions and refined as riding atoms with relative isotropic displacement parameters. The final full matrix least squares refinement converged to R1 = 0.0542 and wR2 = 0.1443 ($F^2$, all data). The goodness-of-fit was 1.051. On the basis of the final model, the calculated density was 1.862 $g/cm^3$ and F(000), 1314 $e^-$.

**Table S1. Crystal data and structure refinement for $Na_2Ce(hfac)_5{\cdot}3H_2O{\cdot}Et_2O$ mo_21211_0m_a.**

| | | |
|---|---|---|
| Empirical formula | C29 H21 Ce F30 Na2 O14 | |
| Formula weight | 1349.56 | |
| Crystal color, shape, size | yellow block, 0.38 × 0.18 × 0.14 $mm^3$ | |
| Temperature | 173.0 K | |
| Wavelength | 0.71073 Å | |
| Crystal system, space group | Triclinic, P-1 | |
| Unit cell dimensions | a = 9.9933(7) Å | α = 92.353(2)°. |
| | b = 12.5907(9) Å | β = 104.273(2)°. |
| | c = 19.7678(14) Å | γ = 91.517(2)°. |
| Volume | 2406.7(3) $Å^3$ | |
| Z | 2 | |

## Table S1. Crystal data and structure refinement for $Na_2Ce(hfac)_5 \cdot 3H_2O \cdot Et_2O$ mo_21211_0m_a.

| | |
|---|---|
| Density (calculated) | 1.862 g/cm$^3$ |
| Absorption coefficient | 1.137 mm$^{-1}$ |
| F(000) | 1314 |
| **Data collection** | |
| Diffractometer | Bruker VENTURE D8 |
| Theta range for data collection | 1.982 to 30.297°. |
| Index ranges | -14<=h<=14, -17<=k<=17, -27<=l<=28 |
| Reflections collected | 159133 |
| Independent reflections | 14367 [$R_{int}$ = 0.0758] |
| Observed Reflections | 11963 |
| Completeness to theta = 25.242° | 100.0 % |
| **Solution and Refinement** | |
| Absorption correction | Semi-empirical from equivalents |
| Max. and min. transmission | 0.7460 and 0.6700 |
| Solution | Intrinsic methods |
| Refinement method | Full-matrix least-squares on F$^2$ |
| Weighting scheme | w = [$\sigma^2$Fo$^2$+ AP$^2$+ BP]$^{-1}$, with |
| | P = (Fo$^2$+ 2 Fc$^2$)/3, A = 0.0716, B = 6.9061 |
| Data / restraints / parameters | 14367 / 73 / 704 |
| Goodness-of-fit on F$^2$ | 1.051 |
| Final R indices [I>2$\sigma$(I)] | R1 = 0.0542, wR2 = 0.1340 |
| R indices (all data) | R1 = 0.0705, wR2 = 0.1443 |
| Extinction coefficient | n/a |
| Largest diff. peak and hole | 1.343 and -1.159 e.Å$^{-3}$ |

## Table S2. Hydrogen bonds for $Na_2Ce(hfac)_5 \cdot 3H_2O \cdot Et_2O$ mo_21211_0m_a [Å and °].

| D-H...A | d(D-H) | d(H...A) | d(D...A) | <(DHA) |
|---|---|---|---|---|
| C28-H28A...F16A#4 | 0.99 | 2.51 | 3.442(11) | 155.9 |
| C29-H29B...F30#3 | 0.98 | 2.67 | 3.431(12) | 134.5 |
| O11-H11A...O9 | 0.99 | 2.40 | 3.005(4) | 118.9 |
| O11-H11A...F25 | 0.99 | 2.58 | 3.562(14) | 172.1 |
| O11-H11A...F25A | 0.99 | 2.18 | 3.124(13) | 158.6 |
| O11-H11A...F33 | 0.99 | 2.43 | 3.242(18) | 138.9 |
| O11-H11B...O5 | 0.99 | 1.95 | 2.792(4) | 141.1 |
| O11-H11B...F14 | 0.99 | 2.54 | 3.371(6) | 141.6 |
| O13A-H13C...F13#3 | 0.92 | 2.12 | 2.93(4) | 146.8 |
| O13A-H13D...F26A#3 | 0.92 | 2.60 | 3.27(5) | 129.8 |

Symmetry transformations used to generate equivalent atoms:

#1 x-1,y,z #2 -x+1,-y,-z+1 #3 x+1,y,z #4 x+1,y+1,z

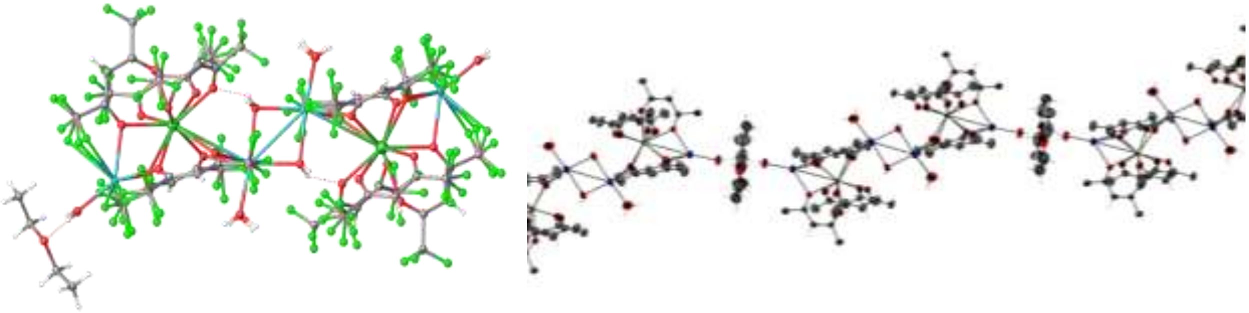

**Figure S18**: Molecular structure of $Na_2Ce(hfac)_5{\cdot}3H_2O{\cdot}Et_2O$ illustrating dimer formation facilitated by $Na^+$ coordinated to $H_2O$ and chain formation from $Et_2O$ hydrogen bonding to $H_2O$ coordinated to an $Na^+$.

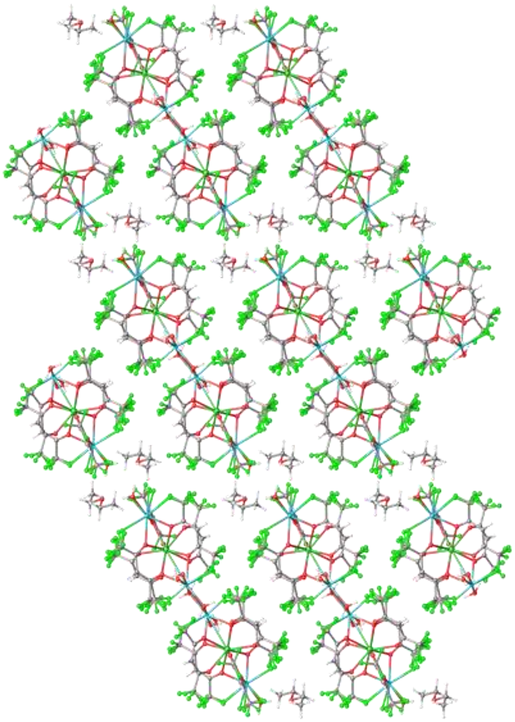

**Figure S19**: Packing of $Na_2Ce(hfac)_5{\cdot}3H_2O{\cdot}Et_2O$ along the *a* axis.

**S3b. Crystal structure of Pentakis-hfac praseodymium salt $Na_2Pr(hfac)_5{\cdot}3H_2O{\cdot}Et_2O$**

**Data collection**

A light green crystal (block, approximate dimensions 0.16 × 0.22 × 0.32 $mm^3$) was placed onto the tip of a MiTeGen pin and mounted on a Bruker Venture D8 diffractometer equipped with a PhotonIII detector at 110.00 K. The data collection was carried out using Cu Kα radiation (λ =

1.54178 Å, IμS micro-source) with a frame time of 5 seconds and a detector distance of 37 mm. The frames were integrated with the Bruker SAINT[7] software package using a narrow-frame algorithm. Data were corrected for absorption effects using the Multi-Scan method (SADABS).[8] Please refer to Table S3 for additional crystal and refinement information.

**Structure solution and refinement**

The space group P-1 was determined based on intensity statistics and systematic absences. The structure was solved using the SHELX suite of programs[9] and refined using full-matrix least-squares on $F^2$ within the OLEX2 suite.[11] An intrinsic phasing solution was calculated, which provided most non-hydrogen atoms from the E-map. Full-matrix least squares / difference Fourier cycles were performed, which located the remaining non-hydrogen atoms. All non-hydrogen atoms were refined with anisotropic displacement parameters. The hydrogen atoms were placed in ideal positions and refined as riding atoms with relative isotropic displacement parameters. The final full matrix least squares refinement converged to R1 = 0.0729 and wR2 = 0.1933 ($F^2$, all data). The goodness-of-fit was 1.045. On the basis of the final model, the calculated density was 1.890 g/cm$^3$ and F(000), 1312 $e^-$.

For this structure, the Pr analog was considered a dimer. The software considered the whole dimer formed by the bridging Na-O-Na as formula unit. That is consistent with Z=1 and Z' = 0.5. If the formula unit is halved (Pr=1, Na = 2 and so on), then Z= 2 and Z' = 1, for the triclinic structure.

**Table S3. Crystal data and structure refinement for $Na_2Pr(hfac)_5 \cdot 3H_2O \cdot Et_2O$.**

| | | |
|---|---|---|
| Empirical formula | C58 H38 F60 Na4 O28 Pr2 | |
| Formula weight | 2696.66 | |
| Crystal color, shape, size | light green, block, 0.16 × 0.22 × 0.32 mm$^3$ | |
| Temperature | 110.00 K | |
| Wavelength | 1.54178 Å | |
| Crystal system, space group | Triclinic, P-1 | |
| Unit cell dimensions | a = 9.9073(7) Å | α = 92.910(3)°. |
| | b = 12.5664(8) Å | β = 104.416(3)°. |
| | c = 19.6930(13) Å | γ = 91.879(3)°. |
| Volume | 2368.9(3) Å$^3$ | |
| Z | 1 | |
| Density (calculated) | 1.890 g/cm$^3$ | |
| Absorption coefficient | 9.734 mm$^{-1}$ | |
| F(000) | 1312 | |

**Table S3. Crystal data and structure refinement for $Na_2Pr(hfac)_5{\cdot}3H_2O{\cdot}Et_2O$.**

| | |
|---|---|
| **Data collection** | |
| Diffractometer | Bruker Venture D8 |
| Theta range for data collection | 4.102 to 68.428°. |
| Index ranges | -11<=h<=11, -15<=k<=15, -23<=l<=23 |
| Reflections collected | 37432 |
| Independent reflections | 8615 [$R_{int}$ = 0.0784] |
| Observed Reflections | 8064 |
| Completeness to theta = 67.679° | 99.5 % |
| | |
| **Solution and Refinement** | |
| Absorption correction | Semi-empirical from equivalents |
| Max. and min. transmission | 0.7531 and 0.3004 |
| Solution | Intrinsic methods |
| Refinement method | Full-matrix least-squares on $F^2$ |
| Weighting scheme | $w = [\sigma^2Fo^2 + AP^2 + BP]^{-1}$, with |
| | $P = (Fo^2 + 2\ Fc^2)/3$, A = 0.12, B = 19.8 |
| Data / restraints / parameters | 8615 / 26 / 677 |
| Goodness-of-fit on $F^2$ | 1.045 |
| Final R indices [I>2σ(I)] | R1 = 0.0729, wR2 = 0.1882 |
| R indices (all data) | R1 = 0.0768, wR2 = 0.1933 |
| Largest diff. peak and hole | 2.688 and -3.260 e.Å$^{-3}$ |

## Table S4. Hydrogen bonds for $Na_2Pr(hfac)_5{\cdot}3H_2O{\cdot}Et_2O$. [Å and °].

| D-H...A | d(D-H) | d(H...A) | d(D...A) | <(DHA) |
|---|---|---|---|---|
| C27-H27B...F38#4 | 0.99 | 2.50 | 3.421(17) | 155.2 |
| C26-H26C...F2#5 | 0.98 | 2.60 | 3.290(15) | 127.5 |
| O13-H13A...F10#1 | 0.89 | 2.19 | 2.920(18) | 139.0 |
| O13-H13B...F43#1 | 0.89 | 2.56 | 3.054(11) | 115.7 |

Symmetry transformations used to generate equivalent atoms:
#1 x-1,y,z #2 -x,-y+1,-z #3 x+1,y,z #4 -x+1,-y+1,-z+1
#5 -x+1,-y,-z+1

**S3c. Comparison of structural data for $Na_2Ln(hfac)_5{\cdot}3H_2O{\cdot}Et_2O$**

**Table S5:** Comparison of Unit Cells for Pentakis Complexes $Na_2Ln(hfac)_5{\cdot}3H_2O{\cdot}Et_2O$

| | **La** | **Ce** | **Pr** | **Nd** |
|---|---|---|---|---|
| **Crystal system** | triclinic | triclinic | triclinic | triclinic |
| **Space group** | *P*−1 | *P*−1 | *P*−1 | *P*−1 |
| **a** | 9.943 | 9.9933(7) | 9.9073(7) | 9.894 |
| **b** | 12.603 | 12.5907(9) | 12.5664(8) | 12.572 |
| **c** | 19.69 | 19.7678(14) | 19.6930(13) | 19.663 |
| **α** | 92.66 | 92.353(2) | 92.910(3) | 93.02 |
| **β** | 104.26 | 104.273(2) | 104.416(3) | 104.36 |
| **γ** | 92.06 | 91.517(2) | 91.879(3) | 92.06 |
| **V (Å³)** | 2385.92 | 2406.7(3) | 2368.9(3) | 2363.08 |
| ***T* (K)** | 110 | 173 | 110 | 110 |

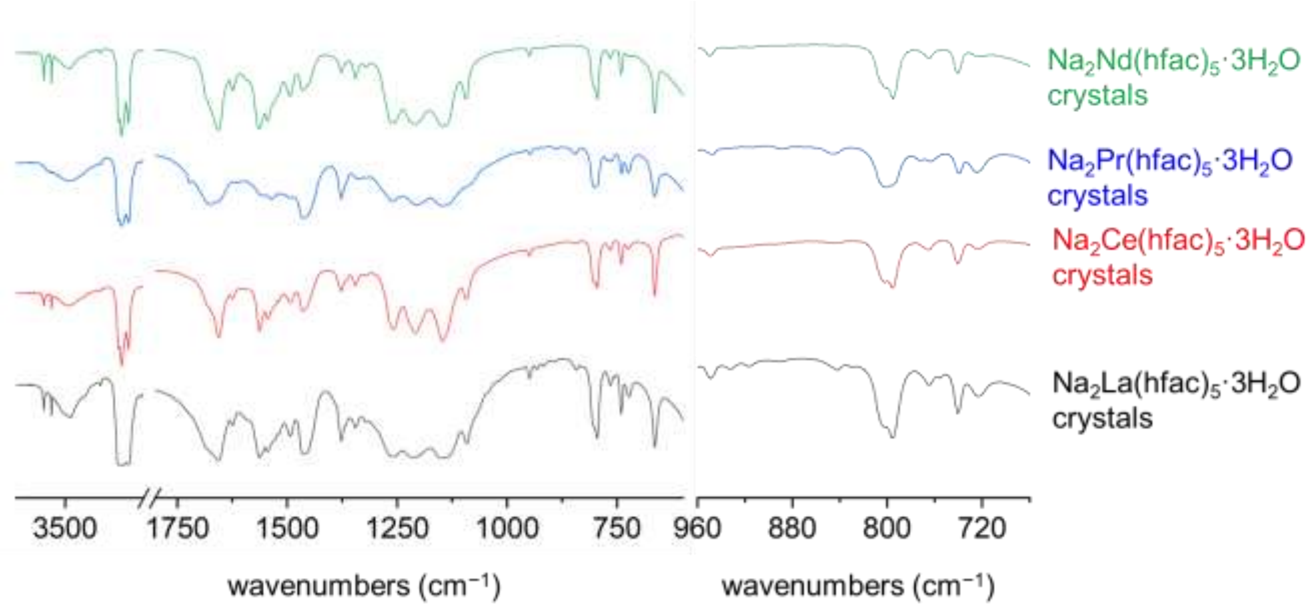


**Figure S20:** FT-IR of Crystals of $Na_2Ln(hfac)_5{\cdot}3H_2O{\cdot}Et_2O$ (NaCl, Nujol/paratone, 4 $cm^{-1}$ res.), $Na_2Ce(hfac)_5$ (KBr, Nujol/paratone, 2 $cm^{-1}$ res.) same batch from main text **Figure 3**.

**S4a: Crystal structure of $NaTb(hfac)_4$**

**Data collection**

A light yellow crystal (block, approximate dimensions 0.22 × 0.16 × 0.14 $mm^3$) was placed onto the tip of a MiTeGen pin and mounted on a Bruker Venture D8 diffractometer equipped with a

PhotonIII detector at 110.00 K. The data collection was carried out using Cu Kα radiation (λ = 1.54178 Å, IμS micro-source) with a frame time of 4 seconds and a detector distance of 37 mm. The frames were integrated with the Bruker SAINT[7] software package using a narrow-frame algorithm to a resolution of 0.82 Å. Data were corrected for absorption effects using the Multi-Scan method (SADABS).[8]

## Structure solution and refinement

The space group C 1 2/c 1 was determined based on intensity statistics and systematic absences. The structure was solved using the SHELX suite of programs[9] and refined using full-matrix least-squares on $F^2$ within the OLEX2 suite.[11] An intrinsic phasing solution was calculated, which provided most non-hydrogen atoms from the E-map. Full-matrix least squares / difference Fourier cycles were performed, which located the remaining non-hydrogen atoms. All non-hydrogen atoms were refined with anisotropic displacement parameters. The hydrogen atoms were placed in ideal positions and refined as riding atoms with relative isotropic displacement parameters. The final full matrix least squares refinement converged to $R_1$ = 0.0341 and $wR_2$ = 0.0848 ($F^2$, all data). The goodness-of-fit was 1.051. On the basis of the final model, the calculated density was 2.195 g/cm$^3$ and F(000), 1920 e$^-$.

**Table S6. Crystal data and structure refinement for NaTb(hfac)$_4$**

| | | |
|---|---|---|
| Empirical formula | C20 H4 F24 Na O8 Tb | |
| Formula weight | 1010.14 | |
| Crystal color, shape, size | light yellow block, 0.22 × 0.16 × 0.14 mm$^3$ | |
| Temperature | 110.00 K | |
| Wavelength | 1.54178 Å | |
| Crystal system, space group | Monoclinic, C 1 2/c 1 | |
| Unit cell dimensions | a = 21.6984(14) Å | α = 90°. |
| | b = 12.1402(8) Å | β = 125.345(2)°. |
| | c = 14.2264(18) Å | γ = 90°. |
| Volume | 3056.8(5) Å$^3$ | |
| Z | 4 | |
| Density (calculated) | 2.195 g/cm$^3$ | |
| Absorption coefficient | 13.350 mm$^{-1}$ | |
| F(000) | 1920 | |
| **Data collection** | | |
| Diffractometer | Bruker Venture D8 | |
| Theta range for data collection | 4.797 to 70.215°. | |

**Table S6. Crystal data and structure refinement for $NaTb(hfac)_4$**

| | |
|---|---|
| Index ranges | -26<=h<=24, -14<=k<=14, -17<=l<=17 |
| Reflections collected | 25193 |
| Independent reflections | 2912 [$R_{int}$ = 0.0554] |
| Observed Reflections | 2835 |
| Completeness to theta = 67.679° | 99.9 % |
| **Solution and Refinement** | |
| Absorption correction | Semi-empirical from equivalents |
| Max. and min. transmission | 0.7533 and 0.4579 |
| Solution | Intrinsic methods |
| Refinement method | Full-matrix least-squares on $F^2$ |
| Weighting scheme | $w = [\sigma^2 Fo^2 + AP^2 + BP]^{-1}$, with |
| | $P = (Fo^2 + 2 Fc^2)/3$, A = 0.039, B = 25.8 |
| Data / restraints / parameters | 2912 / 14 / 233 |
| Goodness-of-fit on $F^2$ | 1.051 |
| Final R indices [$I > 2\sigma(I)$] | R1 = 0.0341, wR2 = 0.0839 |
| R indices (all data) | R1 = 0.0350, wR2 = 0.0848 |
| Largest diff. peak and hole | 0.742 and -1.017 e.Å$^{-3}$ |

## S4b. Crystal structure of $NaSm(hfac)_4Na(TFA)$

### Data collection

A light yellow crystal (block, approximate dimensions 0.04 × 0.06 × 0.16 mm$^3$) was placed onto the tip of a MiTeGen pin and mounted on a Bruker Venture D8 diffractometer equipped with a PhotonIII detector at 110.00 K. The data collection was carried out using Cu K$\alpha$ radiation ($\lambda$ = 1.54178 Å, with a frame time of 32 seconds and a detector distance of 37 mm. The frames were integrated with the Bruker SAINT[7] software package using a narrow-frame algorithm. Data were corrected for absorption effects using the Multi-Scan method (SADABS).[8]

### Structure solution and refinement

The space group P 1 21/n 1 was determined based on intensity statistics and systematic absences. The structure was solved using the SHELX suite of programs[9] and refined using full-matrix least-squares on $F^2$ within the OLEX2 suite.[11] An intrinsic phasing solution was calculated, which provided most non-hydrogen atoms from the E-map. Full-matrix least squares / difference Fourier cycles were performed, which located the remaining non-hydrogen atoms. All non-hydrogen atoms were refined with anisotropic displacement parameters. The hydrogen atoms were placed in ideal positions and refined as riding atoms with relative isotropic displacement parameters. The

final full matrix least squares refinement converged to R1 = 0.0431 and wR2 = 0.1114 ($F^2$, all data). The goodness-of-fit was 1.082. On the basis of the final model, the calculated density was 2.215 g/cm$^3$ and F(000), 2172 e$^-$.

**Table S7. Crystal data and structure refinement for NaSm(hfac)$_4$Na(TFA)**

| | | |
|---|---|---|
| Empirical formula | C22 H4 F27 Na2 O10 Sm | |
| Formula weight | 1137.58 | |
| Crystal color, shape, size | light yellow, block, 0.04 × 0.06 × 0.16 mm$^3$ | |
| Temperature | 110.00 K | |
| Wavelength | 1.54178 Å | |
| Crystal system, space group | Monoclinic, P 1 21/n 1 | |
| Unit cell dimensions | a = 13.9951(7) Å | $\alpha$ = 90°. |
| | b = 12.3332(6) Å | $\beta$ = 95.308(2)°. |
| | c = 19.8514(10) Å | $\gamma$ = 90°. |
| Volume | 3411.7(3) Å$^3$ | |
| Z | 4 | |
| Density (calculated) | 2.215 g/cm$^3$ | |
| Absorption coefficient | 15.094 mm$^{-1}$ | |
| F(000) | 2172 | |
| **Data collection** | | |
| Diffractometer | Bruker Venture D8 | |
| Theta range for data collection | 3.708 to 68.489°. | |
| Index ranges | -16<=h<=16, -14<=k<=14, -23<=l<=23 | |
| Reflections collected | 36802 | |
| Independent reflections | 6240 [$R_{int}$ = 0.0913] | |
| Observed Reflections | 5602 | |
| Completeness to theta = 67.679° | 99.9 % | |
| **Solution and Refinement** | | |
| Absorption correction | Semi-empirical from equivalents | |
| Max. and min. transmission | 0.7531 and 0.3572 | |
| Solution | Intrinsic methods | |
| Refinement method | Full-matrix least-squares on $F^2$ | |
| Weighting scheme | w = [$\sigma^2$Fo$^2$+ AP$^2$+ BP]$^{-1}$, with | |
| | P = (Fo$^2$+ 2 Fc$^2$)/3, A = 0.062, B = 4.94 | |
| Data / restraints / parameters | 6240 / 4 / 557 | |
| Goodness-of-fit on $F^2$ | 1.082 | |
| Final R indices [I>2$\sigma$(I)] | R1 = 0.0431, wR2 = 0.1061 | |
| R indices (all data) | R1 = 0.0494, wR2 = 0.1114 | |
| Largest diff. peak and hole | 2.129 and -1.368 e.Å$^{-3}$ | |

**S4c**. **Preliminary structure connectivity plot**

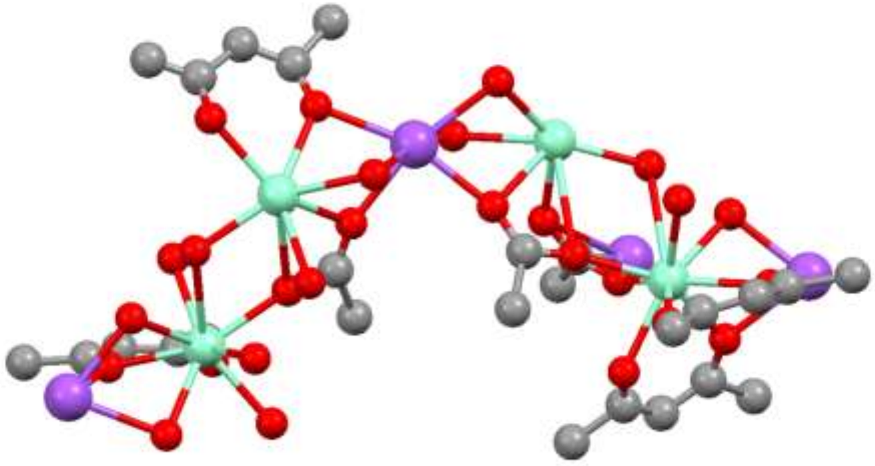

**Figure S21:** Connectivity plot of NaSm(hfac)$_x$NaTFA$_y$ from unrefined crystallographic data. Fluorine atoms were removed for clarity. Samarium (aqua), sodium (purple), oxygen (red), carbon (grey).

**S5a. One-pot Hydrate Syntheses and Characterization (La to Sm)**

**La(hfac)$_3$(H$_2$O)$_3$ solution synthesis:**

In a 250 mL Erlenmeyer flask, $Na_2CO_3 \cdot H_2O$ (0.7421 g, 5.985 mmol) was dissolved in 75 mL of water. To this solution, Hhfac (1.9 mL, 14 mmol) was added and allowed to stir for five minutes following the cessation of bubbles. This solution had a pH of 5. To this solution $LaCl_3 \cdot 7H_2O$ (1.7380 g, 4.679 mmol) was added. A white suspension formed immediately, 25 mL of water was added and the solution became transparent. This solution was allowed to stir for 2 hours. Following stirring, the solution was extracted with 3×100 mL portions of $Et_2O$. The $Et_2O$ was then dried over $MgSO_4$ and washed with an additional 20 mL aliquot of $Et_2O$. The solution was then concentrated down to yield a colorless oil. Hexanes (~20 mL) were added and the solution, then slowly evaporated to yield a white solid. The yield was 2.8916 g (3.5519 mmol, 76 %). FT-IR (Nujol, cm$^{-1}$): 3690mw, 3574m-sh, 3508m, 3405m, 3313m-sh, 3149w, 1652ms, 1616m, 1564m, 1559m-sh, 1541m-sh, 1538ms, 1487s-br, 1464s-br*, 1456s, 1365mw, 1349mw, 1265ms-sh, 1253s, 1208s, 1198s, 1144vs, 1098ms, 988w, 970w, 949vw, 909w, 899vw-sh, *870vw*, *807ms-br*, 801m-sh, 774w, 763vw, 742mw, *734w-sh*, *670m-sh*, 662ms cm$^{-1}$. Elem. Anal. Calcd. for $LaC_{15}H_9F_{18}O_9$:

%C 22.13, %H 1.11, Found %C 22.97, %H 1.23. $^{1}$H NMR (400 MHz, $d_6$-acetone): 5.98 (s), 3.60 (br, s) ppm. $^{19}$F NMR (376 MHz, $d_6$-acetone): −76.6 (minor), −77.5, −77.6 ppm.

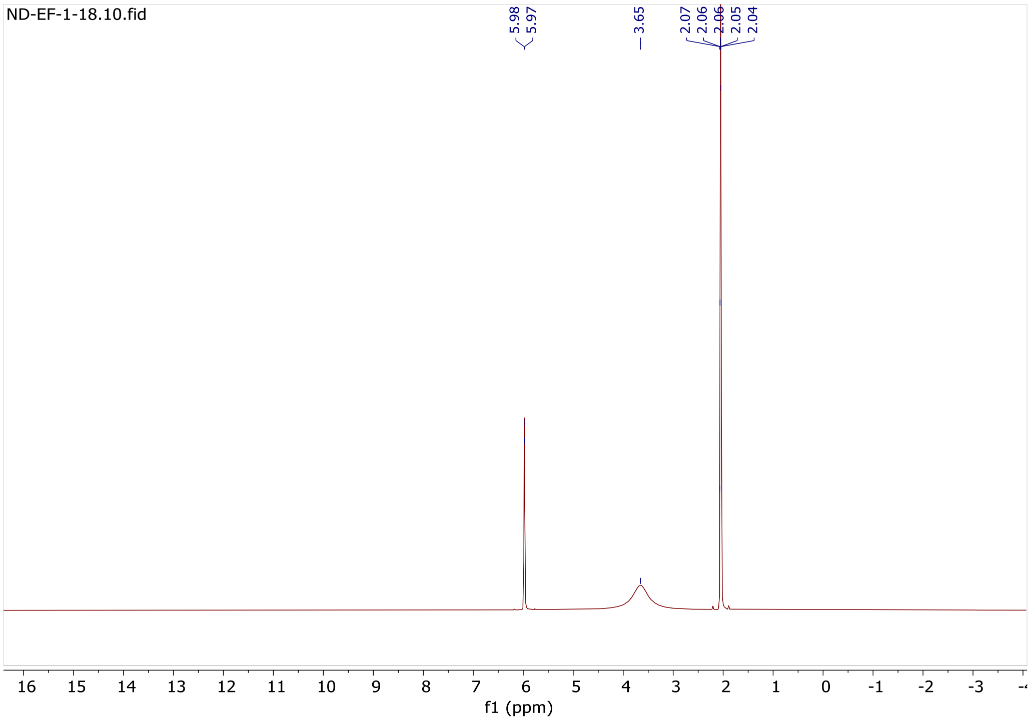


**Figure S22:** $^{1}$H NMR of solution $La(hfac)_3(H_2O)_3$ (298 K, $d_6$-acetone, 400 MHz).

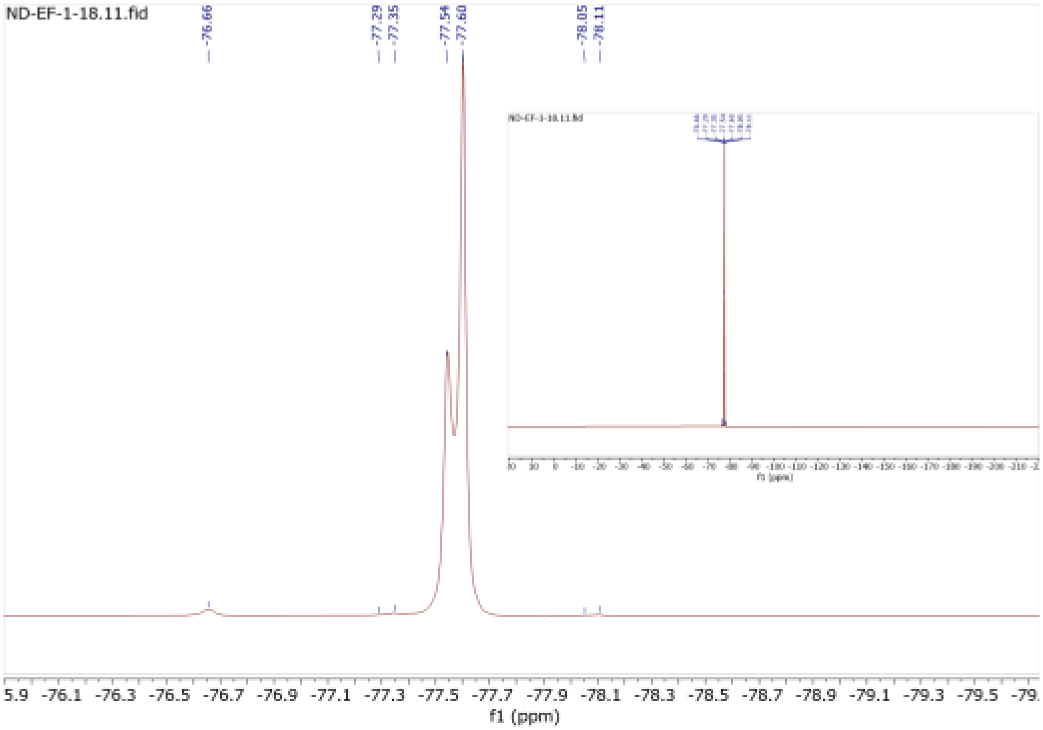


**Figure S23:** $^{19}$F NMR of solution $La(hfac)_3(H_2O)_3$ (298 K, $d_6$-acetone, 376 MHz).

**$La(hfac)_3(H_2O)_3$ solution reaction 2:**

In a 250 mL Erlenmeyer flask, $Na_2CO_3{\cdot}H_2O$ (0.7415 g, 5.985 mmol) was dissolved in 75 mL of water. To this solution, Hhfac (1.9 mL, 14 mmol) was added and allowed to stir for five minutes following the cessation of bubbles. This solution had a pH of 5. To this solution $LaCl_3{\cdot}7H_2O$ (1.7290 g, 4.655 mmol) was added. A white suspension formed immediately, 25 mL of water was added and the solution became transparent after 1 minute. This solution was allowed to stir for 3 hours. Following stirring, the solution was extracted with 3×100 mL portions of $Et_2O$. The pH of the aqueous layer was between 5 to 6. The $Et_2O$ was then dried over $MgSO_4$. The solution was then concentrated down to yield a white hygroscopic solid. Hexanes (10 mL) was added and rotovapped to yield a powdery, hygroscopic white solid. This reaction yielded 2.8277 g of white solid (3.4734 mmol, 75%). FT-IR (Nujol, $cm^{-1}$): 3685vw, 3505mw-br, 3407mw-br, 3286w-sh, 1654m, 1614w, 1566mw, 1541 mw, 1490m-sh, 1464ms*, 1367mw, 1352w-sh, 1340vw-sh, 1266m-sh, 1254ms, 1207ms, 1199ms-sh, 1145s, 1099m, 987w, 971vw-br, 949vw, 908w, 807mw-br, 774vw, 763vw, 741w, 669 mw-sh, 662m $cm^{-1}$. Elem. Anal. Calcd. for $LaC_{15}H_9F_{18}O_9$: %C 22.13, %H 1.11, Found %C 23.36, %H 1.36, duplicate %C 23.51, %H 1.26. $^1H$ NMR ($d_6$-acetone, 298 K): 7.03 (s, 2H), 6.63 (s, 0.20), 5.96 (s, 3H, presumed hfac-H), 4.15 (s, br, $H_2O$), 3.36 (s, 0.28H), 2.25 (s, 1H) ppm. $^{19}F$ NMR ($d_6$-acetone, 298 K, unreferenced): −77.51, −79.76 (minor), −86.65 (minor), −87.84 ppm.

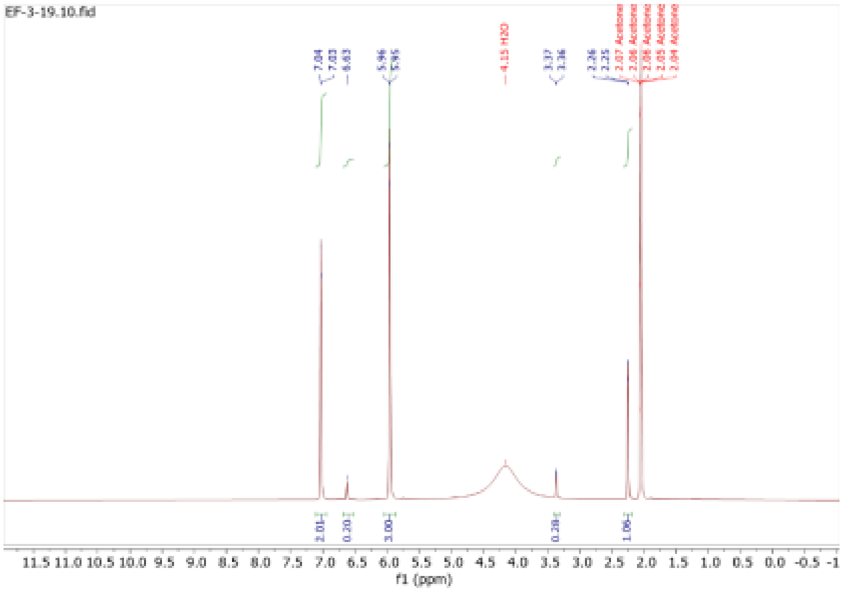


**Figure S24**: $^1H$ NMR of $La(hfac)_3(H_2O)_3$ solution reaction 2 synthesis (298 K, 400 MHz, $d_6$-acetone).

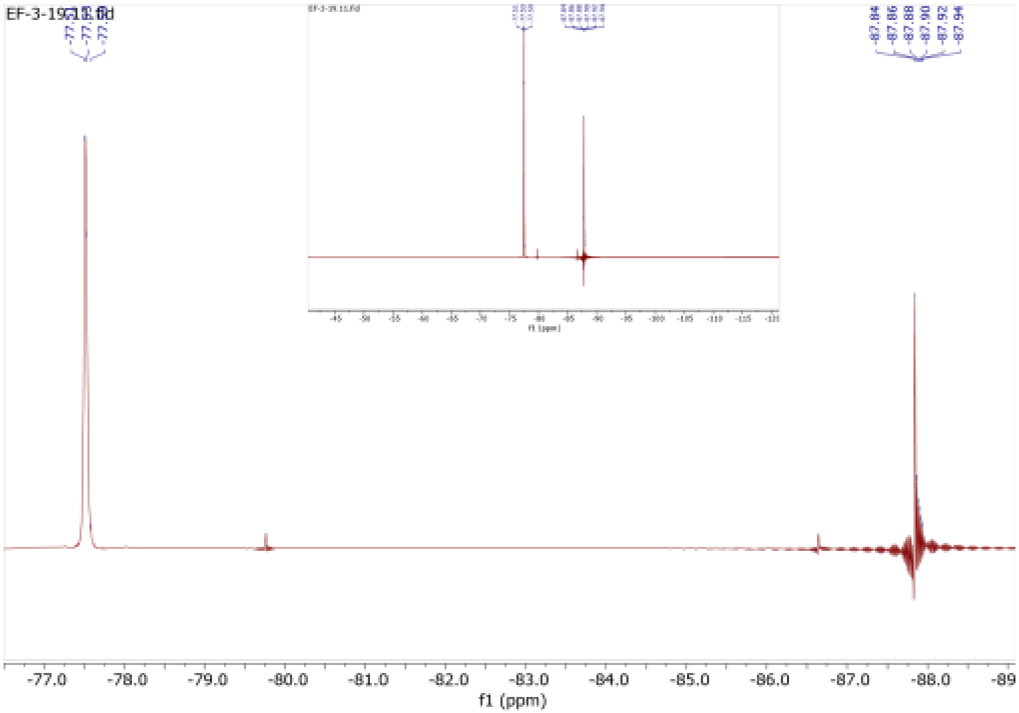


**Figure S25**: $^{19}$F NMR of La(hfac)$_3$($H_2O$)$_3$ solution reaction 2 synthesis (298 K, 376 MHz, d$_6$-acetone).

**La(hfac)$_3$($H_2O$)$_3$ mechanochemical synthesis (I) M1-SAG:**

To a porcelain mortar, $Na_2CO_3 \cdot H_2O$ (0.4888 g, 3.942 mmol) was added and ground lightly until powdery. Hhfac (1.2 mL, 8.6 mmol) was added and ground until a fine white solid. $LaCl_3 \cdot 7H_2O$ (1.1789 g, 3.1741 mmol) was added and ground for ~10 min until it formed a white solid (not pasty). The white solid was extracted with 150 mL of $Et_2O$ and concentrated to an oil. The soluble extracts were further diluted with 100 mL of hexane. After two days at room temperature, white waxy crystals were obtained, 0.4996 g (0.6137 mmol, 19%). FT-IR (Nujol, cm$^{-1}$): 3689mw, 3579mw-sh, 3523m-br, 3329w-sh, 3216w-br, 3143w, *1706mw*, 1650vs-br, 1613ms, 1560ms, 1535ms, 1480s-br, 1464s-sh*, 1365mw, 1347mw, 1323mw, 1255vs-br, 1220s-br, 1204s-br, 1147vs-br, 1101ms, 1095ms-sh, 949w, 934vw, 918vw-br, 890vw-br, *872vw*, 847vvw-br, 806ms, 800ms, 771w-br, 764w, 742m, *663s-br* cm$^{-1}$. Elem. Anal. Calcd. for $LaC_{15}H_9F_{18}O_9$: %C 22.13, %H 1.11, Found %C 24.52, %H 1.66. Re-sent EA: %C 23.70, %H 1.66, %C 23.85, %H 1.77. $^1$H NMR (400 MHz, d$_6$-acetone): 5.98 (s), 3.60 (s) ppm. Minor resonances are observed at 1.28 (s), 0.87 (s, br) ppm. $^{19}$F NMR (376 MHz, d$_6$-acetone): −76.9 (minor), −77.5, −77.6 (small shoulder) ppm.

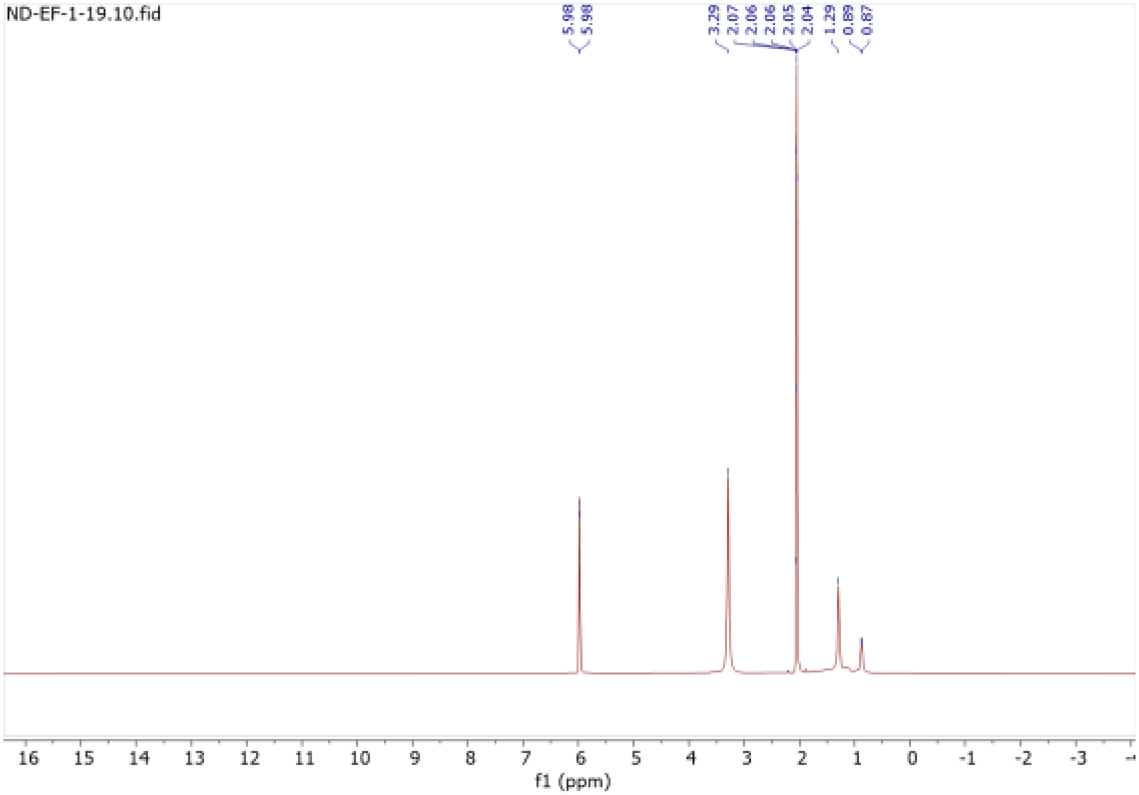


**Figure S26:** $^{1}H$ NMR of M1-SAG $La(hfac)_3(H_2O)_3$ (298 K, $d_6$-acetone, 400 MHz).

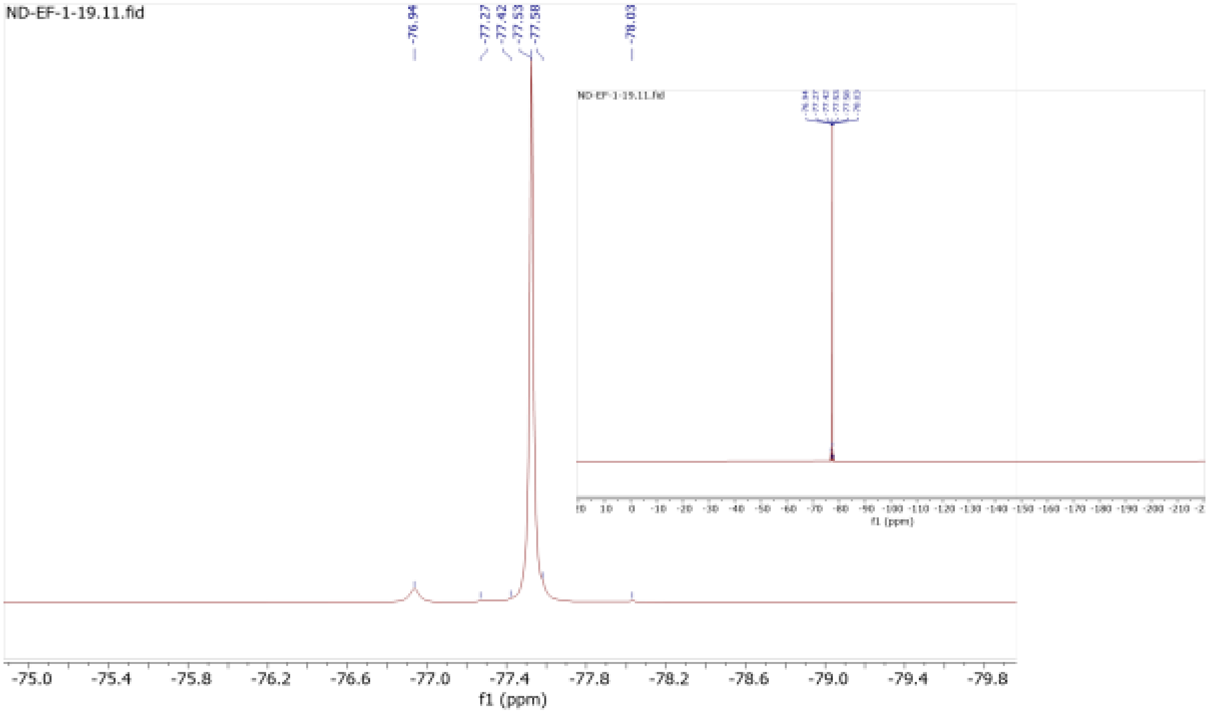


**Figure S27:** $^{19}F$ NMR of M1-SAG $La(hfac)_3(H_2O)_3$ (298 K, $d_6$-acetone, 376 MHz).

**La(hfac)$_3$(H$_2$O)$_3$ M1-SAG reaction 2:**

To a porcelain mortar, $Na_2CO_3{\cdot}H_2O$ (0.5572 g, 4.494 mmol) was added and ground lightly until powdery. Hhfac (1.3 mL, 9.3 mmol) was added and ground until a fine white solid. $LaCl_3{\cdot}7H_2O$ (1.1155 g, 3.0034 mmol) was added and ground for ~10 min until it formed a white solid (not pasty). The white solid was extracted with 70 mL of $Et_2O$ and concentrated to an oil. Approximately 10 mL of hexanes were added and the solution was concentrated down to a white solid. The yield of the white solid was 1.2211 g (1.4999 mmol, 50%). FT-IR (Nujol, cm$^{-1}$): 3687w-sp, 3673w-sh, 3668mw-sh, 3654mw, 3583w-sp, 3527mw-br, 3147w, 1652s-br, 1560m, 1533m, 1481s-sh, 1465s-br*, 1457s-br*, 1366mw, 1349w, 1322w, 1256s-br, 1219s-sh, 1204s-br, 1146vs-br, 1094m, 1043vvw, 1023vvw, 969vvw-br, 949vw, 934vvw, 918vvw, 891vvw-br, *872vvw*, 846vvw-br, 807mw-sh, 800ms, 768vw-br, 759vw, 741mw, 662m cm$^{-1}$. $^{1}$H NMR (d$_6$-acetone, 298 K): 5.96 (s, 3H, presumed hfac-H), 3.88 (s, $H_2O$) ppm. $^{19}$F NMR (d$_6$-acetone, 298 K, unreferenced): −76.84 (minor), −77.49 ppm. $^{13}$C NMR (d$_6$-acetone, 298 K): 176.2 (q, $^{2}J_{C\text{-}F}$=33 Hz), 118.8 (q, $^{1}J_{C\text{-}F}$=285 Hz), 89.8 ppm. Elem. Anal. Calcd. for $LaC_{15}H_9F_{18}O_9$: %C 22.13, %H 1.11, Found %C 22.14, %H 1.17.

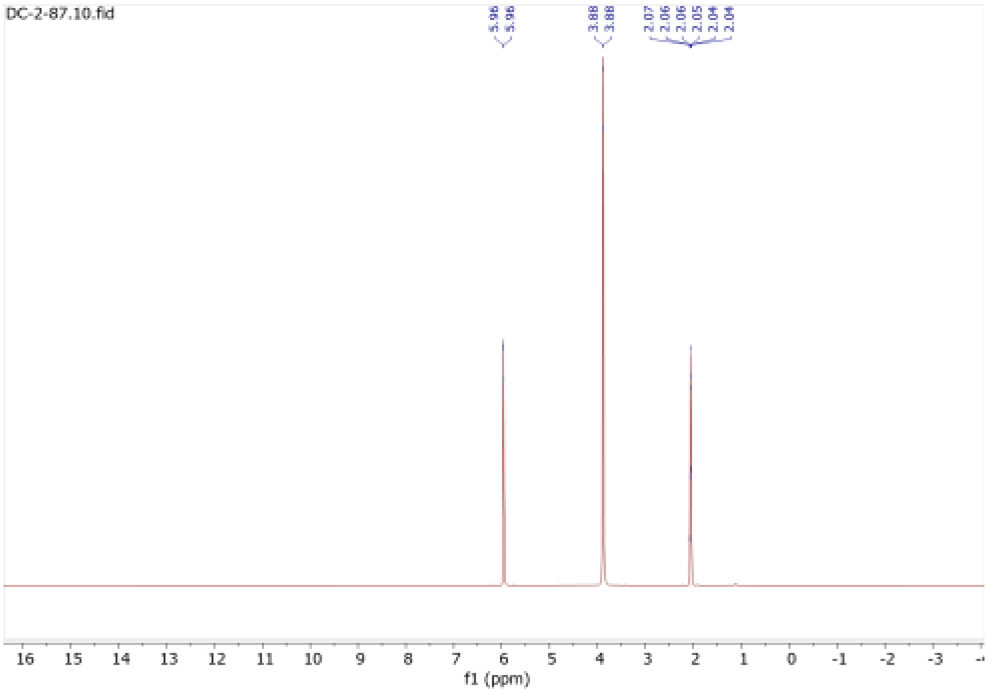


**Figure S28**: $^{1}$H NMR of La(hfac)$_3$(H$_2$O)$_3$ M1-SAG reaction 2 (298 K, 400 MHz, d$_6$-acetone).

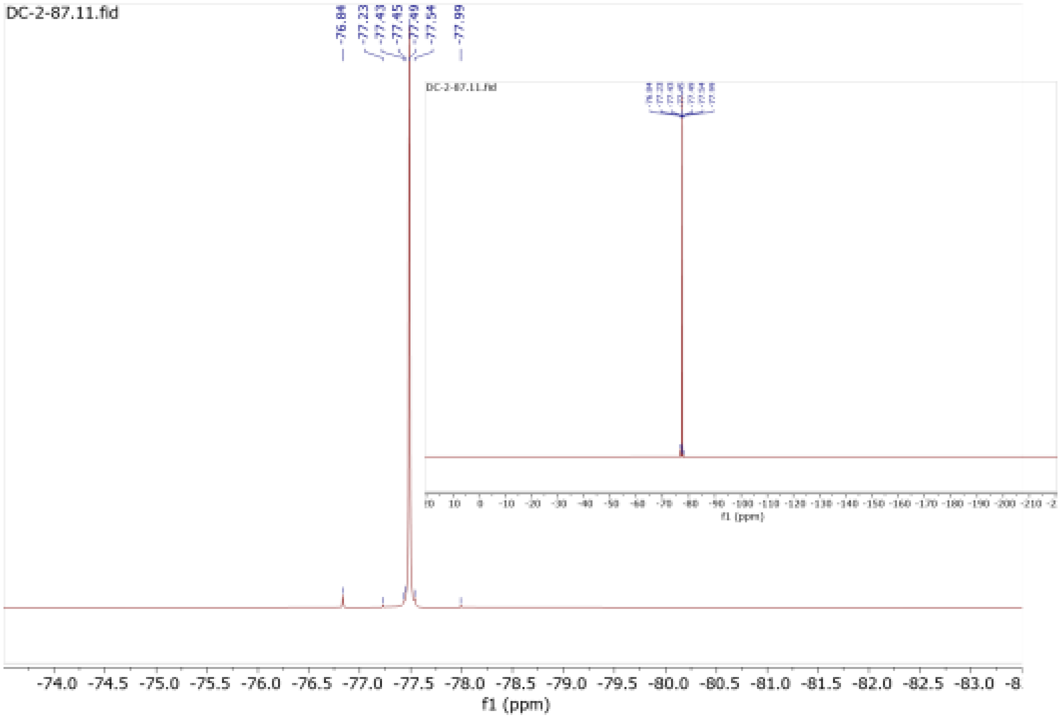


**Figure S29**: $^{19}$F NMR of La(hfac)$_3$(H$_2$O)$_3$ M1-SAG reaction 2 (298 K, 376 MHz, d$_6$-acetone).

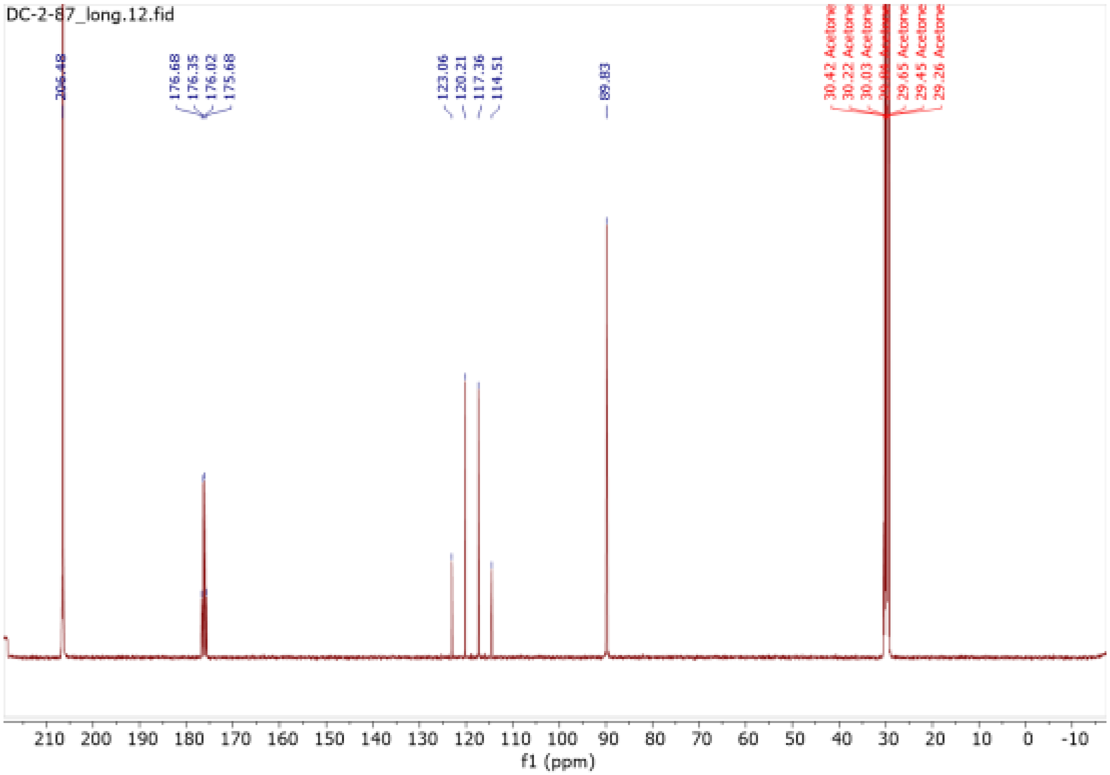


**Figure S30**: $^{13}$C NMR of La(hfac)$_3$(H$_2$O)$_3$ M1-SAG reaction 2 (298 K, 100 MHz, d$_6$-acetone).

**La(hfac)$_3$(H$_2$O)$_3$ mechanochemical synthesis M2 slurry method:**

To a porcelain mortar, $Na_2CO_3{\cdot}H_2O$ (0.5009 g, 4.040 mmol) was added and ground lightly until powdery. Hhfac (1.2 mL, 8.6 mmol) was added and ground until a powdery solid. $LaCl_3(H_2O)_7$ (1.0867 g, 2.9259 mmol) was added along with 2 mL of $Et_2O$. The mixture was ground together for about 5 minutes and then hexanes (2 mL) were added and lightly ground for about 1 minute. The white solid was extracted with 40 mL (2×15 mL + 10 mL) of $Et_2O$. To the $Et_2O$ extracts, 15 mL hexanes was added and concentrated down to yield white waxy solid material. The yield of white solid was 0.7487 g, (0.9197 mmol, 31%). FT-IR (Nujol, cm$^{-1}$): 3663m-sh, 3652m, 3586m, 3528m-br, 3147w, 1653ms-sh, 1649s, 1627ms-sh, 1560ms, 1533ms, 1476s-sh, 1466s*, 1459s-br*, 1366m, 1347m, 1322m, 1254s, 1219s-sh, 1202s-br, 1145vs-br, 1094ms, 1044vw-sh, 1025vw-sh, 948vw, 933vw, 919vw, 890vw-br, *872w*, 847vvw, 837vvw-br, *807m-sh*, 800ms, 768w-br, 760w-br, 741mw, *735w-sh*, 661ms cm$^{-1}$. Elem. Anal. Calcd. for $LaC_{15}H_9F_{18}O_9$: %C 22.13, %H 1.11, Found %C 22.13, %H 1.20. $^1$H NMR (400 MHz, d$_6$-acetone): 5.97 (s), 3.22 (s) ppm. Minor resonances are seen at 1.28 (s, br), 1.10 (t, *J*=7.0 Hz), 0.87 (s, br) ppm. $^{19}$F NMR (376 MHz, d$_6$-acetone): −77.0 (minor), −77.5, −77.6 (small shoulder) ppm.

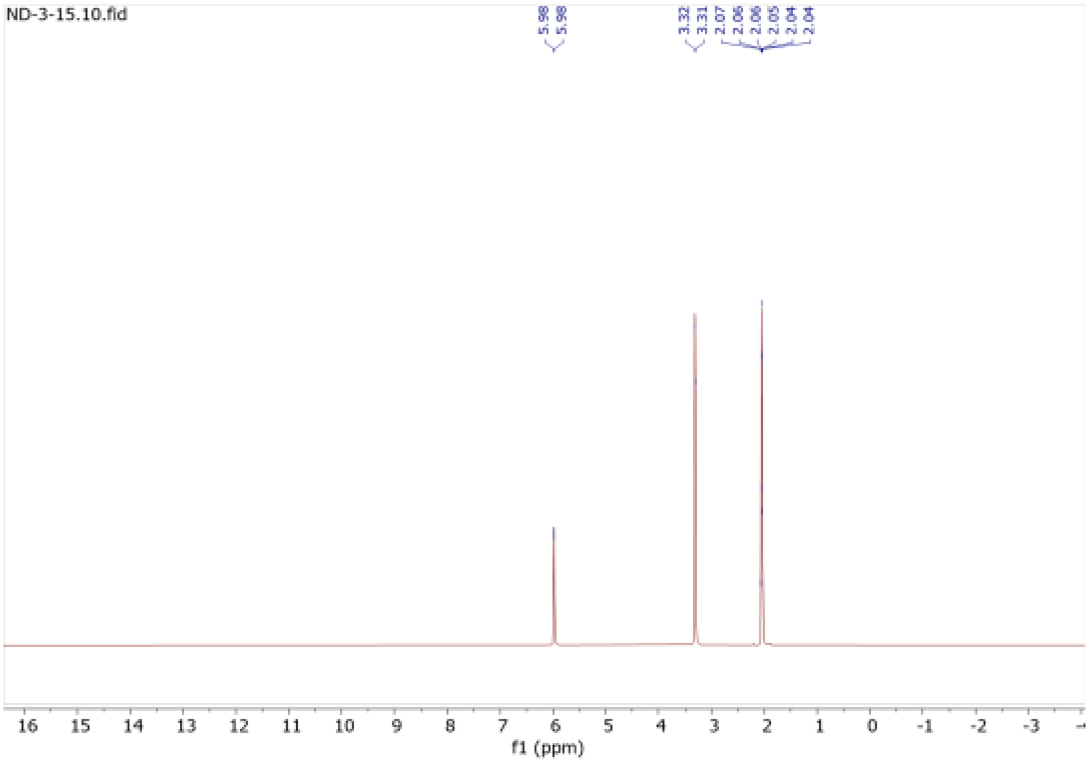


**Figure S31:** $^1$H NMR of M2 slurry La(hfac)$_3$(H$_2$O)$_3$ (298 K, d$_6$-acetone, 400 MHz).

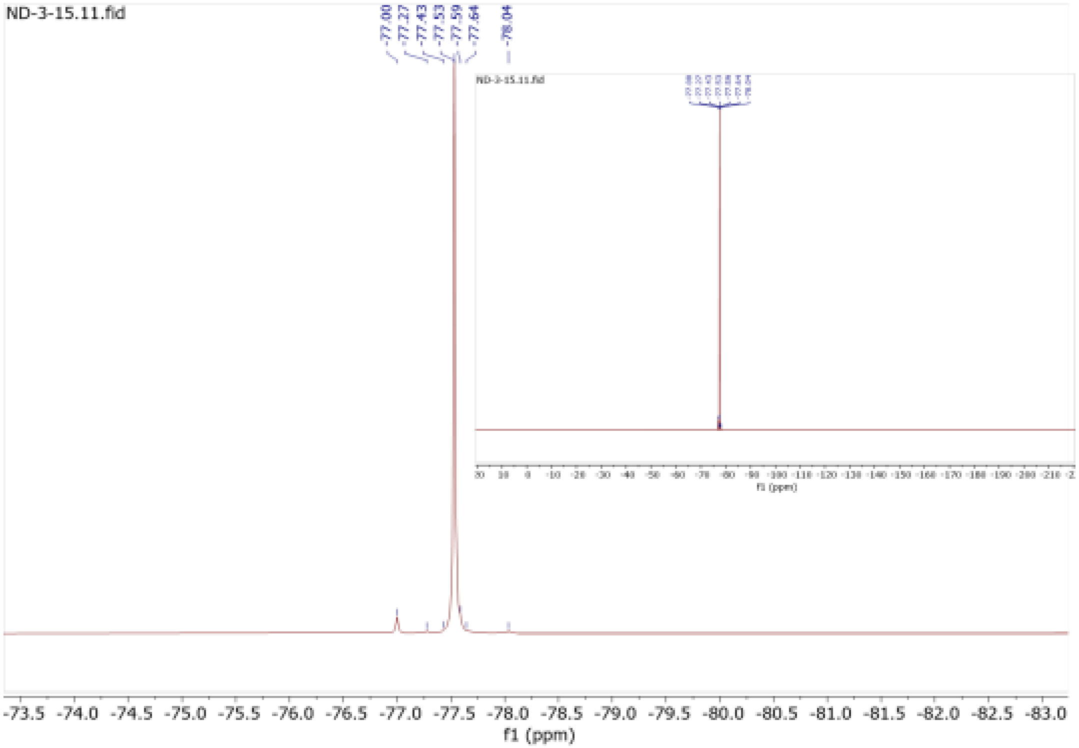


**Figure S32:** $^{19}F$ NMR of M2 slurry La(hfac)$_3$(H$_2$O)$_3$ (298 K, d$_6$-acetone, 376 MHz).

**Ce(hfac)$_3$(H$_2$O)$_3$ solution synthesis:**

In a 250 mL Erlenmeyer flask, $Na_2CO_3 \cdot H_2O$ (0.4994 g, 4.027 mmol) was dissolved in 75 mL of deionized water. To this solution, Hhfac (1.2 mL, 8.6 mmol) was added and allowed to stir for five minutes, following the cessation of bubbles. To this solution $CeCl_3 \cdot H_2O_7$ (1.0067 g, 2.7020 mmol) was added. A yellow suspension was immediately formed, 25 mL of water was added and the solution became transparent. This solution was allowed to stir for 2 hours. Following stirring, the solution was extracted with 3×100 mL portions of $Et_2O$. The $Et_2O$ was then dried over $MgSO_4$. The $MgSO_4$ was washed with an extra 20 mL of $Et_2O$, then concentrated down to an oil. Approximately 10 mL of hexanes was added to facilitate formation of a yellow solid. The reaction yielded 1.4382 g of a pale-yellow solid (1.819 mmol, 67%). FT-IR (Nujol, cm$^{-1}$): 3687vw, 3506mw, 3401mw, 3306w-sh, 3144vw-br, 1655s, 1615mw, 1567m, 1542m, 1493s-br, 1467s*, 1365w, 1351w, 1267s-sh, 1254s-br, 1228s-br, 1209s-br, 1197ms-sh, 1146vs-br, 1100m, 986w, 972vw-br, 950vw, 909w, *871vw-br*, 807m, 774vw, 765vvw-br, 743mw, 742mw, *734vvw*, 670m, 663m cm$^{-1}$. Elem. Anal. Calcd. for $CeC_{15}H_9F_{18}O_9$: %C 22.10, %H 1.11. Found %C 22.47, %H 1.30.

**Ce(hfac)$_3$(H$_2$O)$_3$ M1-SAG:**

To a porcelain mortar, $Na_2CO_3·H_2O$ (0.5015 g, 4.044 mmol) was added and ground lightly until powdery. Hhfac (1.2 mL, 8.6 mmol) was added and ground until a powdery solid. $CeCl_3·H_2O_7$ (1.0067 g, 2.7020 mmol) was added and ground for ~10 min. This was coincident with a color change from colorless $CeCl_3(H_2O)_7$ to yellow Ce(hfac)$_3$(H$_2$O)$_3$. A crude FT-IR spectrum taken matched up well with the La(hfac)$_3$(H$_2$O)$_3$ prepared mechanochemically. The yellow solid was extracted with 3×20 mL of $Et_2O$. The remaining insoluble inorganic material was white. The yellow solution was slowly evaporated to yield a yellow precipitate with a yield of 0.4967 g (0.6092 mmol). To the remaining residue, 10 mL of hexanes were added and a second crop of yellow precipitate with a yield of 0.3708 g (0.4548 mmol) was obtained. These two crops were combined for a total yield of 39%. FT-IR (Nujol, cm$^{-1}$): 3689w, 3602w-sh, 3523w-br, 3319vvw-br, 3143vvw, 1653s-br, 1613mw-br, 1602mw-br, 1561m, 1534m, 1479s-br, 1468s-br*, 1365w, 1349w, 1323w, 1257s-br, 1221s-sh, 1209s-br, 1145vs-br, 1099m-br, 950vw, 933vvw, 918vvw, 890vvw, *870vw*, *807m-sh*, 801ms, 771vw-br, 765w-br, 742mw, *735w-sh*, *664ms-sh*, 662ms cm$^{-1}$. Elem. Anal. Calcd. for $CeC_{15}H_9F_{18}O_9$: %C 22.10, %H 1.11. Found %C 22.22, %H 1.28.

**Ce(hfac)$_3$(H$_2$O)$_3$ M1-SAG reaction 2:**

To a porcelain mortar, $Na_2CO_3·H_2O$ (0.5724 g, 4.616 mmol) was added and ground lightly until powdery. Hhfac (1.3 mL, 9.3 mmol) was added and ground until a powdery solid. $CeCl_3·7H_2O$ (1.1574 g, 3.1064 mmol) was added and ground for ~10 min. This was coincident with a color change from colorless $CeCl_3·7H_2O$ to yellow Ce(hfac)$_3$(H$_2$O)$_3$. The yellow solid was extracted with 3×20 mL of $Et_2O$. In order to facilitate formation of a solid, 15 mL of hexanes was added. The yield of yellow solid was 0.8588 g (1.053 mmol, 34%). FT-IR (Nujol, cm$^{-1}$): 3686mw, 3668mw, 3600mw, 3521mw-br, 3225w-br, 3146vw, 1699mw, 1649s, 1621m-sh, 1561m, 1534m, 1477s-br, 1467s 1366mw, 1349mw, 1324w, 1256s-br, 1224s-sh, 1205s-br, 1147s-br, 1097m-br, 950vw, 935vvw, 919vvw, 890vvw-br, *874vw*, 847vvw-br, 806m-sh, 801m, 771w-br, 741mw, *737mw-sh*, 662ms cm$^{-1}$. Elem. Anal. Calcd. for $CeC_{15}H_9F_{18}O_9$: %C 22.10, %H 1.11. Found %C 22.21, %H 1.14.

**Ce(hfac)$_3$(H$_2$O)$_3$ M2 slurry method:**

To a porcelain mortar, $Na_2CO_3 \cdot H_2O$ (1.0003 g, 8.0669 mmol) was added and ground lightly until powdery. Hhfac (2.3 mL, 16 mmol) was added and ground until a powdery solid. $CeCl_3 \cdot 7H_2O$ (1.9568 g, 5.252 mmol) was added along with 20 mL of $Et_2O$. The mixture was ground together for about 20 minutes and then 10 mL of hexanes were added. The yellow material was ground until dried and allowed to sit overnight. The yellow solid was extracted with 4×20 mL of $Et_2O$. Some yellow color remained in the insoluble extracts. To the yellow solution in $Et_2O$, 10 mL of hexanes were added and allowed to evaporate to yield a yellow crystalline solid. The yield of the yellow crystalline solid was 1.5684 g (1.924 mmol, 37%). FT-IR (Nujol, cm$^{-1}$): 3688w, 3615w-sh, 3523 mw-br, 3144vw, 1699w-sh, 1649s, 1616mw-sh, 1560mw, 1534m, 1482ms-sh, 1462s-br*, 1366mw-sh, 1348w, 1324w, 1256s, 1210s-br, 1146vs, 1100m, 949vvw-br, 918vvw, 890vvw, *871w*, *816w-sh*, *806mw-sh*, 802mw, 772w-br, 742mw, *735w*, 663m cm$^{-1}$. Elem. Anal. Calcd. for $CeC_{15}H_9F_{18}O_9$: %C 22.10, %H 1.11. Found %C 22.19, %H 1.01.

**Ce(hfac)$_3$(H$_2$O)$_3$ M2 slurry reaction 2:**

To a porcelain mortar, $Na_2CO_3 \cdot H_2O$ (1.0062 g, 8.1145 mmol) was added and ground lightly until powdery. Hhfac (2.3 mL, 16 mmol) was added and ground until a powdery solid. $CeCl_3 \cdot 7H_2O$ (2.0024 g, 5.37 mmol) was added along with 2 mL of $Et_2O$. The mixture was ground together for 10 minutes and then 1 mL of hexanes were added. The yellow material was ground until dried (10 min) and then allowed to sit for 10 minutes. The yellow solid was extracted with 3×20 mL of $Et_2O$. To the yellow solution in $Et_2O$, 5 mL of hexanes were added and concentrated down to a yellow crystalline solid. The yield from this reaction was 1.5601 g of yellow material (1.9135 mmol, 36%). FT-IR (Nujol, cm$^{-1}$): 3686m, 3646m, 3517mw-br, 3309mw-br, 3146mw, 1649s, 1624m-sh, 1604m-sh, 1560m, 1533m, 1480s-br, 1462s*, 1367mw-sh, 1348w, 1322w, 1256s, 1218s-sh, 1206s-br, 1144vs, 1095ms, 1043w, 988vvw-br, 949w, 935vvw, 919vvw, 908vvw, 890vvw, *871mw*, 847vw, 836vvw, 816mw-sh, 807m-sh, 800m, 770mw-br, 759mw-sh, 741m, *734mw*, 661ms cm$^{-1}$. Elem. Anal. Calcd. for $CeC_{15}H_9F_{18}O_9$: %C 22.10, %H 1.11. Found %C 22.22, %H 1.01. Re-running the IR spectrum and grinding the larger particles to a finer solid removed the extremely weak 988, 935, 908 cm$^{-1}$ peaks (**See section S7**-notes about IR sample preparation).

**Pr(hfac)$_3$(H$_2$O)$_3$ solution synthesis:**

In a 250 mL Erlenmeyer flask, $Na_2CO_3 \cdot H_2O$ (0.5173 g, 4.172 mmol) was dissolved in 75 mL of deionized water. To this solution, Hhfac (1.2 mL, 8.6 mmol) was added and allowed to stir for five minutes, following the cessation of bubbles. To this solution $PrCl_3 \cdot 7H_2O$ (1.0271 g, 2.748 mmol) was added. A pale green suspension was immediately formed, 25 mL of DI water was added and the solution became transparent. This solution was allowed to stir for 3 hours. Following stirring, the solution was extracted with 3×100 mL portions of $Et_2O$. The $Et_2O$ was dried over $MgSO_4$. The $MgSO_4$ was then washed with an additional 20 mL aliquot of $Et_2O$. The $Et_2O$ evaporated to yield a green oil. Approximately 10 mL of hexanes were added to obtain a green solid. The reaction yielded 1.8221 g of pale green solid (2.2327 mmol, 81%). FT-IR (Nujol, cm$^{-1}$): 3690w, 3593mw-sh, 3508m-br, 3405m-br, 3306mw-sh, 3147w, 1652s-br, 1616mw-sh, 1605mw-sh, 1566ms, 1541ms, 1491s-br, 1464s-br*, 1456s-sh, 1365mw, 1351mw, 1325w, 1266s-sh, 1254s-br, 1221s-sh, 1209s-br, 1144s-br, 1099m, 987w, 971vw, 951vw, 909w, *871vw*, *808ms-br*, *804m-sh*, 802m-sh, 772w, 742mw, *735vw*, 670ms, *668ms*, *664ms*, 662ms cm$^{-1}$. Elem. Anal. Calcd. for $PrC_{15}H_9F_{18}O_9$: %C 22.08, %H 1.11. Found %C 21.83, %H 1.17.

**Pr(hfac)$_3$(H$_2$O)$_3$ M1-SAG:**

To a porcelain mortar, $Na_2CO_3 \cdot H_2O$ (0.5030 g, 4.056 mmol) was added and ground lightly until powdery. Hhfac (1.2 mL, 8.6 mmol) was added and ground until a powdery solid. $PrCl_3 \cdot 7H_2O$ (1.0026 g, 2.6824 mmol) was added and ground for ~20 min. The green solid was extracted with 3×20 mL plus an additional 10 mL of $Et_2O$. An additional 10 mL of hexanes was added to facilitate formation of a solid. The pale green solution of was allowed to evaporate overnight to yield a green precipitate with mass 0.7614 g (0.9330 mmol, 35%). FT-IR (Nujol, cm$^{-1}$): 3689m, 3601m, 3526m-br, 3216w, 3145w, 1646s-br, 1623ms-sh, 1602m, 1561ms, 1534ms, 1486ms-sh, 1464vs-br*, 1456vs, 1365mw, 1349mw, 1323mw, 1255s-br, 1207s-br, 1142vs-br, 1096s, 950w, 891vvw-br, *871w*, 817mw-sh, 808m-sh, 801s, 771mw, 741m, *734mw*, *668s-sp*, *664s-sh*, 661s cm$^{-1}$. Elem. Anal. Calcd. for $PrC_{15}H_9F_{18}O_9$: %C 22.08, %H 1.11. Found %C 21.61, %H 0.93, duplicate %C 21.49, %H 0.93.

**$Pr(hfac)_3(H_2O)_3$ M1-SAG reaction 2:**

To a porcelain mortar, $Na_2CO_3·H_2O$ (0.5004 g, 4.035 mmol) was added and ground lightly until powdery. Hhfac (1.2 mL, 8.6 mmol) was added and ground until a powdery solid. $PrCl_3·7H_2O$ (1.0685 g, 2.8587 mmol) was added and ground for ~20 min. The green solid was extracted with 3×20 mL plus an additional 10 mL of $Et_2O$. An additional 20 mL of hexanes was added to facilitate formation of a solid. This reaction was concentrated down to yield 1.1159 g (1.3673 mmol, 48%) of light green solid. FT-IR (Nujol, $cm^{-1}$): 3690mw, 3602mw, 3528w-br, 3318vw-br, 3204vw-br, 3144vw, 1649s-br, 1624m-sh, 1603mw, 1561m, 1534m, 1482s-sh, 1466s*, 1458s-sh*, 1367mw, 1350w, 1324w, 1259s, 1213s-br, 1146vs, 1098m, 970vvw-br, 950vw, 933vvw-br, 918vvw, 890vvw, *871vw*, 846vvw-br, 802m, 771w, 741mw, *734w*, 662m $cm^{-1}$. Elem. Anal. Calcd. for $PrC_{15}H_9F_{18}O_9$: %C 22.08, %H 1.11. Found %C 21.95, %H 0.90.

**$Pr(hfac)_3(H_2O)_3$ M2 slurry method:**

To a porcelain mortar, $Na_2CO_3·H_2O$ (0.5270 g, 4.250 mmol) was added and ground lightly until powdery. Hhfac (1.2 mL, 8.6 mmol) was added and ground until a powdery solid. $PrCl_3·7H_2O$ (1.0842 g, 2.9007 mmol) was added and ground for 2 minutes followed by addition of 2 mL of $Et_2O$ with grinding. After 1.5 hours following evaporation, 2 mL of hexanes were added, lightly ground and allowed to evaporate. After 2.5 hours, the very moist green solid was extracted with 3×20 mL $Et_2O$. There still appeared to be green material remaining so the solid was extracted with additional 2×15 mL portions of $Et_2O$ followed by 10 mL of hexanes. The pale green solution was concentrated down to yield green precipitate 0.2757 g (0.3378 mmol, 12%). FT-IR (Nujol, $cm^{-1}$): 3687w, 3600w, 3521w-br, 3145vw, 1649s, 1622w-br, 1561m, 1534m, 1479ms-sh, 1466s-br*, 1366w-sh, 1348w, 1322vw-br, 1256s, 1206s-br, 1145s, 1097mw, 950vvw, 917vvw-br, 890vvw, *871vvw-br*, *813w-sh*, 802m, 768vw, 748vw-sh, 741w, *736w-sh*, 662m $cm^{-1}$. Elem. Anal. Calcd. for $PrC_{15}H_9F_{18}O_9$: %C 22.08, %H 1.11. Found %C 21.92, %H 0.99.

**$Pr(hfac)_3(H_2O)_3$ M2 slurry reaction 2:**

To a porcelain mortar, $Na_2CO_3·H_2O$ (0.559 g, 4.51 mmol) was added and ground lightly until powdery. Hhfac (1.3 mL, 9.3 mmol) was added and ground until a powdery solid. $PrCl_3·7H_2O$ (1.1242 g, 2.9007 mmol) was added followed by addition of 2 mL of $Et_2O$ with grinding for 15 min. After allowing the mixture to sit for 20 min, 1 mL of hexanes was added, lightly ground and

allowed to evaporate. The dried solid was extracted with 3×20 mL $Et_2O$. To facilitate solid formation 10 mL of hexanes was added. Upon concentration to a solid, this reaction yielded 0.8681 g of green solid (1.0637 mmol, 35%). FT-IR (Nujol, $cm^{-1}$): 3618mw, 3420mw-br, 3223vw-br, 3144w, 1718m, 1700ms, 1661ms-sh, 1653s, 1627m-sh, 1613mw-sh, 1559ms, 1538ms-sh, 1534ms, 1506m-sh, 1489ms-sh, 1459s-br*, 1366ms-sh, 1349m, 1321mw, 1260s-br, 1226s-sh, 1207s-br, 1149s-br, 1095ms, 972vw-br, 949w, 934vvw, 918vvw, 890vw, 845vw-br, 801ms, 770w, 741mw, 663ms $cm^{-1}$. No peak *ca.* 3690 $cm^{-1}$ observed (trihydrate). Sample also contains solvent. Elem. Anal. Calcd. for $PrC_{15}H_9F_{18}O_9$: %C 22.08, %H 1.11. Elem. Anal. Calcd. for $PrC_{15}H_7F_{18}O_8$ (dihydrate): %C 22.57, %H 0.88. Found %C 22.86, %H 0.85, duplicate %C 22.96, %H 0.79.

**$Nd(hfac)_3(H_2O)_3$ solution synthesis:**

In a 250 mL Erlenmeyer flask, $Na_2CO_3{\cdot}H_2O$ (0.5024 g, 4.052 mmol) was dissolved in 75 mL of deionized water. To this solution, Hhfac (1.3 mL, 9.3 mmol) was added and allowed to stir for five minutes. Following the cessation of bubble formation, $NdCl_3{\cdot}7H_2O$ (1.2400 g, 3.292 mmol) was added. An additional 25 mL of DI water was added to obtain a transparent solution. This solution was allowed to stir overnight (18 hours). Following stirring, the solution was extracted with 3×100 mL portions of $Et_2O$. The $Et_2O$ was dried over $MgSO_4$. The $MgSO_4$ was washed with 20 mL of $Et_2O$. Approximately 10 mL of hexanes were added to the solution to facilitate formation of a lilac solid by slow evaporation. The yield was 1.6608 g (2.0267 mmol, 62%). FT-IR (Nujol, $cm^{-1}$): 3499w-br, 3394w-br, 3294w-br, 1653ms, 1648ms, 1614mw-sh, 1564m, 1558m-sh, 1539m, 1506m-sh, 1493ms-br, 1465vs*, 1457s*, 1366mw-sh, 1351w-br, 1268s-sh, 1256s-br, 1208s, 1200s-sh, 1144s-br, 1097mw, 987w, 971vvw-br, 950vw, 908vw, 893vvw-br, 860vvw-br, *807ms-br*, 803m-sh, 744w, 741w, *670mw-sh*, *665m-sh*, 661m $cm^{-1}$. Elem. Anal. Calcd. for $NdC_{15}H_9F_{18}O_9$: %C 21.99, %H 1.11. Found %C 22.19, %H 1.30.

**$Nd(hfac)_3(H_2O)_3$ M1-SAG:**

To a porcelain mortar, $Na_2CO_3{\cdot}H_2O$ (0.5318 g, 4.29 mmol) was added and ground lightly until powdery. Hhfac (1.3 mL, 9.3 mmol) was added and ground until a powdery solid. $NdCl_3{\cdot}7H_2O$ (1.1329 g, 3.01 mmol) was added and ground for ~30 min. The solid was extracted with 3×20 mL portions of $Et_2O$. Approximately 10 mL of hexanes were added to facilitate formation of a solid precipitate through slow evaporation. The pale purple-pink solution was allowed to evaporate overnight to yield a light-colored lilac solid 0.7316 g (0.8928 mmol, 30%). FT-IR (Nujol, $cm^{-1}$):

3690mw, 3599mw, 3567mw, 3504mw-br, 3327w-br, 3141vw, 1654m-sh, 1647ms, 1601w, 1562mw, 1533mw, 1486m-br, 1458m-br*, 1364vw, 1345vw-br, 1320vw, 1257s, 1212s, 1198ms-sh, 1176mw-sh, 1145vs, 1098mw, 1012vw-br, 961vw-br, 957vw-sh, 950vw, 894vvw-br, *871vw*, 840vvw-br, 802mw, 800mw-sh, 768w, 741w, *734w*, *665m-sh*, 661m $cm^{-1}$. Elem. Anal. Calcd. for $NdC_{15}H_9F_{18}O_9$: %C 21.99, %H 1.11. Found %C 24.48, %H 1.11.

**Nd(hfac)$_3$(H$_2$O)$_3$ M1-SAG reaction 2:**

To a porcelain mortar, $Na_2CO_3 \cdot H_2O$ (0.5584 g, 4.502 mmol) was added and ground lightly until powdery. Hhfac (1.3 mL, 9.3 mmol) was added and ground until a powdery solid. $NdCl_3 \cdot 7H_2O$ (1.132 g, 3.005 mmol) was added and ground for 30 min. The solid was extracted with 3×20 mL portions of $Et_2O$. Approximately 10 mL of hexanes were added to facilitate formation of a solid. The solution was concentrated down to a light lilac solid with a mass of 1.2018 g (1.4666 mmol, 47%). FT-IR (Nujol, $cm^{-1}$): 3690vw, 3640vw, 3600vw, 3405vw-br, 3208vw-br, 3146vw, 1700w, 1648ms, 1616mw, 1604w-sh, 1562m, 1534m, 1483ms-sh, 1464vs-br*, 1367mw, 1349mw, 1324w, 1260s-br, 1222ms-sh, 1208ms-br, 1148vs-br, 1097m, 1061mw, 1035vw, 949vw, 928vw, 889vvw-br, 833vw, 809mw, 802m, 769w, 742mw, 662m $cm^{-1}$. Elem. Anal. Calcd. for $NdC_{15}H_9F_{18}O_9$: %C 21.99, %H 1.11. Found %C 22.25, %H 1.13.

**Nd(hfac)$_3$(H$_2$O)$_3$ M2 slurry method:**

To a porcelain mortar, $Na_2CO_3 \cdot H_2O$ (0.4929 g, 3.975 mmol) was added and ground lightly until powdery. Hhfac (1.2 mL, 8.6 mmol) was added and ground until a powdery solid. $NdCl_3 \cdot H_2O_7$ (1.0210 g, 2.7468 mmol) was added and ground for ~2 min. To this pale solid was added 2 mL of $Et_2O$ and ground for 1 minute. The solid dried after about 15 min. After which 2 mL of hexanes was added, lightly ground and then allowed to dry. The solid was extracted with one 25 mL portion followed by one 15 mL portion of $Et_2O$. The pale purple-pink solution was concentrated down to a light purple oil. To this oil, 10 mL of hexanes was added and concentrated down to form a purple-pink solid with a yield of 0.9370 g (1.143 mmol, 42%). FT-IR (Nujol, $cm^{-1}$): 3689mw, 3600mw, 3529mw-br, 3315w-br, 3143w, 1654s, 1647s, 1621w-br, 1602w, 1560m, 1533m, 1484s-br, 1466s-sh*, 1458ms-sh*, 1367w-sh, 1347w, 1341w-sh, 1324vw, 1259s-br, 1212s, 1147s, 1098mw, 950vvw, 891vvw-br, *874vvw*, 802m, 768vw-br, 741w, *664m-sh*, 662m $cm^{-1}$. Elem. Anal. Calcd. for $NdC_{15}H_9F_{18}O_9$: %C 21.99, %H 1.11. Found %C 22.04, %H 1.00.

**Sm(hfac)$_3$(H$_2$O)$_2$ solution synthesis:**

In a 250 mL Erlenmeyer flask, $Na_2CO_3 \cdot H_2O$ (0.5118 g, 4.13 mmol) was dissolved in 100 mL of deionized water. To this solution, Hhfac (1.2 mL, 8.6 mmol) was added and allowed to stir for five minutes following the cessation of bubbles. To this solution $SmCl_3 \cdot 7H_2O$ (1.0545 g, 2.75 mmol) was added. An additional 50 mL of DI water was added to obtain a transparent solution. This solution was allowed to stir overnight (18 hours). Following stirring, the solution was extracted with 3×100 mL portions of $Et_2O$. The $Et_2O$ layer was then dried over $MgSO_4$. An additional 20 mL of $Et_2O$ was used to elute any product from the $MgSO_4$ solid. Approximately 10 mL of hexanes was added to assist in yielding a beige solid by slow evaporation. The yield of beige solid was 1.1454 g (1.4184 mmol, 52%). FT-IR (Nujol, $cm^{-1}$): 3505mw-br, 3404mw-br, 3290mw-br, 3151w, 1651m-sh, 1648ms, 1617mw-br, 1567m, 1540m, 1506m-sh, 1496ms-br, 1472ms-br, 1465ms-br*,1457ms*, 1436mw-br, 1374w-br, 1362w, 1351w-br, 1254s-br, 1225ms, 1207ms, 1201ms-sh, 1178mw, 1146vs, 1111mw, 1099mw, 988w-br, 951vw, 908vw, *869vvw*, 810m, *807m-sh*, 773w, 763vw, 744w, *734vvw-sh*, 720vw, *670mw-sh*, 663m $cm^{-1}$. Elem. Anal. Calcd. for $SmC_{15}H_7F_{18}O_8$: %C 22.31, %H 0.87. Found %C 22.05, %H 1.19.

**Sm(hfac)$_3$(H$_2$O)$_2$ solution reaction 2:**

In a 250 mL Erlenmeyer flask, $Na_2CO_3 \cdot H_2O$ (0.5289 g, 4.265 mmol) was dissolved in 100 mL of deionized water. To this solution, Hhfac (1.3 mL, 9.3 mmol) was added and allowed to stir for five minutes following the cessation of bubbles. To this solution $SmCl_3 \cdot 7H_2O$ (1.1267 g, 2.943 mmol) was added. The $SmCl_3 \cdot 7H_2O$ material dissolved, the solution then became temporarily cloudy. After a couple of minutes, the solution became transparent. This solution was allowed to stir for two hours. Following stirring, the solution was extracted with one 100 mL and one 75 mL portion of $Et_2O$. The $Et_2O$ layer was then dried over $MgSO_4$. An additional 30 mL of $Et_2O$ was used to elute any product from the $MgSO_4$ solid. Approximately 5 mL of hexanes was added to assist in yielding a beige waxy solid with rotary evaporation. Yield for the reaction was 1.9529 g (assuming only Sm(hfac)$_3$(H$_2$O)$_2$ 2.4183 mmol, 82%). Based on FT-IR and EA, this material is contaminated with high amounts of tetraol. FT-IR (Nujol, $cm^{-1}$): 3495m-sh, 3400m-br, 3295m-sh, 3152w, 1658ms-sh, 1648s, 1619m, 1567ms, 1541ms, 1493ms-br, 1466s-sh*, 1457s*, 1366mw, 1351mw, 1338mw, 1267s-sh, 1256 s-br, 1224s, 1207s, 1178ms-sh, 1145vs-br, 1100ms, 987mw, 971w-sh, 950vvw-sh, 908mw, 822vw-sh, 809ms, 772vw, 744mw, *670ms-sh*, 664ms $cm^{-1}$. Elem. Anal.

Calcd. for $SmC_{15}H_7F_{18}O_8$: %C 22.31, %H 0.87. Found %C 24.02, %H 1.67. This compound contains large amounts of tetraol.

**$Sm(hfac)_3(H_2O)_2$ M1-SAG:**

To a porcelain mortar, $Na_2CO_3{\cdot}H_2O$ (0.5174, 4.173 mmol) was added and ground lightly until powdery. Hhfac (1.2 mL, 8.6 mmol) was added and ground until a powdery solid. $SmCl_3{\cdot}7H_2O$ (1.1072 g, 2.892 mmol) was added and ground for ~20 min, then allowed to sit overnight (~ 18 hours). The light-beige solid was extracted with 3×20 mL portions of $Et_2O$. Approximately 10 mL of hexanes was added to the $Et_2O$ soluble material to assist in solid formation through slow evaporation. The yield of beige solid was 0.6052 g (0.7494 mmol, 26%). FT-IR (Nujol, $cm^{-1}$): 3457m-br, 3146w, 1651m, 1647ms, 1635mw-sh, 1618mw-br, 1568mw-br, 1540mw-br, 1538m, 1488mw-sh, 1458ms-br*, 1434mw-br, 1366w-sh, 1349w-br, 1327vw-br, 1255ms-br, 1225ms-br, 1200ms-br, 1143vs-br, 1109mw, 1098m, 950vvw, 810m, 773vw-br, 770vw-sh, 744w, *734vw-sh*, 662m $cm^{-1}$. Elem. Anal. Calcd. for $SmC_{15}H_7F_{18}O_8$: %C 22.31, %H 0.87. For the retro-Claisen impurity, %C 20.20, %H 0.84. Found %C 21.08, %H 0.93, duplicate %C 20.97, %H 0.95.

**$Sm(hfac)_3(H_2O)_2$ M1 SAG reaction 2:**

To a porcelain mortar, $Na_2CO_3{\cdot}H_2O$ (0.5008 g, 4.038 mmol) was added and ground lightly until powdery. Hhfac (1.2 mL, 8.6 mmol) was added and ground until a powdery solid. $SmCl_3{\cdot}7H_2O$ (1.0962 g, 2.8634 mmol) was added and ground for 20 min. The light-beige solid was extracted with 3×20 mL + one 5 mL portions of $Et_2O$. Approximately 15 mL of hexanes was added to the $Et_2O$ soluble material to assist in solid formation. The solution was concentrated down to yield 0.8629 g of beige solid (1.068 mmol, 37%). FT-IR (Nujol, $cm^{-1}$): 3604w-br, 3518w-br, 3150vw, 1702mw, 1662m, 1649ms, 1620m, 1568m, 1562mw-sh, 1541m, 1463vs-sh*, 1457vs-sh*, 1366m, 1352mw-sh, 1341mw-sh, 1326mw-br, 1257ms, 1227ms, 1202ms, 1146s, 1101m, 1097ms, 970w-br, 950w, 937vw, 918vw, 890w, *870vvvw-br,* 846vw, 811m, 773w, 744mw, 663m $cm^{-1}$. Elem. Anal. Calcd. for $SmC_{15}H_7F_{18}O_8$: %C 22.31, %H 0.87. Found %C 22.46, %H 1.19.

**$Sm(hfac)_3(H_2O)_2$ M2 slurry method:**

To a porcelain mortar, $Na_2CO_3{\cdot}H_2O$ (0.5161 g, 4.162 mmol) was added and ground lightly until powdery. Hhfac (1.2 mL, 8.6 mmol) was added and ground until a powdery solid. $SmCl_3{\cdot}7H_2O$ (1.0915 g, 2.8511 mmol) was added and ground for 1 minute followed by addition of 5 mL of $Et_2O$

and ground gently for a couple of minutes, the suspension was evaporated over 1.5 hours. Following evaporation of the $Et_2O$, 2 mL of hexanes were added and ground for five minutes. After 1 hour, the light beige solid was extracted with 2×15 mL portions and a 10 mL portion of $Et_2O$. To this $Et_2O$ solution, 10 mL of hexanes were added. The nearly colorless solution was concentrated down to yield a pale light-yellow oil. An additional, 2 mL of hexanes was added to the oil and concentrated down to yield a pale beige solid 0.7612 g (0.9426 mmol, 33%). FT-IR (Nujol, $cm^{-1}$): 3588mw-sh, 3486s-br, 3255w-sh, 3151w, 1648s, 1619ms, 1582m-sh, 1568ms, 1541ms, 1462vs-br*, 1366ms-sh, 1352ms, 1327ms, 1256s-br, 1224s-br, 1202s-br, 1144vs-br, 1110ms, 1099s, 951w, 918vw, 890vw-br, 848vw-br, 811s, 773w, 744m, 663s $cm^{-1}$. Elem. Anal. Calcd. for $SmC_{15}H_7F_{18}O_8$: %C 22.31, %H 0.87. Found %C 22.02, %H 0.85.

**Table S8:** Yields of isolated products containing $Ln(hfac)_3(H_2O)_{2,3}$ from various synthetic methods (parentheses indicate yields from additional reactions)[a]

| Lanthanide | Solution | M1 (SAG) mortar and pestle[a] | Yield (M1-SAG with only 70% Hhfac)[b] | M2 (slurry) mortar and pestlel[a] | Yield M2 with only 70% Hhfac[b] |
|---|---|---|---|---|---|
| **La** | 76%, | 19% (50%) | 30% (69%) | 31% | 46% |
| **Ce** | 67% | 39% (34%) | 53% (54%) | 37% (36%) | 51 (51%) |
| **Pr** | 81% | 35% (48%) | 46% (68%) | 12% (35%) | 17 (49%) |
| **Nd** | 62% | 30% (47%) | 41% (68%) | 42% | 57% |
| **Sm** | 52% | 26% (37%) | 37% (53%) | 33% | 47% |
| **Tb** | 75% | 33%, (45%, 34%, 46%) | 50% (60%, 45%, 65%) | 44%[c] | 65%[c] |

**[a]** Yields are based on mass of $Ln(hfac)_3(H_2O)_x$ (x=2,3) and assuming the $LnCl_3(H_2O)_x$ starting material was the limiting reagent. Using the $LnCl_3$ results in an apparent lowering of the yield of the open mortar and pestle reactions.

**[b]** The 70% value was selected as that is a possible maximum amount of soluble Na(hfac) material based on optimization studies. **[c]** Hexanes not added to this reaction.

## S5b. Two-pot mechanochemical synthesis with recrystallized Na(hfac)

### Synthesis of recrystallized Na(hfac)

To a porcelain mortar, in the fumehood, $Na_2CO_3{\cdot}H_2O$ (0.9906 g, 7.989 mmol) was added and ground lightly until powdery. Hhfac (2.3 mL, 16.5 mmol) was added and ground for 15 minutes until a powdery solid. The solid was dissolved in slightly warmed acetone. Insoluble inorganic material was filtered out. The product crystallized out of acetone as a white solid. The yield of white crystalline material was 56% (2.1352 g, 9.2819 mmol). $^1H$ NMR ($d_6$-acetone, 298 K): 5.60

(s, hfac-H), 2.98 (s, $H_2O$) ppm. $^{19}$F NMR ($d_6$-acetone, 298 K, unreferenced): −75.72 (minor), −77.54 ppm.

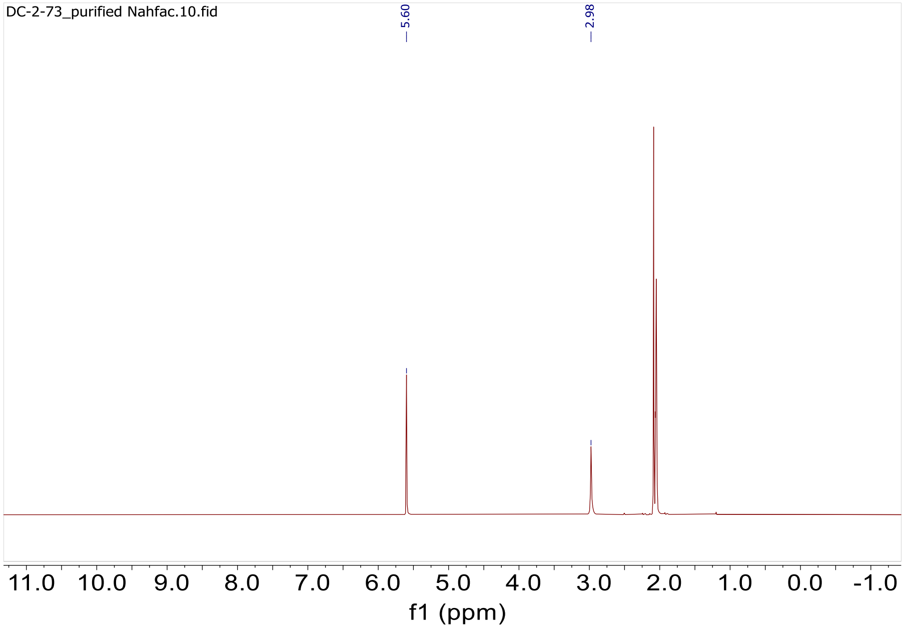


**Figure S33:** $^{1}$H NMR Spectrum of recrystallized Na(hfac) (298 K, $d_6$-acetone, 400 MHz).

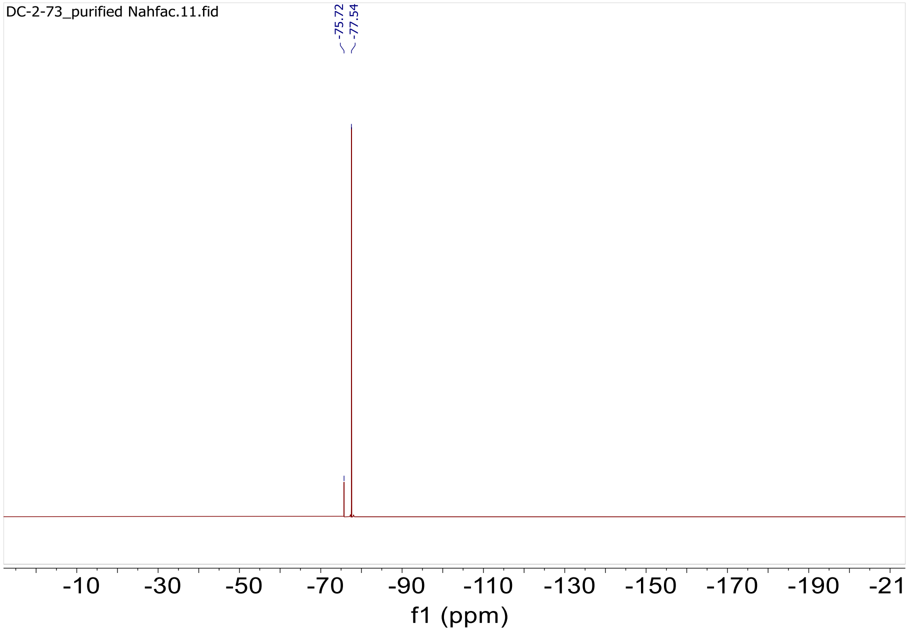


**Figure S34:** $^{19}$F NMR Spectrum of recrystallized Na(hfac) (298 K, $d_6$-acetone, 376 MHz).

**$La(hfac)_3(H_2O)_3$ modified M1-SAG (I)**

A modified version of SAG (M1) using Na(hfac) recrystallized from acetone was conducted. A porcelain mortar was charged with purified Na(hfac) (0.2880 g, 1.252 mmol), $LaCl_3{\cdot}H_2O_7$ (0.1527 g, 0.411 mmol) was then added and ground for 15 minutes. The product was extracted with 2×10 mL of $Et_2O$. To the $Et_2O$ extract, 20 mL of hexanes was added to facilitate formation of a white solid (0.1547 g, 0.1900 mmol) with a 46% yield. FT-IR (Nujol, $cm^{-1}$): 3674w, 3530w-br, 3181vw-br, 3143vw, 1671mw-sh, 1649s-br, 1612w, 1561m, 1535m, 1490s-sh, 1482 s-sh, 1465s-br*, 1398w, 1367w, 1346w, 1322w, 1255s-br, 1210s-br, 1146vs-br, 1094m-br, 1062w, 1038w, 948vw, 916vvw, 883vvw, *872w*, 847vw, 804m, 769vw-br, 741w, 662ms $cm^{-1}$. $^1H$ NMR ($d_6$-acetone, 298 K): 5.97 (s, 3H, presumed hfac-H), 3.04 (s, 9H, $H_2O$) ppm. Additional peaks presumed to be ethanol were observed at: 3.58 (m-br, 2H), 3.42 (s-br, 1H), 1.12 (t, 3H, 6.3 Hz). $^{19}F$ NMR ($d_6$-acetone, 298 K, unreferenced): −77.05 (minor), −77.28 (minor), −77.53 (minor), −77.65, −78.04 (minor) ppm. Elem. Anal. Calcd. for $LaC_{15}H_9F_{18}O_9$: %C 22.13, %H 1.11. Found %C 22.80, %H 1.18. Some small amount of solvent present in sample.

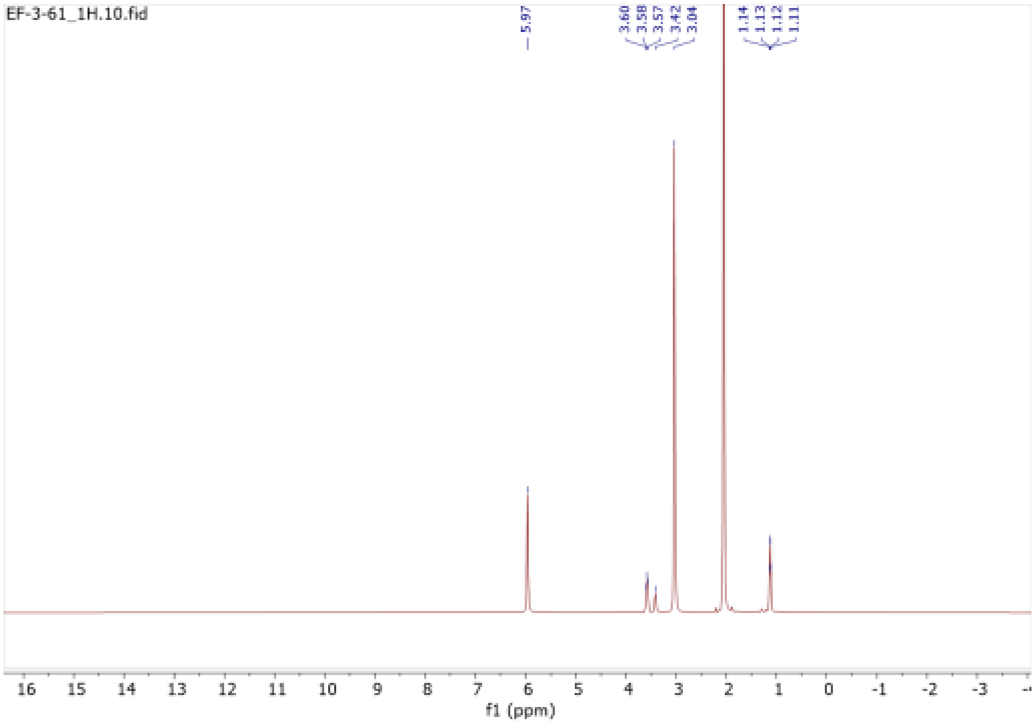


**Figure S35:** $^1H$ NMR of modified M1-SAG $La(hfac)_3(H_2O)_3$ (298 K, $d_6$-acetone, 400 MHz).

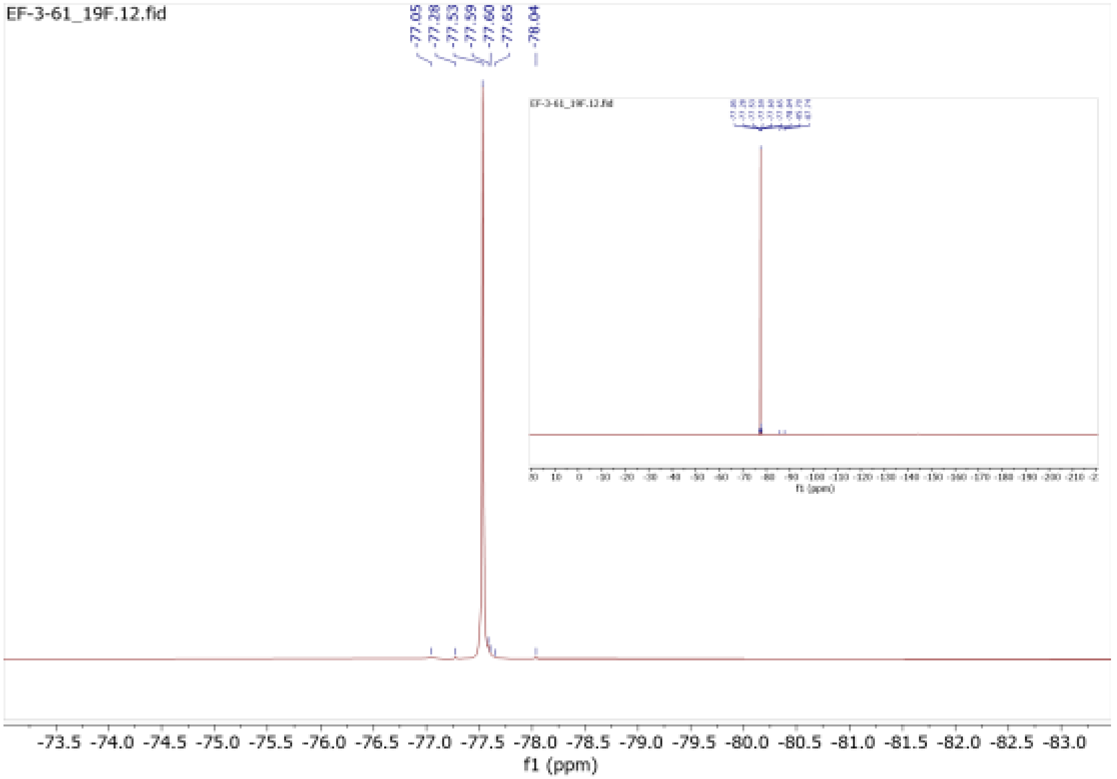


**Figure S36:** $^{19}$F NMR of modified M1-SAG La(hfac)$_3$($H_2O$)$_3$ (298 K, d$_6$-acetone, 376 MHz).

**Ce(hfac)$_3$($H_2O$)$_3$ modified M1-SAG (I)**

A porcelain mortar was charged with purified Na(hfac) (0.8238 g, 3.581 mmol), $CeCl_3{\cdot}H_2O_7$ (0.4467 g, 1.1989 mmol) was then added and ground for approximately 15 minutes. The product was extracted with 30 mL of $Et_2O$. To the $Et_2O$ extract, 10 mL of hexanes was added. Slow evaporation of the $Et_2O$ with hexanes yielded crystalline materials in the form of yellow plates and yellow blocks. The yellow plates were found to be the impurity [Ce(hfac)$_2$($CF_3COO$)($H_2O$)$_2$]$_2$ and the block crystals were found to be Ce(hfac)$_3$($H_2O$)$_3$ from single crystal X-ray Diffraction. The compounds could not be separated for bulk analysis. FT-IR (Nujol, cm$^{-1}$): 3688vw, 3509w-br, 3212vw-br, 3143vw, 1679m-sh, 1648s-br, 1561m, 1538m, 1480s-sh, 1459s-br*, 1366w, 1348w, 1325w, 1257s-br, 1211s-br, 1147vs-br, 1099m-br, 949vw, 935vvw, 917vvw, 889vvw, *871w*, 844vw, *807mw-sh*, 802m, 773vw-br, 765vvw-br, 742w, *735mw*, *664m-sh*, 662ms cm$^{-1}$. Elem. Anal. Calcd. for $CeC_{15}H_9F_{18}O_9$: %C 22.10, %H 1.11. For the retro-Claisen impurity, %C 20.49, %H 0.86. Found %C 21.09, %H 0.89, duplicate %C 21.10, %H 0.89. Low %C expected for bulk material based on presence of substantial amount of retro-Claisen impurity.

**Ce(hfac)$_3$(H$_2$O)$_3$ modified M1 SAG reaction 2:**

Repeating this reaction including slow evaporation of Et$_2$O and hexanes yielded primarily yellow blocks with some plates. A yield was not obtained for the recrystallization in order to not disturb material needed for single crystal analysis. FT-IR (Nujol, cm$^{-1}$): 3689mw-sp, 3602m-sp, 3504m-br, 3200vw-br, 3144w, 1655s-sh, 1649s-br, 1629ms-sh, 1567m, 1540m, 1482s-sh, 1458s-br*, 1366mw, 1351mw, 1324mw, 1258s-br, 1234s-br, 1211s-br, 1197s-br, 1173m-sh, 1143s-br, 1097ms, 972vvw-br, 950w, 918vvw-br, 891vw-br, *871mw*, 843vw-br, *812m*, 808m, 803ms, 772w-br, 742mw, *735m*, *664s*, 661s cm$^{-1}$. Elem. Anal. Calcd. for mixture CeC$_{15}$H$_9$F$_{18}$O$_9$: %C 22.10, %H 1.11. For the retro-Claisen impurity, %C 20.49, %H 0.86. Found %C 21.07, %H 0.98.

Block crystals were obtained and analyzed separately: FT-IR (Nujol, cm$^{-1}$): 3689mw-sp, 3602mw-sp, 3529mw-br, 3210vvw-br, 3141vw, 1652s-br, 1614mw-sh, 1603mw, 1561m, 1534m, 1477s-sh, 1468s-br, 1366w, 1349w, 1324w, 1258s-br, 1214s-br, 1147s-br, 1109mw-sh, 1098mw, 950vw, 918vvw-br, 891vvw-br, 845vvw-br, 802m, 770w, 741mw, 663m cm$^{-1}$. A small amount (~0.7 mg) was submitted for elemental analysis with low %C. The isolated amount of material was too small for elemental analysis. $^1$H NMR (d$_6$-acetone, 298 K): 11.27 (s, presumed hfac-H), 2.94 (s, presumed H$_2$O) ppm. $^{19}$F NMR (d$_6$-acetone, 298 K, unreferenced): −76.08, −76.74 (minor), −77.53 (minor) ppm. $^{13}$C NMR (d$_6$-acetone, 298 K): 187.8 (q, $J_{C\text{-}F}$=34 Hz), 130.2 (q, $J_{C\text{-}F}$=284 Hz), 105.5 ppm.

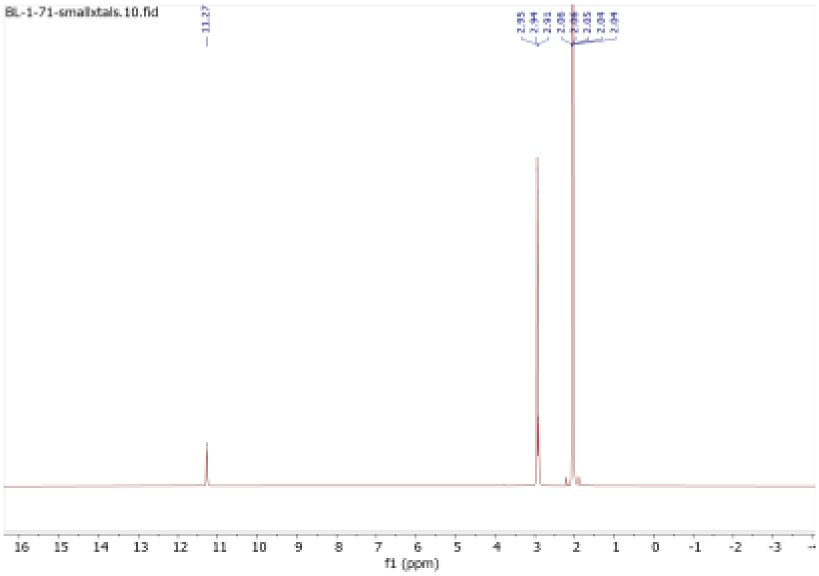


**Figure S37**: $^1$H NMR of crystals of Ce(hfac)$_3$(H$_2$O)$_3$ from modified M1-SAG synthesis (298 K, 400 MHz, d$_6$-acetone).

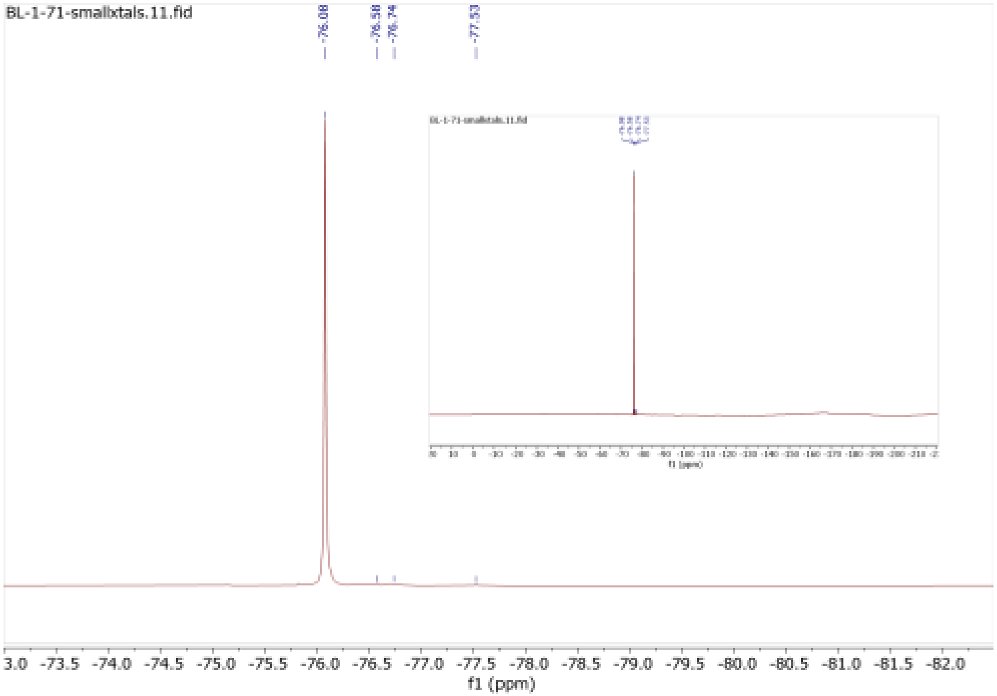


**Figure S38**: $^{19}F$ NMR of crystals of $Ce(hfac)_3(H_2O)_3$ from modified M1-SAG synthesis (298 K, 376 MHz, $d_6$-acetone).

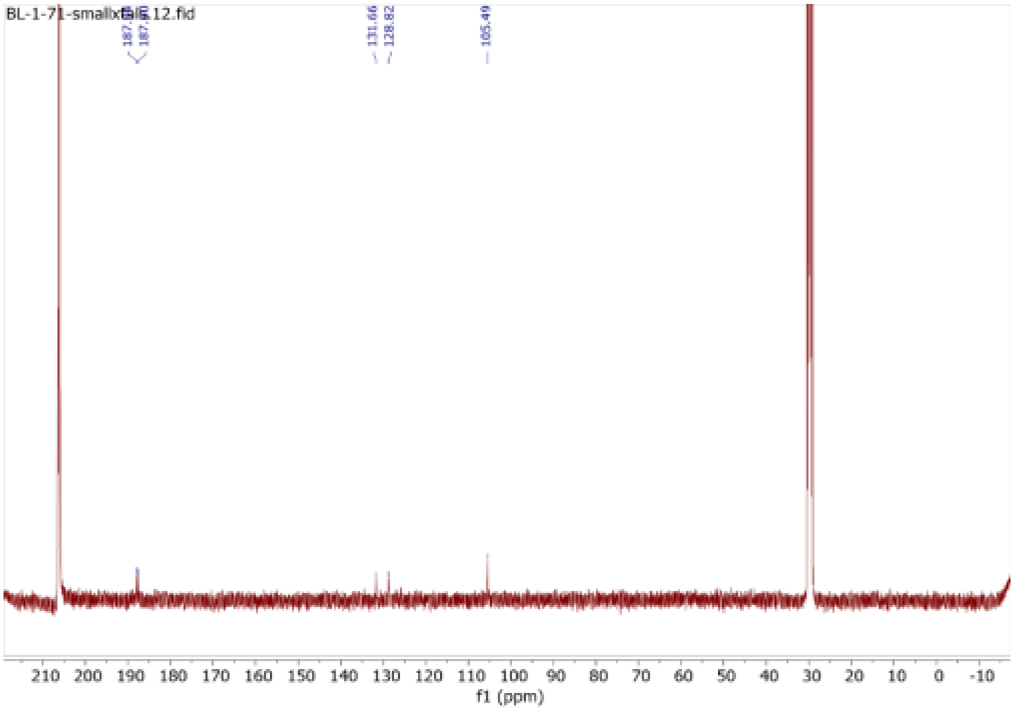


**Figure S39**: $^{13}C$ NMR of crystals of $Ce(hfac)_3(H_2O)_3$ from modified M1-SAG synthesis (298 K, 100 MHz, $d_6$-acetone).

**$Ce(hfac)_3(H_2O)_3$ modified M1 SAG reaction 3:**

A third reaction was performed using 20 mL of $Et_2O$ for extraction and 10 mL of hexanes. A crude yield was not obtained. Slow evaporation from a solution of $Et_2O$ and hexanes yielded, by eye, solely yellow needle crystals. FT-IR (Nujol, $cm^{-1}$): 3502mw-br, 3194vw-br, 3148vw, 1651ms-sh, 1648ms, 1637mw-sh, 1564mw-sh, 1559m, 1539m, 1534m, 1507mw, 1486ms-sh, 1464vs-sh*, 1458vs*, 1418vw, 1365mw-br, 1349w-br, 1339w-br, 1256s-br, 1209ms-br, 1145vs-br, 1097m, 971vvw-br, 949vvw, 932vvw-br, 918vvw-br, *870w*, 807mw-sh, 802mw, 771w-br, 742w, *734w*, 662m $cm^{-1}$. Elem. Anal. Calcd. for $CeC_{15}H_9F_{18}O_9$: %C 22.10, %H 1.11. Found %C 22.05, %H 1.00.

**$Ce(hfac)_3(H_2O)_3$ modified M1 SAG reaction 4:**

A fourth reaction was performed using 35 mL of $Et_2O$ for extraction. The product was rapidly concentrated down to an oil and then hexanes were later added to obtain a solid yellow product. The yield of yellow solid isolated and weighed was 0.4629 g (0.5678 mmol, 47%). FT-IR (Nujol, $cm^{-1}$): 3668m-sp, 3625mw-sh, 3506m-br, 3407m-br, 3229w-br, 3192w-br, 3150w, 1707mw, 1649s-br, 1619ms-br, 1597mw-sh, 1562m, 1534m, 1483ms-sh, 1464vs-br*, 1366m, 1349mw, 1324w, 1256s-br, 1222s-sh, 1203s-br, 1144vs-br, 1098m-br, 972vw-br, 950vw, 917vvw, 890vvw, *873mw*, 844vvw-br, 807m, 800m, 772w-br, 742mw, *736m*, *664ms-sh*, 661ms $cm^{-1}$. Elem. Anal. Calcd. for $CeC_{15}H_9F_{18}O_9$: %C 22.10, %H 1.11. Found %C 21.86, %H 1.10. Rerunning the sample several months later: %C 21.60, %H 1.01, duplicate %C 21.53, %H 0.96.

**Synthesis of Na(hfac)**

To a porcelain mortar, in the fumehood, $Na_2CO_3{\cdot}H_2O$ (1.0000 g, 8.065 mmol) was added and ground lightly until powdery. Hhfac (2.3 mL, 16.5 mmol) was added and ground for 15 minutes until a powdery solid. The solid was dissolved in acetone. Insoluble inorganic material was filtered out. The product crystallized out of acetone as a white solid. The yield of white crystalline material was 46% (1.7381 g, 7.5556 mmol). $^1$H NMR ($d_6$-acetone, 298 K): 5.60 (s, hfac-H), 2.85 (s, $H_2O$) ppm. $^{19}$F NMR ($d_6$-acetone, 298 K, unreferenced): −75.56 (minor), −77.55 ppm.

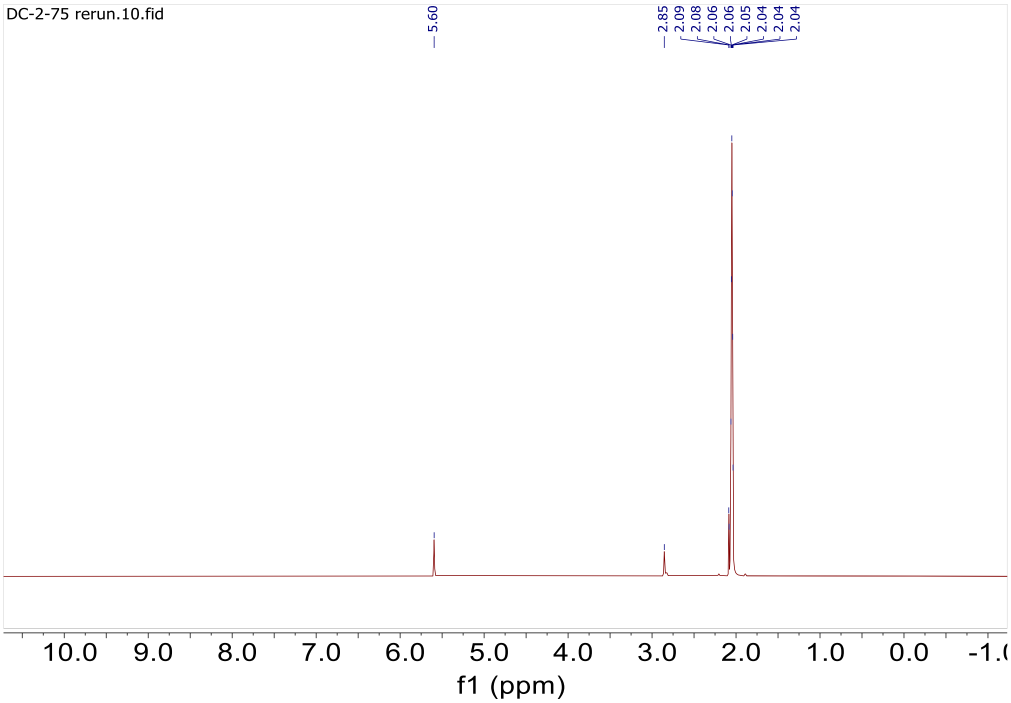


**Figure S40:** $^{1}H$ NMR Spectrum of recrystallized Na(hfac) (298 K, $d_6$-acetone, 400 MHz).

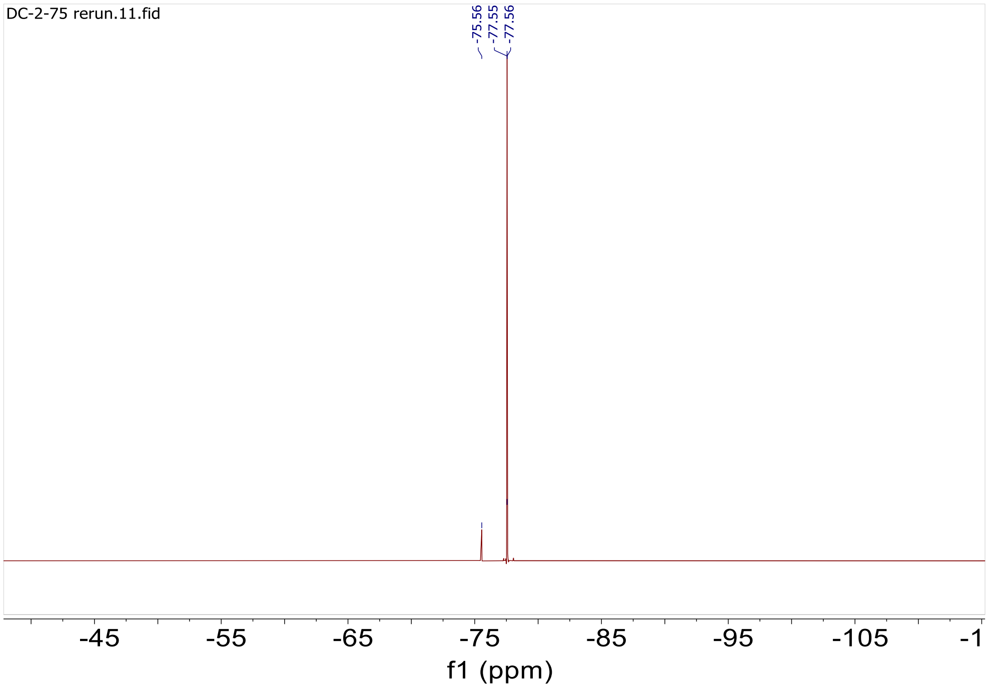


**Figure S41:** $^{19}F$ NMR Spectrum of recrystallized Na(hfac) (298 K, $d_6$-acetone, 376 MHz).

**$Pr(hfac)_3(H_2O)_3$ modified M1-SAG (1)**

A porcelain mortar was charged with recrystallized Na(hfac) (0.5025 g, 2.1845 mmol), $PrCl_3\cdot7H_2O$ (0.2723 g, 0.7285 mmol) was then added and ground for 20 minutes. The product was extracted with 4×10 mL of $Et_2O$. To the $Et_2O$ extract, 5 mL of hexanes was added to facilitate formation of a green solid (0.2898 g was extracted from 0.5 g of crude). FT-IR (Nujol, $cm^{-1}$): 3690vw-sp, 3600vw, 3495w-br, 3187vw-br, 3144vw, 1648ms, 1613w, 1564w, 1538m, 1487ms-sh, 1462s-br*, 1366w, 1352w, 1340vw, 1301w, 1255s, 1224m, 1210m, 1201m, 1154s, 1143m, 1097mw, 1033vw, 969vw, 949vw, 934vw, 918vvw, 890vw, *870w*, 846vw, *807m*, 801m, 771vw-br, 740m, *734m*, 662ms $cm^{-1}$. Elem. Anal. Calcd. for $PrC_{15}H_9F_{18}O_9$: %C 22.08, %H 1.11. Found %C 22.23, %H 1.10.

**S6a: One Pot Ball Mill Synthesis of $La(hfac)_3(H_2O)_3$ and $Sm(hfac)_3(H_2O)_2$**

**Ball milling optimization**

We tested several different conditions to try to optimize for the desired $Ln(hfac)_3(H_2O)_3$ product. Most tests were done with La because NMR could be used to investigate the products and obtain semi-quantitative data regarding the ratio of product to impurity. Parameters that were used for optimization studies include length of reaction, ball milling size, identity of base, equivalents of base and speed of milling. All large-scale ball milling reactions used *ca*. 18 g of milling material. A minimum period of 15 to 20 minutes was necessary to ensure that the first step of the reaction, which involved deprotonation of Hhfac went to completion. This was indicated by the formation of solid that finely coated the beads and the buildup of gas. Secondly, after the reaction with the $LnCl_3\cdot7H_2O$ salt, no gas should be released. The 3 mm bead size and more impactful speed of 500 rpm was associated with a relatively high yield of $La(hfac)_3(H_2O)_3$ and a reduction in the tetraol impurity. Samples with greater amounts of tetraol will require substantially more time for removal via vacuum. This is likely because of enhanced intermolecular hydrogen bonding.

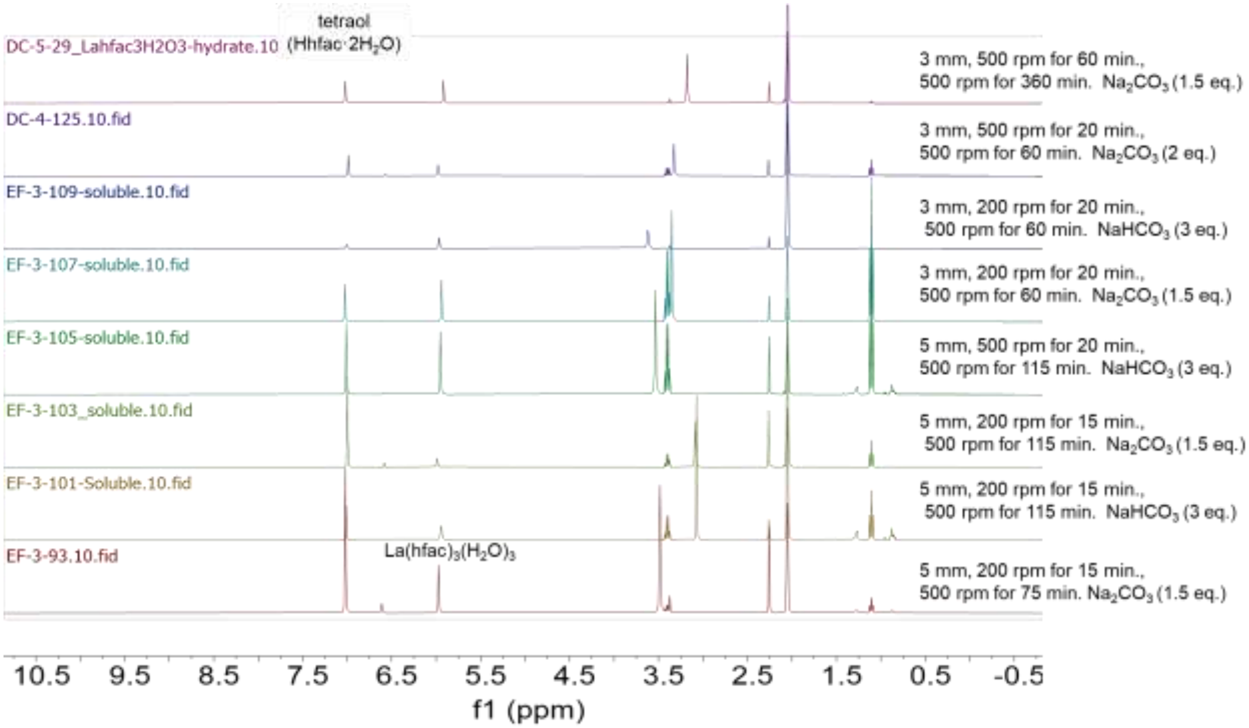


**Figure S42:** Comparison of $^{1}$H NMR spectra for optimization of ball milling for La(hfac)$_3$(H$_2$O)$_3$ (400 MHz, d$_6$-acetone, 298 K). All products are extracted using Et$_2$O. No further purification performed.

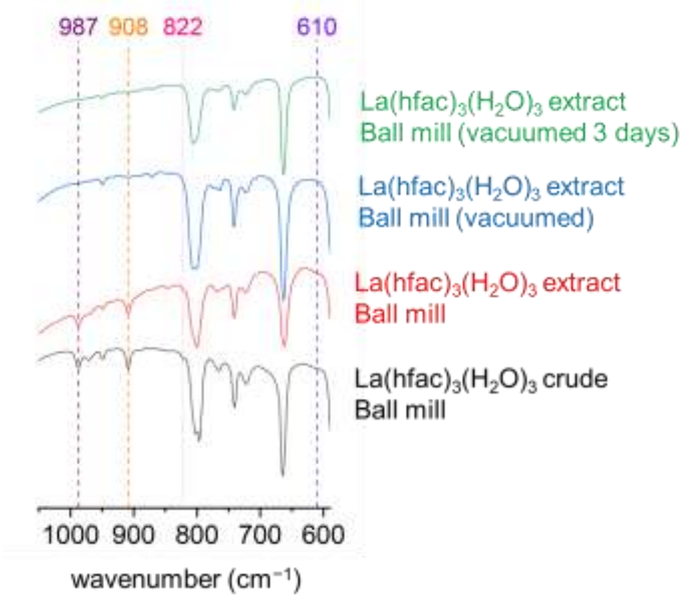


**Figure S43:** Comparison of FT-IR spectra of fingerprint region of La(hfac)$_3$(H$_2$O)$_3$ following ball milling (Nujol, 2 cm$^{-1}$ res.) (expansion of Figure 8 in main text).

**Table S9:** Summary of optimization of ball milling conditions for $La(hfac)_3(H_2O)_3$ synthesis

| Rxn ID | Bead size (mm) | Step 1 Time (min) | Step 1 speed (rpm) | Step 2 time (min) | Step 2 speed (rpm) | Based used/eq. | Ratio product to tetraol ($^1$H NMR) | Yield of $La(hfac)_3(H_2O)_3$ based on $^1$H NMR integration |
|---|---|---|---|---|---|---|---|---|
| 3-93 | 5 | 15 | 200 | 75 | 500 | $Na_2CO_3$/1.5 | 1:2.1 | 27% |
| 101 | 5 | 15 | 200 | 115 | 500 | $NaHCO_3$/3 | 1:0.65 | 48% |
| 103 | 5 | 15 | 200 | 115 | 500 | $Na_2CO_3$/1.5 | 1:3.3 | 33% |
| 105 | 5 | 20 | 500 | 115 | 500 | $Na_2CO_3$/1.5 | 1:0.79 | 41% |
| 107 | 5 | 20 | 500 | 115 | 500 | $NaHCO_3$/3 | 1:0.52 | 32% |
| 109 | 3 | 20 | 500 | 60 | 500 | $Na_2CO_3$/1.5 | 1:0.41 | 38% |
| 111 | 3 | 20 | 500 | 60 | 500 | $NaHCO_3$/3 | 1:0.46 | 54% |
| 111-vac | Placed under vacuum for 6 hours (40 Torr) | | | | | | **1:0.01** | **50%** |
| 125 | 3 | 20 | 500 | 60 | 500 | $Na_2CO_3$/2 | 1:0.84 | 55% |
| 5-29 | 3 | 60 | 500 | 360 | 500 | $Na_2CO_3$/1.5 | 1:0.76 | 67% |

**Ball milling Reactions:**

To a 50 mL YSZ container, $NaHCO_3$ (0.5241 g, 6.238 mmol) and Hhfac (0.9 mL, 6(4) mmol) was added and ball milled with 18.2 g of 3 mm YSZ-balls for 20 min at 500 rpm. Caution must be taken when opening the container as there is a build-up of $CO_2$. $LaCl_3 \cdot 7H_2O$ (0.7990 g, 2.151 mmol) was added and ball milled for one hour at 500 rpm. Analysis of the crude reaction was completed. $^1$H NMR crude (400 MHz, $d_6$-acetone): 5.91 (3H, s), 3.56 (7.4 H, s) ppm. Minor resonances are observed at 7.04 (1.8H, s), 2.25 (1H, s) ppm. $^{19}$F NMR (376 MHz, $d_6$-acetone): −77.5, −87.8 (minor) ppm.

The white solid was extracted with 25mL of $Et_2O$ and concentrated to an oil. To facilitate solid formation, 3 mL of hexanes was added. This reaction yielded 1.1563 g of white solid. FT-IR (Nujol, $cm^{-1}$): 3688mw, 3650mw, 3601mw, 3523m-br, 3391mw-sh, 3334mw-sh, 3142w, 1651vs-br, 1613m, 1605m, 1562ms, 1559ms, 1537ms, 1534ms, 1464s-br*, 1367m, 1348mw, 1324mw, 1256vs-br, 1207s-br, 1145vs-br, 1094ms, *987mw*, 973w-sh, 949w, 934vw, *909mw-br*, *872vw*, 846vw-br, 801s, 767w-br, 741m, 662s $cm^{-1}$. $^1$H NMR (400 MHz, $d_6$-acetone): 5.94 (s, 3H, hfac), 3.35 (s, 6.3H) ($La(hfac)_3(H_2O)_3$), 7.03 (s, 1.85H, tetraol), 2.26 (s, 1.03H, tetraol), 6.61 (s, Hhfac,

0.23H), 3.41 (q, 8 Hz, $Et_2O$), 1.11 (t, 6.8 Hz, 1.45H, $Et_2O$) ppm. $^{19}$F NMR (376 MHz, $d_6$-acetone): −77.5, −87.8 ppm.

After vacuuming the sample for 6 hours, the NMR analysis was repeated. $^1$H NMR crude (400 MHz, $d_6$-acetone): 5.94 (3H, s), 3.65 (6.4 H, s) ppm. Minor resonances are observed at 7.04 (0.02H, s), 2.25 (0.01H, s) ppm. $^{19}$F NMR (376 MHz, $d_6$-acetone): −77.0 (minor), −77.5, −78.0 (minor), −87.8 (minor, tetraol) ppm. FT-IR (Nujol, $cm^{-1}$): 3687mw-sp, 3638w-sp, 3581mw, 3522m-br, 3309w-sh, 3220w-sh, 3144w, 1652vs-br, 1614mw, 1561m, 1534m, 1479s, 1469s-br*, 1366w, 1346w, 1323w, 1256vs-br, 1214vs-br, 1147vs-br, 1101m, *987vw*, 968vw, 949w, 934vw, *909vw*-br, 890vvw, *871w*, 844vvw-br, 802s-br, 773w-br, 765w, 741mw, 663s $cm^{-1}$. Elem. Anal. Calcd. for $LaC_{15}H_9F_{18}O_9$: %C 22.13, %H 1.11. Found %C 22.25, %H 1.05.

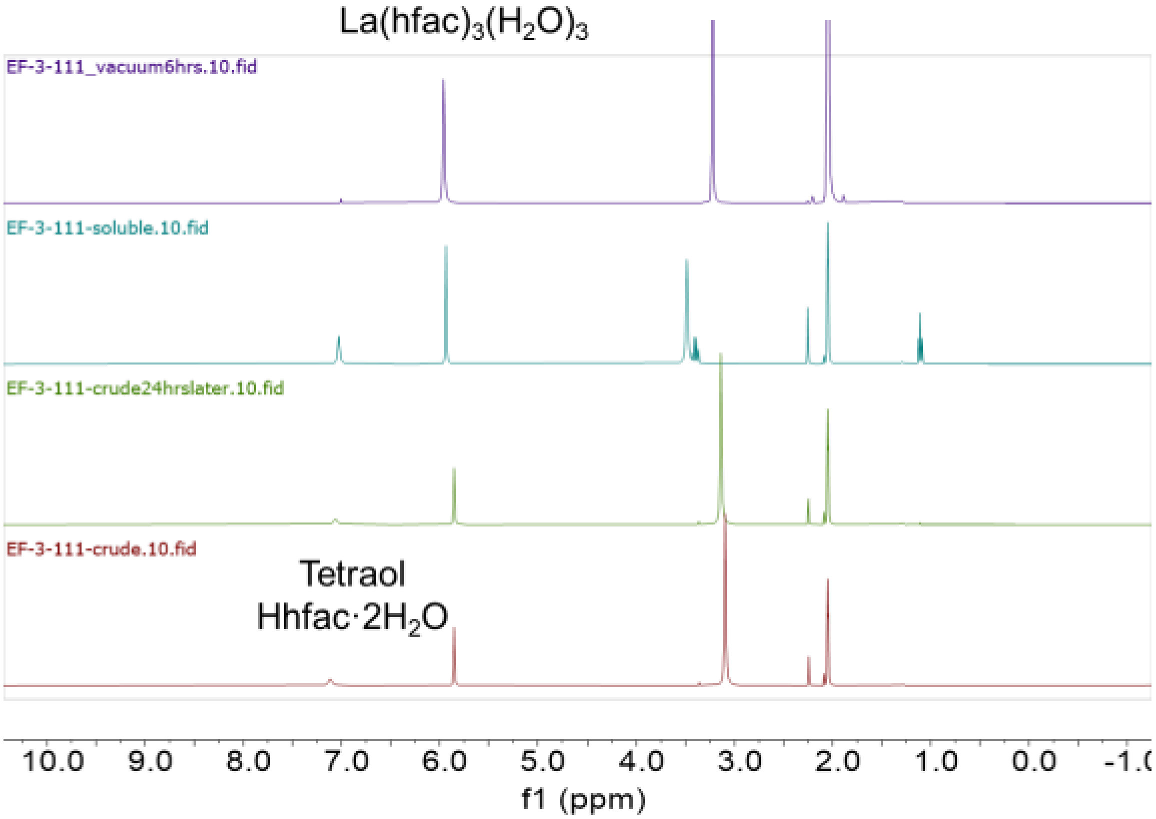


**Figure S44:** Comparison of $^1$H NMR spectra of crude (bottom), crude after 24 hours (2$^{nd}$ from bottom), extracted product (second from top) and extracted product vacuumed for 6 hours (top) (400 MHz, $d_6$-acetone, 298 K).

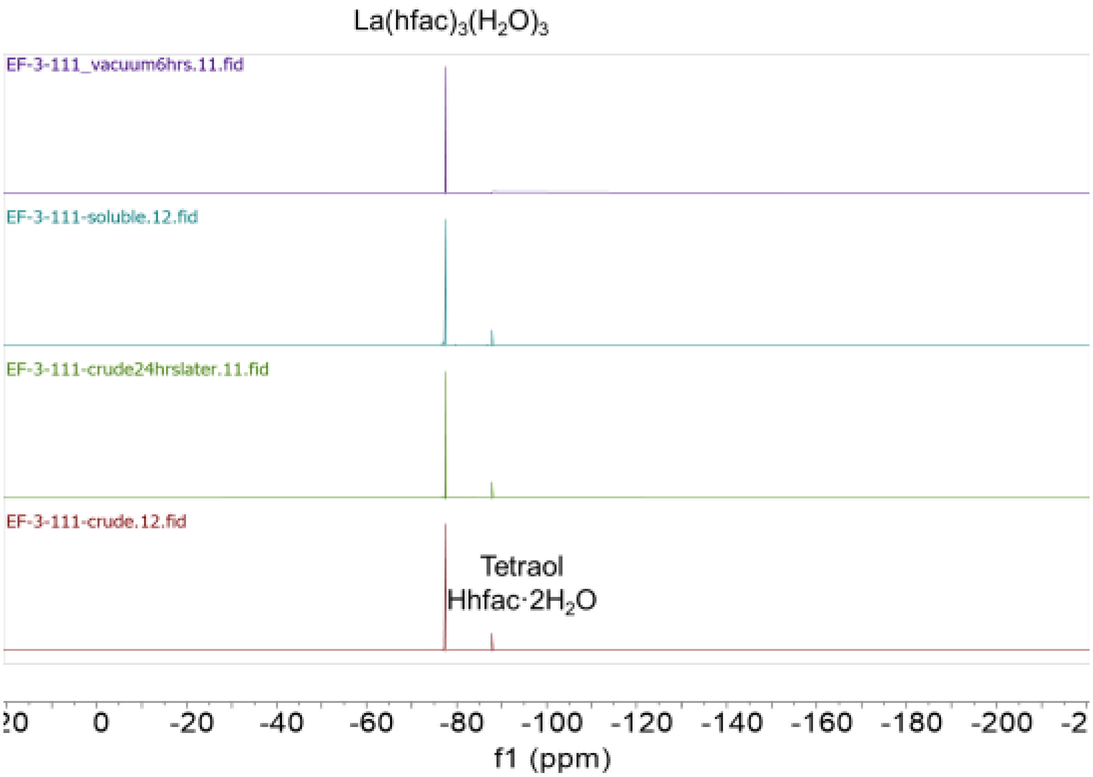


**Figure S45:** Comparison of $^{19}$F NMR spectra of crude (bottom), crude after 24 hours (2$^{nd}$ from bottom), extracted product (second from top) and extracted product vacuumed for 6 hours (top) (400 MHz, $d_6$-acetone, 298 K). (376 MHz, $d_6$-acetone, 298 K).

**Less Optimized Reactions of $Na_2CO_3 \cdot H_2O$, Hhfac and $SmCl_3 \cdot 7H_2O$**

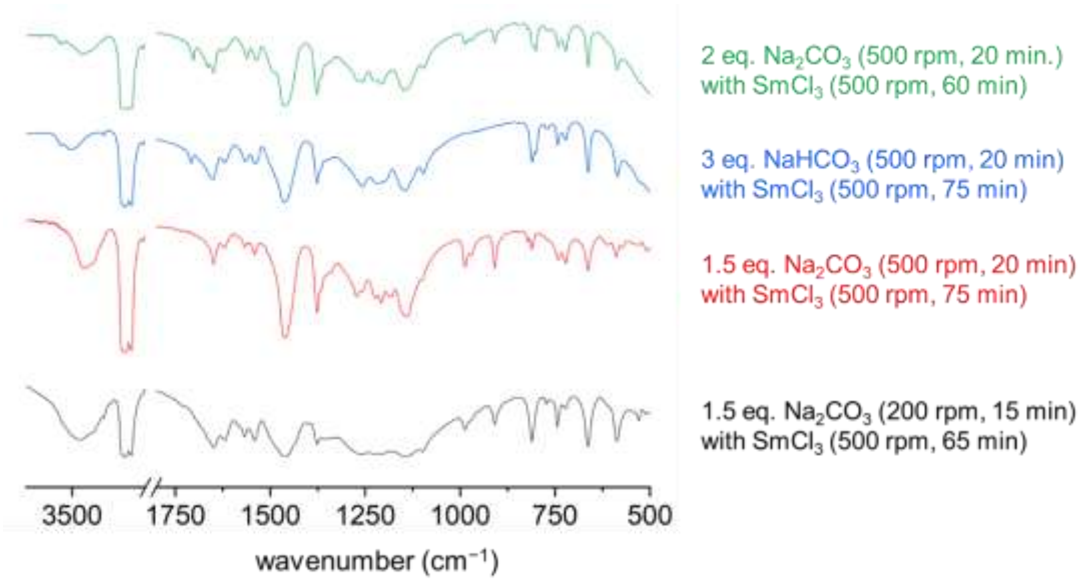


**Figure S46**: Comparison of IR spectra of $Sm(hfac)_3(H_2O)_2$ extracted from ball milling reactions.

**S6b: Ball Mill Synthesis using $LnCl_3 \cdot 7H_2O$ and Purified Na(hfac)**

**Synthesis of Na(hfac) using mortar and pestle for ball mill reactions:**

To a porcelain mortar, in the fumehood, $Na_2CO_3 \cdot H_2O$ (1.0001 g, 8.065 mmol) was added and ground lightly until powdery. Hhfac (2.25 mL, 16.1 mmol) was added and ground for 15 minutes until a powdery solid. The solid was dissolved in acetone. Insoluble inorganic material was filtered out. The product crystallized out of acetone as a white solid. The yield of white crystalline material was 53% (1.9622 g, 8.5298 mmol). $^1$H NMR ($d_6$-acetone, 298 K): 5.59 (s, hfac-H), 3.08 (s, $H_2O$) ppm. $^{19}$F NMR ($d_6$-acetone, 298 K, unreferenced): −75.82 (minor), −77.53 ppm. $^{13}$C NMR ($d_6$-acetone, 298 K): 175.0 (q, $^2J_{C-F}$=30 Hz, hfac), 119.0 (q, $^1J_{C-F}$=288 Hz, hfac), 85.1 (hfac), 161.8 (q, $^2J_{C-F}$=33 Hz, tfa), 118.0 (q, $^1J_{C-F}$=293 Hz, tfa) ppm.

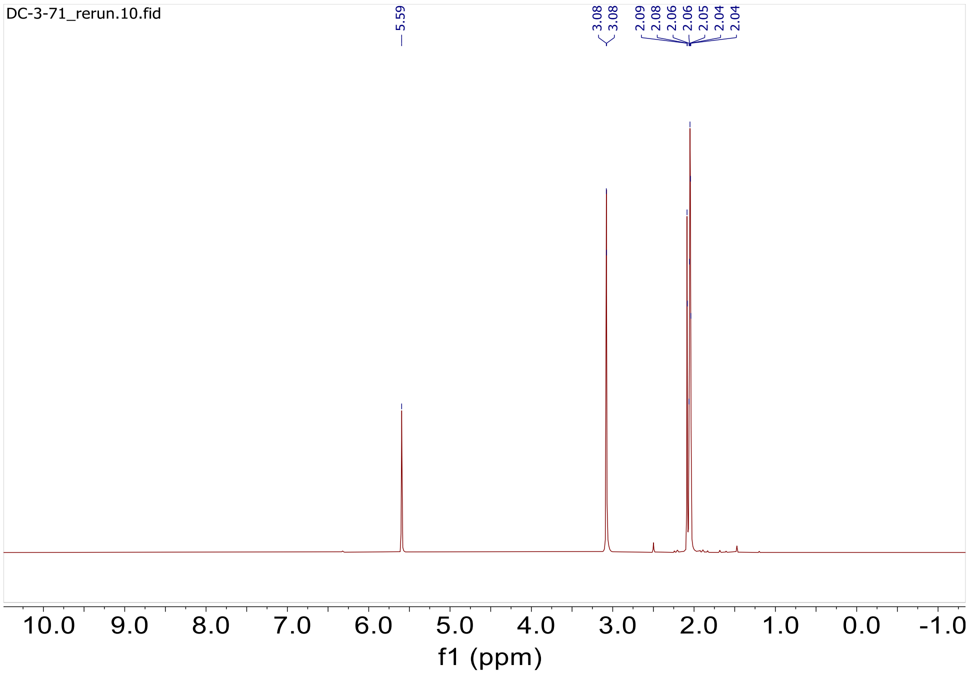


**Figure S47**: $^1$H NMR of recrystallized Na(hfac) prepared using mortar and pestle (400 MHz, $d_6$-acetone, 298 K).

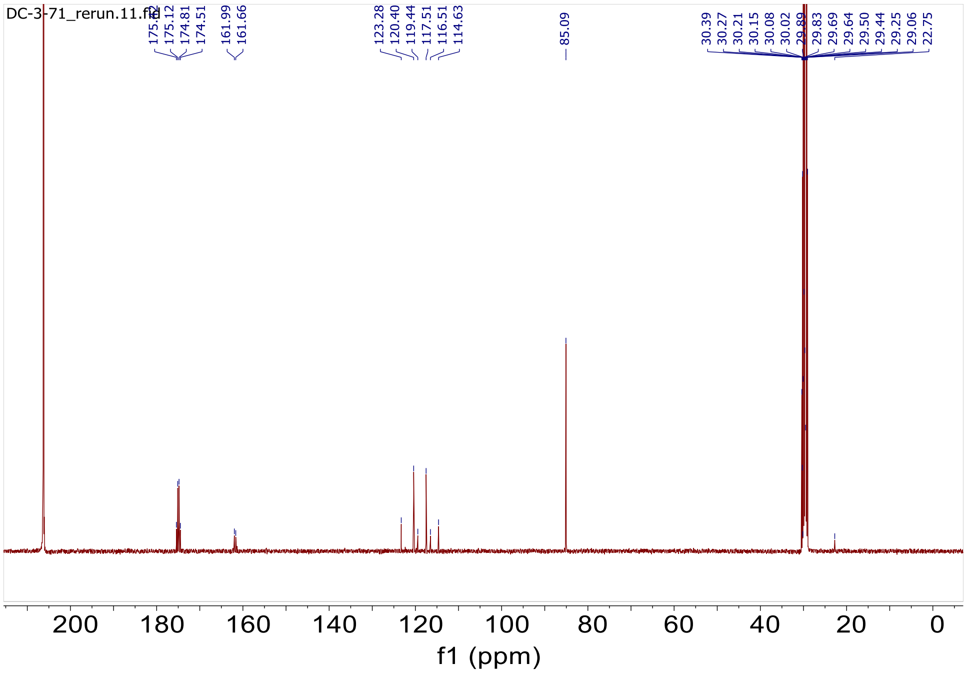


**Figure S48**: $^{13}$C NMR of recrystallized Na(hfac) prepared using mortar and pestle. (100 MHz, $d_6$-acetone, 298 K).

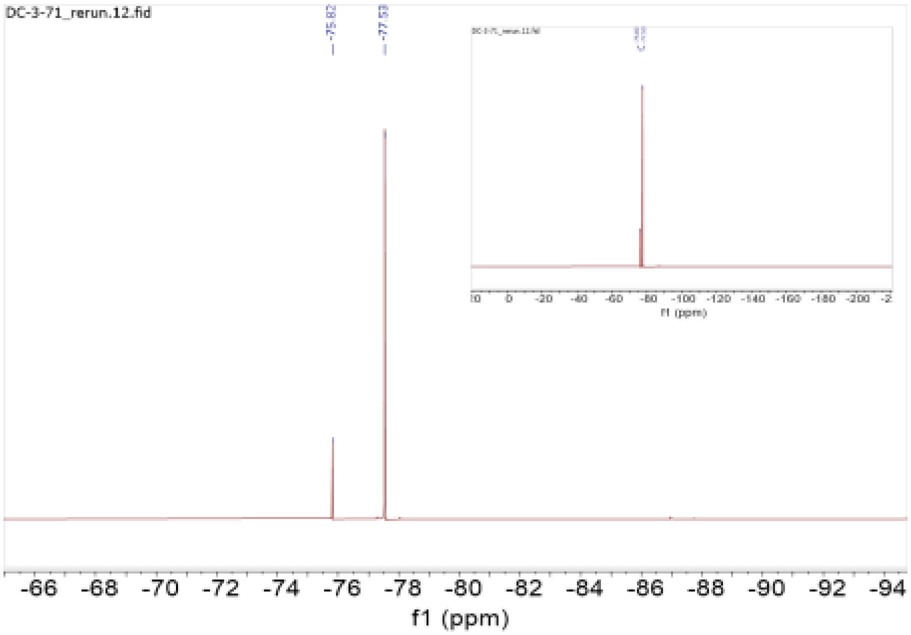


**Figure S49**: $^{19}$F NMR of recrystallized Na(hfac) prepared using mortar and pestle (376 MHz, $d_6$-acetone, 298 K).

In a 2 mL plastic container charged with 0.5 mL of 3 mm YSZ grinding media, Na(hfac) (0.1952 g, 0.8486 mmol) and $LaCl_3{\cdot}7H_2O$ (0.1055 g, 0.2841 mmol) were combined. The reaction was ball milled for 180 sec at 2800 rpm. The wet product was removed and extracted with 7 mL of $Et_2O$. The yield of the solid product after slow evaporation was 79% (0.1823 g, 0.2239 mmol). FT-IR (Nujol, $cm^{-1}$): 3679w-sh, 3609w-sh, 3503mw-br, 3194w, 3150w, 1648m-br, 1567mw, 1559w-sh, 1541mw, 1466s*-br, 1366mw, 1350w, 1338w-sh, 1258ms-br, 1234ms, 1210ms, 1195ms, 1145s-br, 1094m, 970vw, 949vw, 935vw, 918vw, 890vw, *871mw*, 846vw, 808m, 802m, 772vw, 740mw, *734mw*, 662m $cm^{-1}$. Elem. Anal. Calcd. for $LaC_{15}H_9F_{18}O_9$: %C 22.13, %H 1.11. Elem. Anal. Calcd. for retro-Claisen: %C 20.53, %H 0.86. Found %C 21.69, %H 0.99, duplicate %C 21.57, %H 0.90.

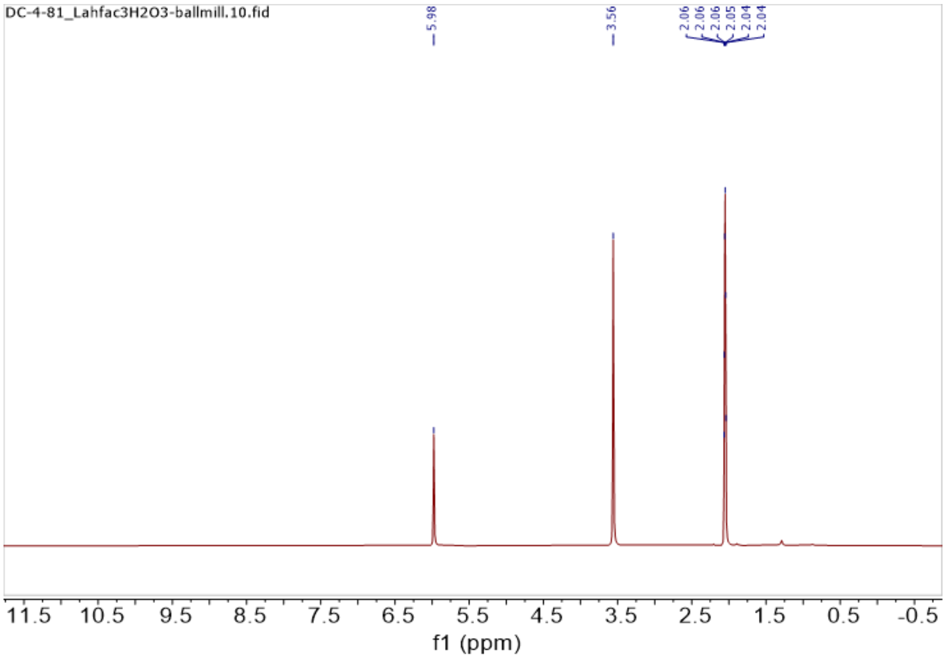


**Figure S50**: $^1$H NMR of ball mill reaction of $LaCl_3{\cdot}7H_2O$ with 3 eq. recrystallized Na(hfac). (400 MHz, $d_6$-acetone, 298 K).

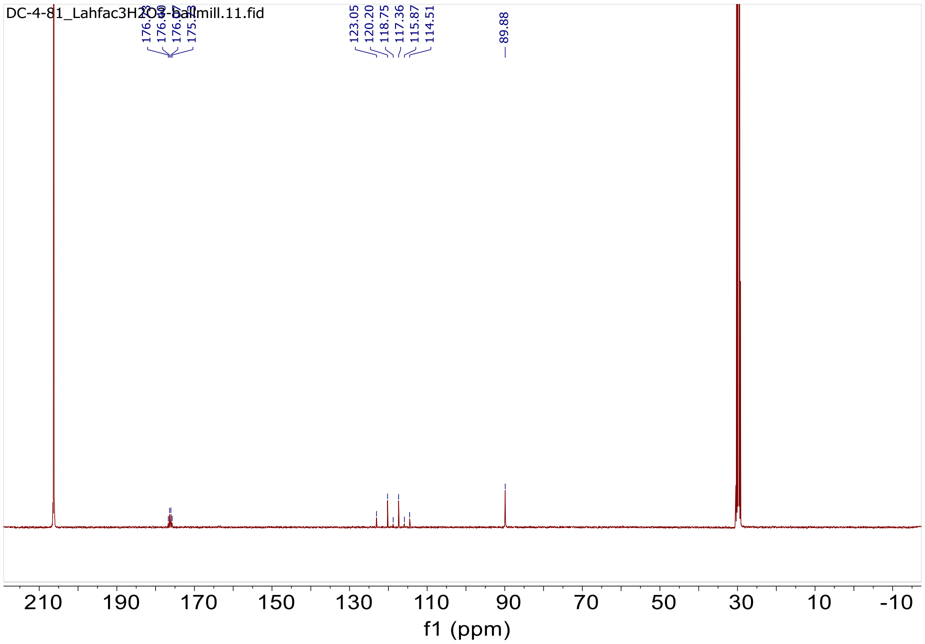


**Figure S51**: $^{13}$C NMR of ball mill reaction of $LaCl_3 \cdot 7H_2O$ with 3 eq. recrystallized Na(hfac). (100 MHz, $d_6$-acetone, 298 K).

In a 2 mL plastic container charged with 0.5 mL of 3 mm YSZ grinding media, Na(hfac) (0.2020 g, 0.8782 mmol) and $SmCl_3 \cdot 7H_2O$ (0.1119 g, 0.2923 mmol) were combined. The reaction was ball milled for 180 sec at 2800 rpm. The wet product was removed and extracted with 7 mL of $Et_2O$. The yield of the solid product after slow evaporation was 75% (0.1782 g, 0.2207 mmol). FT-IR (Nujol, cm$^{-1}$): 3608mw-sh, 3503mw-br, 3191w, 3153w, 1661m-sh, 1649ms-br, 1625m-sh, 1569mw, 1542mw, 1487m-sh, 1461s*-br, 1366m, 1353w, 1339mw, 1257s-br, 1209ms-br, 1172ms, 1143s-br, 1096ms, 973vw, 949vw, 918vw, 889vw, *869mw*, 811m, 802mw-sh, 773w, 743mw, *733m*, 662m cm$^{-1}$. Elem. Anal. Calcd. for $SmC_{15}H_7F_{18}O_8$: %C 22.31, %H 0.87. Elem. Anal. Calcd. for retro-Claisen: %C 20.20, %H 0.85. Found %C 21.47, %H 0.98, duplicate %C 21.38, %H 0.86.

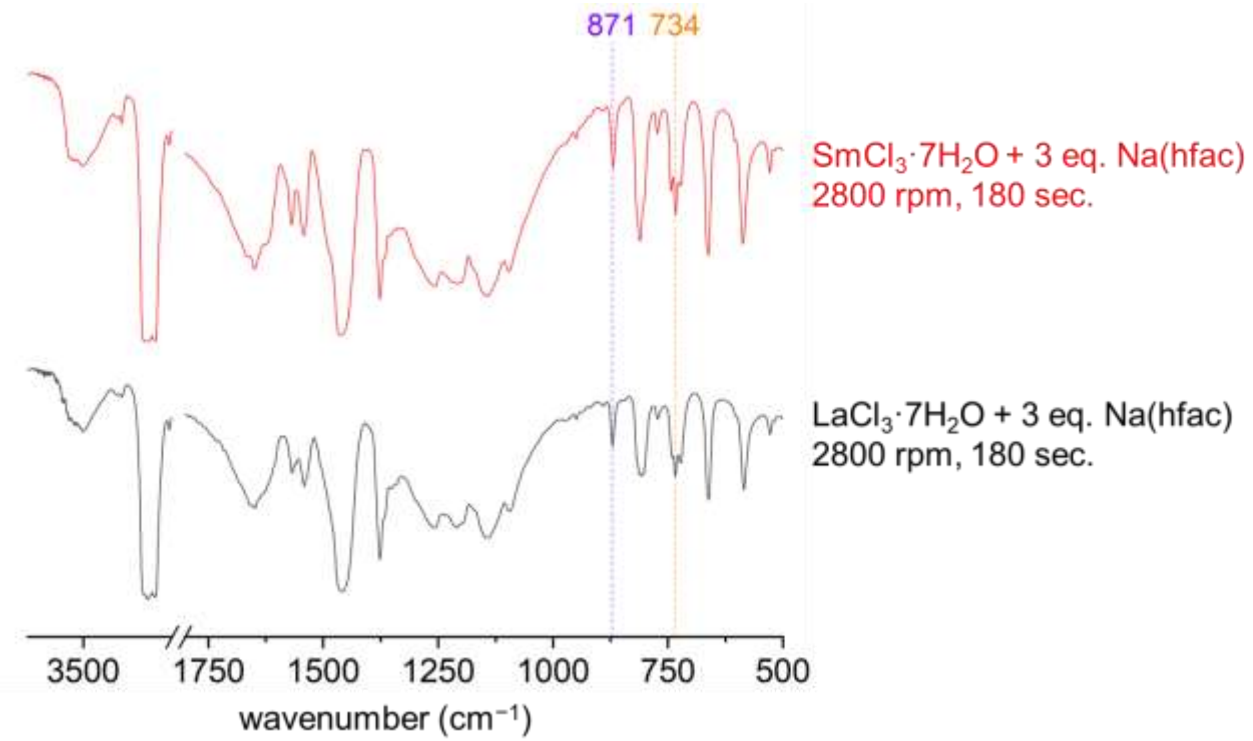


**Figure S52**: FT-IR spectra of $LaCl_3 \cdot 7H_2O$ with 3 eq. purified Na(hfac) (bottom) and $SmCl_3 \cdot 7H_2O$ with 3 eq. purified Na(hfac) (top) (KBr, Nujol, 2cm$^{-1}$). Dotted lines indicate presence of retro-Claisen impurity.

**Preparation of Na(hfac) using a ball mill**

To a 50 mL YSZ container, $Na_2CO_3 \cdot H_2O$ (0.5485 g, 4.423 mmol) and Hhfac (1.3 mL, 9.3 mmol) was added and ball milled with 18.4 g of 3 mm YSZ-balls for 35 min at 500 rpm. *Caution must be taken when opening the container as there is a build-up of $CO_2$.* Analysis of the crude reaction was completed. $^1$H NMR crude (400 MHz, $d_6$-acetone): 7.03 (1.49H, s, tetraol), 6.60 (0.21H, s, Hhfac-enol), 5.60 (1H, s, Na(hfac)), 3.37 (0.23H, s), 3.12 (0.50H, s-br), 2.26 (0.80H, s, tetraol) ppm. $^{19}$F NMR (376 MHz, $d_6$-acetone, unreferenced): −77.5 (major), −79.8 (minor), −86.7 (minor), −87.8 ppm.

An aliquot of this material was then extracted with 10 mL of $Et_2O$. FT-IR (KBr, Nujol): 3690mw-sp, 3515mw-sh, 3390m-br, 3337mw-sh, 3264mw-sh, 3135w-sh, 1673s, 1660s-sh, 1619w, 1557s, 1532s, 1511s-sh, 1494vs, 1470s, 1366w, 1335mw, 1317w, 1261s, 1222s-sh, 1203s, 1191vs, 1155vs-sh, 1141vs, 1083m, *987mw*, 973w-sh, 947vw, *909w*, 845vw, 822vw, 798m, 769w, 761w, 740mw, 665ms cm$^{-1}$. The white solid was then placed under vacuum for two hours and re-analyzed

by NMR. $^{1}$H NMR (400 MHz, $d_6$-acetone): 5.62 (1H, s), 3.83 (0.52H, s), 3.37 (0.05H, s-br), 2.26 (0.05H, s, tetraol) ppm. $^{19}$F NMR (376 MHz, $d_6$-acetone, unreferenced): −76.9 (minor), −77.5 (major), −87.8 (minor) ppm. $^{13}$C NMR ($d_6$-acetone, 298 K): 175.1 (q, $^{2}J_{C\text{-}F}$=30 Hz, hfac), 119.1 (q, $^{1}J_{C\text{-}F}$=288 Hz, hfac), 85.3 (hfac) ppm. Small peaks were observed that are associated with small remnants of tetraol and related species at 34.1 and 42.1 ppm. FT-IR (KBr, Nujol): 3671vw-br, 3396w-sh, 3346vw-br, 3302w-br, 3264w-sh, 3182vw-sh, 3137w, 1674m, 1651m, 1613w, 1553mw, 1531ms, 1496s, 1464ms, 1365w, 1343w, 1329w, 1317w, 1257s, 1217s, 1197ms, 1146vs-sh, 1139vs, 1099m-sh, 1079m, 1047w-sh, *988w*, 972vw-br, 953vw, 946w, *909w*, 844vw, 834vw, 804m, 793m, 775w, 758w, 739w, 665ms cm$^{-1}$.

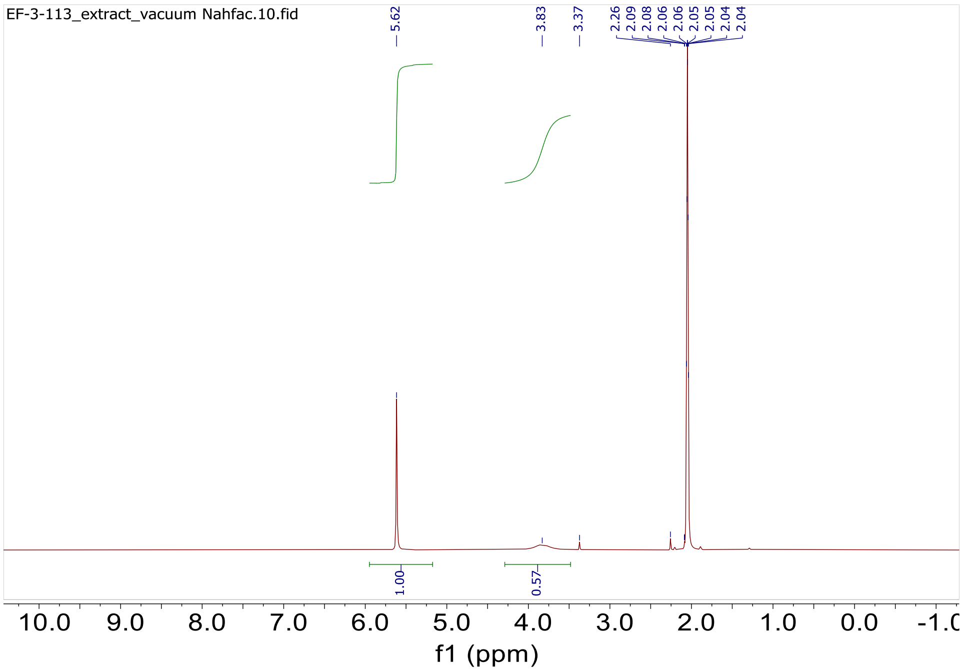


**Figure S53:** $^{1}$H NMR of recrystallized Na(hfac) prepared using ball milling and after vacuum (400 MHz, $d_6$-acetone, 298 K).

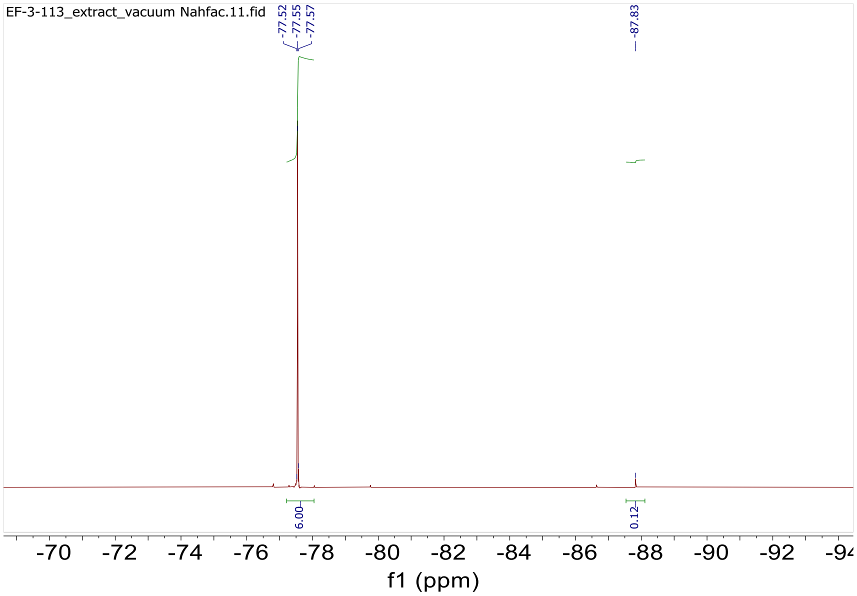


**Figure S54:** $^{19}F$ NMR of recrystallized Na(hfac) prepared using ball milling and after vacuum (376 MHz, $d_6$-acetone, 298 K).

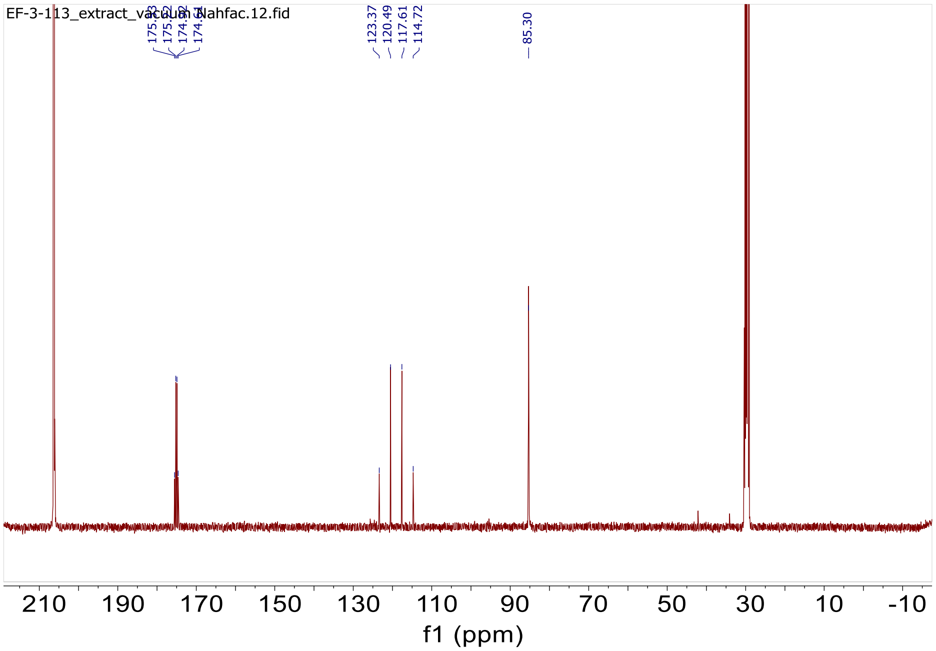


**Figure S55:** $^{13}C$ NMR of recrystallized Na(hfac) prepared using ball milling and after vacuum (100 MHz, $d_6$-acetone, 298 K).

In a 2 mL plastic container charged with 0.5 mL of 1.4-1.7 mm YSZ grinding media, Na(hfac) (0.1465 g, 0.6368 mmol) and $LaCl_3 \cdot 7H_2O$ (0.0816 g, 0.220 mmol) were combined. The reaction

was ball milled for 180 sec at 2800 rpm. The wet product was removed and extracted with 7 mL of $Et_2O$. The yield of the solid product after slow evaporation was 45% (0.0802 g, 0.0985 mmol). FT-IR (Nujol, $cm^{-1}$): 3689w-sp, 3524mw-br, 3143w, 1650m, 1614w-sh, 1561m, 1534mw, 1479s-br, 1466s*, 1365w, 1346w, 1322w, 1255ms-br, 1214ms-br, 1147s-br, 1100m, 987vw, 970vvw, 949vw, 909vw, 893vvw, *871vw*, 843vvw, 804mw-br, 771vw, 765vw, 741mw, *735vw-sh*, 663m $cm^{-1}$. Regrinding the sample led to the disappearance of peaks at 987, 909 and the appearance of a very weak broad peak at 915 $cm^{-1}$. $^1H$ NMR crude (400 MHz, $d_6$-acetone): 5.95 (3H, s), 3.30 (7.6H, s, $H_2O$) ppm. A small amount of $Et_2O$ is present. $^{19}F$ NMR (376 MHz, $d_6$-acetone): −76.9, −77.5 (major) ppm. $^{13}C$ NMR ($d_6$-acetone, 298 K): 176.2 (q, $^2J_{C\text{-}F}$=30 Hz, hfac), 118.8 (q, $^1J_{C\text{-}F}$=288 Hz, hfac), 89.8 (hfac) ppm. Some $Et_2O$ observed in $^1H$ and $^{13}C$ NMR spectra (**Figures S56 and S58**).

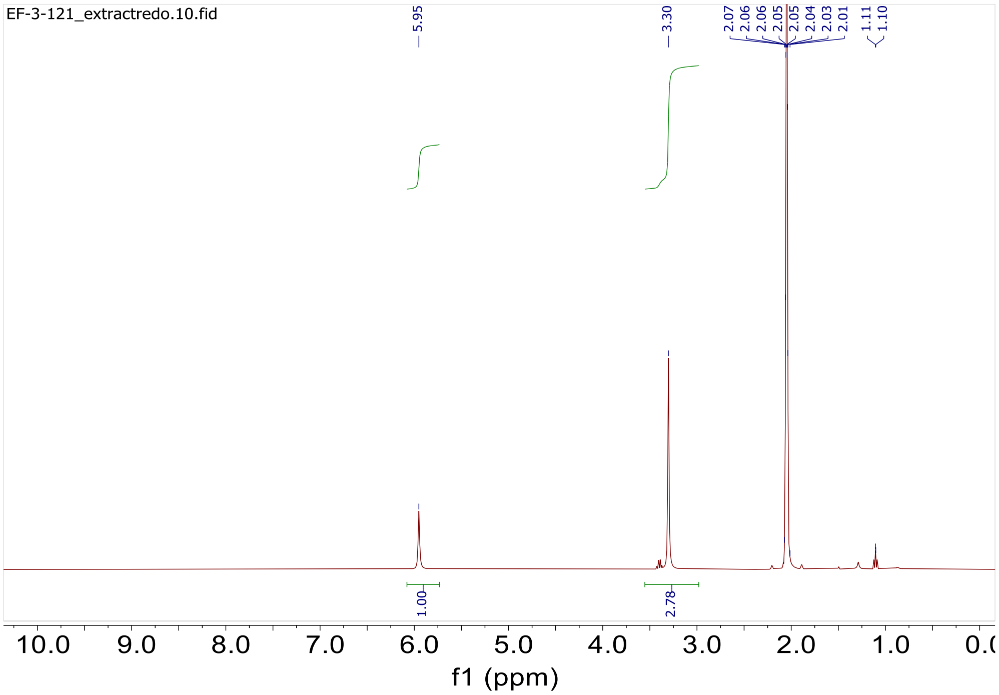


**Figure S56**: $^1H$ NMR of ball mill reaction of $LaCl_3{\cdot}7H_2O$ with 3 eq. ball milled Na(hfac). (400 MHz, $d_6$-acetone, 298 K).

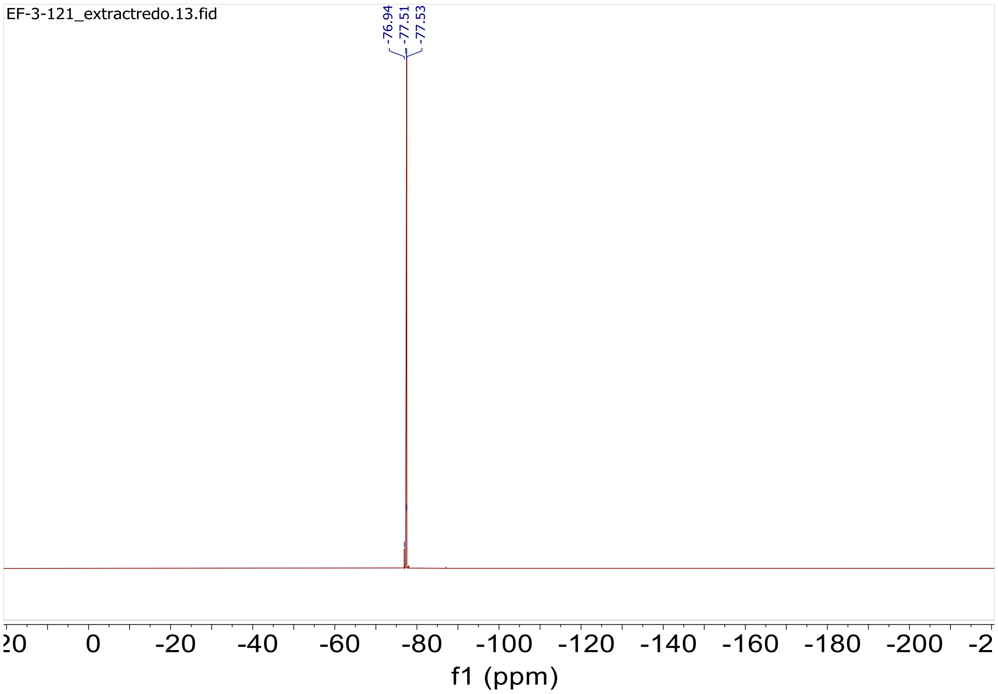


**Figure S57:** $^{19}$F NMR of ball mill reaction of $LaCl_3{\cdot}7H_2O$ with 3 eq. ball milled Na(hfac) (376 MHz, $d_6$-acetone, 298 K).

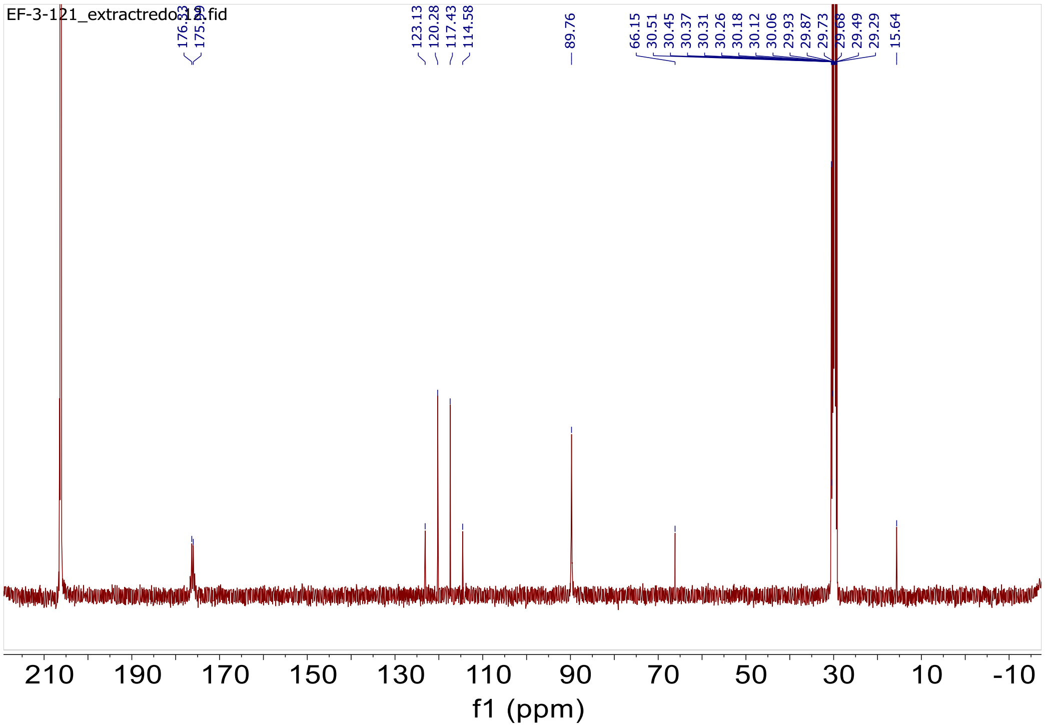


**Figure S58:** $^{13}$C NMR of ball mill reaction of $LaCl_3{\cdot}7H_2O$ with 3 eq. ball milled Na(hfac) (100 MHz, $d_6$-acetone, 298 K).

## S7. FT-IR spectra of $Ln(hfac)_3(H_2O)_x$ complexes: comparison between different methods

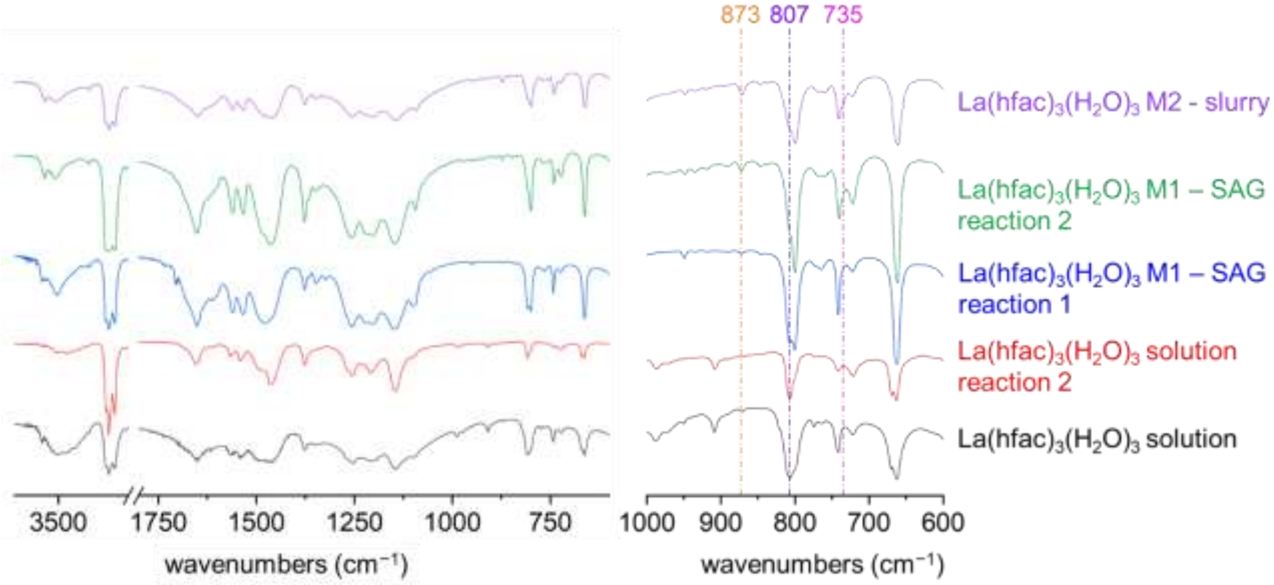


**Figure S59**: Comparison of FT-IR spectra for different $La(hfac)_3(H_2O)_3$ samples (Nujol, first and third spectra: 64 scans, 1 $cm^{-1}$ res., other spectra: 32 scans, 2 $cm^{-1}$ res.). **Note**: solution samples contain tetraol impurity.

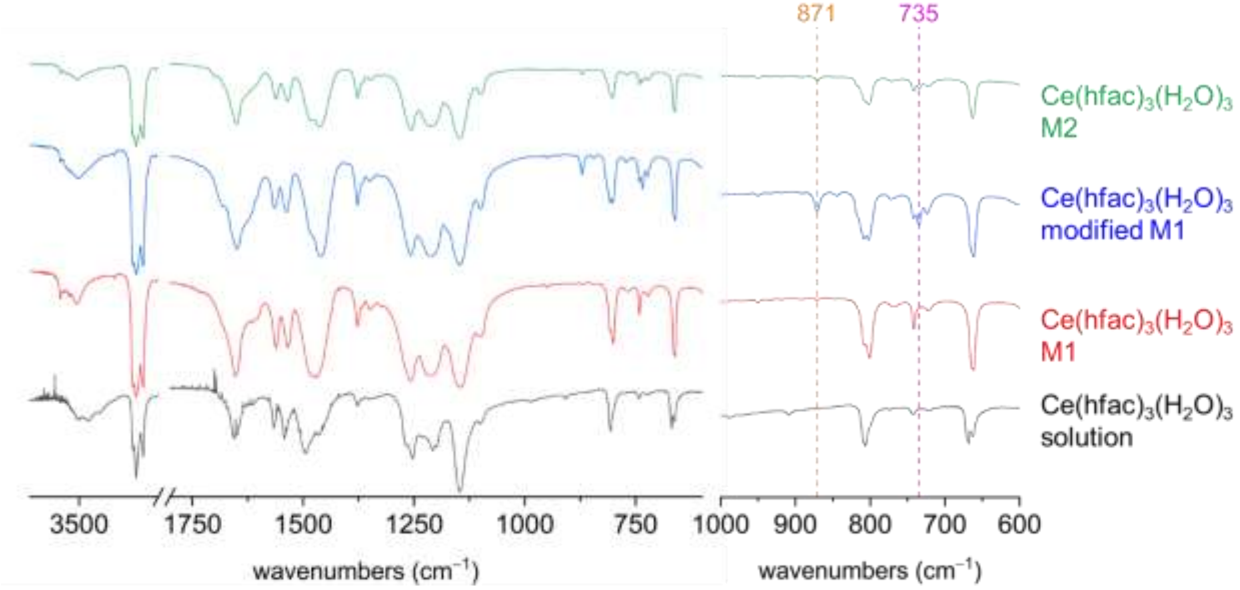


**Figure S60:** Comparison of FT-IR Spectra for different syntheses of $Ce(hfac)_3(H_2O)_3$ (Nujol, bottom two spectra: 64 scans, 1 $cm^{-1}$ res., top two spectra: 32 scans, 2 $cm^{-1}$ res.).

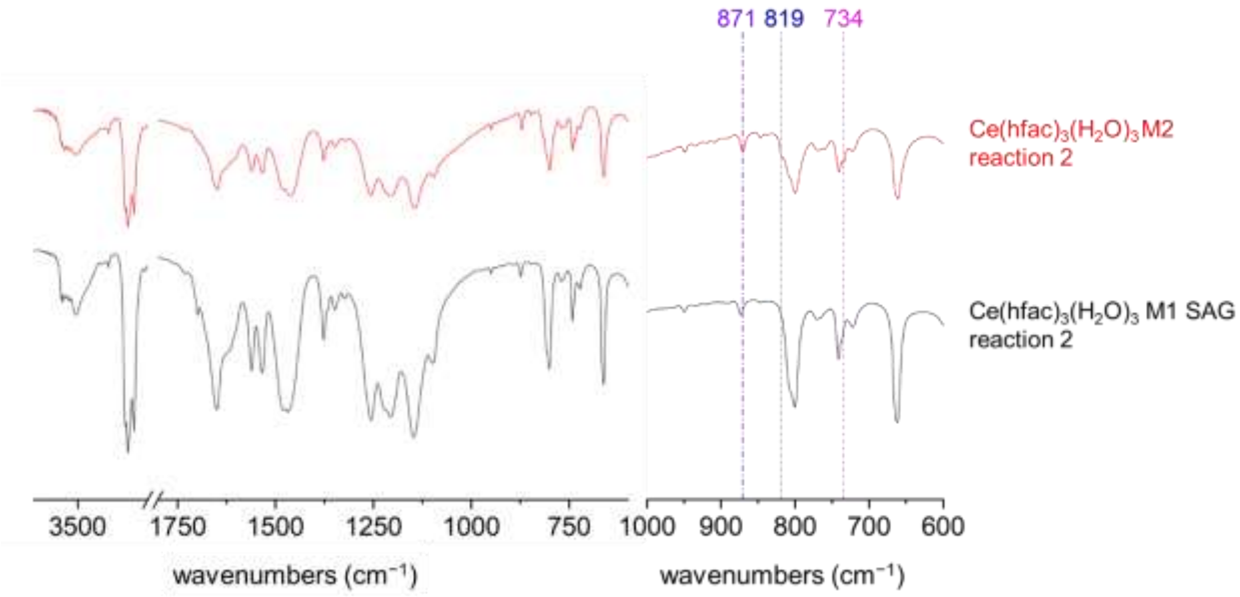


**Figure 61:** Comparison of FT-IR Spectra for repeat reactions of Ce(hfac)$_3$(H$_2$O)$_3$ M1 SAG and M2 slurry (Nujol, 32 scans, 2 cm$^{-1}$ res.).

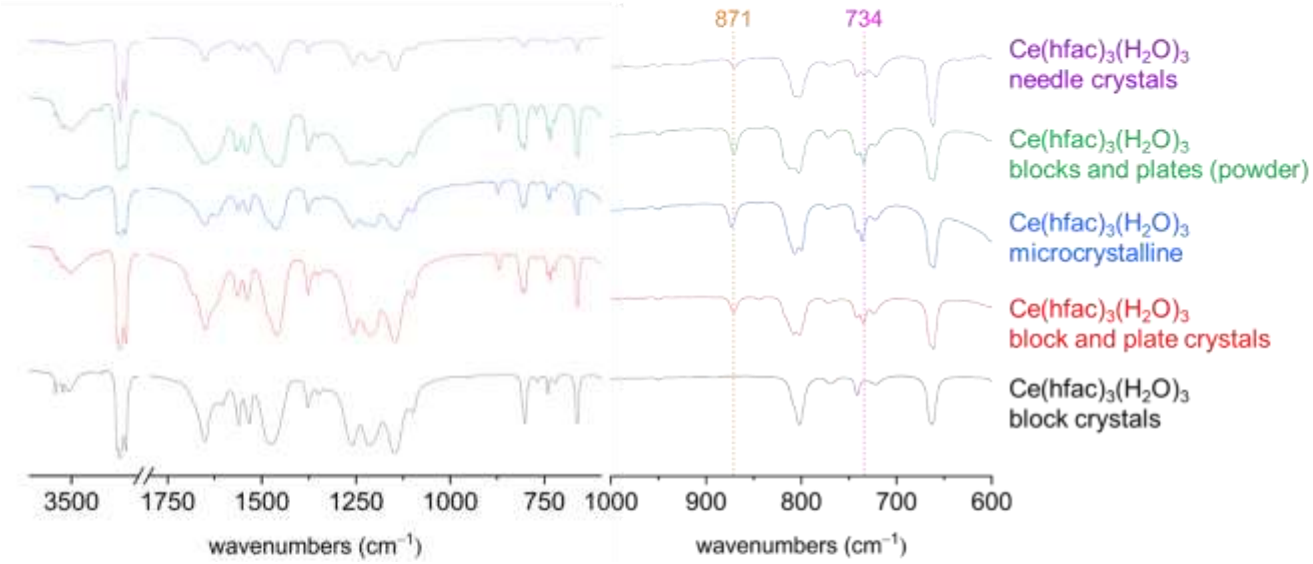


**Figure S62:** Comparison of FT-IR Spectra for products obtained from trials of modified M1 syntheses of Ce(hfac)$_3$(H$_2$O)$_3$ (Nujol, 32 scans, 2 cm$^{-1}$ res.). Block crystals are used for comparison to crystal batches in **Figure S103**. Ce(hfac)$_3$(H$_2$O)$_3$ blocks and plates (powder) IR spectrum is same as modified M1 in **Figure S60**.

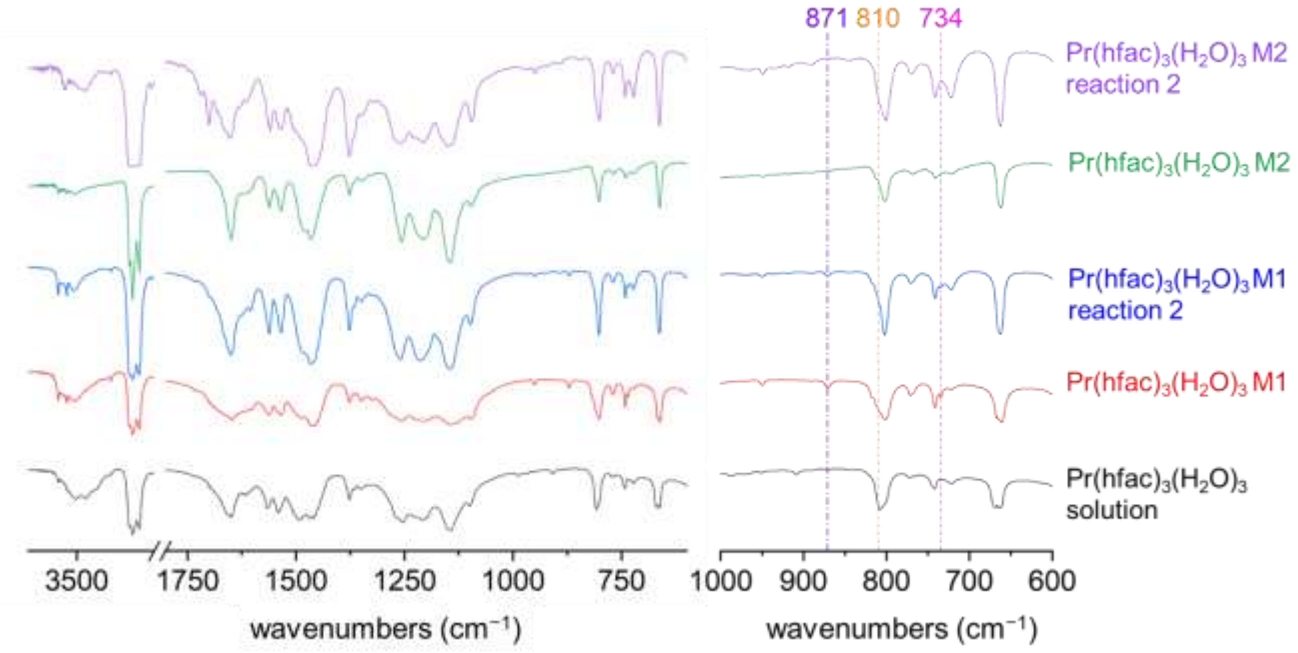


**Figure S63:** Comparison of FT-IR spectra for different syntheses of Pr(hfac)$_3$(H$_2$O)$_3$ including repeat reactions (Nujol, bottom two spectra: 64 scans, 1 cm$^{-1}$ res., top spectra: 32 scans, 2 cm$^{-1}$ res.).

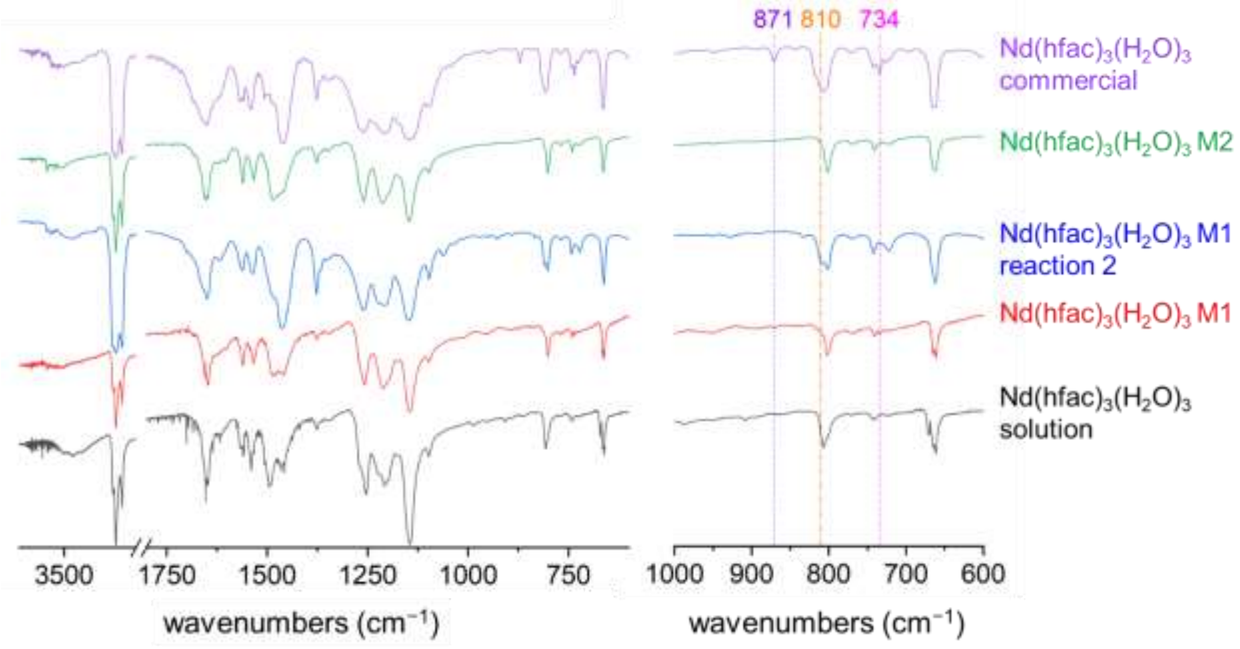


**Figure 64:** Comparison of FT-IR Spectra for different syntheses of Nd(hfac)$_3$(H$_2$O)$_3$ including repeat reactions and commercial (Nujol, solution and M1 reaction 1 spectra: 64 scans, 1 cm$^{-1}$ res., top spectra: 32 scans, 2 cm$^{-1}$ res.).

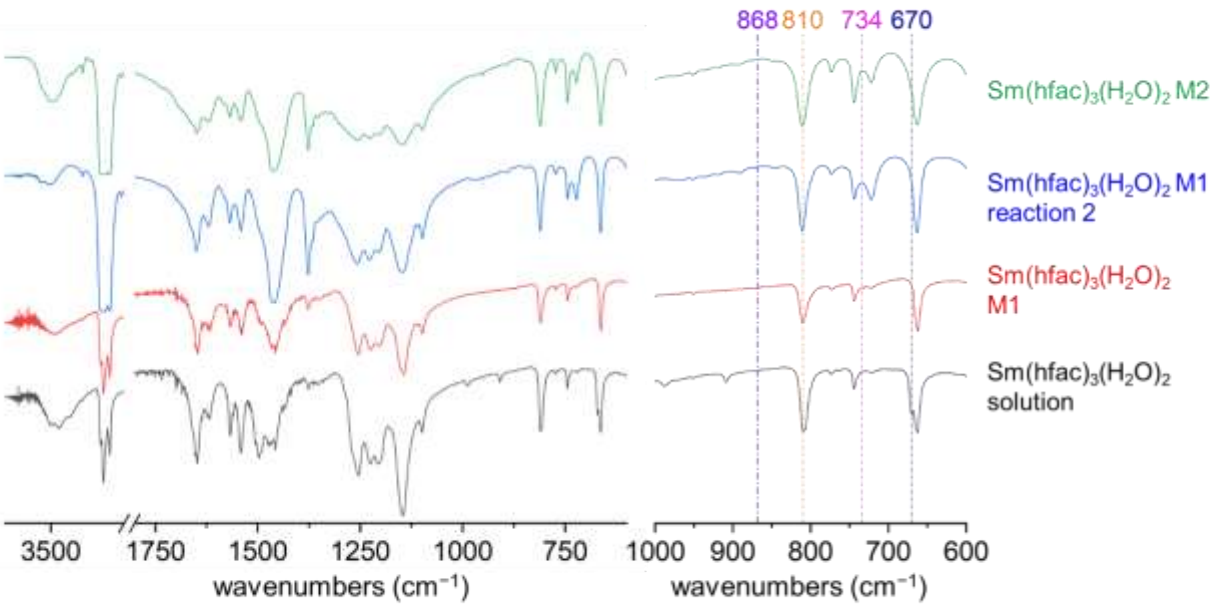


**Figure 65:** Comparison of FT-IR Spectra for different syntheses of $Sm(hfac)_3(H_2O)_2$ including repeat reaction (Nujol, bottom two spectra: 64 scans, 1 $cm^{-1}$ res., two top spectra : 32 scans, 2 $cm^{-1}$ res.).

**Notes about characterization of $Ln(hfac)_3(H_2O)_x$ Complexes and Impurities Using IR Spectroscopy and Other Techniques**

FT-IR spectroscopy is useful for characterizing the products and impurities in these reactions. The dinuclear cluster that can form from a retro-Claisen condensation has some characteristic peaks in the fingerprint region (**Figure S66a**).[1] In **Figure S66a**, the IR spectra were collected on samples from the same batch. The bottom spectrum is of block crystals of $Ce(hfac)_3(H_2O)_3$ that first formed from slow evaporation of $Et_2O$ and hexanes whereas the top spectrum containing the impurity is from material that recrystallized from the same sample batch several hours later. For the tetraol impurity, some common peaks are also observed in the fingerprint region (**Figure S66b**). These peaks are observed in some of the solution syntheses and the ball milling reactions during which Na(hfac) is prepared; however, the presence of the tetraol is negligible in the mortar and pestle M1 and M2 reactions. The removal of tetraol observed using NMR spectroscopy was corroborated by the reduction of the peaks listed in **Figures S66b** and **Figure 8**. We found that certain methods were more useful for identifying the presence of impurities that may be present even if the compound passes elemental analysis (**Tables S10 and S11**).

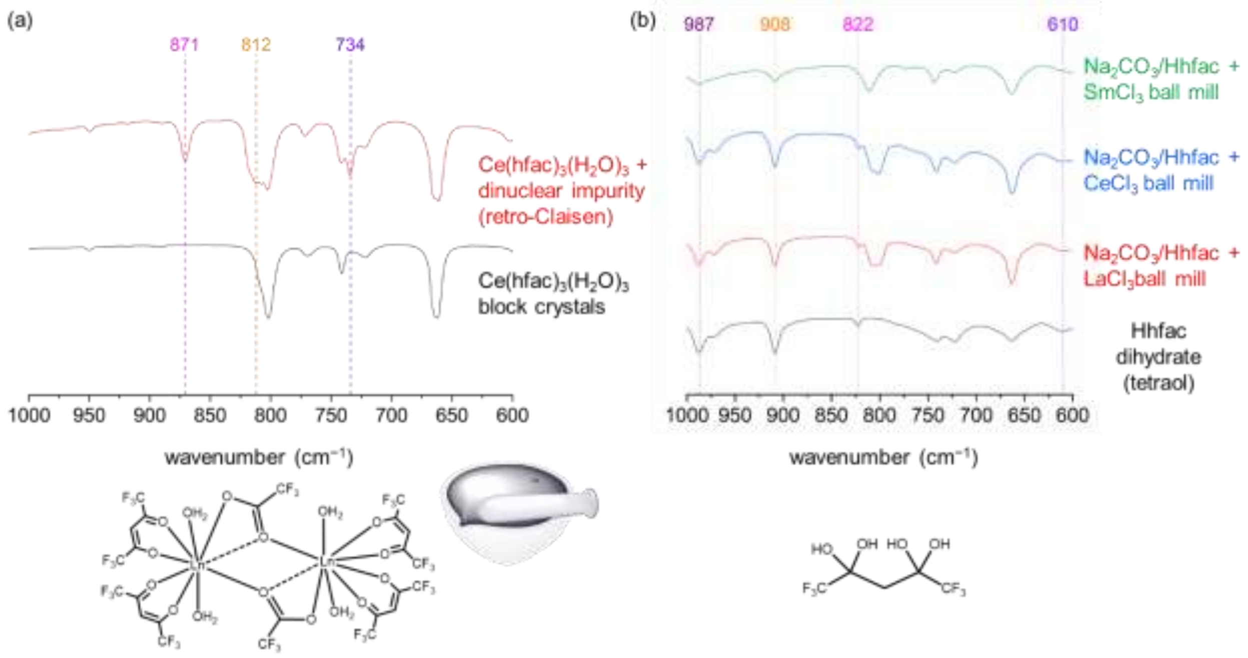


**Figure S66**: FT-IR spectral handles for most common impurities from mechanochemical reactions (a) FT-IR Spectra for isolated block crystals of $Ce(hfac)_3(H_2O)_3$ (seen in **Figure S62**) and polycrystalline material of $Ce(hfac)_3(H_2O)_3$ and dinuclear impurity $[Ce(hfac)_2(CF_3COO)(H_2O)_2]_2$ (b) FT-IR spectra of the tetraol species and the ball milling reactions showing common peaks (Nujol, 32 scans, 2 $cm^{-1}$ res.).

For $Ln(hfac)_3(H_2O)_x$ products solution syntheses show similar IR spectra amongst themselves with multiple broad OH stretches observed, the M1-SAG method yielded similar IR spectra with one sharp and one broad OH stretch and the M2-slurry method yielded another family of IR spectra, which were similar to the products obtained from the M1-SAG method. The $Sm(hfac)_3(H_2O)_2$ -OH region was different from the larger lanthanides, as expected based on its smaller size and lower hydration number. The hydrogen bonding region also changes depending on the method employed, with the solution syntheses having a more complex structure, whereas there appear to be fewer peaks in complexes prepared from the manual grinding M1 and M2 methods. As expected for La-Nd, most complexes have a clear peak *ca*. 3690 $cm^{-1}$ which is indicative of $Ln(hfac)_3(H_2O)_3$ formation (**SI-section 5a**).[1]

**Table S10: Useful spectroscopic handles for impurities in synthesizing Ln(hfac)$_3$(H$_2$O)$_x$.**

| | IR (cm$^{-1}$) | $^1$H NMR (ppm) ($d_6$-acetone) | $^{19}$F NMR (ppm) (ref to hfb at −164.67 ppm in $d_6$-acetone) |
|---|---|---|---|
| **Hhfac (enol)**[a] | | 6.6 singlet[12] | −77.32, (−77.33 shoulder), small peaks at −74.7, −76.9 −79.7 (multiple peaks), −75.8[12] |
| **Tetraol** | *ca*. 988[13,b], 908[13,b], 822w[13], 610w-sh | 7.04 singlet<br>6.96[12] | −87.9[c]<br>less intense −86.8 peak also observed |
| **Retro-Claisen ($CF_3COO$)**[1 d] | *ca*.870[b], 734[b]<br>+3665 for Tb | No $^1$H signal | −76.0 ($Na^+$)<br>−77.0 ($La^{3+}$) |
| **$Na_2La(hfac)_5\cdot 3H_2O$** | 3716, 3639, 795 | 5.8 singlet | −77.5 (major), −76.3 (minor) |

[a] Wet $d_6$-acetone used, over time peaks at −86.7 and −87.8 become more prominent
[b] Retained solvent ($Et_2O$) in complexes can lead to similar peaks.
[c] −86.13 ppm in $d_6$-acetone, −88.05 ppm in $D_2O$ (ref. Aygen and Eldik, *ChemBer* 1989)[12]
[d] $^{13}$C NMR used when impurity present in sufficient quantities

**Table S11**: **Common impurities found alongside Ln(hfac)$_3$(H$_2$O)$_x$ shortly following isolation for La-Sm, Tb.**

| **Impurity or Product (where observed)** | **EA** | **Further characterization** |
|---|---|---|
| Retro-Claisen (solution, M1-SAG, M2 open mortar and pestle) | Low %C, Low %H | IR peaks ~ 871, 734[1]<br>If possible, confirm with $^{13}$C NMR in sufficient amounts.<br>For Tb complexes, peaks at 868 and 734 can also be associated with solvent incorporation. (**Figure S67**). |
| Tetraol or solvent (Tetraol: solution and ball milling, solvent: all extracted reactions) | High %C, High %H | For tetraol peaks at 989 and 909[13], ensure sample well ground or is already a fine powder. Solvent will evaporate readily, tetraol will not. Must have $^1$H NMR at 7.04, 2.24 for tetraol, $Et_2O$, hexanes and EtOH solvent signals.[3] |
| $Na_2Ln(hfac)_5\cdot 3H_2O$ (M1-SAG isolated, crude ball milling) | High %C, Low %H | Sharp IR peaks at 3716, 3639 peak at ~795 cm$^{-1}$ |
| Dihydrate for La-Nd (M2-Pr) | High %C, Low %H | IR peak at *ca*. 3690 cm$^{-1}$ absent[1] |

## Solvent incorporation from samples immediately obtained after extraction

Following extraction, the open mortar and pestle samples can form larger grain sizes, these can hold solvent. The effects of solvent incorporation are shown in **Figures S67 to S69**. Note that some of the peaks that disappear are also consistent with the retro-Claisen impurity and the tetraol in terms of peak position; however, based on quick re-grinding (30 sec.) of the sample, the peaks are actually consistent with solvent. These peaks are extremely weak in the IR. For this reason, $^1$H

and $^{19}F$ NMR should also be consulted to look for peaks associated with tetraol. For the early lanthanides such as $Ce(hfac)_3(H_2O)_3$, the peaks in the fingerprint region that correspond with possible tetraol disappear with simply grinding to a finer solid, then adding Nujol. For two of the M1-SAG Tb samples, peaks at 988, 909, 868 and 734 disappeared with reanalysis of the sample. If the retro-Claisen is present in sufficient amounts, it can be most easily identified by $^{13}C$ NMR. The M2 sample of $Tb(hfac)_3(H_2O)_2$ even with grinding the sample finer showed the same intensity of peaks associated with retro-Claisen.

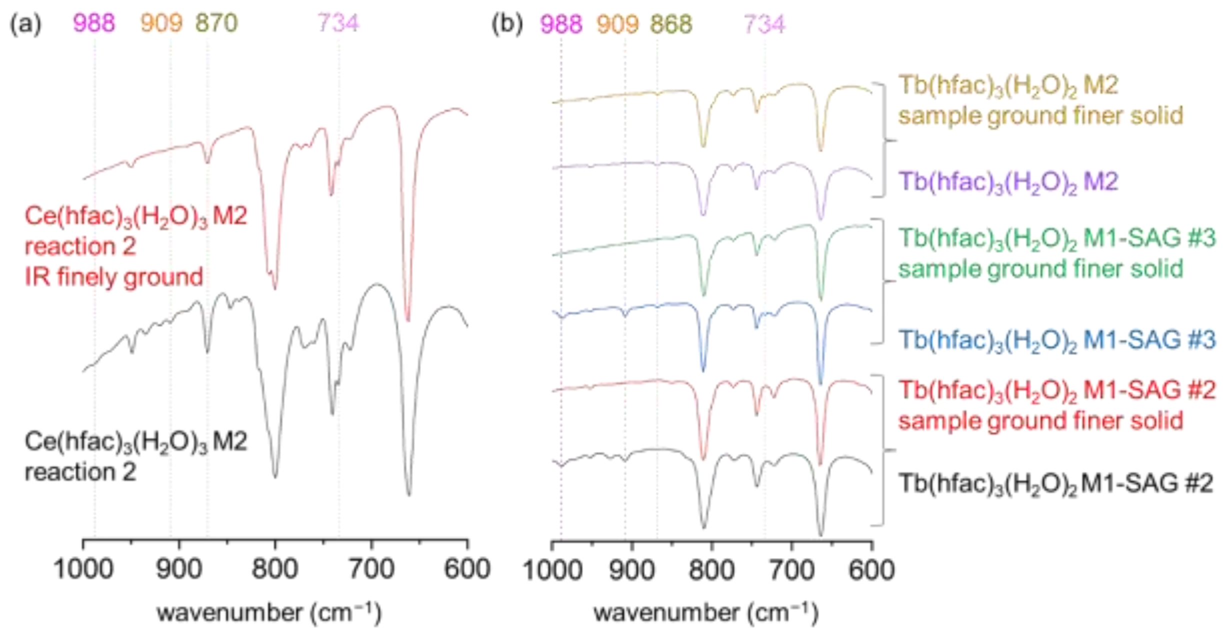


**Figure S67:** The effect of grinding large granular crystals of (a) $Ce(hfac)_3(H_2O)_3$ M2 slurry (b) $Tb(hfac)_3(H_2O)_2$ for IR analysis (Nujol, 2 $cm^{-1}$ res.). For (a) IR spectra for well ground solid in Figure S60 and (b) in main text Figure 9.

A similar effect was seen for the pentakis complex of $Na_2Pr(hfac)_5{\cdot}3H_2O$ where there was a disappearance of the characteristic sharp OH peaks and the appearance of additional peaks at ca. 1700 $cm^{-1}$ (**Figure S68**). This was also seen for the small amount of crystal sample of $Na_2Pr(hfac)_5{\cdot}3H_2O{\cdot}Et_2O$ (**Figure S20**).

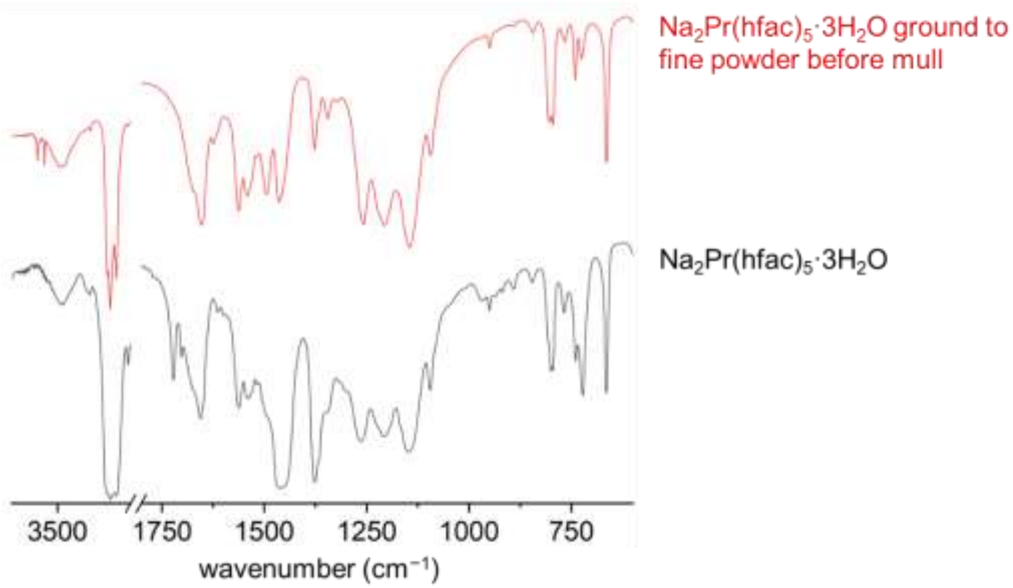


**Figure S68:** The effect of grinding large granulated material of $Na_2Pr(hfac)_5 \cdot 3H_2O$. Top spectrum from Figure 2 in main text. (Nujol, 2 $cm^{-1}$ res.).

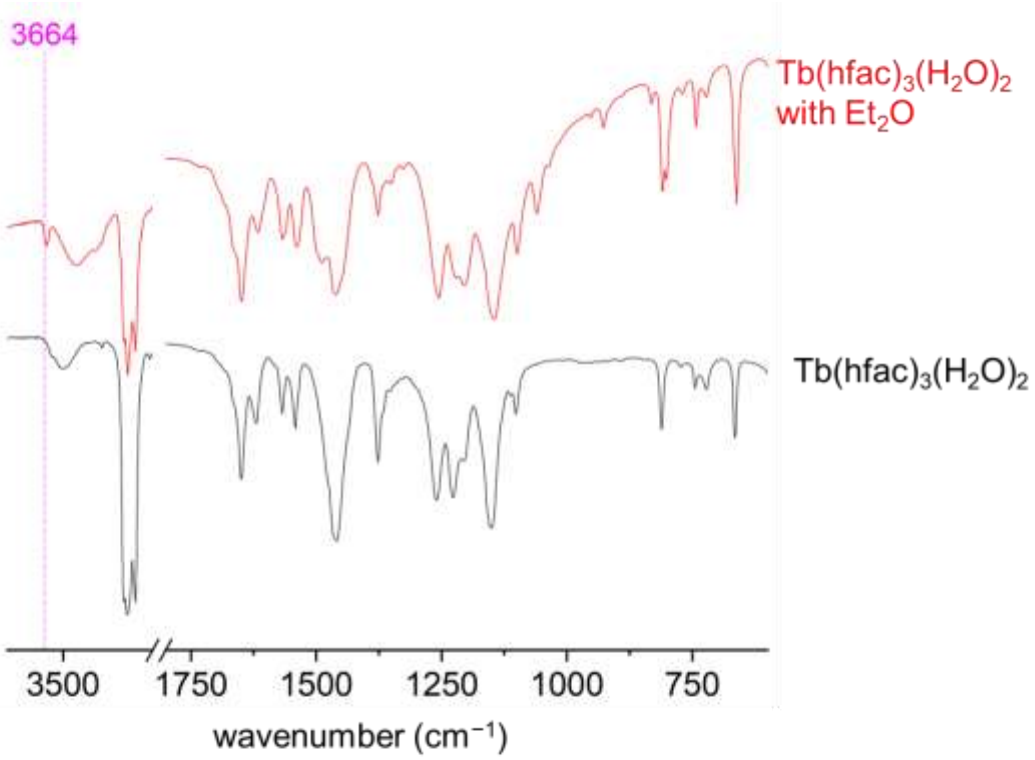


**Figure S69:** The effect of adding $Et_2O$ to $Tb(hfac)_3(H_2O)_2$ Bottom spectrum seen in Figure 9 in main text (M1-SAG #4). (Nujol, 2 $cm^{-1}$ res.).

**S8. DSC-TGA Studies of $Ln(hfac)_3(H_2O)_x$ Complexes**

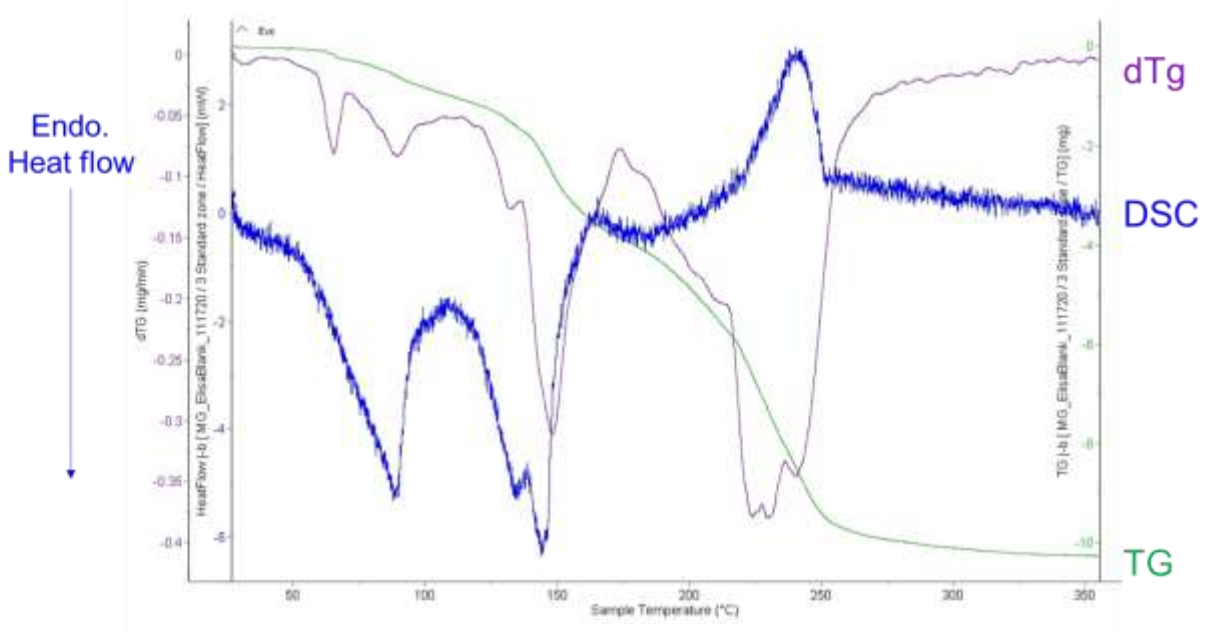


**Figure S70:** Combined TGA-DSC Plot of solution derived $La(hfac)_3(H_2O)_3$ ($T_{initial}$ = 25 °C, $T_{ramp}$= 3 °C/min.)

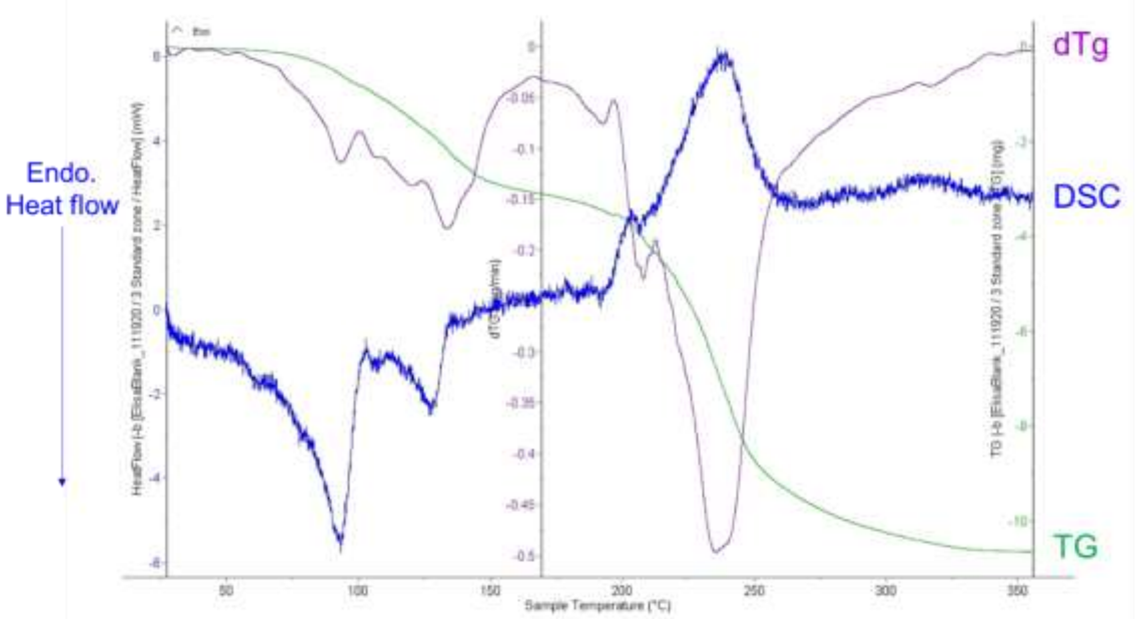


**Figure S71**: Combined TGA-DSC Plot of M1-SAG $La(hfac)_3(H_2O)_3$ ($T_{initial}$ = 25 °C, $T_{ramp}$= 3 °C/min.)

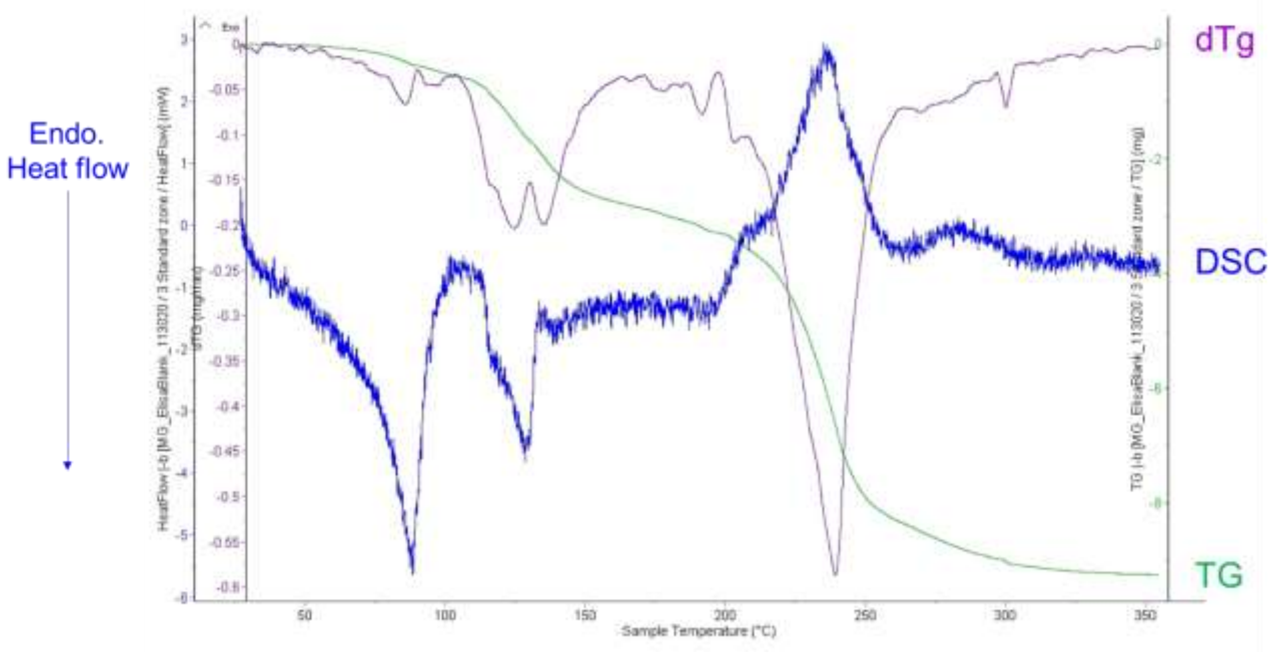


**Figure S72**: Combined TGA-DSC Plot of M2 slurry La(hfac)$_3$($H_2O$)$_3$ ($T_{initial}$ = 25 °C, $T_{ramp}$= 3 °C/min.)

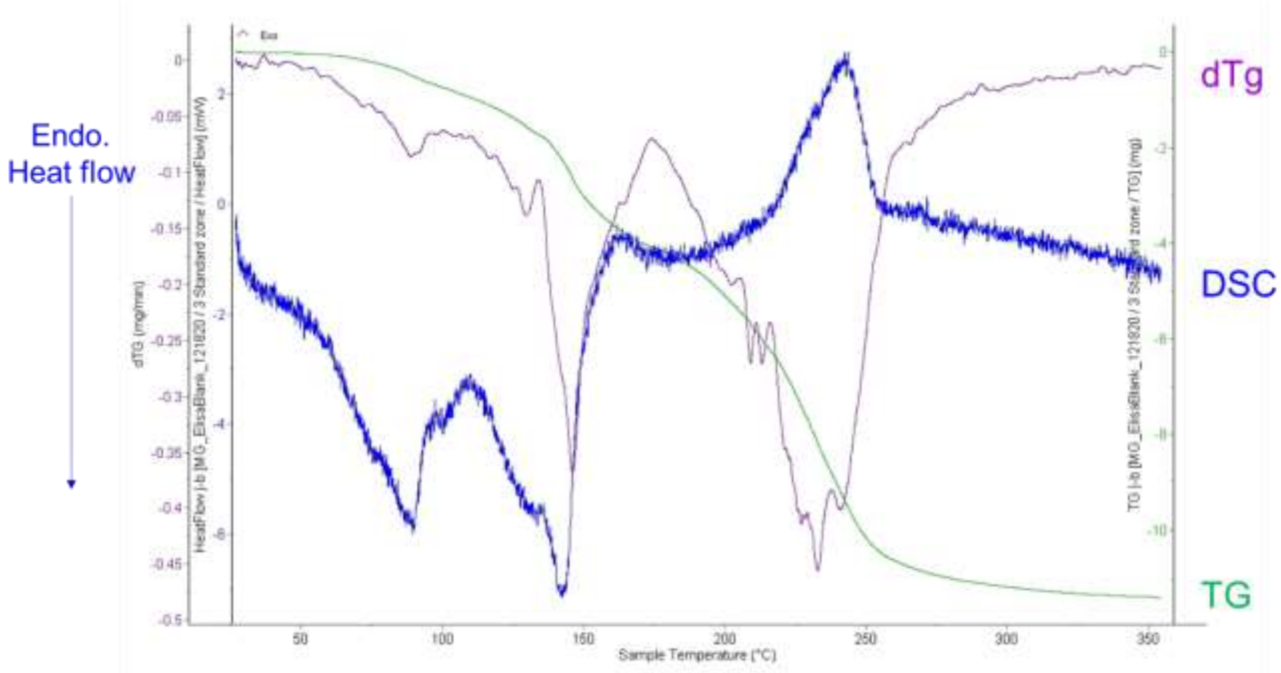


**Figure S73:** Combined TGA-DSC Plot of solution derived Ce(hfac)$_3$($H_2O$)$_3$ ($T_{initial}$ = 25 °C, $T_{ramp}$= 3 °C/min.)

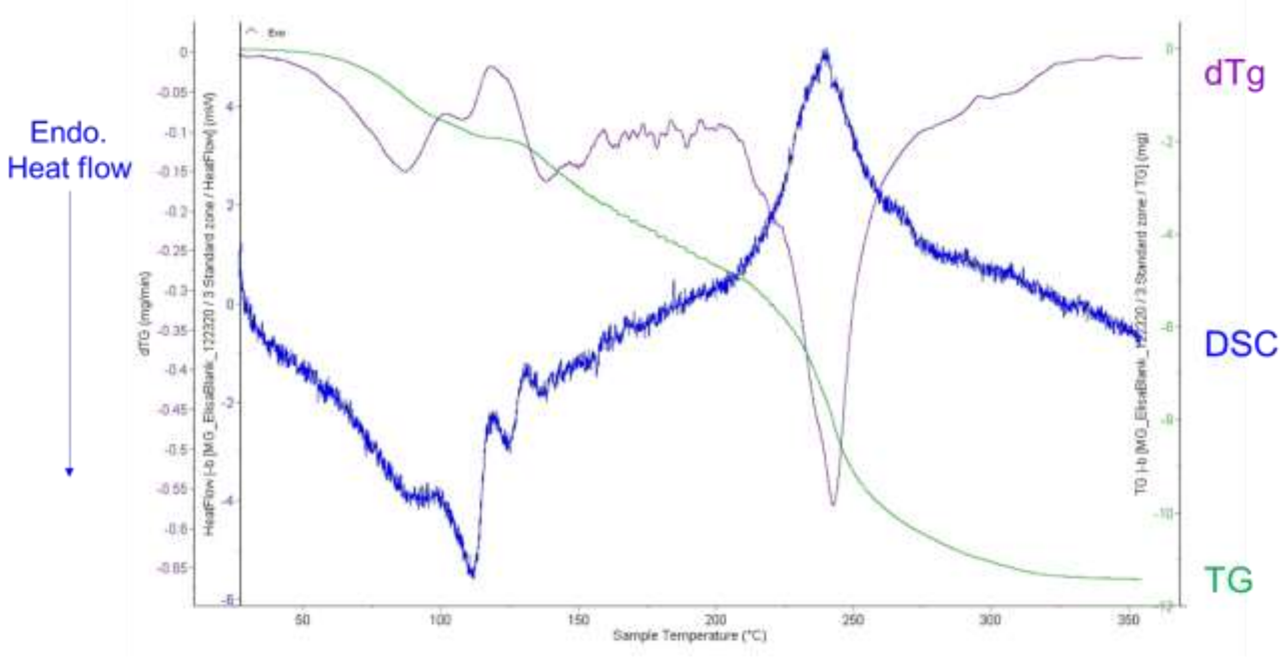


**Figure S74**: Combined TGA-DSC Plot of M1-SAG Ce(hfac)3(H2O)3 ($T_{initial}$ = 25 °C, $T_{ramp}$= 3 °C/min.)

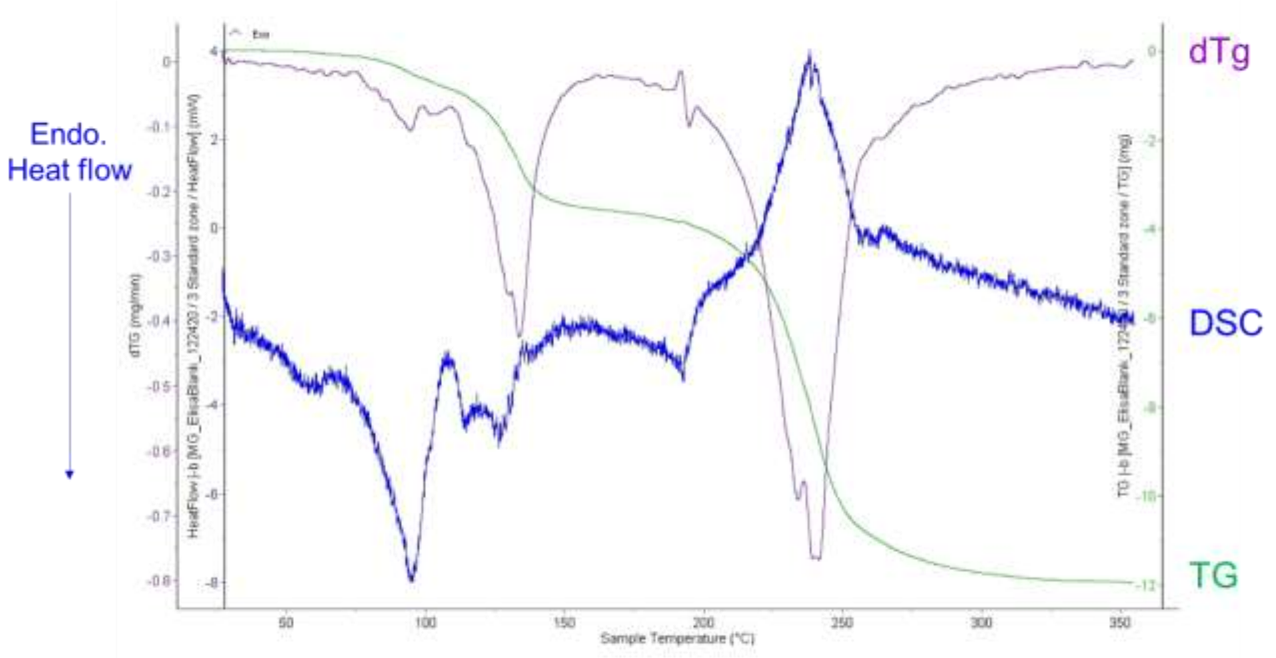


**Figure S75**: Combined TGA-DSC Plot of M2 slurry Ce(hfac)3(H2O)3 ($T_{initial}$ = 25 °C, $T_{ramp}$= 3 °C/min.)

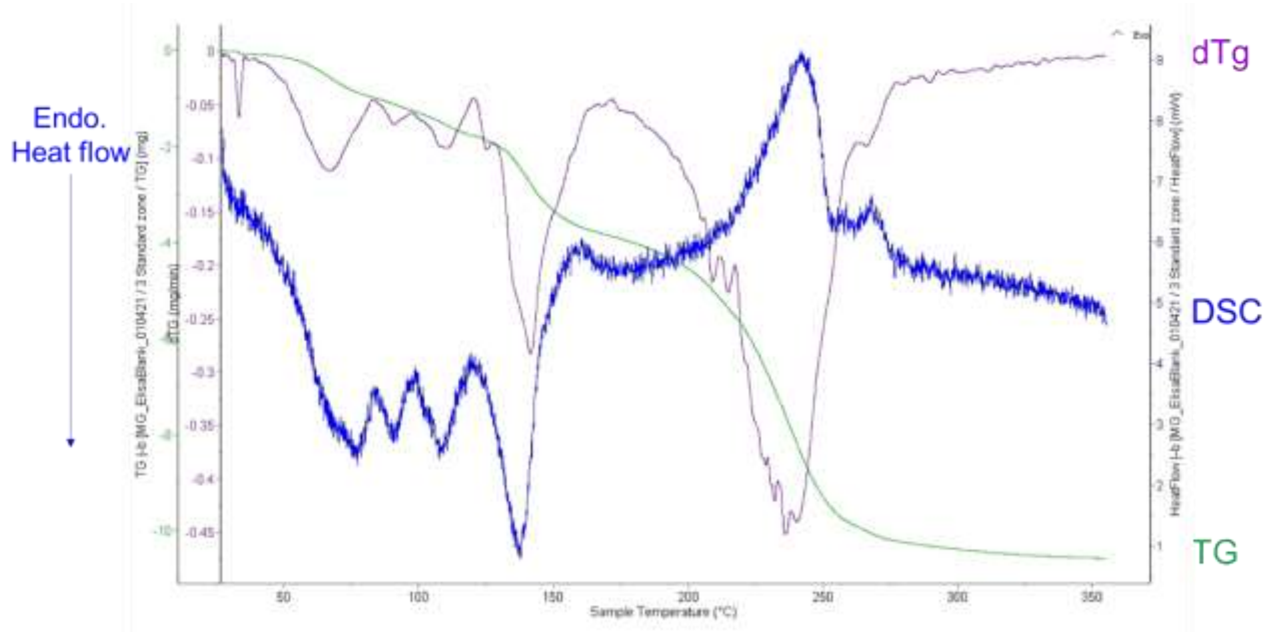


**Figure S76:** Combined TGA-DSC Plot of solution derived $Pr(hfac)_3(H_2O)_3$ ($T_{initial}$ = 25 °C, $T_{ramp}$= 3 °C/min.)

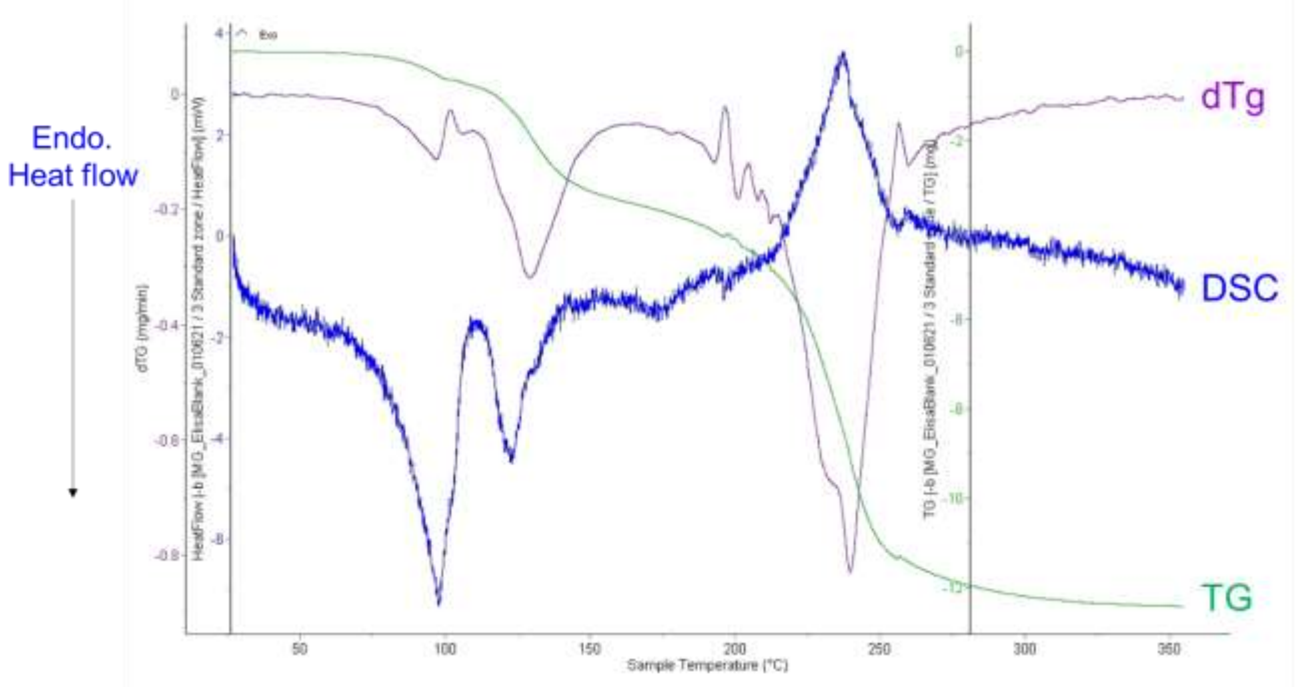


**Figure S77**: Combined TGA-DSC Plot of M1-SAG $Pr(hfac)_3(H_2O)_3$ ($T_{initial}$ = 25 °C, $T_{ramp}$= 3 °C/min.)

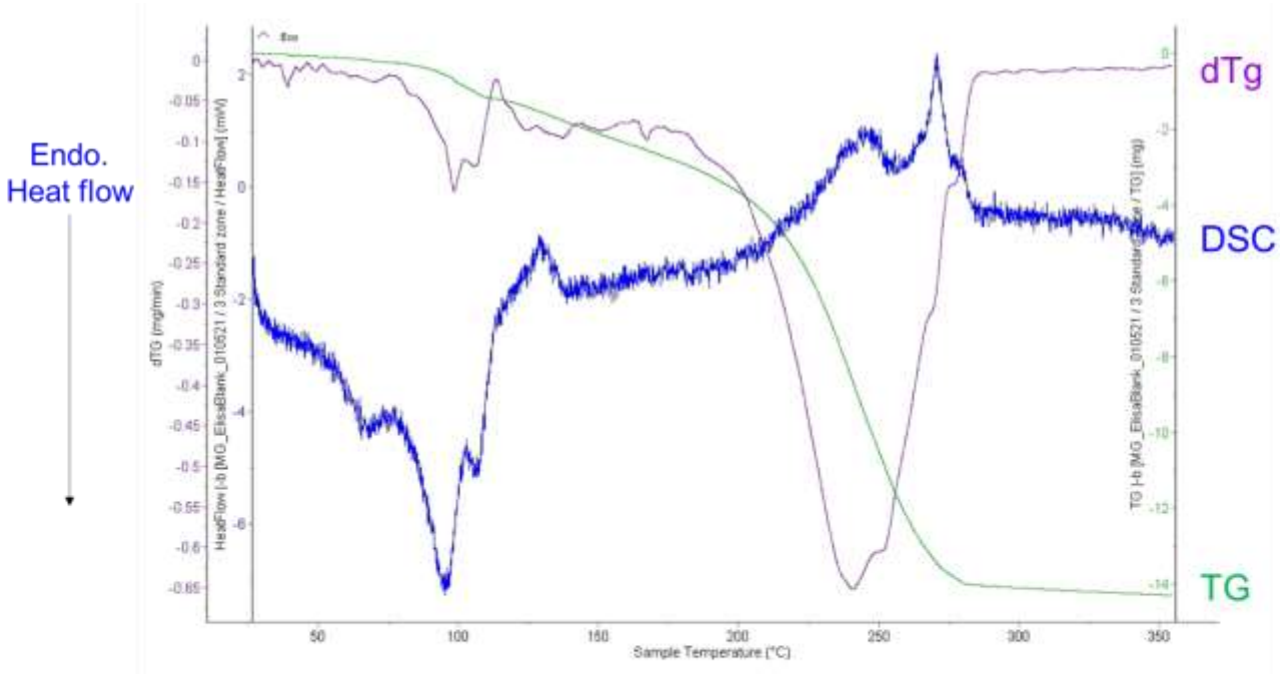


**Figure S78**: Combined TGA-DSC Plot of M2 slurry Pr(hfac)$_3$(H$_2$O)$_3$ (T$_{initial}$ = 25 °C, T$_{ramp}$= 3 °C/min.)

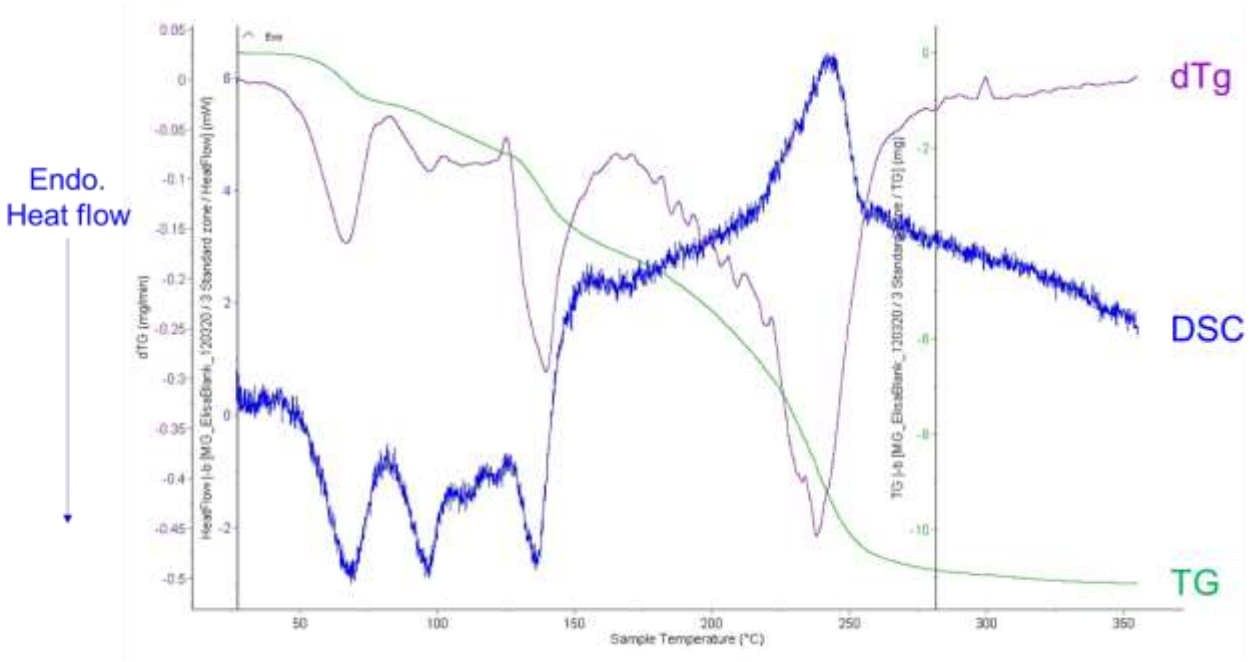


**Figure S79:** Combined TGA-DSC Plot of solution derived Nd(hfac)$_3$(H$_2$O)$_3$ (T$_{initial}$ = 25 °C, T$_{ramp}$= 3 °C/min.)

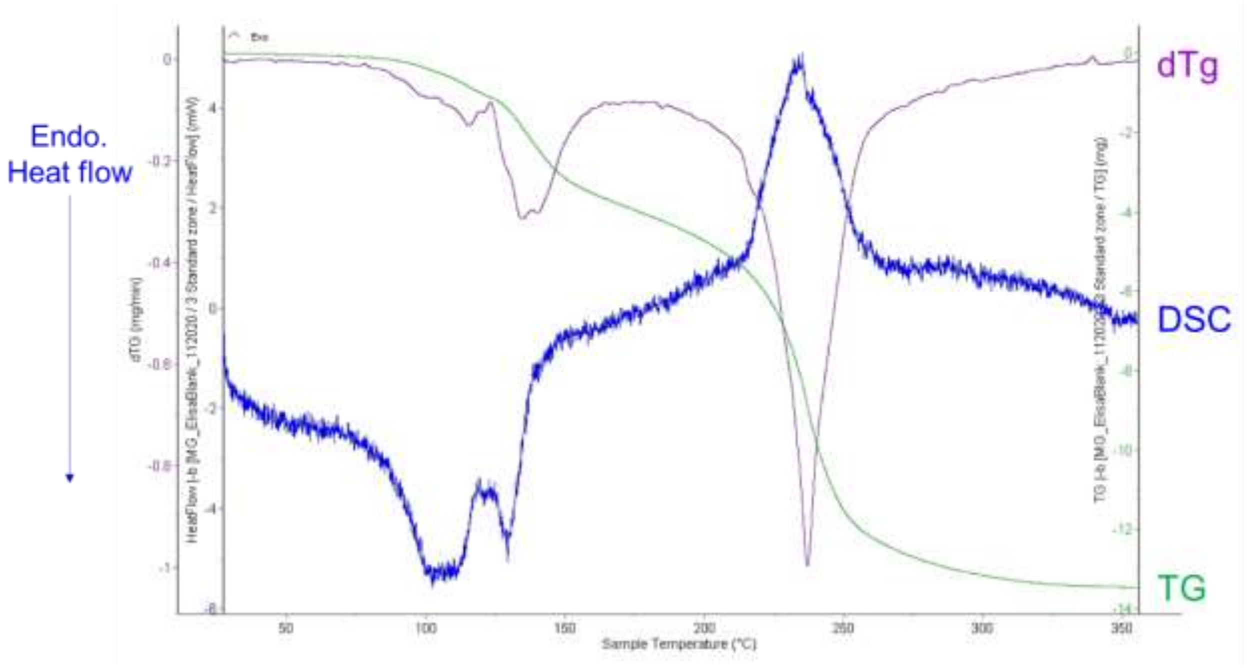


**Figure S80**: Combined TGA-DSC Plot of M1-SAG Nd(hfac)3(H2O)3 ($T_{initial}$ = 25 °C, $T_{ramp}$= 3 °C/min.)

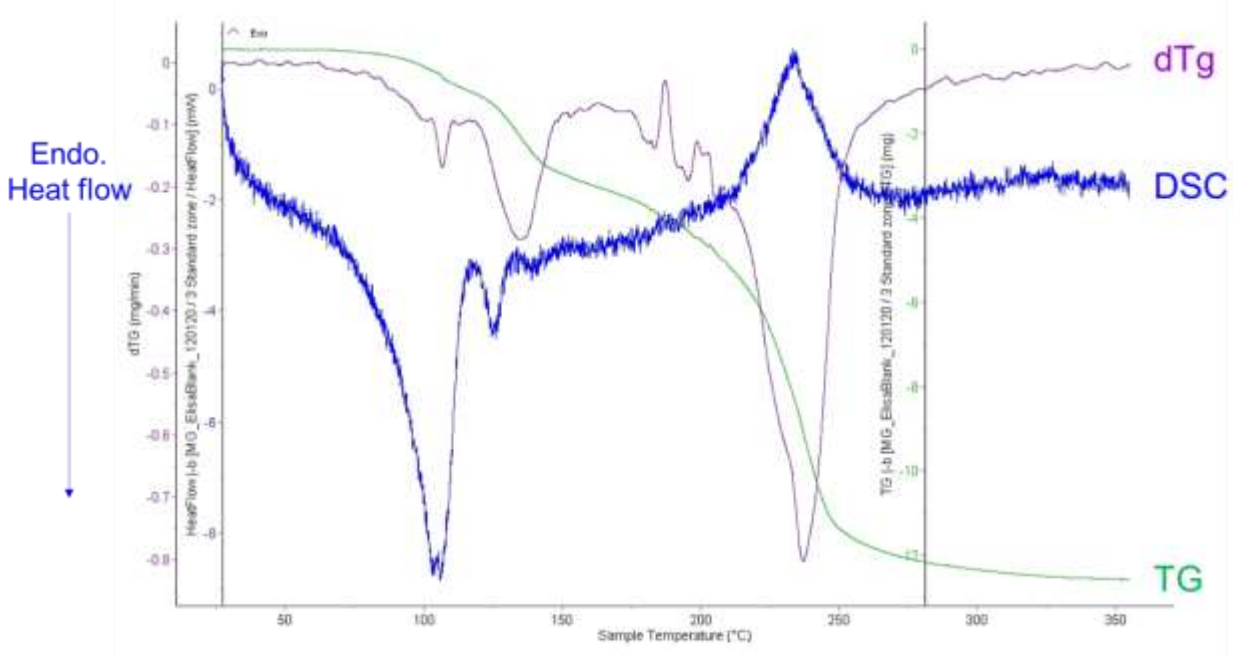


**Figure S81**: Combined TGA-DSC Plot of M2 slurry Nd(hfac)3(H2O)3 ($T_{initial}$ = 25 °C, $T_{ramp}$= 3 °C/min.)

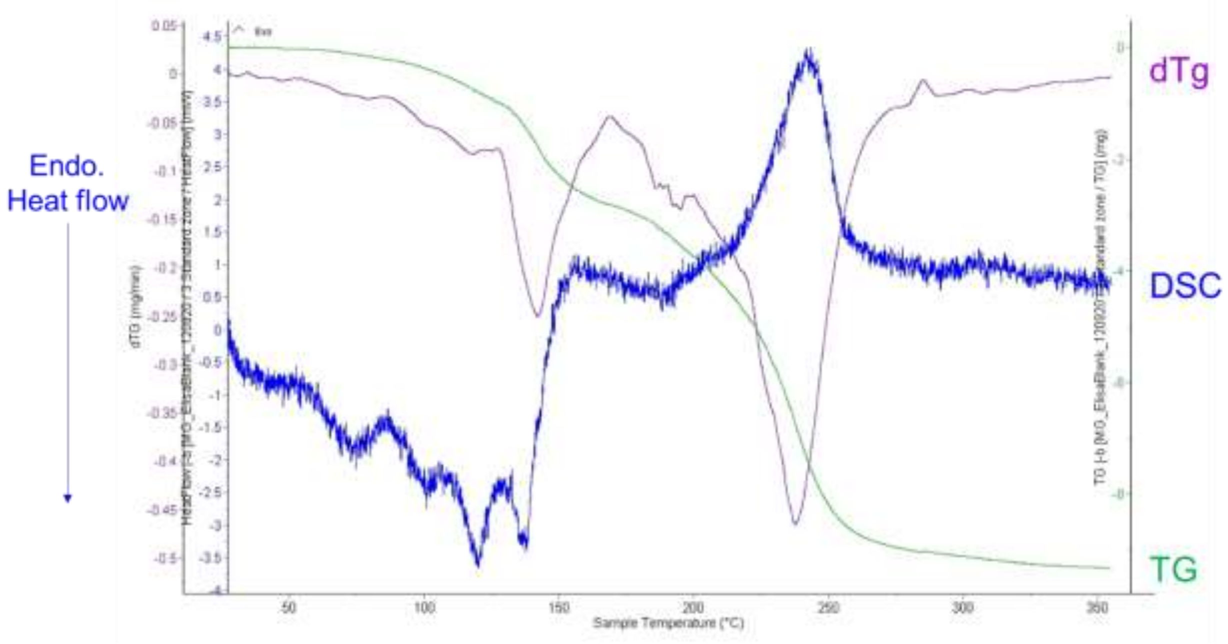


**Figure S82:** Combined TGA-DSC Plot of solution derived Sm(hfac)$_3$($H_2O$)$_2$ ($T_{initial}$ = 25 °C, $T_{ramp}$= 3 °C/min.)

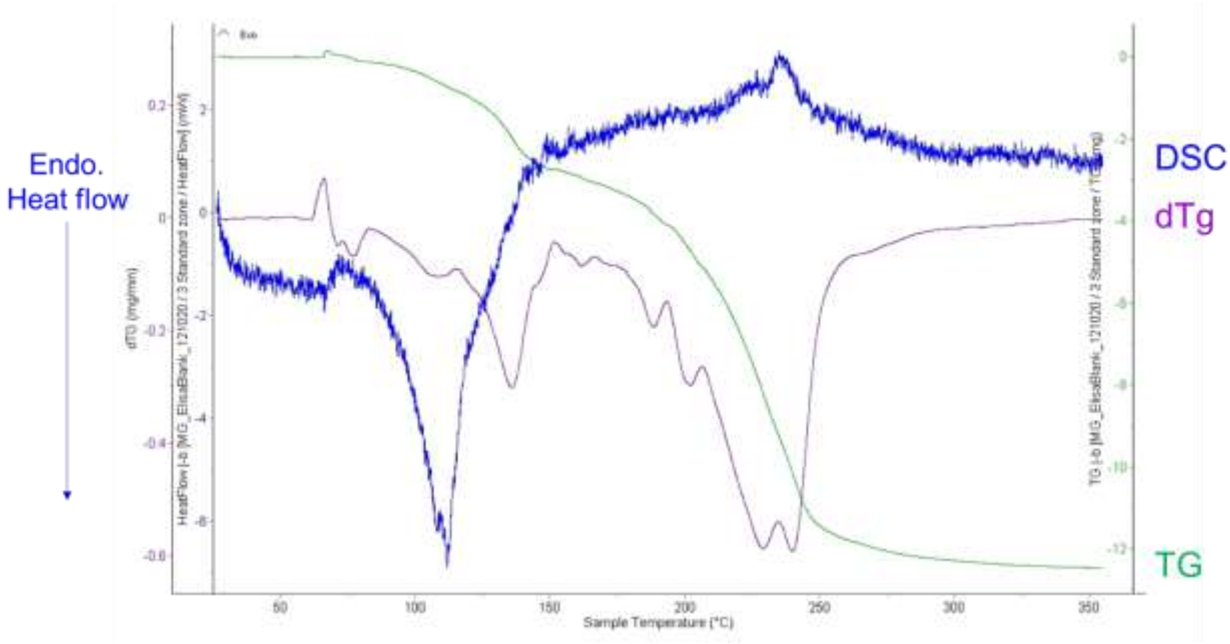


**Figure S83**: Combined TGA-DSC Plot of M1-SAG Sm(hfac)$_3$($H_2O$)$_2$ ($T_{initial}$ = 25 °C, $T_{ramp}$= 3 °C/min.)

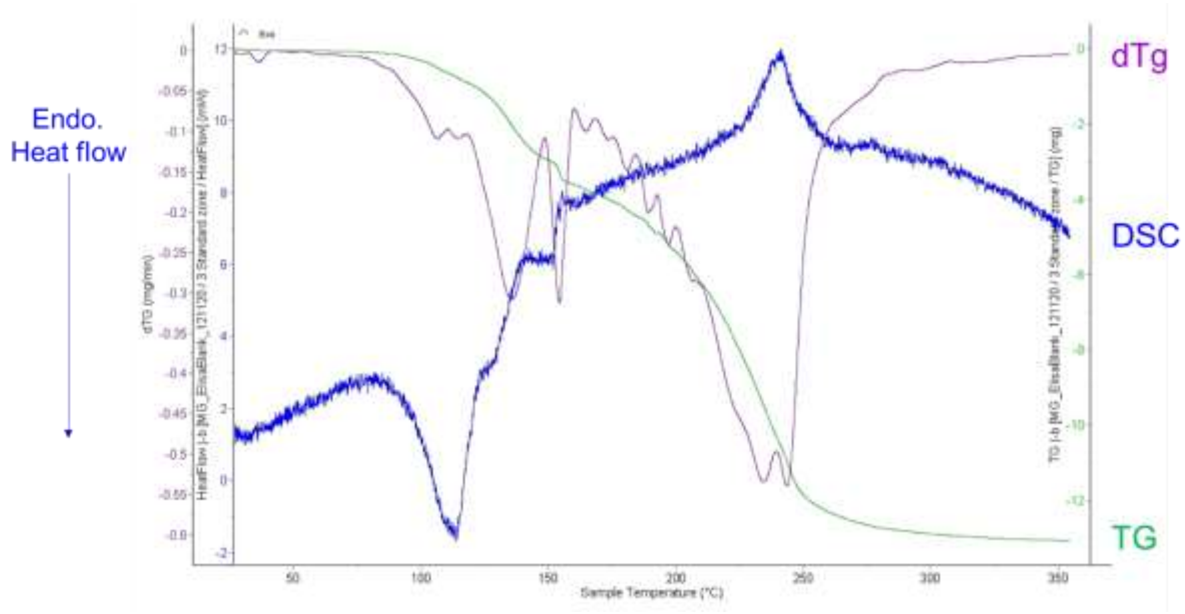


**Figure S84**: Combined TGA-DSC Plot of M2 slurry $Sm(hfac)_3(H_2O)_2$ ($T_{initial}$ = 25 °C, $T_{ramp}$= 3 °C/min.)

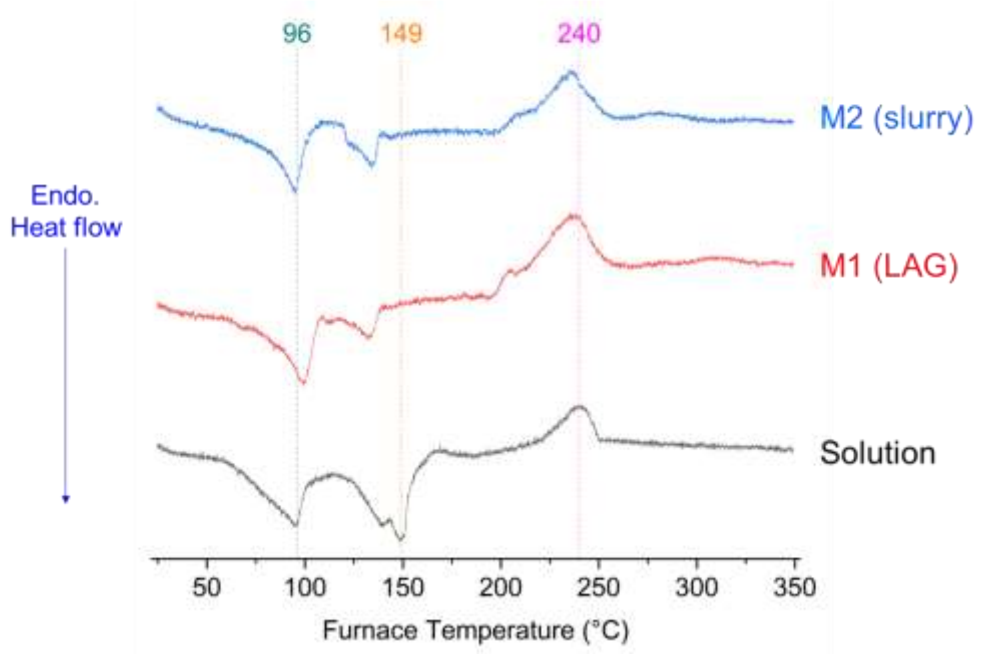


**Figure S85:** DSC plots of the various methods for synthesizing $La(hfac)_3(H_2O)_3$ ($T_{initial}$ = 25 °C, $T_{ramp}$= 3 °C/min.). Contains DSC data from same run in Figures S70-S72.

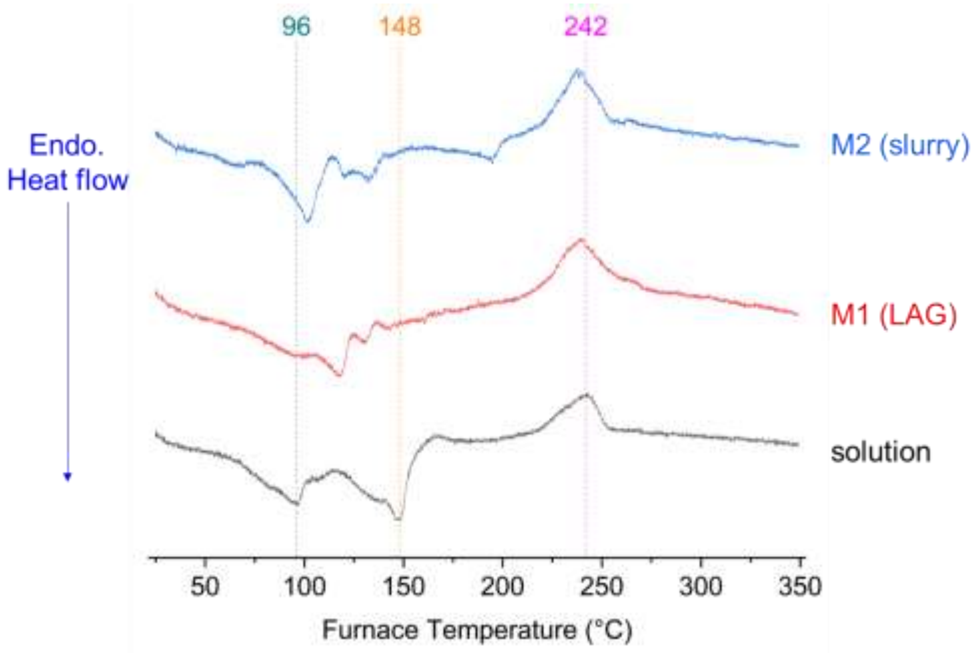


**Figure S86**: DSC plots of the various methods for synthesizing Ce(hfac)$_3$($H_2O$)$_3$ ($T_{initial}$ = 25 °C, $T_{ramp}$= 3 °C/min.)  Contains DSC data from same run in Figures S73-S75.

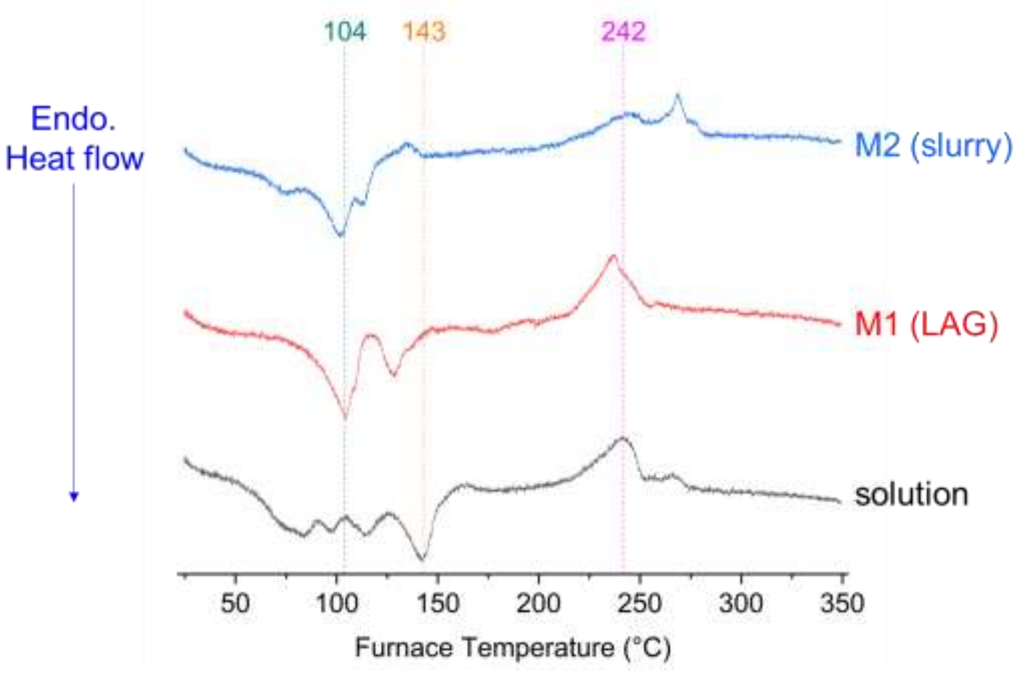


**Figure S87**: DSC plots of the various methods for synthesizing Pr(hfac)$_3$($H_2O$)$_3$ ($T_{initial}$ = 25 °C, $T_{ramp}$= 3 °C/min.) Contains DSC data from same run in Figures S76-S78.

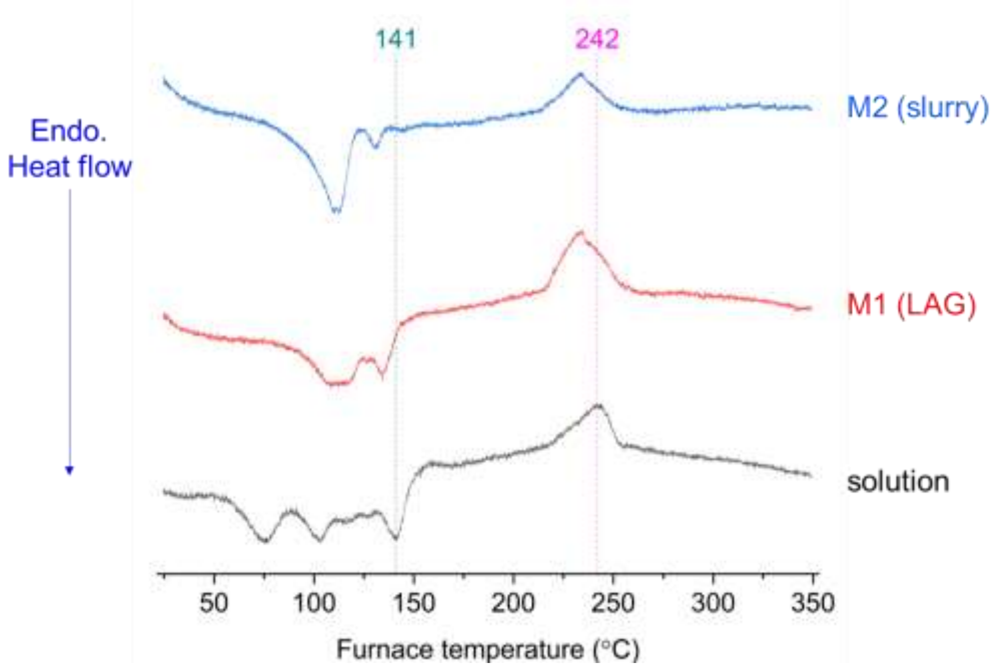


**Figure S88**: DSC plots of the various methods for synthesizing $Nd(hfac)_3(H_2O)_3$ ($T_{initial}$ = 25 °C, $T_{ramp}$= 3 °C/min.) Contains DSC data from same run in Figures S79-S81.

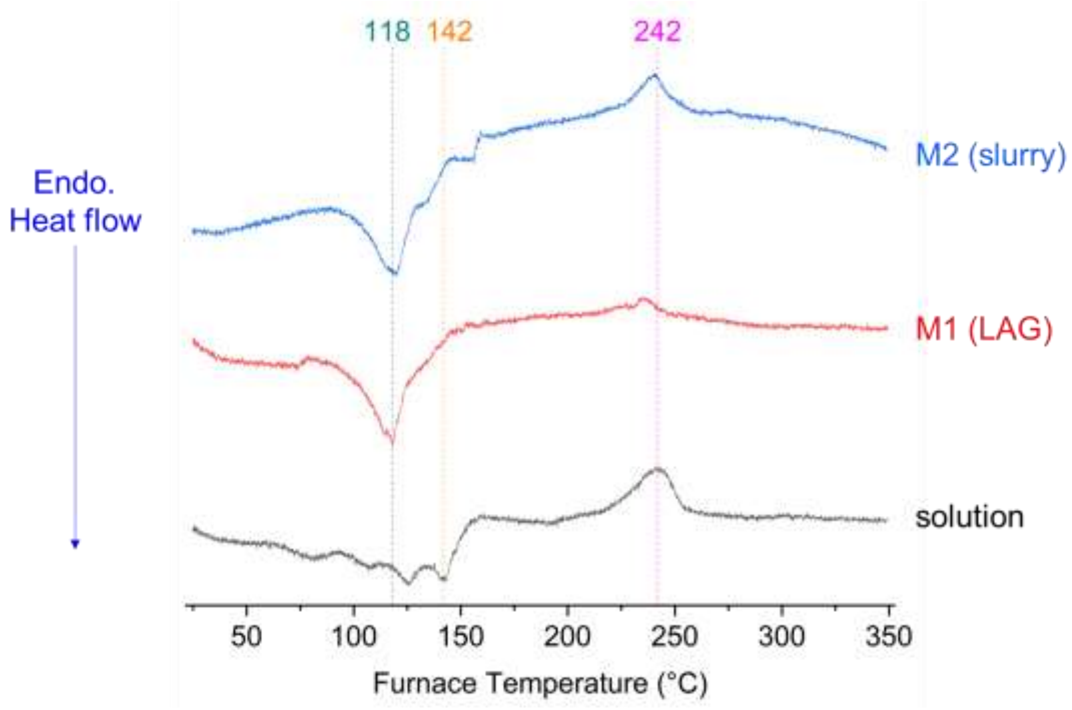


**Figure S89**: DSC plots of the various methods for synthesizing $Sm(hfac)_3(H_2O)_2$ ($T_{initial}$ = 25 °C, $T_{ramp}$= 3 °C/min.) Contains DSC data from same run in Figures S82-S84.

## S9: PXRD Studies of Complexes

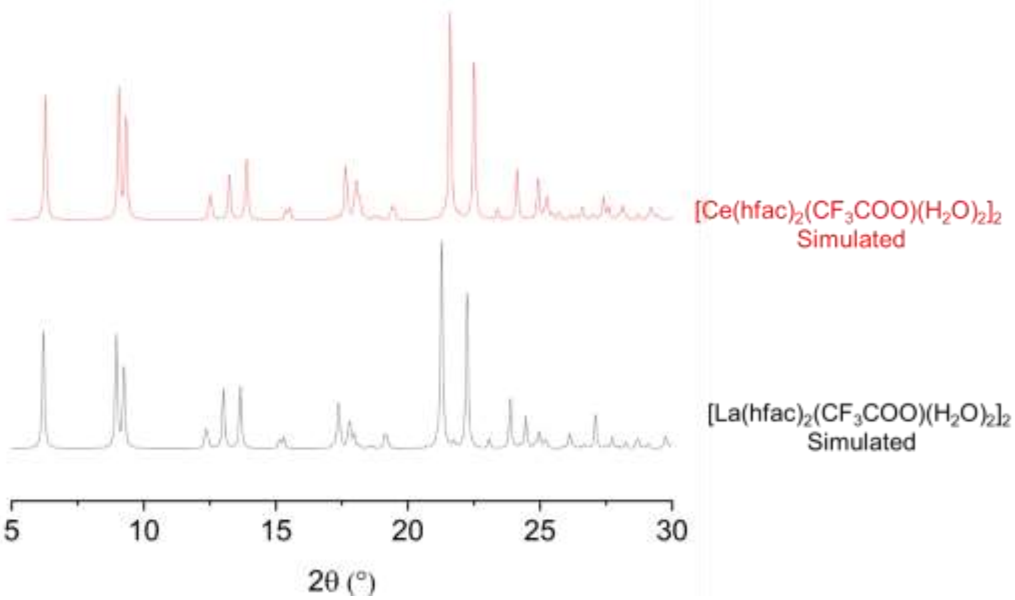


**Figure S90**: Comparison of simulated PXRD patterns for [La(hfac)$_2$(CF$_3$COO)(H$_2$O)$_2$]$_2$ (293(2) K) (Refcode: KAMVAW) [14] and [Ce(hfac)$_2$(CF$_3$COO)(H$_2$O)$_2$]$_2$ ($T$=123 K) ($\lambda$=1.54056 Å).

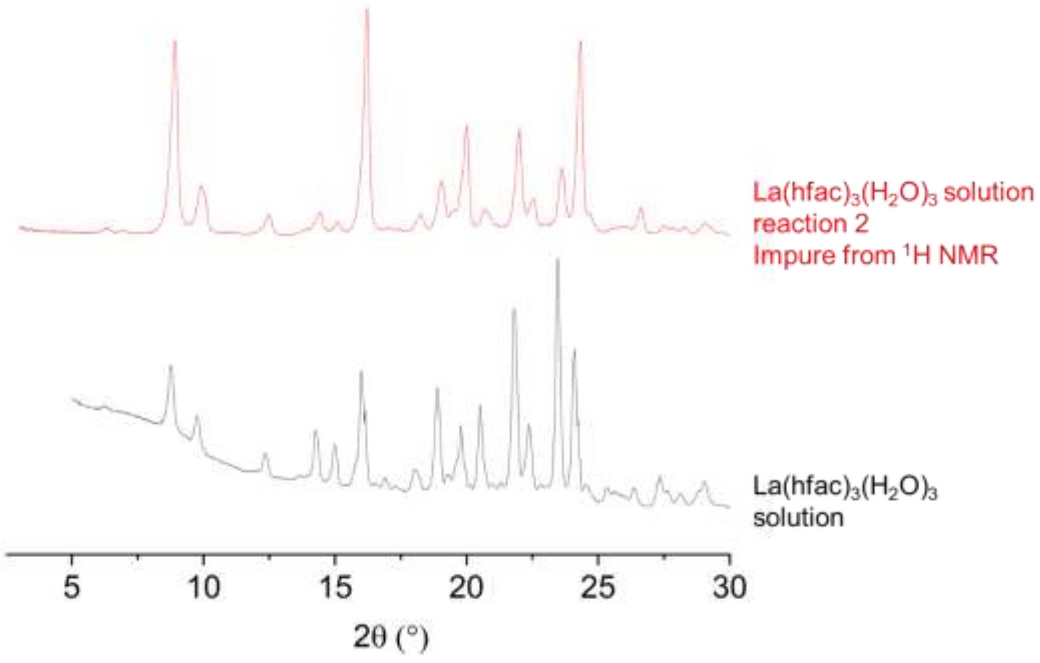


**Figure S91**: Comparison of PXRD patterns for two reactions to prepare La(hfac)$_3$(H$_2$O)$_3$ using the solution method. Bottom diffraction pattern from **Figure 7a** in main text.

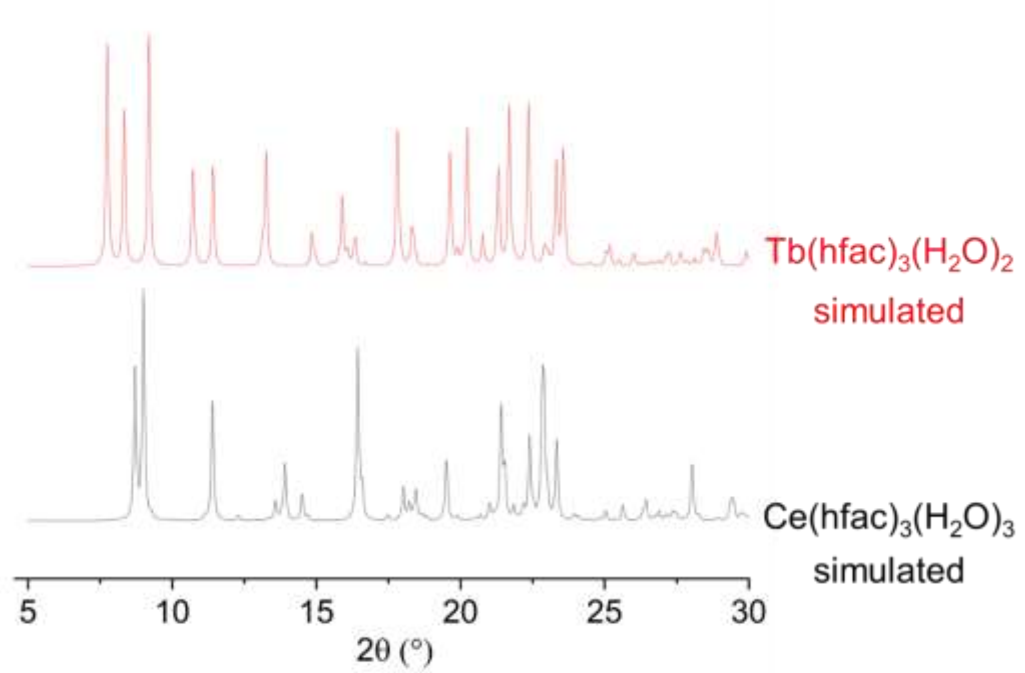


**Figure S92**: Comparison of simulated PXRD patterns for Ce(hfac)3(H2O)3 ($T$=123 K) and Tb(hfac)3(H2O)2 (293(2) K) (Refcode: NALCEL) [15] (λ=1.54056 Å).

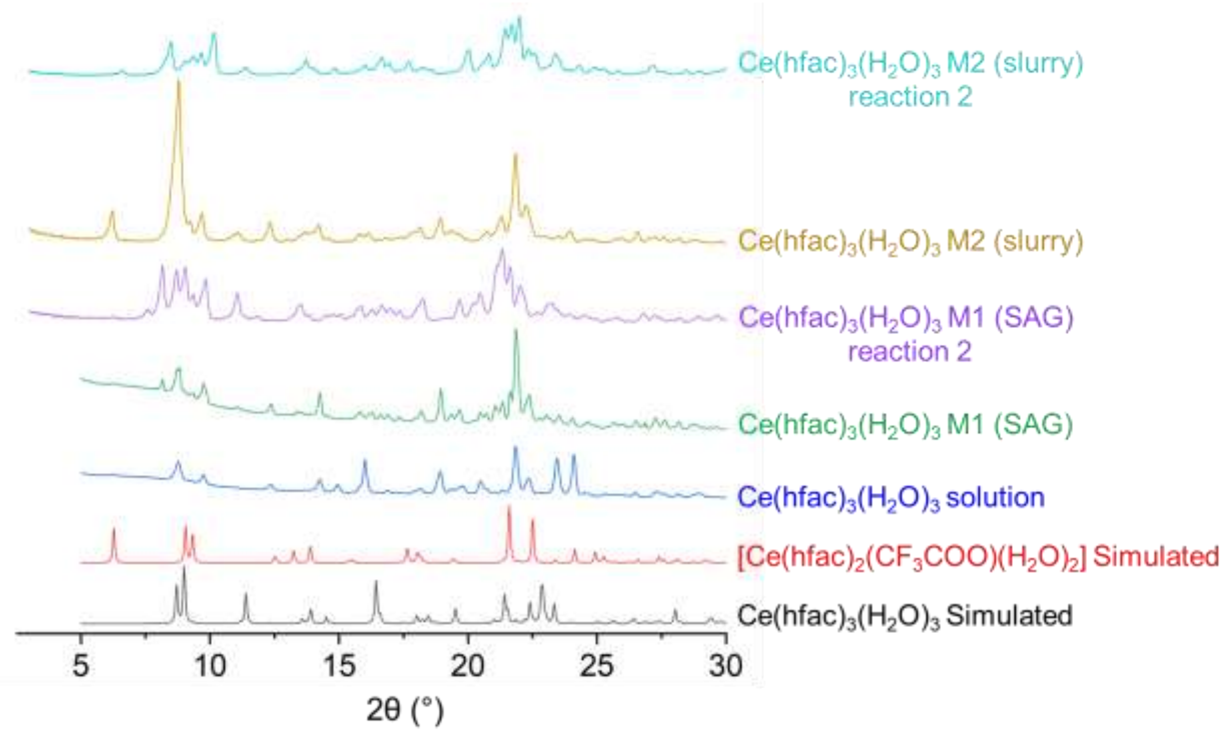


**Figure S93:** PXRD patterns for the various methods for synthesizing Ce(hfac)3(H2O)3 including repeat reactions and comparison to simulated PXRD patterns (λ=1.54056 Å, $T$=123 K).

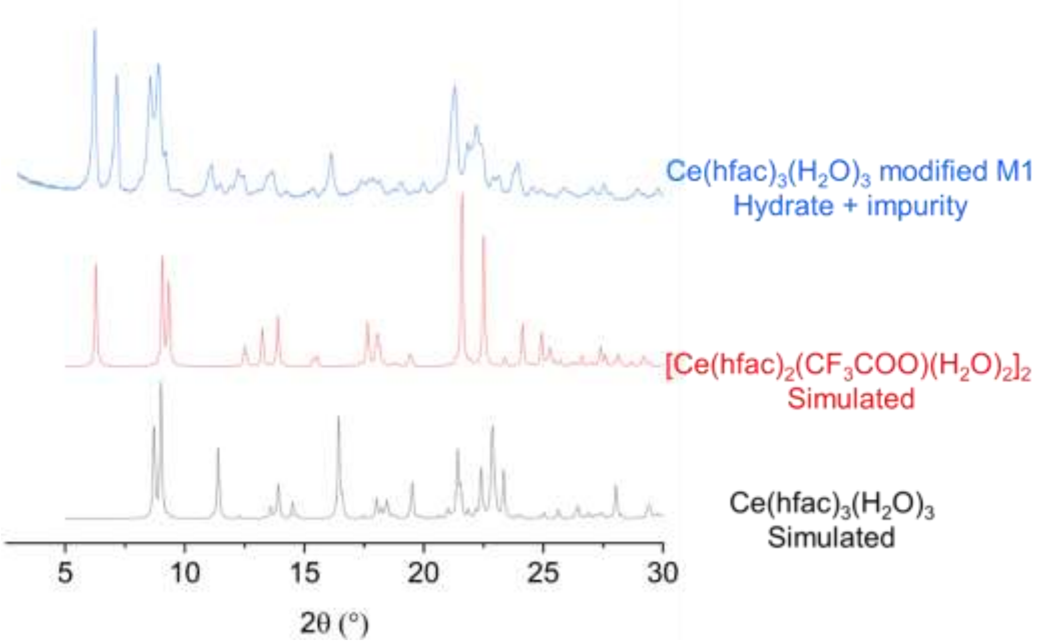


**Figure S94**: PXRD patterns for the bulk Ce(hfac)$_3$(H$_2$O)$_3$ material containing blocks (hydrates) and plates (dinuclear impurity) and simulated PXRD patterns (λ=1.54056 Å, *T*=123 K).

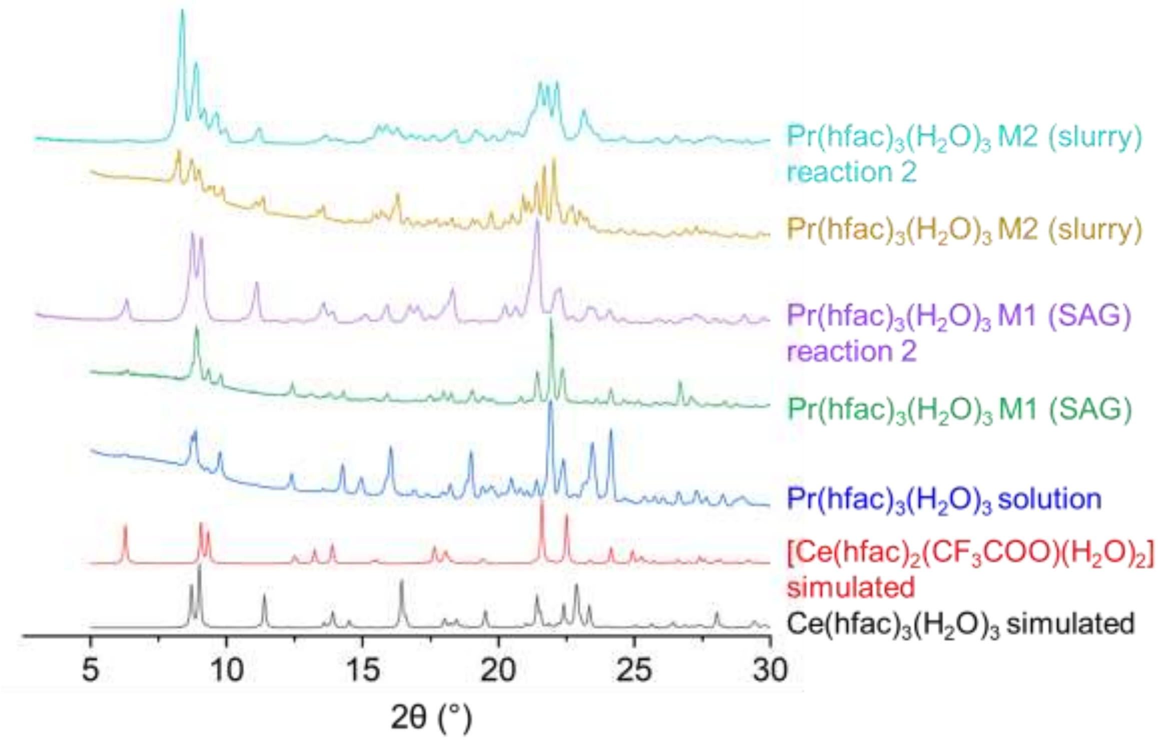


**Figure S95:** PXRD patterns for the various methods for synthesizing Pr(hfac)$_3$(H$_2$O)$_3$ including repeat reactions and comparison to simulated PXRD patterns (λ=1.54056 Å, *T* = 123 K).

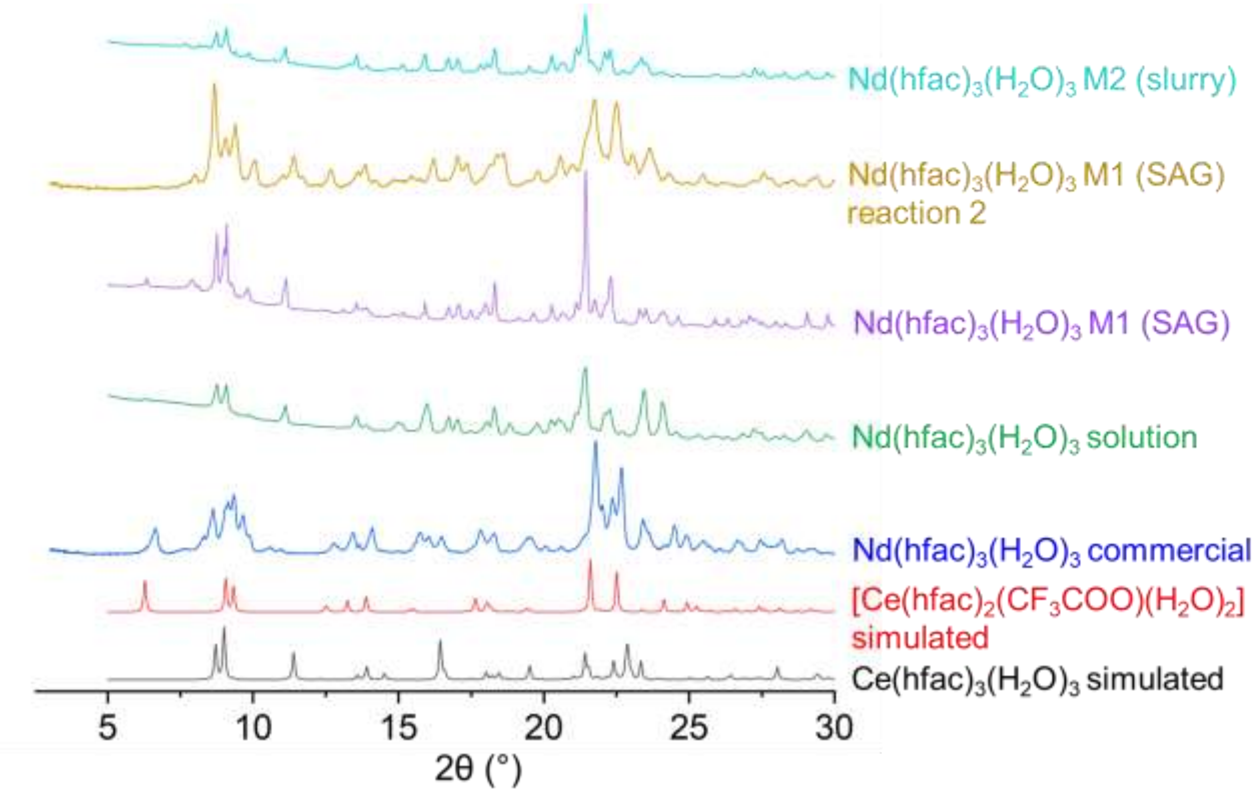


**Figure S96:** PXRD patterns for the various methods for synthesizing $Nd(hfac)_3(H_2O)_3$ including repeat reactions and comparison to simulated PXRD patterns (λ=1.54056 Å, $T$ = 123 K).

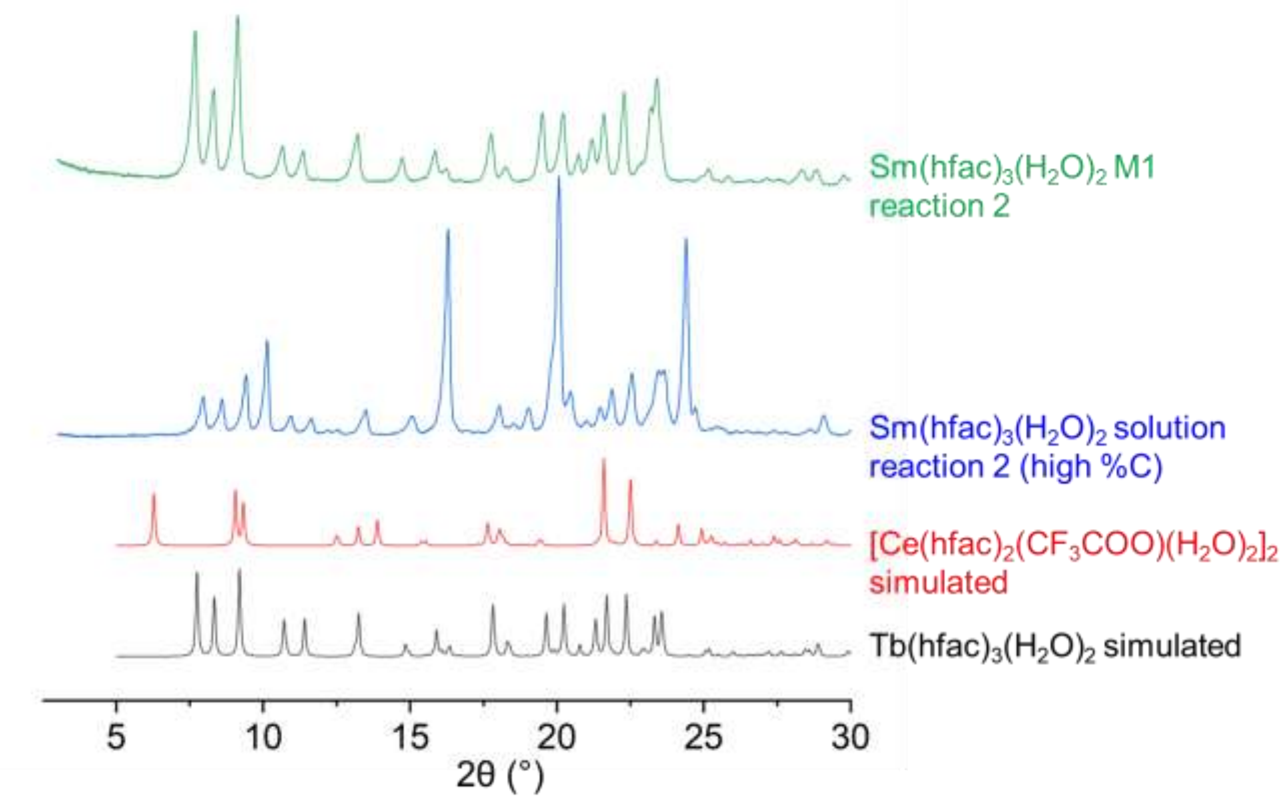


**Figure S97:** PXRD patterns of repeat reactions of solution and M1-SAG prepared $Sm(hfac)_3(H_2O)_2$ and comparison to simulated pattern for $Tb(hfac)_3(H_2O)_2$ (296 K) (Refcode: NALCEL)[15] and the Ce cluster (T=123 K, λ=1.54056 Å).

**S10: Crystallographic Details for Single Crystal Structure of $Ce(hfac)_3(H_2O)_3$**

For the complex $Ce(hfac)_3(H_2O)_3$, single crystals suitable for X-ray diffraction were grown by slow evaporation from a diethyl ether/hexanes solution. A yellow crystal (block, approximate dimensions 0.2 × 0.17 × 0.13 $mm^3$) was placed onto the tip of a MiTeGen pin and mounted on a Bruker Venture D8 diffractometer equipped with a PhotonIII detector at 123.0 K.

**Data collection**

The data collection was carried out using Mo Kα radiation (λ = 0.71073 Å, graphite monochromator) with a frame time of 2 seconds and a detector distance of 60 mm. A collection strategy was calculated and complete data to a resolution of 0.5 Å with a redundancy of 5.7 were collected. The frames were integrated with the Bruker SAINT[7] software package using a narrow-frame algorithm. The integration of the data using a monoclinic unit cell yielded a total of 111304

reflections to a maximum θ angle of 27.49° (0.77 Å resolution), of which 8970 were independent (average redundancy 12.408, completeness = 99.9%, $R_{int}$ = 4.06%, $R_{sig}$ = 1.90%) and 7858 (87.60%) were greater than $2\sigma(F^2)$. The final cell constants of a = 9.9693(7) Å, b = 38.163(3) Å, c = 10.3001(8) Å, β = 99.993(3)°, volume = 3859.3(5) Å$^3$, are based upon the refinement of the XYZ-centroids of reflections above 20 σ(I). Data were corrected for absorption effects using the Multi-Scan method (SADABS).[8] The calculated minimum and maximum transmission coefficients (based on crystal size) are 0.6970 and 0.7860.

**Structure solution and refinement**

The space group P1 21/m 1 was determined based on intensity statistics and systematic absences. The structure was solved using the SHELX suite of programs[9,10] and refined using full-matrix least-squares on $F^2$ within the OLEX2 suite.[11] An intrinsic phasing solution was calculated, which provided most non-hydrogen atoms from the E-map. Full-matrix least squares / difference Fourier cycles were performed, which located the remaining non-hydrogen atoms. All non-hydrogen atoms were refined with anisotropic displacement parameters. The hydrogen atoms were placed in ideal positions and refined as riding atoms with relative isotropic displacement parameters. The final full matrix least squares refinement converged to $R_1$ = 0.0631 and $wR_2$ = 0.1670 ($F^2$, all data). The goodness-of-fit was 1.031. On the basis of the final model, the calculated density was 2.105 g/cm$^3$ and F(000), 2346 $e^-$. The structure has commensurate modulation along the b axes. It was solved and refined in a supercell, with one and half molecules in the asymmetric unit (Z'=1.5). The modulation is particularly visible by looking at the Ce position along the *b* axes (following image **Figure S100**).

**Table S12. Crystal data and structure refinement for Ce(hfac)$_3$($H_2O$)$_3$ (mo_21017_0m_a).**

| | | |
|---|---|---|
| Empirical formula | C15 H9 Ce F18 O9 | |
| Formula weight | 815.34 | |
| Crystal color, shape, size | yellow block, 0.2 × 0.17 × 0.13 mm$^3$ | |
| Temperature | 123.0 K | |
| Wavelength | 0.71073 Å | |
| Crystal system, space group | Monoclinic, P 1 21/m 1 | |
| Unit cell dimensions | a = 9.9693(7) Å | α = 90°. |
| | b = 38.163(3) Å | β = 99.993(3)°. |
| | c = 10.3001(8) Å | γ = 90°. |
| Volume | 3859.3(5) Å$^3$ | |
| Z | 6 | |
| Density (calculated) | 2.105 g/cm$^3$ | |
| Absorption coefficient | 1.943 mm$^{-1}$ | |

| | |
|---|---|
| F(000) | 2346 |

**Data collection**

| | |
|---|---|
| Diffractometer | Bruker Venture D8 |
| Theta range for data collection | 2.008 to 27.489°. |
| Index ranges | -12<=h<=11, -49<=k<=49, -13<=l<=13 |
| Reflections collected | 111304 |
| Independent reflections | 8970 [$R_{int}$ = 0.0406] |
| Observed Reflections | 7858 |
| Completeness to theta = 25.242° | 99.9 % |

**Solution and Refinement**

| | |
|---|---|
| Absorption correction | Semi-empirical from equivalents |
| Max. and min. transmission | 0.7456 and 0.6783 |
| Solution | Intrinsic methods |
| Refinement method | Full-matrix least-squares on $F^2$ |
| Weighting scheme | w = $[\sigma^2 Fo^2 + AP^2 + BP]^{-1}$, with |
| | P = $(Fo^2 + 2 Fc^2)/3$, A = 0.0798, B = 41.288502 |
| Data / restraints / parameters | 8970 / 1223 / 564 |
| Goodness-of-fit on $F^2$ | 1.031 |
| Final R indices [I>2σ(I)] | R1 = 0.0631, wR2 = 0.1608 |
| R indices (all data) | R1 = 0.0707, wR2 = 0.1670 |
| Extinction coefficient | n/a |
| Largest diff. peak and hole | 1.992 and -2.416 e.Å$^{-3}$ |

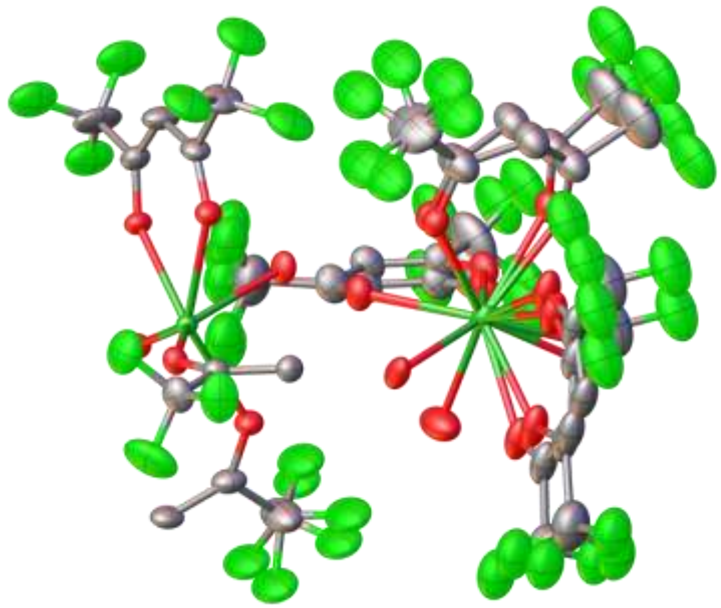

**Figure S98**: Asymmetric unit of $Ce(hfac)_3(H_2O)_3$ with ellipsoids plotted at 50% probability

**Table S13. Hydrogen bonds for $Ce(hfac)_3(H_2O)_3$ mo_21017_0m_a [Å and °].**

| D-H...A | d(D-H) | d(H...A) | d(D...A) | <(DHA) |
|---|---|---|---|---|
| O1-H1A...O11 | 0.80 | 2.19 | 2.96(4) | 163.4 |
| O1-H1A...F15 | 0.80 | 2.48 | 2.94(2) | 118.1 |
| O1-H1B...O4#1 | 0.94 | 2.27 | 2.881(6) | 121.7 |
| O1-H1B...O15 | 0.94 | 2.57 | 3.02(2) | 110.1 |
| O1-H1B...O7 | 0.94 | 2.33 | 2.941(7) | 122.0 |
| O7-H7A...F1 | 0.88 | 2.31 | 2.907(13) | 125.5 |
| O7-H7B...O3 | 0.88 | 2.27 | 2.978(7) | 137.2 |
| O7-H7B...O1 | 0.88 | 2.48 | 2.941(7) | 113.1 |
| O7-H7B...F2A | 0.88 | 2.58 | 3.23(2) | 132.1 |
| O8-H8A...O14#2 | 0.88 | 2.37 | 2.91(2) | 120.3 |
| O8-H8A...O8#2 | 0.88 | 2.33 | 3.13(5) | 152.1 |
| O8-H8B...F24#2 | 0.88 | 2.49 | 3.17(3) | 133.6 |
| O8-H8B...F23#2 | 0.88 | 2.40 | 3.21(3) | 153.4 |
| O9-H9A...F2 | 0.87 | 2.18 | 3.029(15) | 166.3 |
| O9-H9A...F2A | 0.87 | 2.05 | 2.912(19) | 167.8 |
| O8A-H8AA...O14A#2 | 0.91 | 2.38 | 3.02(3) | 128.0 |
| O8A-H8AA...F24A#2 | 0.91 | 2.55 | 3.41(4) | 159.3 |
| O8A-H8AB...F12A#2 | 0.91 | 2.42 | 3.18(4) | 142.0 |

Symmetry transformations used to generate equivalent atoms:
#1 x,-y+1/2,z    #2 -x+2,-y+1,-z+2

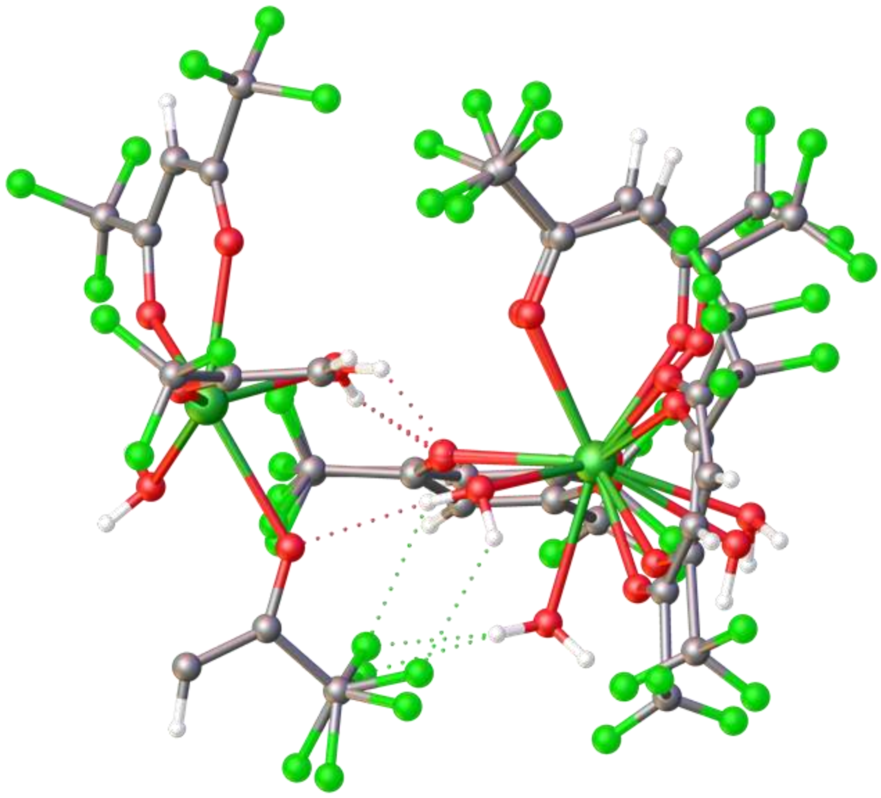

**Figure S99**: Hydrogen atoms and hydrogen bonding between molecules of $Ce(hfac)_3(H_2O)_3$.

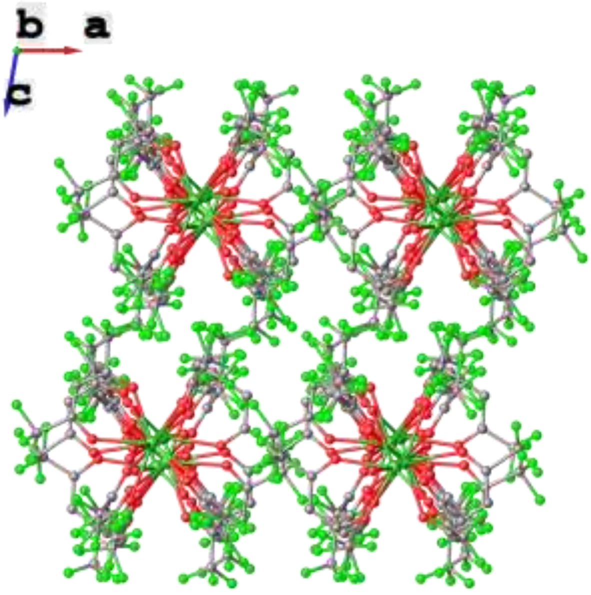


**Figure S100**: View of packing of $Ce(hfac)_3(H_2O)_3$ along the *b* axis.

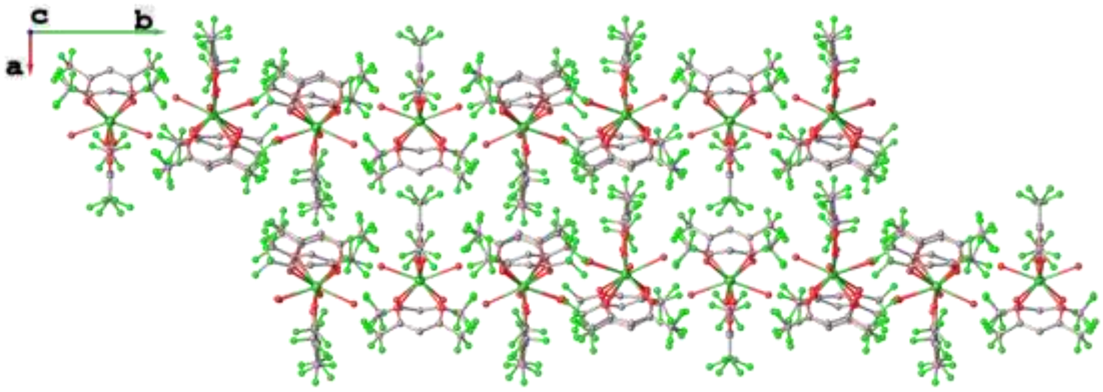


**Figure S101**: View of packing of $Ce(hfac)_3(H_2O)_3$ along the *c* axis.

**The $Ce(hfac)_3(H_2O)_3$ crystal: bulk analysis of crystal batches**

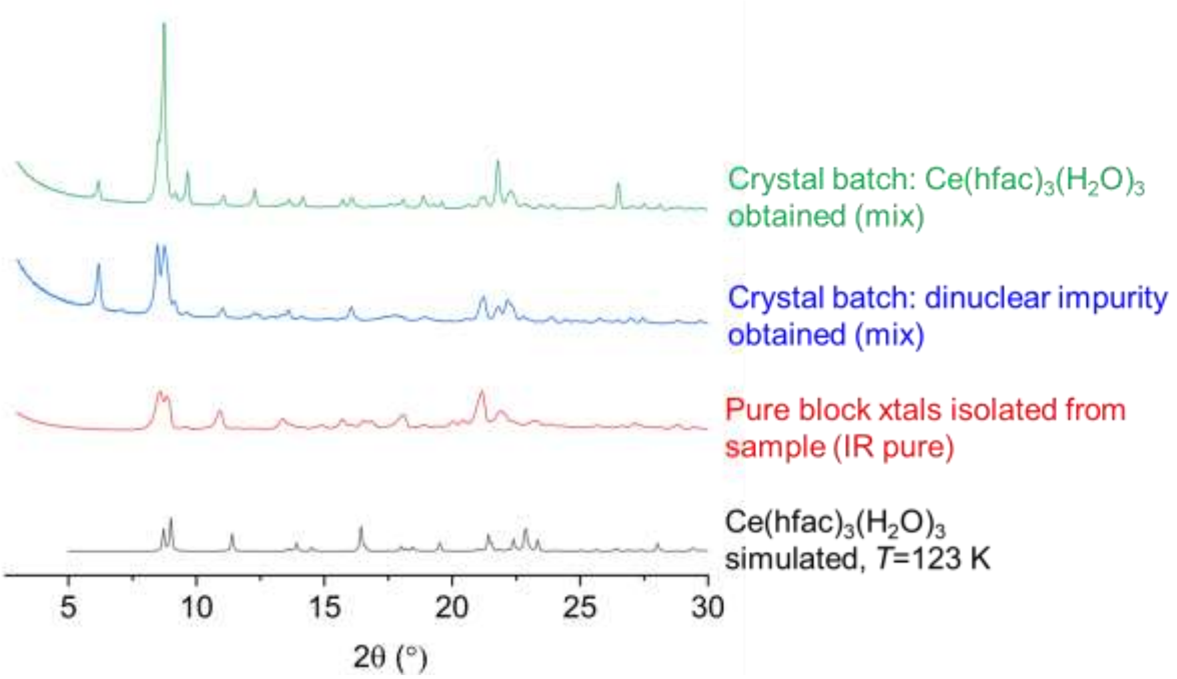


**Figure S102:** Comparison of PXRD patterns of simulated $Ce(hfac)_3(H_2O)_3$ (λ=1.54056 Å) to pure block crystals and sample batches from which the $Ce(hfac)_3(H_2O)_3$ crystal and a crystal of the dinuclear impurity were obtained.

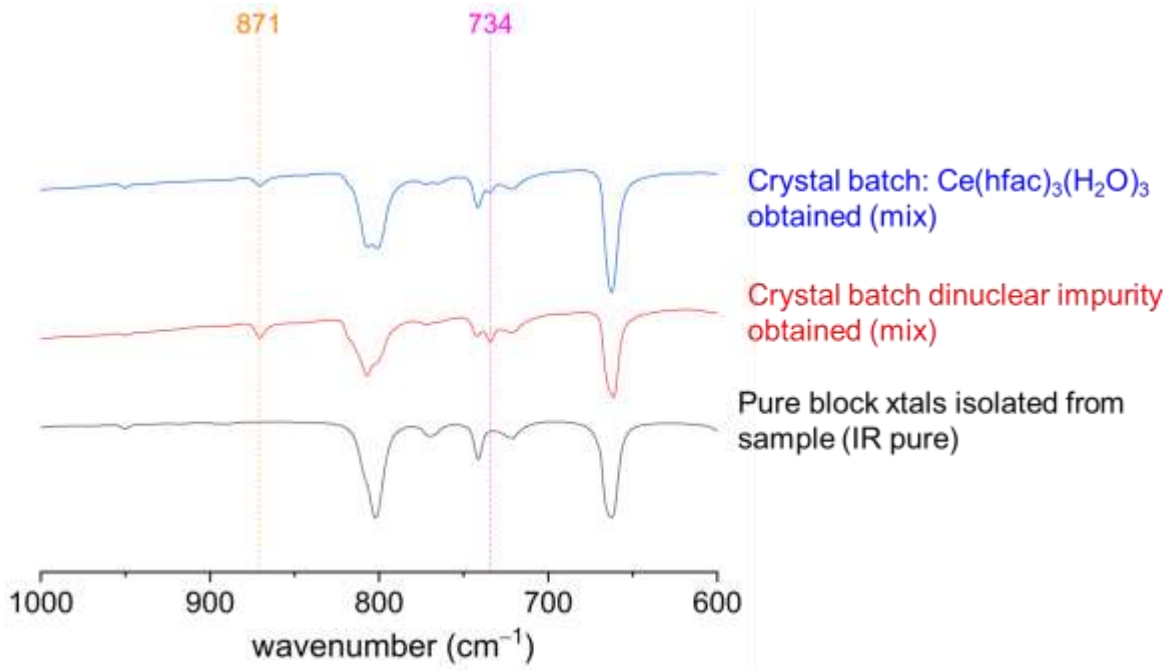


**Figure S103:** Comparison of FT-IR spectra of $Ce(hfac)_3(H_2O)_3$ pure block crystals (seen in Figure S62) and sample batches from which the $Ce(hfac)_3(H_2O)_3$ crystal and a crystal of the dinuclear impurity were obtained. (Nujol, KBr, 2 cm$^{-1}$ res. 32 scans)

**S11: Reactions of the mid-lanthanides $TbCl_3·6H_2O$**

**$Tb(hfac)_3(H_2O)_2$ solution synthesis:**

In an Erlenmeyer flask, $Na_2CO_3·H_2O$ (0.4841 g, 3.882 mmol) was dissolved in 75 mL of deionized $H_2O$. To the solution was added Hhfac (1.1 mL, 7.9 mmol) with 10 mL of DI $H_2O$ to wash in Hhfac. The reaction was stirred for five minutes until bubbling ceased. To this solution was added $TbCl_3·6H_2O$ (1.0035 g, 2.6876 mmol) with an additional 75 mL of DI $H_2O$ then added to bring everything in solution. The solution was stirred for four hours, then extracted with (3 × 100 mL) of $Et_2O$. The material was then recrystallized with 50/50 *v*/*v* $Et_2O$/hexanes. The yield was 1.6586 g (2.0324 mmol, 76%).

IR data: 3503m-br, 3416m-sh, 3298mw-sh, 3153w, 3087vw, 3060vw, 1657s-sh, 1649s, 1619m, 1568m, 1541ms, 1492s-sh, 1466*s, 1457s-sh, 1364w, 1353mw, 1328w, 1256s, 1227s-br, 1204s, 1147vs-br, 1112m, 1101ms, 951vw, 917vvw, 890vvw, 810ms, 774vw, 744mw, 664ms.

**Tb(hfac)$_3$(H$_2$O)$_2$ M1-SAG synthesis:**

In a porcelain mortar, $Na_2CO_3 \cdot H_2O$ (0.4715 g, 3.802 mmol) was ground into a fine powder. To the thin film of material, Hhfac was syringed in (1.1 mL, 7.9 mmol) and ground for ten minutes until a fine powder. To this solid, $TbCl_3 \cdot 6H_2O$ (1.0457 g, 2.8006 mmol) and ground for 15 minutes until a fine white powder and extracted with 2×20 mL of ether. To this solution was added 20 mL of hexanes to facilitate solid formation. The yield was 0.7509 g (33%, 0.9201 mmol) of light yellow-green solid. Elem. Anal. Calcd. for $TbC_{15}H_8F_{18}O_8$: %C 22.08, %H 0.86. Found %C 22.22, %H 0.89.

IR data: 3596mw-sh, 3493m-br, 3265mw-sh, 3153w, 3087vw, 3060vw, 1649s, 1620m, 1568m, 1541ms, 1492s-sh, 1470s, 1459*s-br, 1434m-sh, 1353mw, 1327mw, 1258s, 1227s-br, 1203s, 1149vs-br, 1102ms, 952vw, 890vvw, 868vvw-br, 811ms, 773vw, 744mw, 664s.

**Tb(hfac)$_3$(H$_2$O)$_2$ M1-SAG synthesis 2:**

In a porcelain mortar, $Na_2CO_3 \cdot H_2O$ (0.5279 g, 4.257 mmol) was ground into a fine powder. To the thin film of material, Hhfac was syringed in (1.3 mL, 9.3 mmol) and ground for 10 minutes until a fine powder. To this solid, $TbCl_3 \cdot 6H_2O$ (1.0689 g, 2.8628 mmol) and ground for 10 minutes until a fine white powder and extracted with 3×20 mL of ether. The yield was 1.0598 g (45%, 1.2986 mmol). Elem. Anal. Calcd. for $TbC_{15}H_8F_{18}O_8$: %C 22.08, %H 0.86. Found %C 22.04, %H 0.82.

IR (Nujol, KBr): 3659w-sh, 3594mw-sh, 3489m-br, 3254w-sh, 3152w, 3084vw, 3064vw, 1699mw, 1650s, 1620m, 1568m, 1541m, 1475ms-br, 1456s-sh, 1435m-sh, 1366w, 1352w, 1327w, 1260s, 1227s- br, 1209s, 1150vs-sh, 1111mw-sh, 1101ms, 1060vvw, 1025vvw, 1003vvw, 97v2vw, 951vw, 920vvw, 890vvw, 849vw, 811ms, 774vw, 744mw, 665s, 615vvw.

**Tb(hfac)$_3$(H$_2$O)$_2$ M1-SAG synthesis 3:**

In a porcelain mortar, $Na_2CO_3 \cdot H_2O$ (0.5685 g, 4.585 mmol) was ground into a fine powder. To the thin film of material, Hhfac was syringed in (1.4 mL, 10 mmol) and ground for 5 minutes until a fine powder. To this solid, $TbCl_3 \cdot 6H_2O$ (1.1476 g, 3.0735 mmol) and ground for 15 minutes until a fine white powder and extracted with 3×20 mL of ether. The yield was 0.8477 g (34%, 1.039 mmol). Elem. Anal. Calcd. for $TbC_{15}H_8F_{18}O_8$: %C 22.08, %H 0.86. Found %C 21.89, %H 0.75.

IR (Nujol, KBr): 3661vw-sh, 3595w-sh, 3476mw-br, 3258mw-sh, 3151w, 3086vw, 3064vw, 1699w-sp, 1649s, 1619m, 1567m, 1541m, 1475*s-br, 1456s-sh, 1435mw-sh, 1366w-sh, 1352w, 1327w, 1259s, 1226s-br, 1204s-sh, 1150vs-sh, 1112m-sh, 1101ms, 951vw, 849vw, 810ms, 774vw, 744mw, 664s.

**$Tb(hfac)_3(H_2O)_2$ M1-SAG synthesis 4:**

In a porcelain mortar, $Na_2CO_3 \cdot H_2O$ (0.4410 g, 3.556 mmol) was ground into a fine powder. To the thin film of material, Hhfac was syringed in (1.0 mL, 7.2 mmol) and ground until a fine powder for five minutes. To this solid, $TbCl_3 \cdot 6H_2O$ (0.8913 g, 2.387 mmol) and ground for 20 minutes until a fine white powder and extracted with 3×10 mL of $Et_2O$. To this solution was added 10 mL of hexanes to facilitate solid formation. The yield was 0.8975 g (1.100 mmol, 46%) of light green solid. Elem. Anal. Calcd. for $TbC_{15}H_8F_{18}O_8$: %C 22.08, %H 0.86. Found %C 21.94, %H 0.81.

IR data: 3596mw-sh, 3493m-br, 3265mw-sh, 3153w, 3087vw, 3060vw, 1649s, 1620m, 1568 m, 1541ms, 1492s-sh, 1470s, 1459*s-br, 1434m-sh, 1353mw, 1327mw, 1258s, 1227s-br, 1203s, 1149vs-br, 1102ms, 952vw, 890vvw, 868vvw, 811ms, 773vw, 744mw, 664s.

**$Tb(hfac)_3(H_2O)_2$ prepared from modified M2 reaction:**

In a porcelain mortar, $Na_2CO_3 \cdot H_2O$ (1.0726 g, 8.65 mmol) was ground into a fine powder. To the thin film of material, Hhfac was syringed in (2.4 mL, 17 mmol) and ground for five minutes until a fine powder. To this solid was added 10 mL of $Et_2O$ followed by the addition of, $TbCl_3 \cdot 6H_2O$ (2.1623 g, 5.79 mmol) and ground for 30 minutes. The material was then left to sit overnight. The material was extracted with 5×20 mL of ether. Hexanes (20 mL) was added to facilitate solid formation. The yield was 2.10 g (2.57 mmol, 44 % yield) of light-yellow solid. Elem. Anal. Calcd. for $TbC_{15}H_8F_{18}O_8$: %C 22.08, %H 0.86. Found %C 21.82, %H 0.74.

IR: *3664*mw-sh, 3599m-sh, 3484m-br, 3261mw-sh, 3153w, 3086vw, 3065vw, 1649s, 1619ms, 1568m, 1541ms, 1467*s, 1458*s, 1434m, 1364w, 1353w, 1327w, 1257s, 1226s, 1203s, 1148vs, 1110m, 1102m, 953vw, 917vw, 893vw, *867*w, 811s, 774w, 744mw, *733*w, 664s.

**Reaction of $TbCl_3 \cdot 6H_2O$ and Recrystallized Na(hfac):**

To a porcelain mortar, in the fumehood, $Na_2CO_3 \cdot H_2O$ (1.335 g, 10.77 mmol) was added and ground lightly until powdery. Hhfac (3.0 mL, 21 mmol) was added and ground for 15 minutes

until a powdery solid. The solid was dissolved in acetone. Insoluble inorganic material was filtered out. The product crystallized out of acetone as a white solid. The yield of white crystalline material was 58% (2.8245 g, 12.278 mmol). $^1$H NMR ($d_6$-acetone, 298 K): 5.59 (s, hfac-H), 3.08 (s, $H_2O$) ppm. $^{19}$F NMR ($d_6$-acetone, 298 K, unreferenced): −75.87 (minor), −77.56 ppm. $^{13}$C NMR ($d_6$-acetone, 298 K): 175.0 (q, $^2$JC-F=30 Hz, hfac), 119.0 (q, $^1$JC-F=288 Hz, hfac), 85.1 (hfac), 161.7 (q, $^2J_{C\text{-}F}$=33 Hz, tfa) ppm.

A porcelain mortar was charged with recrystallized Na(hfac) (0.6040 g, 2.626 mmol), $TbCl_3{\cdot}6H_2O$ (0.3271 g, 0.8761 mmol) was then added and ground for 20 minutes. The product was extracted with 3×15 mL of $Et_2O$. To the $Et_2O$ extract, 5 mL of hexanes was added to facilitate formation of a pale beige solid (0.5495 g, 0.6733 mmol) with a 77% yield. FT-IR (Nujol, cm$^{-1}$): *3662vw-sp*, 3591mw, 3489m-br, 3259w-br, 3152vw, 1649s, 1620m, 1568m, 1541ms, 1467ms-sh, 1459s-br*, 1365w, 1353w, 1328vw, 1259s, 1228s, 1205ms, 1151vs, 1110mw, 1102m, 951vw, *868w*, 811m, 774w-br, 744mw, *733w*, 664ms. Elem. Anal. Calcd. for $TbC_{15}H_7F_{18}O_8$: %C 22.08, %H 0.86. Found %C 22.33, %H 0.89.

**Ball milling Reactions of $TbCl_3{\cdot}6H_2O$:**

An example of a ball mill reaction with $TbCl_3{\cdot}6H_2O$ proceeds as follows: to a 50 mL YSZ container, $Na_2CO_3{\cdot}H_2O$ (0.4464 g, 3.600 mmol) and Hhfac (1.0 mL, 7.2 mmol) was added and ball milled with 18 g of 3 mm YSZ-balls for 20 min at 500 rpm. $TbCl_3{\cdot}6H_2O$ (0.8922 g, 2.389 mmol) was added and ball milled for one hour at 500 rpm. The material was extracted with 3×10 mL of $Et_2O$. The yield of light green crystalline material was 1.6935 g. This material contains tetraol impurity in addition to $Tb(hfac)_3(H_2O)_2$ (**Figure S104**).

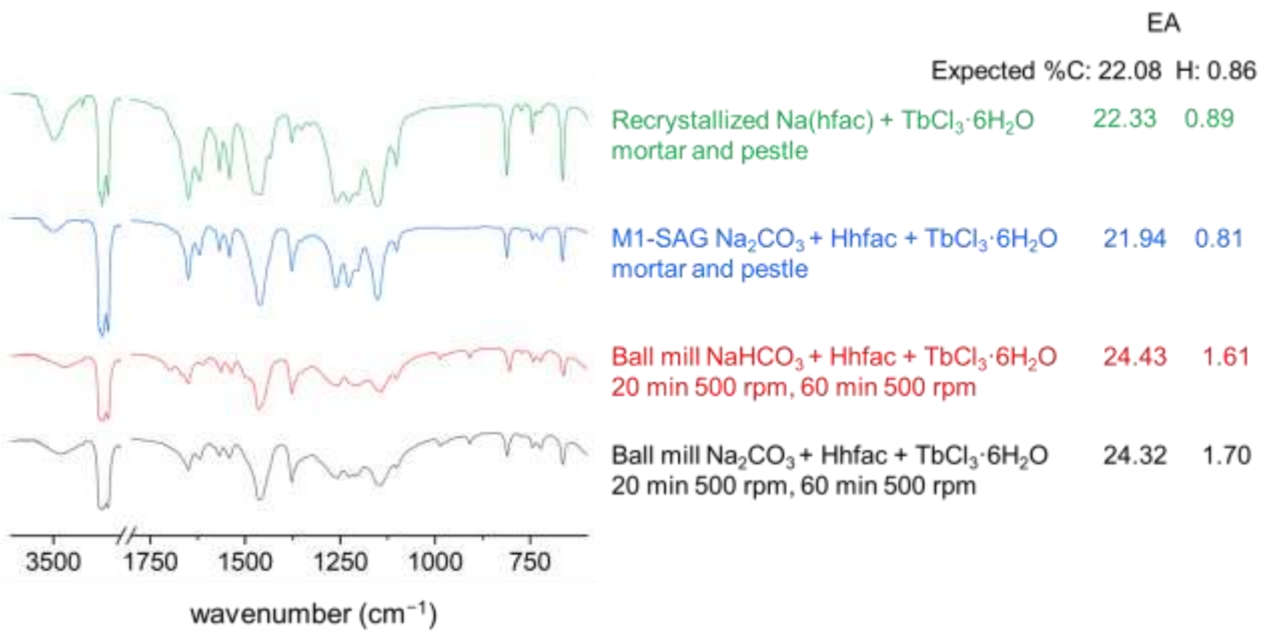


**Figure S104:** Comparison of FT-IR spectra of mechanochemical reactions to prepare $Tb(hfac)_3(H_2O)_2$. (Nujol, KBr plates, 2 $cm^{-1}$ res.). Third IR spectrum from bottom is from main text Figure 9b (M1-SAG #4). Note: Ball mill reactions were not placed under vacuum.

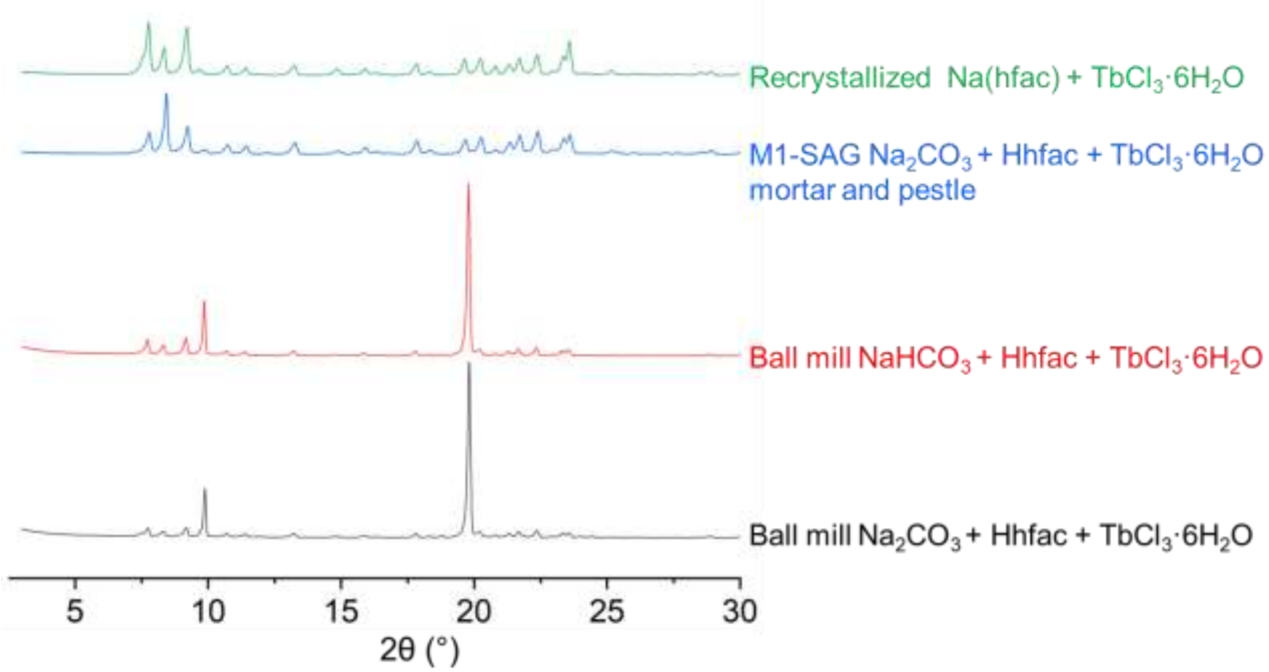


**Figure S105:** Comparison of XRD diffraction patterns of mechanochemical reactions to prepare $Tb(hfac)_3(H_2O)_2$. Third diffraction pattern is from main text Figure 9a (M1-SAG #4) for comparison purposes. Note: Ball mill reactions were not placed under vacuum.

**S12. References:**

(1) Richardson, M. F.; Wagner, W. F.; Sands, D. E. Rare-earth trishexafluoroacetylacetonates and related compounds. *J. Inorg. Nucl. Chem.* **1968**, *30*, 1275-1289.

(2) Fatila, E. M.; Hetherington, E. E.; Jennings, M.; Lough, A. J.; Preuss, K. E. Syntheses and crystal structures of anhydrous Ln(hfac)$_3$(monoglyme). Ln = La, Ce, Pr, Sm, Eu, Gd, Tb, Dy, Er, Tm. *Dalton Trans.* **2012**, *41*, 1352-1362.

(3) Fulmer, G. R.; Miller, A. J. M.; Sherden, N. H.; Gottlieb, H. E.; Nudelman, A.; Stoltz, B. M.; Bercaw, J. E.; Goldberg, K. I. NMR Chemical Shifts of Trace Impurities: Common Laboratory Solvents, Organics, and Gases in Deuterated Solvents Relevant to the Organometallic Chemist. *Organometallics* **2010**, *29*, 2176-2179.

(4) Macrae, C. F.; Edgington, P. R.; McCabe, P.; Pidcock, E.; Shields, G. P.; Taylor, R.; Towler, M.; van de Streek, J. Mercury: visualization and analysis of crystal structures. *J. Appl. Crystallogr.* **2006**, *39*, 453-457.

(5) Farrugia, L. ORTEP-3 for Windows - a version of ORTEP-III with a Graphical User Interface (GUI). *J. Appl. Crystallogr.* **1997**, *30*, 565.

(6) Persistence of Vision Raytracer. Version 3.6 ed.; Persistence of Vision Pty. Ltd.: http://www.povray.org/download/, 2004.

(7) SAINT. V8.30A ed.; Bruker Analytical X-Ray Systems Madison, WI, 2012.

(8) SADABS. 2.03 ed.; Bruker Analytical X-Ray Systems: Madison, WI, 2016.

(9) Sheldrick, G. A short history of SHELX. *Acta Crystallogr. A* **2008**, *64*, 112-122.

(10) Sheldrick, G. Crystal structure refinement with SHELXL. *Acta Crystallogr. C* **2015**, *71*, 3-8.

(11) Dolomanov, O. V.; Bourhis, L. J.; Gildea, R. J.; Howard, J. A. K.; Puschmann, H. OLEX2: a complete structure solution, refinement and analysis program. *J. Appl. Crystallogr.* **2009**, *42*, 339-341.

(12) Aygen, S.; van Eldik, R. A Spectroscopic and Mechanistic Study of the Enolization and Diol Formation of Hexafluoroacetylacetone in the Presence of Water and Alcohol. *Chem. Ber.* **1989**, *122*, 315-320.

(13) Belford, R.; Martell, A.; Calvin, M. Influence of Fluorine Substitution on the Properties of Metal Chelate Compounds. I. Copper (II) Chelates of Bidentate Ligands. *Lawrence Berkeley National Laboratory* *https://escholarship.org/uc/item/9t55q385* **1955**.

(14) Rogachev, A. Y.; Minacheva, L. K.; Sergienko, V. S.; Kuz'mina, N. P. Synthesis and crystal structure of mixed-ligand complex [La(hfa)$_2$(μ-$O_2CCF_3$)($H_2O$)$_2$]$_2$. *Russ. J. Inorg. Chem.* **2004**, *49*, 1814-1821.

(15) Bagryanskaya, I. Y.; Politanskaya, L. V.; Tretyakov, E. V. Frequently used, but still unknown: Terbium(III) tris-hexafluoroacetylacetonate dihydrate. *Inorg. Chem. Commun.* **2016**, *66*, 47-50.
